# Supplementary material for: Macronuclear Genome Sequence of the Ciliate Tetrahymena thermophila, a Model Eukaryote
Source: PLoS Biol. 2006 Aug 29;4(9):e286. doi: 10.1371/journal.pbio.0040286 (PMC1557398; doi:10.1371/journal.pbio.0040286)
Supplement: Supplementary file 1 [file pbio.0040286.sg001.pdf]

A

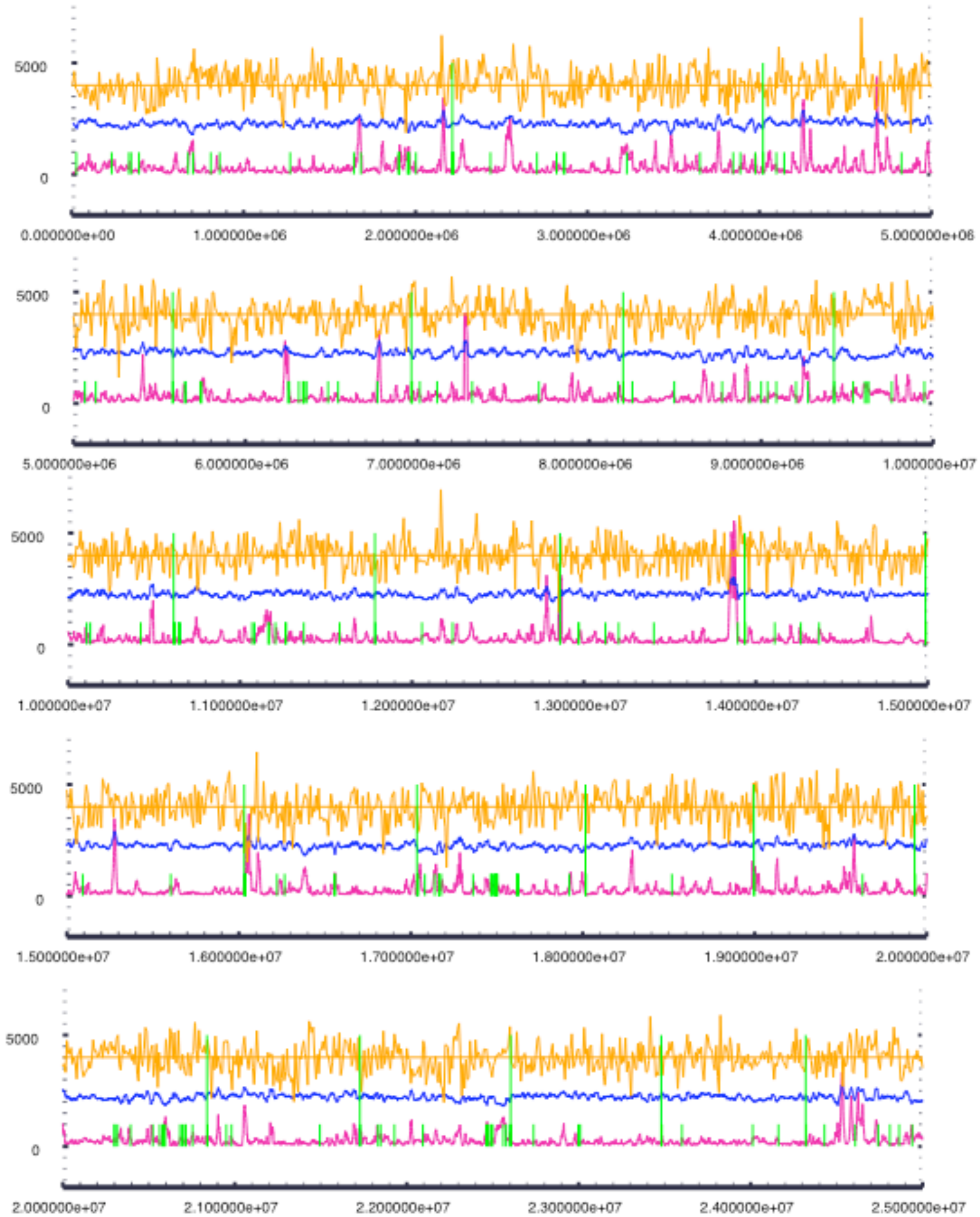
5000
0
0.000000e+00
1.000000e+06
2.000000e+06
3.000000e+06
4.000000e+06
5.000000e+06
5000
0
5.000000e+06
6.000000e+06
7.000000e+06
8.000000e+06
9.000000e+06
1.000000e+07
5000
0
1.000000e+07
1.100000e+07
1.200000e+07
1.300000e+07
1.400000e+07
1.500000e+07
5000
0
1.500000e+07
1.600000e+07
1.700000e+07
1.800000e+07
1.900000e+07
2.000000e+07
5000
0
2.000000e+07
2.100000e+07
2.200000e+07
2.300000e+07
2.400000e+07
2.500000e+07

B

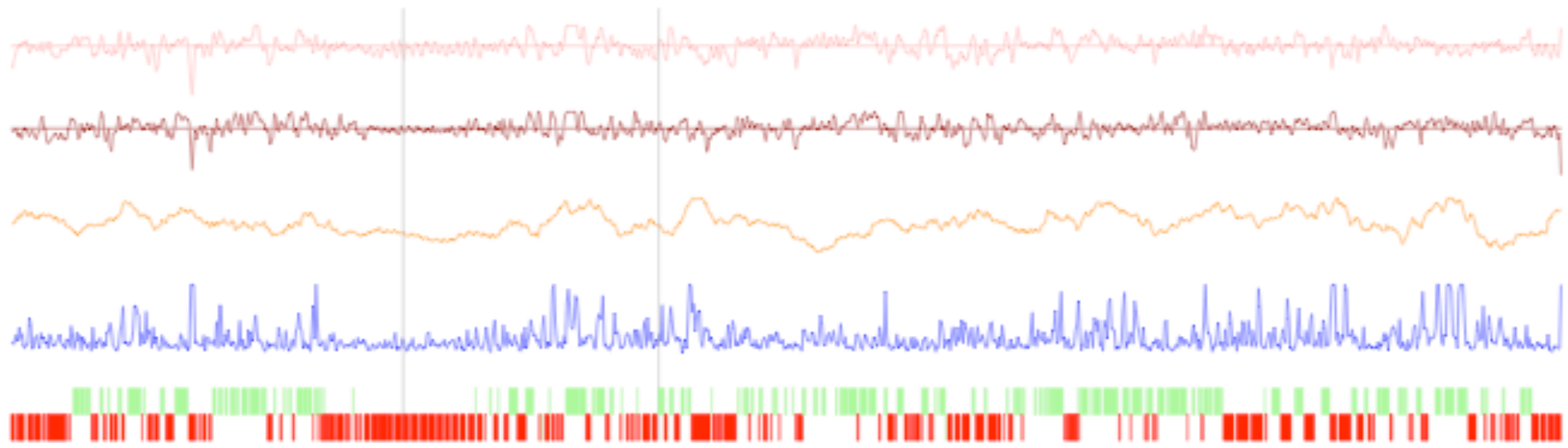

scaffold: 8254645

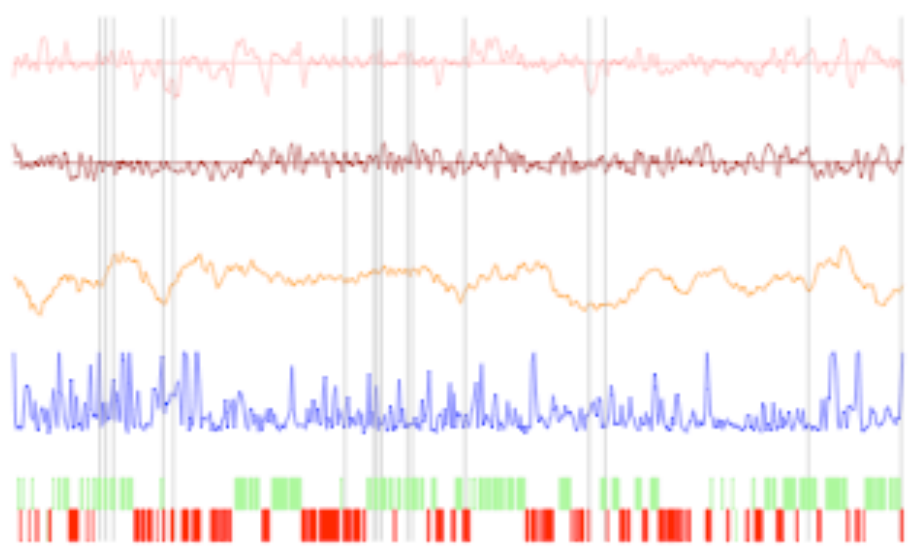

scaffold: 8254654

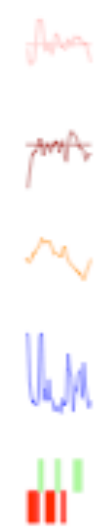

scaffold: 8254072
