# Supplementary material for: Macronuclear Genome Sequence of the Ciliate Tetrahymena thermophila, a Model Eukaryote
Source: PLoS Biol. 2006 Aug 29;4(9):e286. doi: 10.1371/journal.pbio.0040286 (PMC1557398; doi:10.1371/journal.pbio.0040286)
Supplement: Supplementary file 2 [file pbio.0040286.sg002.pdf]

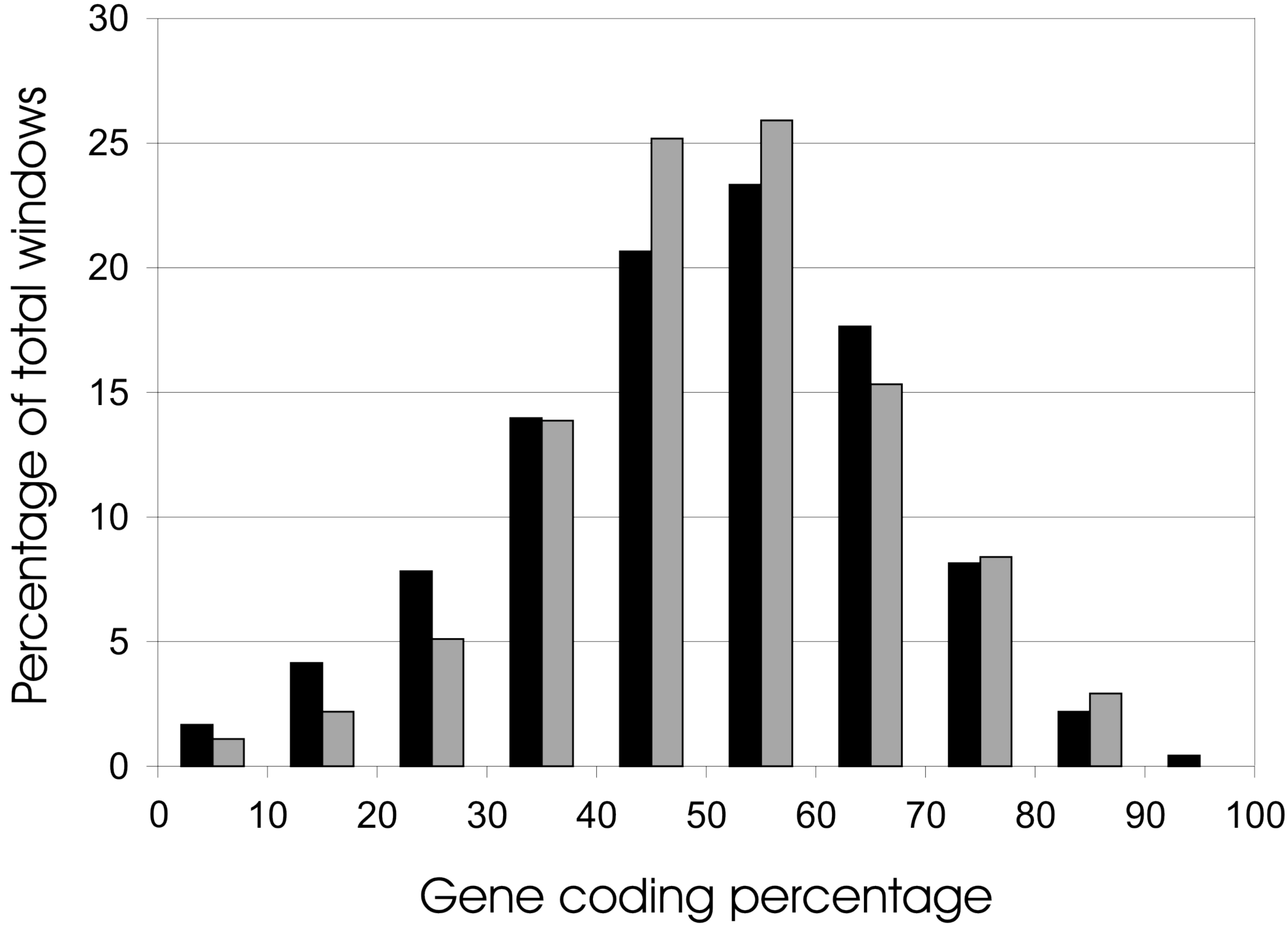

Percentage of total windows
30
25
20
15
10
5
0
0
10
20
30
40
50
60
70
80
90
100
Gene coding percentage
