# Supplementary material for: Macronuclear Genome Sequence of the Ciliate Tetrahymena thermophila, a Model Eukaryote
Source: PLoS Biol. 2006 Aug 29;4(9):e286. doi: 10.1371/journal.pbio.0040286 (PMC1557398; doi:10.1371/journal.pbio.0040286)
Supplement: Supplementary file 3 [file pbio.0040286.sg003.pdf]

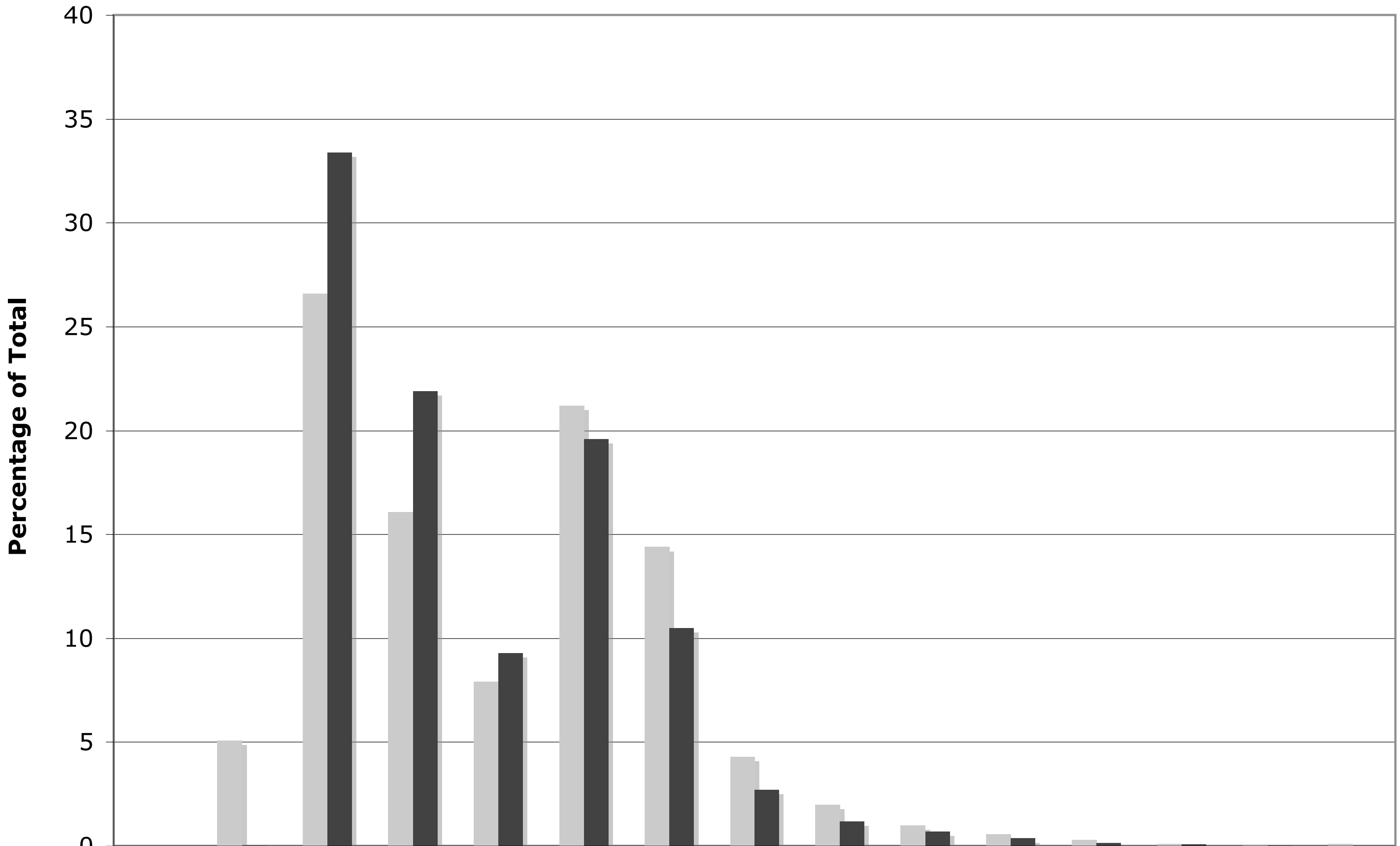

	0-20	21-40	41-60	61-80	81-100	101-200	201-400	401-600	601-800	801-1000	1001-1200	1201-1400	1401-1600	1601-1700	1701-3116
Predicted	0	5.1	26.6	16.1	7.9	21.2	14.4	4.3	2.0	1.0	0.58	0.3	0.12	0.05	0.1
EST-Confirmed	0	0.03	33.4	21.9	9.3	19.6	10.5	2.7	1.2	0.7	0.39	0.15	0.09	0.03	0.008

Intron Size (nt)
