# Supplementary material for: Macronuclear Genome Sequence of the Ciliate Tetrahymena thermophila, a Model Eukaryote
Source: PLoS Biol. 2006 Aug 29;4(9):e286. doi: 10.1371/journal.pbio.0040286 (PMC1557398; doi:10.1371/journal.pbio.0040286)
Supplement: Supplementary file 4 [file pbio.0040286.sg004.pdf]

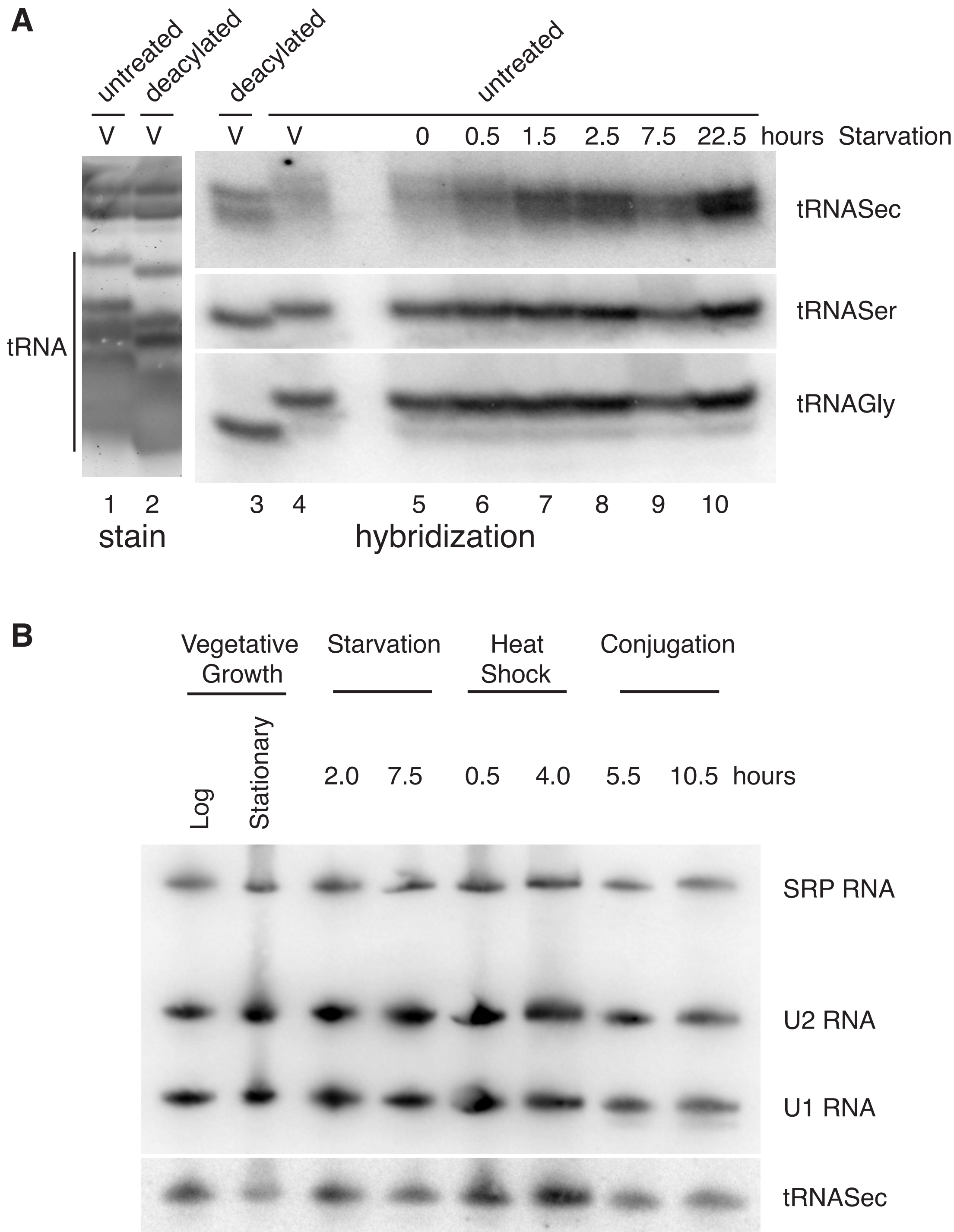
A
untreated
deacylated
deacylated
untreated
V
V
V
V
0
0.5
1.5
2.5
7.5
22.5
hours Starvation
tRNASec
tRNASer
tRNAGly
tRNA
1
2
3
4
5
6
7
8
9
10
stain
hybridization
B
Vegetative Growth
Starvation
Heat Shock
Conjugation
Log
Stationary
2.0
7.5
0.5
4.0
5.5
10.5
hours
SRP RNA
U2 RNA
U1 RNA
tRNASec
