# Supplementary material for: Macronuclear Genome Sequence of the Ciliate Tetrahymena thermophila, a Model Eukaryote
Source: PLoS Biol. 2006 Aug 29;4(9):e286. doi: 10.1371/journal.pbio.0040286 (PMC1557398; doi:10.1371/journal.pbio.0040286)
Supplement: Supplementary file 5 [file pbio.0040286.sg005.pdf]

Figure S5

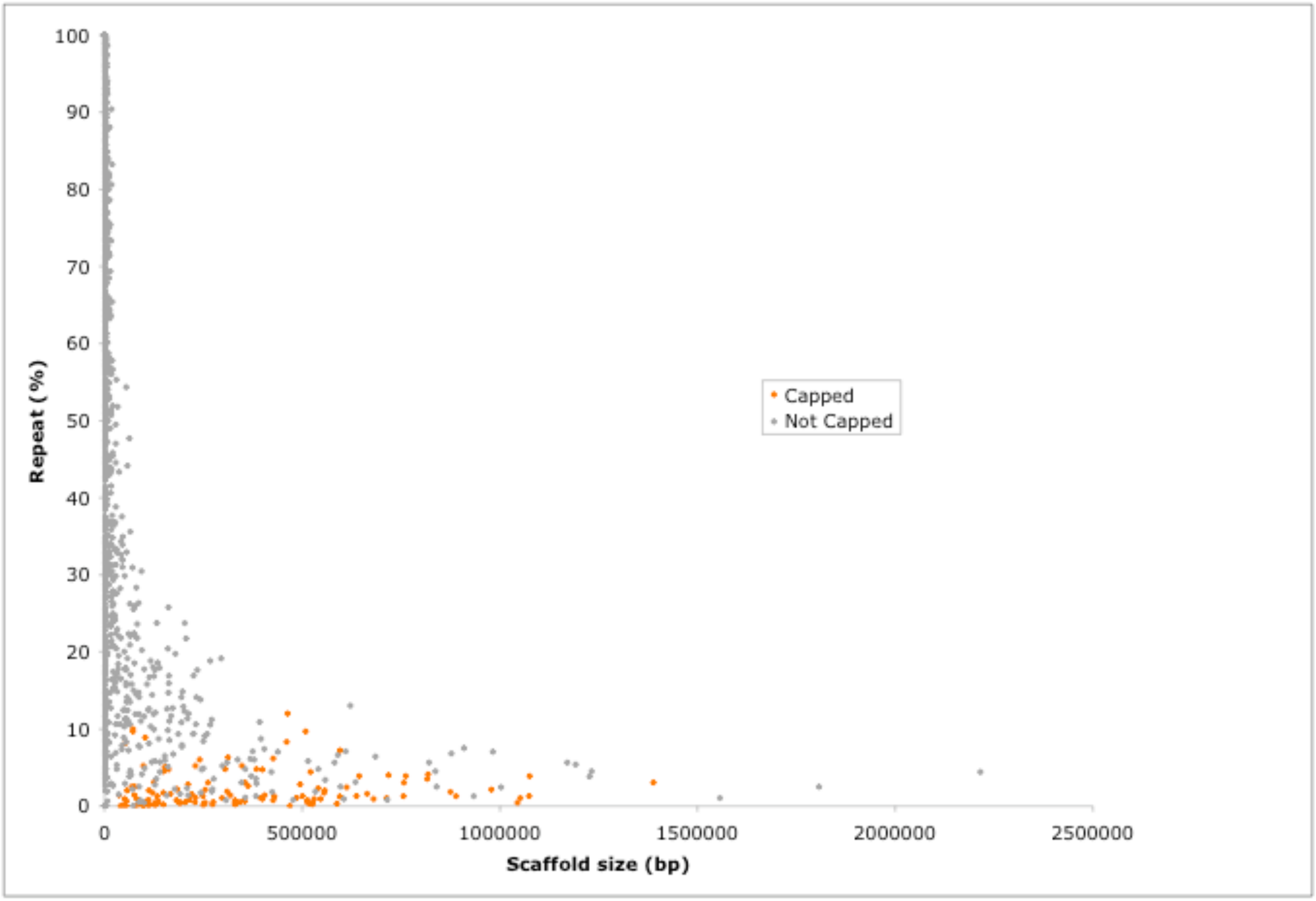

100
90
80
70
60
50
40
30
20
10
0
Repeat (%)
0
500000
1000000
1500000
2000000
2500000
Scaffold size (bp)
Capped
Not Capped
