# Supplementary material for: Macronuclear Genome Sequence of the Ciliate Tetrahymena thermophila, a Model Eukaryote
Source: PLoS Biol. 2006 Aug 29;4(9):e286. doi: 10.1371/journal.pbio.0040286 (PMC1557398; doi:10.1371/journal.pbio.0040286)
Supplement: Supplementary file 6 [file pbio.0040286.sg006.pdf]

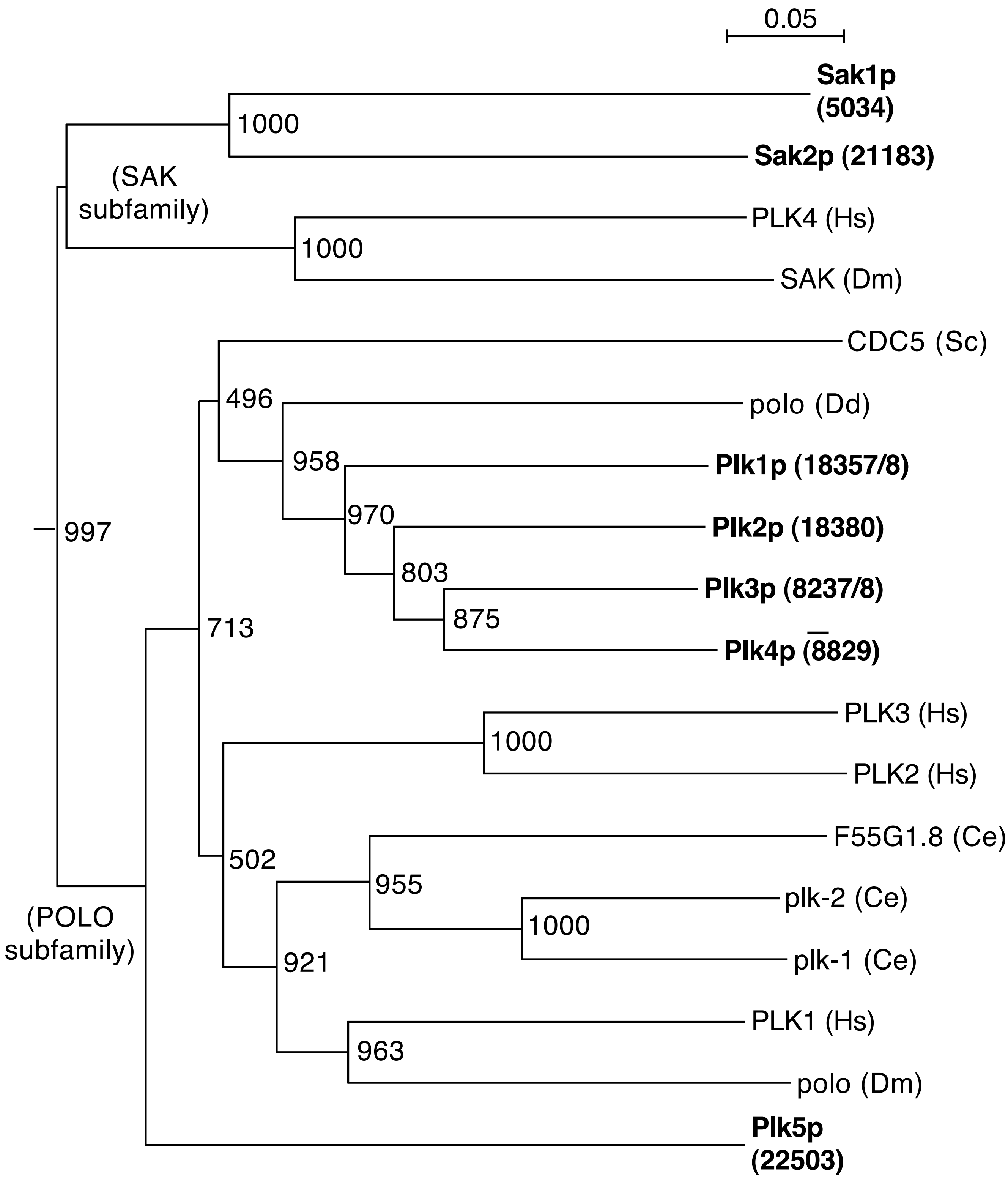
0.05
Sak1p (5034)
Sak2p (21183)
PLK4 (Hs)
SAK (Dm)
CDC5 (Sc)
polo (Dd)
Plk1p (18357/8)
Plk2p (18380)
Plk3p (8237/8)
Plk4p (8829)
PLK3 (Hs)
PLK2 (Hs)
F55G1.8 (Ce)
plk-2 (Ce)
plk-1 (Ce)
PLK1 (Hs)
polo (Dm)
Plk5p (22503)
1000
1000
496
958
970
803
875
997
713
1000
502
955
1000
921
963
(SAK subfamily)
(POLO subfamily)
