# Supplementary material for: Macronuclear Genome Sequence of the Ciliate Tetrahymena thermophila, a Model Eukaryote
Source: PLoS Biol. 2006 Aug 29;4(9):e286. doi: 10.1371/journal.pbio.0040286 (PMC1557398; doi:10.1371/journal.pbio.0040286)
Supplement: Supplementary file 7 [file pbio.0040286.sg007.pdf]

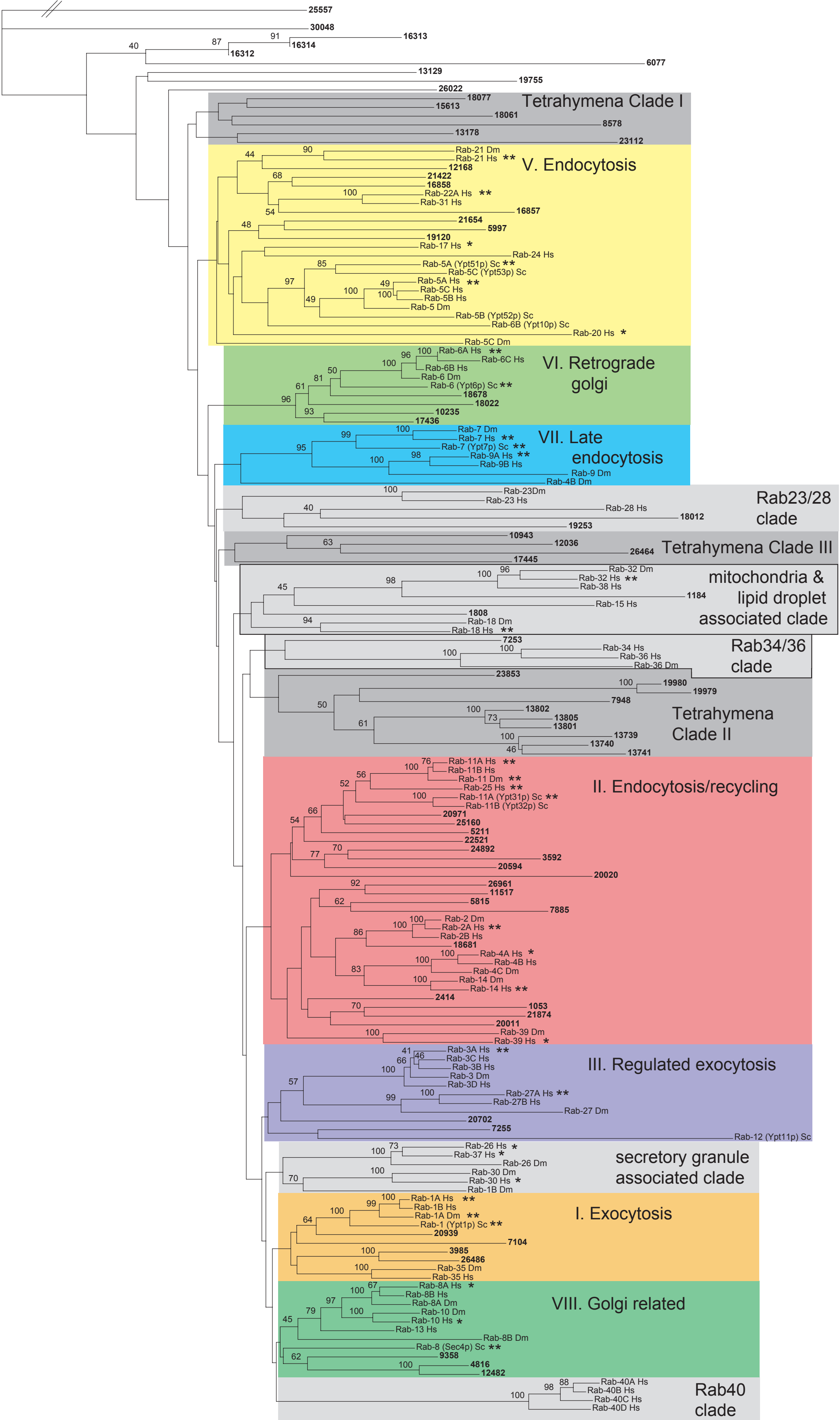
25557
30048
16313
16314
16312
6077
13129
19755
26022
Tetrahymena Clade I
18077
15613
18061
8578
13178
23112
V. Endocytosis
Rab-21 Dm
Rab-21 Hs **
12168
21422
16858
Rab-22A Hs **
Rab-31 Hs
16857
21654
5997
19120
Rab-17 Hs *
Rab-24 Hs
Rab-5A (Ypt51p) Sc **
Rab-5C (Ypt53p) Sc
Rab-5A Hs **
Rab-5C Hs
Rab-5B Hs
Rab-5 Dm
Rab-5B (Ypt52p) Sc
Rab-6B (Ypt10p) Sc
Rab-20 Hs *
Rab-5C Dm
VI. Retrograde golgi
Rab-6A Hs **
Rab-6C Hs
Rab-6B Hs
Rab-6 Dm
Rab-6 (Ypt6p) Sc **
18678
18022
10235
17436
VII. Late endocytosis
Rab-7 Dm
Rab-7 Hs **
Rab-7 (Ypt7p) Sc **
Rab-9A Hs **
Rab-9B Hs
Rab-9 Dm
Rab-4B Dm
Rab23/28 clade
Rab-23Dm
Rab-23 Hs
Rab-28 Hs
18012
19253
Tetrahymena Clade III
10943
12036
26464
17445
mitochondria & lipid droplet associated clade
Rab-32 Dm
Rab-32 Hs **
Rab-38 Hs
1184
Rab-15 Hs
1808
Rab-18 Dm
Rab-18 Hs **
Rab34/36 clade
7253
Rab-34 Hs
Rab-36 Hs
Rab-36 Dm
Tetrahymena Clade II
23853
19980
19979
7948
13802
13805
13801
13739
13740
13741
II. Endocytosis/recycling
Rab-11A Hs **
Rab-11B Hs
Rab-11 Dm **
Rab-25 Hs **
Rab-11A (Ypt31p) Sc **
Rab-11B (Ypt32p) Sc
20971
25160
5211
22521
24892
3592
20594
20020
26961
11517
5815
7885
Rab-2 Dm
Rab-2A Hs **
Rab-2B Hs
18681
Rab-4A Hs *
Rab-4B Hs
Rab-4C Dm
Rab-14 Dm
Rab-14 Hs **
2414
1053
21874
20011
Rab-39 Dm
Rab-39 Hs *
III. Regulated exocytosis
Rab-3A Hs **
Rab-3C Hs
Rab-3B Hs
Rab-3 Dm
Rab-3D Hs
Rab-27A Hs **
Rab-27B Hs
Rab-27 Dm
20702
7255
Rab-12 (Ypt11p) Sc
secretory granule associated clade
Rab-26 Hs *
Rab-37 Hs *
Rab-26 Dm
Rab-30 Dm
Rab-30 Hs *
Rab-1B Dm
I. Exocytosis
Rab-1A Hs **
Rab-1B Hs
Rab-1A Dm **
Rab-1 (Ypt1p) Sc **
20939
7104
3985
26486
Rab-35 Dm
Rab-35 Hs
VIII. Golgi related
Rab-8A Hs *
Rab-8B Hs
Rab-8A Dm
Rab-10 Dm
Rab-10 Hs *
Rab-13 Hs
Rab-8B Dm
Rab-8 (Sec4p) Sc **
9358
4816
12482
Rab40 clade
Rab-40A Hs
Rab-40B Hs
Rab-40C Hs
Rab-40D Hs
