# Supplementary material for: Macronuclear Genome Sequence of the Ciliate Tetrahymena thermophila, a Model Eukaryote
Source: PLoS Biol. 2006 Aug 29;4(9):e286. doi: 10.1371/journal.pbio.0040286 (PMC1557398; doi:10.1371/journal.pbio.0040286)
Supplement: Supplementary file 8 [file pbio.0040286.st001.doc]

Table S1: Genomic DNA libraries.

| **Name** | **Size range of DNA selected for cloning (kb)** | **% Good Sequences**  **in Test** | **% No Insert**  **in Test** | **Shotgun sequences used in Final Assembly** |
| --- | --- | --- | --- | --- |
| **TTAAA** | 1.5-2.0 | 95 | 0 | 4,039 |
| **TUAAA** | 2.0-3.0 | 90 | 0 | 209,373 |
| **TXAAA** | 3.0-4.0 | 88 | 1 | 492,791 |
| **TYAAA** | 4.0-6.0 | 85 | 1 | 485,289 |
| **TQAAA** | 6.0-10.0 | 45 | 27 | 88 |
