# Supplementary material for: Macronuclear Genome Sequence of the Ciliate Tetrahymena thermophila, a Model Eukaryote
Source: PLoS Biol. 2006 Aug 29;4(9):e286. doi: 10.1371/journal.pbio.0040286 (PMC1557398; doi:10.1371/journal.pbio.0040286)
Supplement: Supplementary file 9 [file pbio.0040286.st002.doc]

**Table S2: Statistics on chromosome assemblies and satellite repeats**

**A. Comparison of unpartitioned and partitioned assemblies**

|  | **Assembly 1** | **Assembly 2 (Partitioned Assembly)** | | |
| --- | --- | --- | --- | --- |
|  | **All DNA** | **rDNA** | **Mitochondrion** | **Major chromosomes** |
| **Scaffolds** | 2,988 | 2 | 1 | 1,971 |
| **Contigs** | 4,223 | 2 | 1 | 2,955 |
| **Bases in scaffolds** | 106,196,540 | 12,166 | 45,538 | 103,927,049 |
| **Span of scaffolds (bp)** | 106,780,224 | 12,166 | 45,538 | 104,194,423 |
| **Largest contig (bp)** | 715,652 | 9,027 | 45,538 | 715,652 |
| **Largest scaffold (bp)** | 2,217,035 | 12,166 | 45,538 | 2,214,258 |
| **Coverage** | 9.01 x | 635x | 17.85x | 9.08x |

**B. Additional Major Chromosomes Statistics**

**Scaffolds**

Min: Max: Mean contigs per scaffold = 1: 23: 1.50

Mean scaffold length: span (bp) = 52,728: 52,864

Min scaffold length: span (bp) = 1,000: 1,000

N50 scaffold length = 495,069 bp

IntraScaffold gaps = 984

Mean sequence gap length = 272 bp

Five largest scaffolds:

| **Number of contigs** | **Length (bp)** | **Span (bp)** | **Average contig (bp)** | **Average gap (bp)** |
| --- | --- | --- | --- | --- |
| 20 | 2,214,258 | 2,218,438 | 110,713 | 220.0 |
| 14 | 1,806,240 | 1,807,785 | 129,017 | 118.9 |
| 6 | 1,556,938 | 1,557,319 | 259,490 | 76.2 |
| 15 | 1,387,964 | 1,390,127 | 92,531 | 154.5 |
| 7 | 1,231,306 | 1,231,723 | 175,901 | 69.5 |

**Contigs**

Min: Mean contig length (bp) = 251: 35,170

N50 contig length = 182,859 bp

Contigs >10 kb = 980 (33.2% of total)

Five largest contigs:

| **Number of reads** | **Length (bp)** |
| --- | --- |
| 8218 | 715,652 |
| 8401 | 682,009 |
| 7359 | 628,690 |
| 7423 | 626,575 |
| 7414 | 620,383 |

**Degenerate Contigs**

Total degenerate contigs = 1,699

Total degenerate contig length = 1,524,678 bp

Min: Max: Mean degenerate contig length (bp) = 124: 6,625: 897.4

Percentage of all bases in degenerate contigs = 1.47

**Reads**

Reads in contigs >10 kb = 1,118,135

Reads in contigs <10 kb = 19,624

Degenerate contig reads = 5,920

Reads in surrogates = 8,268

Singleton reads = 29,034

Average edited length = 815 bp

**C. Micro- and mini-satellite repeats***

Total = 9803

Total length = 2.5 Mb (2.4% of genome)

Repeat frequency = 94/Mb

Length mean: mode (bp) = 256: 30-40

Period mean: mode (bp) = 57: 4

Average GC content = 24.9%

*(Benson 1999)
