# Supplementary material for: Macronuclear Genome Sequence of the Ciliate Tetrahymena thermophila, a Model Eukaryote
Source: PLoS Biol. 2006 Aug 29;4(9):e286. doi: 10.1371/journal.pbio.0040286 (PMC1557398; doi:10.1371/journal.pbio.0040286)
Supplement: Supplementary file 10 [file pbio.0040286.st003.doc]

Table S3: Scaffolds capped by telomeres. 5’ and 3’ ends correspond to the orientation of the scaffolds in the November 2003 assembly <http://www.tigr.org/tdb/e2k1/ttg/>.

| **Scaffold ID** | **Scaffold Size (bp)** | **Number of telomere-capped ends** | **Number of 5' Tel-read Links** | **Number of 3' Tel-read Links** |
| --- | --- | --- | --- | --- |
| 8253807 | 16558 | 1 |  | 14 |
| 8253811 | 495369 | 2 | 11 | 4 |
| 8253815 | 514547 | 2 | 6 | 11 |
| 8253817 | 192876 | 2 | 6 | 4 |
| 8253823 | 331797 | 2 | 9 | 9 |
| 8253829 | 3705 | 1 | 2 |  |
| 8253833 | 1419 | 1 | 1 |  |
| 8253834 | 26995 | 1 | 7 |  |
| 8253839 | 5721 | 1 |  | 10 |
| 8253843 | 128647 | 1 | 8 |  |
| 8253848 | 17833 | 1 |  | 9 |
| 8253850 | 425044 | 1 | 7 |  |
| 8253864 | 39867 | 1 | 7 |  |
| 8253870 | 4992 | 1 | 5 |  |
| 8253878 | 2382 | 1 |  | 1 |
| 8253880 | 122320 | 2 | 5 | 7 |
| 8253886 | 149881 | 2 | 8 | 10 |
| 8253887 | 364147 | 2 | 15 | 8 |
| 8253891 | 330571 | 2 | 12 | 17 |
| 8253894 | 215179 | 2 | 15 | 7 |
| 8253902 | 45225 | 1 |  | 12 |
| 8253904 | 274829 | 1 |  | 13 |
| 8253906 | 8877 | 1 |  | 9 |
| 8253908 | 195107 | 1 |  | 4 |
| 8253914 | 67916 | 1 | 13 |  |
| 8253915 | 329665 | 2 | 8 | 10 |
| 8253930 | 173734 | 1 | 8 |  |
| 8253957 | 118128 | 1 | 13 |  |
| 8253964 | 99055 | 2 | 10 | 8 |
| 8253973 | 1918 | 1 |  | 1 |
| 8253974 | 55028 | 2 | 7 | 8 |
| 8253984 | 204589 | 2 | 12 | 9 |
| 8253994 | 48522 | 1 | 3 |  |
| 8254002 | 88194 | 2 | 15 | 12 |
| 8254007 | 84068 | 1 | 15 |  |
| 8254010 | 681777 | 2 | 14 | 8 |
| 8254021 | 21071 | 1 |  | 5 |
| 8254028 | 344051 | 2 | 17 | 9 |
| 8254033 | 32639 | 1 | 9 |  |
| 8254034 | 211760 | 1 |  | 6 |
| 8254043 | 250422 | 2 | 10 | 16 |
| 8254049 | 86667 | 1 | 11 |  |
| 8254051 | 162284 | 2 | 15 | 10 |
| 8254058 | 28992 | 1 | 10 |  |
| 8254062 | 51224 | 2 | 10 | 12 |
| 8254072 | 37300 | 2 | 11 | 12 |
| 8254075 | 56809 | 2 | 17 | 6 |
| 8254092 | 100177 | 1 |  | 10 |
| 8254095 | 95299 | 1 | 13 |  |
| 8254106 | 107234 | 2 | 12 | 13 |
| 8254122 | 57503 | 2 | 6 | 12 |
| 8254159 | 34715 | 1 |  | 8 |
| 8254163 | 1338 | 1 |  | 1 |
| 8254167 | 149771 | 2 | 11 | 12 |
| 8254181 | 184770 | 2 | 5 | 5 |
| 8254184 | 1879 | 1 |  | 1 |
| 8254185 | 114016 | 2 | 7 | 4 |
| 8254187 | 311280 | 2 | 11 | 5 |
| 8254194 | 134315 | 2 | 13 | 11 |
| 8254199 | 125916 | 2 | 8 | 4 |
| 8254211 | 1865 | 1 | 3 |  |
| 8254222 | 20223 | 1 |  | 11 |
| 8254241 | 107418 | 2 | 7 | 10 |
| 8254260 | 41092 | 2 | 6 | 11 |
| 8254265 | 13464 | 1 |  | 12 |
| 8254284 | 255706 | 2 | 9 | 5 |
| 8254305 | 15432 | 1 |  | 8 |
| 8254332 | 50910 | 1 | 8 |  |
| 8254359 | 221702 | 2 | 7 | 8 |
| 8254360 | 79643 | 2 | 10 | 16 |
| 8254361 | 73558 | 1 | 11 |  |
| 8254362 | 275164 | 2 | 9 | 12 |
| 8254365 | 330390 | 2 | 7 | 5 |
| 8254367 | 262110 | 2 | 12 | 5 |
| 8254369 | 157825 | 1 |  | 8 |
| 8254370 | 255838 | 2 | 11 | 10 |
| 8254371 | 530472 | 2 | 14 | 9 |
| 8254373 | 875719 | 2 | 12 | 8 |
| 8254375 | 46133 | 1 |  | 5 |
| 8254376 | 9227 | 1 | 11 |  |
| 8254377 | 298147 | 2 | 11 | 12 |
| 8254378 | 385159 | 2 | 12 | 13 |
| 8254379 | 757155 | 2 | 11 | 2 |
| 8254381 | 55553 | 1 | 11 |  |
| 8254382 | 167276 | 2 | 11 | 9 |
| 8254385 | 477791 | 1 |  | 10 |
| 8254391 | 308699 | 1 |  | 8 |
| 8254392 | 82778 | 2 | 11 | 5 |
| 8254393 | 52933 | 2 | 4 | 9 |
| 8254394 | 11350 | 1 | 8 |  |
| 8254395 | 541259 | 2 | 12 | 8 |
| 8254396 | 98811 | 2 | 19 | 12 |
| 8254397 | 230559 | 2 | 14 | 6 |
| 8254401 | 113371 | 2 | 14 | 9 |
| 8254402 | 355428 | 2 | 9 | 8 |
| 8254403 | 526382 | 2 | 7 | 7 |
| 8254406 | 840097 | 1 | 12 |  |
| 8254410 | 135975 | 2 | 8 | 13 |
| 8254412 | 117973 | 1 | 3 |  |
| 8254415 | 71046 | 1 | 9 |  |
| 8254417 | 272172 | 1 | 7 |  |
| 8254420 | 312865 | 2 | 5 | 9 |
| 8254422 | 319250 | 2 | 9 | 10 |
| 8254423 | 91433 | 1 | 12 |  |
| 8254428 | 470161 | 2 | 10 | 19 |
| 8254429 | 128373 | 2 | 8 | 10 |
| 8254431 | 715652 | 1 | 10 |  |
| 8254433 | 111488 | 2 | 10 | 13 |
| 8254435 | 356818 | 1 | 8 |  |
| 8254436 | 177920 | 1 | 16 |  |
| 8254437 | 547543 | 2 | 7 | 13 |
| 8254440 | 229727 | 2 | 5 | 8 |
| 8254444 | 399810 | 2 | 11 | 11 |
| 8254446 | 515732 | 2 | 3 | 5 |
| 8254447 | 86709 | 1 | 7 |  |
| 8254448 | 189492 | 2 | 12 | 10 |
| 8254449 | 251867 | 2 | 5 | 12 |
| 8254454 | 99508 | 2 | 2 | 16 |
| 8254456 | 75921 | 2 | 6 | 10 |
| 8254457 | 136848 | 2 | 11 | 10 |
| 8254459 | 486561 | 2 | 7 | 10 |
| 8254460 | 638469 | 2 | 12 | 12 |
| 8254464 | 405752 | 2 | 8 | 7 |
| 8254468 | 126255 | 2 | 9 | 13 |
| 8254469 | 111836 | 2 | 14 | 10 |
| 8254470 | 379103 | 1 |  | 11 |
| 8254473 | 40141 | 1 |  | 5 |
| 8254475 | 588109 | 2 | 15 | 6 |
| 8254476 | 274746 | 2 | 5 | 2 |
| 8254479 | 384242 | 1 |  | 14 |
| 8254481 | 36977 | 1 |  | 12 |
| 8254482 | 19320 | 1 |  | 9 |
| 8254483 | 230791 | 2 | 7 | 12 |
| 8254484 | 94841 | 1 | 5 |  |
| 8254486 | 430721 | 2 | 7 | 13 |
| 8254487 | 513792 | 2 | 14 | 6 |
| 8254491 | 250031 | 2 | 12 | 8 |
| 8254495 | 427072 | 2 | 10 | 11 |
| 8254496 | 209627 | 1 | 8 |  |
| 8254497 | 69572 | 2 | 6 | 9 |
| 8254498 | 48661 | 2 | 9 | 10 |
| 8254503 | 182014 | 2 | 12 | 12 |
| 8254504 | 98328 | 2 | 13 | 11 |
| 8254505 | 129549 | 2 | 5 | 4 |
| 8254512 | 64653 | 1 |  | 8 |
| 8254515 | 276251 | 1 | 5 |  |
| 8254517 | 44754 | 2 |  | 15 |
| 8254525 | 56404 | 1 | 8 |  |
| 8254527 | 520468 | 2 | 9 | 13 |
| 8254528 | 371972 | 1 | 10 |  |
| 8254530 | 87247 | 1 |  | 2 |
| 8254544 | 102732 | 1 | 3 |  |
| 8254545 | 1045189 | 2 | 8 | 4 |
| 8254548 | 714158 | 2 | 10 | 10 |
| 8254549 | 250927 | 1 | 7 |  |
| 8254550 | 192263 | 1 |  | 9 |
| 8254551 | 718613 | 2 | 14 | 15 |
| 8254552 | 244997 | 1 |  | 2 |
| 8254555 | 596115 | 2 | 12 | 7 |
| 8254563 | 396064 | 2 | 7 | 9 |
| 8254564 | 889818 | 2 | 15 | 6 |
| 8254565 | 526763 | 2 | 7 | 16 |
| 8254574 | 72513 | 2 | 10 | 14 |
| 8254577 | 348347 | 2 | 3 | 1 |
| 8254579 | 346661 | 1 |  | 9 |
| 8254580 | 399732 | 2 | 13 | 7 |
| 8254582 | 1075194 | 2 | 14 | 6 |
| 8254584 | 383217 | 1 |  | 12 |
| 8254588 | 212214 | 2 | 11 | 10 |
| 8254589 | 153188 | 1 |  | 7 |
| 8254590 | 838362 | 1 |  | 7 |
| 8254592 | 404784 | 1 | 16 |  |
| 8254593 | 533928 | 1 | 9 |  |
| 8254594 | 762169 | 2 | 7 | 7 |
| 8254595 | 152962 | 2 | 5 | 6 |
| 8254597 | 644724 | 2 | 6 | 9 |
| 8254599 | 80448 | 1 |  | 4 |
| 8254600 | 816542 | 2 | 9 | 15 |
| 8254607 | 557789 | 2 | 9 | 12 |
| 8254609 | 555380 | 2 | 13 | 8 |
| 8254610 | 665075 | 2 | 8 | 10 |
| 8254611 | 521720 | 2 | 12 | 11 |
| 8254617 | 935000 | 1 |  | 14 |
| 8254622 | 72856 | 2 | 14 | 8 |
| 8254630 | 260511 | 1 | 9 |  |
| 8254631 | 114510 | 1 |  | 6 |
| 8254632 | 78080 | 2 | 2 | 11 |
| 8254634 | 161471 | 1 | Cbs |  |
| 8254637 | 226025 | 1 | 8 |  |
| 8254638 | 979488 | 2 | 5 | 16 |
| 8254644 | 356951 | 2 | 7 | 10 |
| 8254645 | 1076301 | 2 | 6 | 14 |
| 8254648 | 202485 | 1 |  | 9 |
| 8254649 | 819627 | 2 | 7 | 10 |
| 8254650 | 1231906 | 1 |  | 10 |
| 8254652 | 611172 | 1 |  | 12 |
| 8254654 | 510086 | 2 | 6 | 4 |
| 8254658 | 337613 | 1 |  | 3 |
| 8254659 | 1557438 | 1 | 9 |  |
| 8254662 | 56407 | 1 | 1 |  |
| 8254664 | 501920 | 2 | 8 | 6 |
| 8254666 | 596578 | 2 | 14 | 8 |
| 8254667 | 356833 | 1 |  | 11 |
| 8254670 | 254291 | 1 |  | 6 |
| 8254673 | 199572 | 1 |  | 7 |
| 8254686 | 541833 | 1 |  | 8 |
| 8254688 | 757561 | 2 | 9 | 5 |
| 8254691 | 686164 | 1 |  | 8 |
| 8254693 | 27987 | 1 |  | 4 |
| 8254697 | 1227316 | 1 |  | 10 |
| 8254707 | 196396 | 1 |  | 6 |
| 8254711 | 128752 | 1 | 10 |  |
| 8254712 | 240713 | 2 | 7 | 9 |
| 8254719 | 598405 | 1 |  | 14 |
| 8254731 | 397087 | 1 | 9 |  |
| 8254737 | 250189 | 1 | 8 |  |
| 8254747 | 1051704 | 2 | 18 | 16 |
| 8254748 | 306323 | 2 | 13 | 4 |
| 8254749 | 103417 | 2 | 14 | 6 |
| 8254752 | 1171415 | 1 |  | 7 |
| 8254756 | 635630 | 1 |  | 7 |
| 8254757 | 149000 | 2 | 8 | 9 |
| 8254761 | 125722 | 1 | 12 |  |
| 8254763 | 427219 | 2 | 5 | 11 |
| 8254767 | 29593 | 1 | 2 |  |
| 8254776 | 1004159 | 1 |  | 9 |
| 8254777 | 613210 | 2 | 11 | 9 |
| 8254778 | 133317 | 1 | 10 |  |
| 8254782 | 422999 | 1 |  | 8 |
| 8254786 | 439354 | 1 | 10 |  |
| 8254788 | 821283 | 1 |  | 7 |
| 8254794 | 62198 | 1 |  | 5 |
| 8254798 | 983259 | 1 |  | 13 |
| 8254803 | 2216158 | 1 | 9 |  |
| 8254807 | 464229 | 2 | 23 | 11 |
| 8254811 | 1389364 | 2 | 7 | 4 |
| 8254814 | 591214 | 1 |  | 22 |
| 8254815 | 909308 | 1 |  | 2 |
| 8254823 | 461832 | 2 | 10 | 11 |
| 8255062 | 1866 | 1 | 4 |  |
| 8255237 | 1277 | 1 | 1 |  |
| 8255326 | 1387 | 1 |  | 1 |
| 8255327 | 1006 | 1 |  | 1 |
| 8255497 | 1109 | 1 |  | 2 |
| Average |  |  | 9.3 | 8.8 |

Cbs: The scaffold end was linked to a telomere indirectly, through a match to Cbs-adjacent sequence.
