# Supplementary material for: Macronuclear Genome Sequence of the Ciliate Tetrahymena thermophila, a Model Eukaryote
Source: PLoS Biol. 2006 Aug 29;4(9):e286. doi: 10.1371/journal.pbio.0040286 (PMC1557398; doi:10.1371/journal.pbio.0040286)
Supplement: Supplementary file 11 [file pbio.0040286.st004.doc]

Table S4. Matches of RAPD DNA polymorphisms to scaffolds

| **Scaffold ID** | **Locus** | **MIC linkage group** | **MAC coassortment group** | **Scaffold Size (Mb)** | **Chromosome Size (Mb)** | **# Telo-meres** |
| --- | --- | --- | --- | --- | --- | --- |
| 8253890 | 1AT03R | 1L-mat | 1L-AT03 | 0.03 | ND | 0 |
| 8254475 | 1SP09 | 1L-mat | 1L-BD06(+) | 0.59 | 0.61 | 2 |
| 8254590 | 1CH01 | 1L-mat | 1L-CH01 | 0.84 | ND | 1 |
| 8254428 | 1JB03 | 1L-mat | 1L-JB03(+) | 0.47 | 0.50 | 2 |
| 8254497 | 1JB10R | 1L-mat | 1L-JB10R | 0.07 | 0.07 | 2 |
| 8254417 | 1JO07 | 1L-mat | 1L-JO07 | 0.27 | 0.27 | 1 |
| 8254576 | 1GM09 | 1L-mat | 1L-JO13R | 0.19 | 1.50 | 0 |
| 8254551 | 1JP17 | 1L-mat | 1L-JP17 | 0.72 | 0.70 | 2 |
| 8254435 | 1KN03 | 1L-mat | 1L-KN03 | 0.36 | 1.00 | 1 |
| 8254606 | 1PM08 | 1L-mat | **1L-PM08** | 0.11 | 1.20 | 0 |
| 8254677 | 1JO09 | 1L-mat | **1L-PM08** | 0.09 | 1.20 | 0 |
| 8254720 | 1EO03R | 1L-mat | **1L-PM08** | 0.05 | 1.20 | 0 |
| 8254611 | 1RT01 | 1L-mat | 1L-RT01 | 0.52 | 0.55 | 2 |
| 8254580 | 1XS24 | 1L-mat | 1L-XS24 | 0.40 | 0.44 | 2 |
| 8254688 | 1BD11 | 1L-mat | 1L-XS36 | 0.76 | 0.79 | 2 |
| 8253817 | 1AT01 | 1R-GAL1 | 1R-AT01(+) | 0.19 | 0.22 | 2 |
| 8254047 | 1AT01R | 1R-GAL1 | 1R-AT01R | 0.25 | 1.80 | 0 |
| 8254502 | 1BD03 | 1R-BD03 | 1R-BD03 | 0.11 | 0.79 | 0 |
| **8254572** | 1SN14 | 1R-CHX1 | **1R-CHX1** | 0.20 | 0.95 | 0 |
| **8254572** | 1JB14 | 1R-CHX1 | **1R-CHX1** | 0.20 | ND | 0 |
| 8255548 | 1JO91R | 1R-CHX1 | **1R-CHX1** | 0.00 | 0.95 | 0 |
| 8254371 | 1LC04 | 1R-CHX1 | 1R-EstA | 0.53 | 0.55 | 2 |
| **8254747** | 1YD02 | 1R-GAL1 | **1R-GAL1** | 1.05 | 1.10 | 2 |
| **8254747** | 1JO65 | 1R-GAL1 | **1R-GAL1** | 1.05 | 1.12 | 2 |
| 8254664 | 1LS20R | 1R-GAL1 | 1R-GRL7 | 0.50 | 0.53 | 2 |
| 8254637 | 1JB12 | 1R-CHX1 | 1R-JB12 | 0.23 | ND | 1 |
| 8253984 | 1JO03 | 1R-GAL1 | 1R-JO03 | 0.20 | 0.23 | 2 |
| 8254512 | 1LC09R | 1R-CHX1 | 1R-LC09R | 0.06 | ND | 1 |
| 8254469 | 1LC10 | 1R-GAL1 | 1R-LC10 | 0.11 | 0.10 | 2 |
| 8254525 | 1LS18R | 1R-GAL1 | 1R-LS18R(+) | 0.06 | 0.50 | 1 |
| **8254487** | 1MJ16R | 1R-GAL1 | **1R-MJ16R** | 0.51 | 0.55 | 2 |
| **8254487** | 1MJ13R | 1R-GAL1 | **1R-MJ16R*** | 0.51 | 0.55 | 2 |
| 8254604 | 1JO14 | 1R-CHX1 | 1R-ML03 | 0.02 | ND | 0 |
| 8254737 | 1SP09a | 1R-GAL1 | 1R-SP09a | 0.25 | 0.90 | 1 |
| 8254636 | 1LS17 | 1R-GAL1 | 1R-SP18R | 0.52 | 1.90 | 0 |
| 8254028 | 1YD11 | 1R-GAL1 | 1R-YD11 | 0.34 | 0.37 | 2 |
| 8254406 | 1LR03 | 2L-SP13 | 2L-SP13 | 0.84 | 1.60 | 1 |
| **8254803** | 1KP02R | 2R-MPR1 | **2R-MPR1** | 2.22 | 1.90 | 1 |
| **8254803** | 1AT04 | 2R-MPR1 | **2R-MPR1*** | 2.22 | 1.90 | 1 |
| **8254470** | 1BR07b | 2R-JO66 | **2R-XS27** | 0.38 | 1.30 | 1 |
| **8254470** | 1XS27 | 2R-JO66 | **2R-XS27** | 0.38 | ND | 1 |
| 8253823 | 1XS41 | 2R-XS41 | 2R-XS41 | 0.33 | 0.38 | 2 |
| 8254436 | 1BD22 | 3L-XS43 | 3L-BD22 | 0.18 |  | 1 |
| 8255314 | 1JB26 | 3L-XS43 | 3L-JB26 | 0.00 | ND | 0 |
| **8254819** | 1JB36 | 3L-JB36 | **3L-JB36** | 1.81 | 1.90 | 0 |
| **8254819** | 1JB35 | 3L-JB36 | **3L-JB36*** | 1.81 | 1.90 | 0 |
| 8254610 | 1JP03 | 3L-FBR1 | 3L-JP03 | 0.67 | 0.67 | 2 |
| 8254544 | 1MJ18R | 3L-FBR1 | 3L-JP23 | 0.10 | 0.28 | 1 |
| 8254491 | 1XS43 | 3L-XS43 | 3L-XS43(+) | 0.25 | ND | 2 |
| 8254638 | 1JP24 | 3R-JO06 | 3R-JB16 | 0.98 | ND | 2 |
| 8254790 | 1EM08 | 3R-LC08 | 3R-JB21 | 0.02 | 1.60 | 0 |
| 8254652 | 1LC09 | 3R-JO06 | 3R-LC09(+) | 0.61 | ND | 1 |
| 8254617 | 1LC21R | 3R-JO06 | 3R-LC21R | 0.94 | 1.70 | 1 |
| **8254528** | 1XS08 | 3R-JO06 | **3R-XS60R** | 0.37 | 1.60 | 1 |
| **8254528** | 1JO35R | 3R-JO06 | **3R-XS60R*** | 0.37 | 1.64 | 1 |
| 8254670 | 1XS61R | 3R-AcpA | 3R-XS61R | 0.25 | 1.64 | 1 |
| 8254607 | 1JO63 | 4L-QD02R | 4L-AT18 | 0.56 | 0.60 | 2 |
| 8254646 | 1JO02 | 4L-JB06 | 4L-JO02(+) | 0.23 | 1.59 | 0 |
| 8254484 | 1JP22 | 4L-BR19 | 4L-JP22 | 0.09 | 0.45 | 1 |
| 8254676 | 1AT11 | 4L-BR19 | 4L-LK04 | 0.07 | 1.70 | 0 |
| 8254373 | 1JO82R | 4L-QD02R | 4L-ML04R | 0.88 | 1.00 | 2 |
| 8254515 | 1MJ04 | 4L-AS01 | 4L-YD20 | 0.28 | 0.48 | 1 |
| 8254181 | 1JO06a | 4R-LC01 | 4R-JO62 | 0.18 | ND | 2 |
| 8254665 | 1LC20 | 4L-BR19 | 4R-LC20 | 0.88 | ND | 0 |
| 8254395 | 1LC26 | 4R-LC01 | 4R-LC26 | 0.54 | 0.67 | 2 |
| **8254010** | 1LS04 | 4R-LC01 | **4R-LS04** | 0.68 | ND | 2 |
| **8254010** | 1AT04R | 4R-LC01 | **4R-LS04*** | 0.68 | ND | 2 |
| 8254194 | 1MJ10R | 4R-LC01 | 4R-MJ10R | 0.13 | ND | 2 |
| 8253904 | 1XS16 | 4R-LC01 | **4R-XS16** | 0.27 | 1.12 | 1 |
| **8254587** | 1BR01 | 4R-LC01 | **4R-XS16** | 0.61 | 1.12 | 0 |
| **8254587** | 1JO34R | 4R-LC01 | **4R-XS16** | 0.61 | 1.20 | 0 |
| **8254587** | 1XS74R | 4R-LC01 | **4R-XS16? ND** | 0.61 | ND | 0 |
| 8254577 | 1BD34R | 5X-LC16a | 5X-BD34R | 0.35 | ND | 2 |
| **8254650** | 1JP01R | 5X-JB04 | **5X-BR02** | 1.23 | 1.64 | 1 |
| **8254650** | 1YD14 | 5X-JB04 | **5X-BR02*** | 1.23 | 1.40 | 1 |
| 8254691 | 1JB29 | 5X-JP02 | 5X-JB29 | 0.69 | 0.86 | 1 |
| 8254823 | 1JO67 | 5X-JB04 | 5X-JO67 | 0.46 | 0.49 | 2 |
| 8254597 | 1JP02 | 5X-JP02 | 5X-JP04 | 0.64 | 0.68 | 2 |
| 8254718 | 1LC20R | 5X-JB04 | 5X-JP08d | 0.24 | ND | 0 |
| 8254152 | 1BR14 | 5X-JB04 | 5X-JP12 | 0.02 | 0.65 | 0 |
| 8254799 | 1LC16 | 5X-JB04 | 5X-LC16 | 0.23 | 1.90 | 0 |
| 8254800 | 1LC19 | 5X-JB04 | 5X-LR01R | 0.09 | ND | 0 |
| 8254634 | 1LS28R | 5X-JP02 | 5X-LS28R | 0.16 | 0.90 | 0 |
| 8254686 | 1AS02R | 5X-JB04 | 5X-RK04a | 0.54 | 0.57 | 1 |
| 8254649 | 1RK06R | 5X-JB04 | 5X-RK06R | 0.82 | 0.82 | 2 |
| 8254645 | 1SP16R | 5X-LC16a | 5X-SP16R | 1.08 | ND | 2 |
| 8254811 | 1XS01R | 5X-JP02 | 5X-XS01R(+) | 1.39 | ND | 2 |
| **8254377** | 1YD10 | 5X-LC16a | **5X-YD10** | 0.30 | 0.33 | 2 |
| **8254377** | 1YD16 | 5X-LC16a | **5X-YD10** | 0.30 | 0.35 | 2 |
| 8254668 | 1EM01R | 1R-GAL1 | ND | 0.02 | 0.38 | 0 |

* Coassortment group assignment based on scaffold co-location.

(+) and *: exceptionally strong PCR band of MIC origin hid the MAC assortment pattern. ND: not determined.

Note that:

- DNA polymorphisms mapping to the same scaffold (bold) belong to the same coassortment group (i.e. MAC chromosome);

- DNA polymorphisms mapping to the same coassortment group (bold) can map to different scaffolds. If so, those scaffolds are incomplete (less than 2 telomere caps) and the sum of the length of the scaffolds is less than the MAC chromosome size.

- The relationship between scaffold and MAC chromosome size is plotted in Figure 2.
