# Supplementary material for: Macronuclear Genome Sequence of the Ciliate Tetrahymena thermophila, a Model Eukaryote
Source: PLoS Biol. 2006 Aug 29;4(9):e286. doi: 10.1371/journal.pbio.0040286 (PMC1557398; doi:10.1371/journal.pbio.0040286)
Supplement: Supplementary file 12 [file pbio.0040286.st005.doc]

Table S5: *T. thermophila* ESTs, including available GenBank entries.

|  | **Number of ESTs** | **Average length** | **Average %GC** | **Number align to genome** | **Numberclusters** | **Number sub-clusters** |
| --- | --- | --- | --- | --- | --- | --- |
| **Vegetative** | 20,632 | 665 | 40.86 | 13,974 | 1,569 | 1,472 |
| **Conjugative** | 20,714 | 744 | 30.87 | 18,378 | 8,230 | 7,932 |
| **All ESTs** | 41,346 | 688 | 33.5 | 32,352 | 9,122 | 8,708 |
