# Supplementary material for: Macronuclear Genome Sequence of the Ciliate Tetrahymena thermophila, a Model Eukaryote
Source: PLoS Biol. 2006 Aug 29;4(9):e286. doi: 10.1371/journal.pbio.0040286 (PMC1557398; doi:10.1371/journal.pbio.0040286)
Supplement: Supplementary file 13 [file pbio.0040286.st006.doc]

Table S6. Non-coding RNAs

A: DNA sequences of the 174 genes predicted by BLASTN closest match to be transcribed into 5S rRNA. Unique sequences are sorted first by copy number, then by proximity of length to 120 nt. Variable sequences found at the ends and thought to form intramolecular hydrogen bonds are indicated by bold, underlined characters in the 120 nt examples.

| **Copy Number** | **Length (nt)** | **Sequence** |
| --- | --- | --- |
| 91 | 120 | G**T**T**G**TCGGCCATACTAAGGTGAAAACACCGGATCCCATTCG  AACTCCGAAGTTAAGCGCCTTAAGGCTGGGTTAGTACTAAG  GTGGGGGACCGCTTGGGAAGTCCCAGTGTCGA**C**A**A**CCT |
| 50 | 120 | G**T**T**A**TCGGCCATACTAAGGTGAAAACACCGGATCCCATTCG  AACTCCGAAGTTAAGCGCCTTAAGGCTGGGTTAGTACTAAG  GTGGGGGACCGCTTGGGAAGTCCCAGTGTCGA**T**A**A**CCT |
| 11 | 120 | G**C**T**G**TCGGCCATACTAAGGTGAAAACACCGGATCCCATTCG  AACTCCGAAGTTAAGCGCCTTAAGGCTGGGTTAGTACTAAG  GTGGGGGACCGCTTGGGAAGTCCCAGTGTCGA**C**A**G**CCT |
| 3 | 120 | G**T**T**G**TCGGCCATACTAAGGTGAAAACACCGGATCCCATTCG  AACTCCGAAGTTAAGCGCCTTAAGGCTGGGTTAGTACTAAG  GTGGGGGACCGCTTGGGAAGTCCCAGTGTCGA**C**A**G**CCT |
| 1 | 120 | G**C**T**A**TCGGCCATACTAAGGTGAAAACACCGGATCCCATTCGAA  CTCCGAAGTTAAGCGCCTTAAGGCTGGGTTAGTACTAAGGTGG  GGGACCGCTTGGGAAGTCCCAGTGTCGA**T**A**G**CCT |
| 1 | 120 | G**C**T**G**TCGGCCATACTAAGGTGAAAACACCGGATCCCATTCGAA  CTCCGAAGTTAAGCGCCTTAAGGCTAGGTTAGTACTAAGGTGG  GGGACCGCTTGGGAAGTCCTAGTGTCGA**C**A**G**CCT |
| 1 | 120 | G**T**T**A**TCGGCCATACTAAGGTGAATACACCGGATCCCATTCGAA  CTCCGAAGTTAAGCGCCTTAAGGCTGGGTTAGTACTAAGGTGG  GGGACCGCTTGGGAAGTCCCAGTGTCGA**T**A**A**CCT |
| 1 | 120 | G**A**T**G**TCGGCCATACTAAGGTGAAAACACCGGATCCCATTCGAA  CTCCGAAGTTAAGCGCCTTAAGGCTGGGTTAGTACTAAGGTGGG  GGACCGCTTGGGAAGTCCCAGTGTCGA**C**A**T**CCT |
| 1 | 120 | G**T**T**G**TCGGCCATACTAAGGTGAAAACACCGGATCCCATTCGAAC  TCCGAAGTTAAGCGCCTTAAGGTTGGGTTAGTACTAAGGTGGGG  GACCGCTTGGGAAGTCCCAGTGTCGA**C**A**A**CCT |
| 1 | 120 | G**T**T**G**TCGGCCATACTAAGGTGAAAACACCGGATCCCATTCGAAC  TCCGAATTTAAGCGCCTTAAGGCTGGGTTAGTACTAAGGTGGGG  GACCGCTTGGGAAGTCCCAGTGTCGA**C**A**A**CCT |
| 1 | 120 | G**T**T**G**TCGGCCATACTAAGGTGAAAACACCGGATCCCATTTGAAC  TCCGAAGTTAAGCGCCTTAAGGCTGGGTTAGTACTAAGGTGGGG  GACCGCTTGGGAAGTCCCAGTGTCGA**C**A**A**CCT |
| 1 | 120 | G**T**T**G**TCTGCCATACTAAGGTGAAAACACCGGATCCCATTCGAAC  TCCGAAGTTAAGCGCCTTAAGGCTGGGTTAGTACTAAGGTGGGG  GACCGCTTGGGAAGTCCCAGTGTCGA**C**A**A**CCT |
| 1 | 120 | G**T**T**G**TCGGCCATACTAAGGTGAAAACACCGGATCTCATTCGAAC  TCCGAAGTTAAGCGCCTTAAGGCTGGGTTAGTACTAAGGTGGGG  GACCGCTTGGGAAGTCCCAGTGTCGA**C**A**A**CCT |
| 1 | 120 | G**T**T**G**TCGGCCATACTAAGGTGAAAACACCGGATCCCATTCGAAC  TCTGAAGTTAAGCGCCTTAAGGCTGGGTTAGTACTAAGGTGGGG  GACCGCTTGGGAAGTCCCAGTGTCGA**C**A**A**CCT |
| 1 | 119 | GCTGTCGGCCATACTAAGGTGAAAACACCGGATCCCATTCGAAC  TCCGAAGTTAAGCGCCTTAAGGCTGGGTTAGTACTAAGGTGGGG  GACCGCTTGGGAAGTCCCAGTGTCGACAGCC |
| 1 | 119 | TTATCGGCCATACTAAGGTGAAAACACCGGATCCCATTCGAAC  TCCGAAGTTAAGCGCCTTAAGGCTGGGTTAGTACTAAGGTGGG  GGACCGCTTGGGAAGTCCCAGTGTCGATAACCT |
| 1 | 119 | GTTGTCGGCCATACTAAGTTGAAAACACCGGATCCCATTCGAA  CTCCGAAGTTAAGCGCCTTAAGGCTGGGTTAGTACTAAGGTGG  GGGACCGCTTGGGAAGTCCCAGTGCCGACAACC |
| 1 | 121 | GTTGTTGGTCATACTAAGGTTTTCGAAAACACCAGATCCCAT  TCTAACTCCGAAGTTAAGCGCCTTAAGGCTGGGTTAGTACTA  AGGTGGGGGACCGCTTGGGAAGTCTTAGTGTTGATAA |
| 1 | 117 | GTTGTCGGCCATACTAAGGTGAAAACACCGGATCCCATTCGAA  CTCCGAAGTTAAGCGCCTTAAGGCTGGGTTAGTACTAAGGTGG  GGGACCGCTTGGGAAGTCCCAGTGTATACCC |
| 1 | 105 | TAAGGTGAAAACAACAGATCCCATTCGAACTCCGAAGTTAA  GCACCTTAAGACTGGGTTAGTACTTAGGTAGGGGACCGCTTG  GGAAGTCCCAATGCTGATAACC |
| 1 | 90 | GATCCAATTCGAACTCCGAAGTTAAGCGCCTTAAGGCTGGGTT  AGTACTAAGGTGGGGAACCGCTTGGGAAGTCCCAATGTCGATA  ACCT |
| 1 | 84 | GTTGTCTACCATAATAAGGTGAAAACCCCGGATTCCATTCGA  ACTCCGAAGTAAAGCGCCTTATGGCTGGGTTAGTACTAAGAT |
| 1 | 73 | GAAGTTAAGCGCCTTAAGGCTGGGTTAGTACTAAGGTGGGGGAC  CGCTTGGGAAGTCCCAGTGTCGACAACCT |

B.  The table summarizes tRNA gene copy number (number of genes with identical sequence), gene length (including both tRNA and intron length), type of charged amino acid, tRNA anticodon (indicated as genomic DNA sense strand sequence), tRNAscanSE score value, intron size (if applicable), and tRNA sequence (with intron or intron boundary sequences indicated with lower case lettering; N is ambiguous genome sequence data which was treated as unique sequence).  The final five entries are in the mtDNA genome.

| **Copy No.** | **Length** | **Type** | **Anticodon** | **Score** | **Intron size** | **Sequence** |
| --- | --- | --- | --- | --- | --- | --- |
| 32 | 73 | Ala | AGC | 49.52 |  | GGGGATCTAGCTCAAATGGTAGAGCGCTCGTTTAGCATACGAGAGGCACTGGGATCAATGCCCAGGTTCTCCA |
| 1 | 73 | Ala | CGC | 49.96 |  | GGGTAACTAGCTCAAATGGTAGAGCGTCTGGTTCGCATTCAGAAGGCACGGGGATCGATGCCCCGGTTATCCA |
| 1 | 73 | Ala | CGC | 46.98 |  | GGGTGTGTAGCTCATATGGTAGAGCGCTTGATTCGCATTCAGGAGGCAAGGGGATCGATGCCCCTCACATCTA |
| 1 | 73 | Ala | TGC | 55.57 |  | GGGTAACTAGCTCAAATGGTAGAGCGCCTGATTTGCATTCAGGAGGCACGGGGATCGATGCCCCGGTTATCCA |
| 1 | 73 | Ala | TGC | 46.89 |  | GGGTGTGTAGCTCACACGGCAGAGTACTTGACTTGCACTCAAGAGGCAGGGGGATCGAAGCCCTCCACATCCA |
| 9 | 73 | Ala | TGC | 54.1 |  | GGGTGTGTAGCTCACATGGTAGAGCGCTTGATTTGCATTCAAGAGGCAGGGGGATCGATGCCCCCCACATCCA |
| 6 | 72 | Arg | ACG | 75.3 |  | GGGGTGATGGCTCAATGGTAGAGCATCTGACTACGGATCAGAAGGTTGCAGGTTCGAGTCCTGCTCATCTCG |
| 2 | 72 | Arg | ACG | 77.17 |  | GGGGTGATGGCTCAATGGTAGAGCTTCTGACTACGGATCAGAAGGTTGCAGGTTCGAGTCCTGCTCACCTCG |
| 1 | 71 | Arg | CCG | 56.67 |  | AGCCCATTGGTCCAATGGTAGGGCATGAGATTCCGGCTCTCGAGGTCGGGGTTCGATTCCCCGGTGGGTTG |
| 2 | 72 | Arg | CCT | 71.83 |  | ATCCTTGTGGCGCAATGGTAGCGCAGTGGCCTCCTAAGCCAAAGGTTGTGAGTTCGAATCTCACCAAGGATG |
| 1 | 72 | Arg | TCG | 63.69 |  | GGCCTTGTGGCGCAATGGTAGCGCAGTGGATTTCGGCTCCAAAGGTTTAGGGTTCGAGTCCCTACGGGGTCG |
| 24 | 72 | Arg | TCT | 70.99 |  | AGCCGAGTGGCGCAATGGTAGCGCAGCAGACTTCTAATCTGAAGGTTGTGGGTTCGAGTCCCACCTTGGTTG |
| 1 | 72 | Arg | TCT | 46 |  | GTTACTTTGGTCTAACGGCAAAGACGGCGGACTTCTAATCTGTAAATCTGGGTTCGACTCCTAGAAGTAACA |
| 1 | 253 | Asn | ATT | 33.15 | 185 | ATTTGTTTGTTTTAGTGGTAAAAAGAAGATTATTAaattataagcaaaata … agatttcattttagtgTTCATAGACATAGGTTCAATTCTTAAGTGATTT |
| 21 | 74 | Asn | GTT | 83.97 |  | GCTCGATTAGCTCAGCTGGTTAGAGCATGCGGCTGTTAACCGCAAGGTCGTAGGTTCGATCCCTACATCGAGCG |
| 9 | 74 | Asn | GTT | 83.6 |  | GCTCGGTTAGCTCAGCTGGTTAGAGCATGCGGCTGTTAACCGCAAGGTCGAAGGTTCGATCCCTTCACCGAGCG |
| 1 | 75 | Asn | GTT | 42.81 |  | TCCGAGATAGTATAATGGTTAGTATTCCTGCCTGTTGATAGTCAGGAGAATCAGGTTCAATTCCTGGACTCGGAG |
| 31 | 72 | Asp | GTC | 78.7 |  | TCCGAGATAGTATAGTGGTTAGTATTCCTGCCTGTCACGCAGGAGACCCGGGTTCAATTCCCGGTCTCGGAG |
| 1 | 72 | Asp | GTC | 56.4 |  | TCCGAGATGGTGTAGTGGAAAGCATTGCAGCTTGTCTCGCTGCAGACCTGGGTTCAATTCCCAGTCTTGGAG |
| 1 | 71 | Cys | GCA | 71.29 |  | GGAACCTTAGCTTAGTGGTATAGCGTAGGACTGCAAAGCCTGAGTCGCCGGTTCGATCCCGGCAGGTTCCT |
| 2 | 72 | Cys | GCA | 78.73 |  | GGAGCCTTAGCTTAGTGGTATAGCGTAGGACTGCAAATCCTGAGGTCGCCGGTTCGATCCCGGCAGGTTCCT |
| 9 | 72 | Cys | GCA | 74.11 |  | GGGGCTTTAGCTCAGTGGCAGAGCGGAGGACTGCAAATCCTTAGGTCGCCGGTTCAATCCCGGCAGGTCCCT |
| 6 | 72 | Cys | GCA | 68.79 |  | GGGGCTTTAGCTCAGTGGCAGAGCGGAGGACTGCAAATCCTTAGGTCGCCGGTTCAATCCTGGCAGGTCCCT |
| 13 | 72 | Gln | TTG | 70.67 |  | GGTTGTATGGTGTAGCGGTTAGCACCGAGGACTTTGAATCCTCTGACCTGGGTTCGAATCCCAGTACGACCT |
| 1 | 83 | Gln | CTA | 30.5 |  | GCCGGCATGGTGAAATAGTAATTACGACGGTATCACGGAATCCTCTAACTCTTCAATTCTAGGTTCGAATCCTAGTGTCGGCG |
| 1 | 72 | Gln | CTA | 67.81 |  | GGTTCCATAGTATAGCAGTTAGTACTGGGGACTCTAAATCCCTTGACCTGGGTTCGAATCCCAGTGGGACCT |
| 7 | 72 | Gln | CTA | 74.47 |  | GGTTCTATAGTATAGCGGTTAGTACTGGGGACTCTAAATCCCTTGACCTGGGTTCGAATCCCAGTAGGACCT |
| 1 | 72 | Gln | TTA | 75.36 |  | GGTTCCATAGTATAGCGGTTAGTACTGGGGACTTTAAATCCCTTGACCTGGGTTCGAATCCCAGTGGGACCT |
| 1 | 66 | Gln | TTA | 35.76 |  | GGTTCCATAGTATAGTGGTCAGTACTGGGGACTTTAAATCCCTTGATCTTAGTTCGAATGGGACCT |
| 27 | 72 | Gln | TTA | 77.82 |  | GGTTCCATAGTATAGTGGTTAGTACTGGGGACTTTAAATCCCTTGACCTGGGTTCGAATCCCAGTGGGACCT |
| 1 | 72 | Gln | TTA | 41.14 |  | GGTTTCATAGTATAGTGCTTAGAACTGGGGACTTTAAATCCTTTGACTTGGGTTCGAATTCGAGTGGGACCT |
| 7 | 73 | Glu | CTC | 74.12 |  | TCCCTCATAGTCTAGCTGGTTAGGATACAAGGTTCTCACCCTTGAGACCCGGGTTCAAGTCCCGGTGGGGGAG |
| 1 | 73 | Glu | CTC | 79.63 |  | TCCCTCATAGTCTAGTTGGTTATGATACAAGGTTCTCACCCTTGAGACCCGGGTTCAAATCCCGGTGAGGGAG |
| 1 | 72 | Glu | TTC | 69.79 |  | TCCAGGATGGTGCAGTGGAAAGCATAGCAGCCTTTCAAGCTGCAGACCTGGGTTCAATTCCCAGTCTTGGAG |
| 36 | 73 | Glu | TTC | 66.75 |  | TCCCCTATAGTCTAGCAGGTTAGGATAGAAGGTTTTCACCCTTCAGACCCGGGTTCAAGTCCCGGTGGGGGAA |
| 1 | 71 | Gly | CCC | 68.9 |  | GCTACATTAATCTAGTGGTATGATAATTGCCTCCCACGCAATAAGCTCGGGTTCGATTCCCGAATGTAGCA |
| 29 | 71 | Gly | GCC | 73.9 |  | GCACCGATGGTTTAGTGGTAGAATAGAGCGTTGCCATCGCTTTGACCCGGGTTCGATTCCCGGTCGGTGCA |
| 2 | 71 | Gly | GCC | 73.42 |  | GCATCGATGGTTTAGTGGTAGAATAGAGCGTTGCCATCGCTTTGACCCGGGTTCGATTCCCGGTCGATGCA |
| 1 | 70 | Gly | TCC | 46.13 |  | AGCTGCGTTGGTCTAGTGGCAGGATTATTGCCTTCCAAGCAATCGGCTCGGGTTCGATTCCCGGANNNNN |
| 1 | 71 | Gly | TCC | 69.66 |  | GCAGCGTTGGTCTAATGGTAAGATAATTGCCTTCCAAGCAATAGACTCGGGTTCGATTCCCGGACGTTGCA |
| 9 | 71 | Gly | TCC | 72.22 |  | GCTGCGTTGGTCTAGTGGCAGGATTATTGCCTTCCAAGCAATCGGCTCGGGTTCGATTCCCGGACGCAGCA |
| 4 | 73 | His | GTG | 74.16 |  | GTGGATATAGCTCAGTTGGAAGAGCGTAAGATTGTGGCTCTTAAGGTCGTGGGATCGAAACCCACTATCCACA |
| 8 | 73 | His | GTG | 62.17 |  | GTGGATGTAGCTCAATCGGAAGAGCGTAAGATTGTGGCTCTTGAGGTAGAGGGATCGAAACCCTCCATCCACA |
| 1 | 73 | His | GTG | 74.56 |  | GTGGATGTAGCTCAGTTGGAAGAGCGTAAGATTGTGGCTCTTAAGGTCGTGGGATCGAAACCCACCATCCACA |
| 1 | 73 | His | GTG | 59.62 |  | GTGGATGTAGCTCAGTTGGAAGAGCGTAAGATTGTGGCTCTTAAGGTCGTGGGATCGAAACCCACCATCCNNN |
| 1 | 72 | His | GTG | 47.16 |  | TAAAGCGTGGTGCAGTGGAAGCATACCGGAACGTGTATTCGGTGGTCATGGGATCGAAACCCATCGCTTTAA |
| 1 | 72 | His | GTG | 46.44 |  | TAGAGCGTGGTGCAGTGGAAGCATACCGGAACGTGTATTCGGTGGTCATGGGATCGAAACCCATCGCTTTAA |
| 27 | 74 | Ile | AAT | 80.37 |  | GCTCGGGTAGCTCAGTTGGTTAGAGCGTGGGTCTAATAAACCCAAGGTCGAGGGTTCAAGCCCCTCCCGGAGCA |
| 1 | 74 | Ile | AAT | 77.41 |  | GCTCGGGTAGCTCAGTTGGTTAGAGCGTGGGTCTAATAAACCCGAGGTCGAGGGTTCAAGCCCCTCCCGGAGCA |
| 4 | 74 | Ile | TAT | 82.45 |  | GCTCAGATAGCTCAGTTGGTTAGAGCTTTGGTCTTATGAGCCAAAGGTCGTGGGTTCGAGCCCCACTTTGAGCA |
| 1 | 73 | Ile | TAT | 54.95 |  | GCTCAGGTAGCTCAACGGTTAGAGCAACGGTCTTATCAACCGGAGGTTGTGGGTTCAAGTCCCATCCTGTGTA |
| 1 | 73 | Ile | TAT | 59.14 |  | GCTCAGGTAGCTCAATGGTTAGAGCAACGGTCTTATCAACCGGAGGTTGTGGGTTCGAGTCCCATCCTGTGTA |
| 4 | 74 | Ile | TAT | 80.75 |  | GCTCAGGTAGCTCAGTTGGTTAGAGCGTTGGTCTTATGAGCCAACGGTCGTGGGTTCGACCCCCACCTTGAGCA |
| 1 | 93 | Ile | TAT | 50.89 | 20 | GGCTCAGTGGTGCAGCTGGTAAGCATACTCGATTTATTgaaccattgaagaaaagcttTTCGAGAGACGGAGGTTCAAATCCTCTCTGGGCTA |
| 1 | 99 | Ile | TAT | 56.03 | 26 | GGCTCAGTGGTGCAGCTGGTAAGCATGCTCGATTTATAtttgctgaccaaccttataaaagtgtGTCGAGAGACAGAGGTTCGAACCCTCTCTGAGCTA |
| 18 | 80 | Leu | AAG | 63.81 |  | GATGAAGTGGCCGAGCGGTCTAAGGCGGTAGATTAAGGCTCTATTCCGAAAGGGCGCGAGTTCGAATCTCGCCTTCATCA |
| 1 | 82 | Leu | CAA | 64.43 |  | GCACGTGTGGCCGAGCGGTCTAAGGCGGCGCACTCAAGTTGCGTTGGAGCGATCCGCGTGAGTTCAAATCTCACCGCGTGCA |
| 9 | 82 | Leu | CAA | 66.17 |  | GCACGTGTGGCCGAGCGGTCTAAGGCGGCGCACTCAAGTTGCGTTGGAGCGATCCGCGTGAGTTCGAATCTCACCGCGTGCA |
| 1 | 72 | Leu | CAG | 47.7 |  | CTGATCATAGCTTAATGGTTAAAGCAAGAGAATCAGGCTCTCTCTATCTTGGTTCGATTCCGAGTGGTCAGG |
| 1 | 80 | Leu | CAG | 63.71 |  | GATGAAGTGGCCGAGCGGTCTAAGGCGGTAGATTCAGGTTCTATTCCGAAAGGGCGCGAGTTCGAATCTCGCCTTCATCA |
| 1 | 81 | Leu | TAA | 40.54 |  | ACTTATGTGGCGGAATAGGTAGACGCGTGGGACTTAAAATCTCATTCCTACGGAGTGCCGGTTCGACCCCGGTCATAAGTA |
| 19 | 83 | Leu | TAA | 64.97 |  | GGCAATTTGCCCGAGTTGGTTAAGGGGGACGACTTAAGATCGCCTGGATGTAATCCGCGTGAGTTCGAATCTCACAGTTGCCA |
| 1 | 82 | Leu | TAA | 55.37 |  | GGTAGCTTGGCCGAGAGGTTAAGGCATGCGAGTTAAAATCGCTGGGATTATATCTGCGTGAGTTCGATTCTCACAGCTACCA |
| 3 | 80 | Leu | TAG | 65.32 |  | GATGAAGTGGCCGAGTGGTCTAAGGCGGTAGATTTAGGCTCTATTCCGAAAGGGCGAGGGTTCGAACCCCTCCTTCATCA |
| 33 | 73 | Lys | CTT | 85.3 |  | GCCCGCCTAGCTCAGTCGGTAGAGCGCAAGACTCTTAATCTTGTGGTCGTGGGTTCGAGCCCCACGGCGGGCT |
| 25 | 73 | Lys | TTT | 79.2 |  | ACCCGTCTAGCTCAGTCGGTAGAGCGCCAGCCTTTTAAGCTGGTGGTCGAGGGTTCGAGCCCCTCGGTGGGTG |
| 1 | 73 | Lys | TTT | 74.61 |  | ACCCGTCTAGCTCAGTCGGTAGAGCGCCAGCCTTTTAAGTTGGTGGTCGAGGGTTCGAGCCCCTCGGTGGGTG |
| 1 | 73 | Lys | TTT | 66.02 |  | ACCCGTCTAGCTCAGTCGGTAGAGTGCCAGCCTTTTAAGCTGGTGGTCGAGGGTTCGAGCCCCTAGGTAGGTG |
| 1 | 73 | Lys | TTT | 49.7 |  | ACCCGTCTAGCTCAGTCTGTAGAGCTCCAGCCTTTTAAGCTGGTGGTAGAGGGTTAGAGCCCCTCGGTGCGTG |
| 1 | 72 | Met | CAT | 60.14 |  | AGCAGGGTAGCGAAGTGGGATCGCGTTGTGCTCATAACTCAAAGGTCGGAGGATCGAAACCTCTCTCTGCTA |
| 1 | 72 | Met | CAT | 55.7 |  | AGCAGGGTGGCGAAGTGGAATCGCGTTGGGCTCATAACTCAAAGGGCGGAGGATCGAAGCCTCTCTCTGCTA |
| 19 | 72 | Met | CAT | 64.92 |  | AGCAGGGTGGCGAAGTGGAATCGCGTTGGGCTCATAACTCAAAGGTCGGAGGATCGAAACCTCTCTCTGCTA |
| 12 | 74 | Met | CAT | 77.09 |  | GCCCGTGTAGCTCAGTTGGTTAGAGCGCGAGTCTCATAATCTCGAGGCCGAGGGTTCGATCCCCTCCGCAGGCA |
| 11 | 73 | Phe | GAA | 76.72 |  | GCCTTTATAGCTCAGCTGGGAGAGCGTAAGACTGAAGATCTTACGGTCCTCGGTTCGATCCCGAGTGAAGGCA |
| 1 | 73 | Phe | GAA | 68.81 |  | GCCTTTATAGCTCAGCTGGGAGAGCGTAAGACTGAAGATCTTACGGTCCTCGGTTCGATCCCGAGTGAAGGNN |
| 12 | 73 | Phe | GAA | 79.17 |  | GCCTTTATAGCTCAGTTGGGAGAGCGTAAGACTGAAGATCTTACGGTCCTCGGTTCGATCCCGAGTGAAGGCA |
| 20 | 72 | Pro | AGG | 64.29 |  | GGGTTGTTGGTCTAGTGGCATGATTCTCGTTTAGGGTACGAGAGGTCCCGGGTTCGATTCTCGGACGACCCC |
| 1 | 71 | Pro | CGG | 67.76 |  | GGGCTTGTGGTTTAGTGGTAGAATGCCAGTTTCGGGTACTGGCGATCCGGGTTCGATTCCCGGCAAGCCCT |
| 7 | 72 | Pro | TGG | 71.23 |  | GGGCAATTGGTCTAGTGGTATGATTCTCGTTTTGGGTACGAGAGGTCCCGGGTTCAATCCCCGGATTGCCCC |
| 1 | 81 | Ser | AGA | 76.5 |  | GACAATATGTCCGAGTGGTTAAGGAGGAAGACTAGAAATCTTTTGGGTTTACCCGCGCAGGTTCGAATCCTGCTGTTGTCG |
| 17 | 81 | Ser | AGA | 75.92 |  | GACAATTTGTCCGAGTGGTTAAGGAGGAAGACTAGAAATCTTTTGGGTTTACCCGCGCAGGTTCGAATCCTGCAGTTGTCG |
| 1 | 82 | Ser | AGA | 61.05 |  | GATACCTTGGCTGAGTGGTAAAAGCGGCGGTCTAGAAATCCGTTTCCTATCAGGAGTGAGAGTTCGATTCTCTCAGGTATCG |
| 1 | 82 | Ser | AGA | 60.04 |  | GATACCTTGGCTGAGTGGTAAAAGCGGCGGTCTAGAAATCCGTTTCCTGTCAGGAGTGAGAGTTCAATTCTCTCAGGTATCA |
| 2 | 81 | Ser | CGA | 74.6 |  | GTCAGTTTGTCCGAGTGGTTAAGGAGGTTGACTCGAAATCAACTGGGTTTACCCGCACAGGTTCAAATCCTGTAGCTGACG |
| 15 | 82 | Ser | GCT | 79.36 |  | GACACTTTGGCCGAGTGGTTAAGGCGGCGGCCTGCTAAGCCGTTTCCCTCTGGGAGCGTGAGTTCAATTCTCACAGGTGTCG |
| 1 | 82 | Ser | TGA | 62.47 |  | GATACCTTGGCTGAGTGGTAAAAGCGGCGGTCTTGAAATCCGTTTCCTTTGAGGAGTGAGAGTTCGATTCTCTCAGGTATCG |
| 8 | 81 | Ser | TGA | 80.21 |  | GTCAGTTTGTCCGAGTGGTTAAGGAGGTGGACTTGAAATCCATTGGGTTTACCCGCGCAGGTTCGAATCCTGCAGCTGACG |
| 22 | 73 | Thr | AGT | 81.41 |  | GCCGCTTTAGCTCAGTGGTTAGAGCATTCGCCTAGTAAGCGAAAGGTCGGCGGTTCAATCCCGCCAAGTGGCT |
| 1 | 73 | Thr | CGT | 82.22 |  | GCCGGCATAGCAAAGTGGTTAATGCGTCTGCCTCGTAAGCAGAAGGTCGCCAGTTCGATTCTGGCTGTCGGCT |
| 6 | 73 | Thr | TGT | 83.2 |  | GCCTGTATAGCACAGTGGTTAGTGCATCTGCCTTGTAAGCAGAAGGCCGCCGGTTCGAATCCGGCTACAGGCT |
| 1 | 73 | Trp | CCA | 50.4 |  | AGAATTGTAGCTCAGTTGGAAGAGCGGAGGAATCCAGATCCTTTGGTCAAAGGTTCAAACCTTTTCAATTCCC |
| 1 | 73 | Trp | CCA | 62.47 |  | GGAATTGTAGCTCAGCTGGGAGAGCGAAAGAATCCAAATCTTTAGGTCAAAGGTTCAAATCCTTTCAATTCCC |
| 1 | 73 | Trp | CCA | 56.86 |  | GGAATTGTAGCTCAGTTGGGAGAGCAGAGGAATCCAGATCCTCCGGTCAAAGGTTCAAACCCTTTCAATTCCC |
| 1 | 73 | Trp | CCA | 65.43 |  | GGAATTGTAGCTCAGTTGGGAGAGCGGAAGAATCCAAATCTTTAGGTCAAAGGTTCAAATCCTTTCAATTCCC |
| 1 | 73 | Trp | CCA | 48.04 |  | GGAATTGTAGCTCAGTTGGTAGAGCGGAGGCAGCCAAAACCTCAGGCCAAAGGTTCAAGTCCTTTCAATTCCC |
| 1 | 73 | Trp | CCA | 61.75 |  | GGAGACTTAGCTCAAATGGTAGAGCGTTGGACTCCAAATCCAACGGTTGCGTGTTCGAGTCACATAGTCTTCA |
| 1 | 73 | Trp | CCA | 68.85 |  | GGAGACTTAGCTCAAATGGTAGAGCGTTGGACTCCAAATCCAACGGTTGCGTGTTCGAGTCACGTAGTCTTCA |
| 6 | 73 | Trp | CCA | 70.33 |  | GGAGACTTAGCTCAAGTGGTAGAGCGCTGGACTCCAAATCCAGCGGTTGCGTGTTCGAGTCACGTAGTCTTCA |
| 3 | 73 | Trp | CCA | 69.94 |  | GGAGACTTAGCTCAAGTGGTAGAGCGTTGGACTCCAAATCCAACGGTTGCGTGTTCGAGTCACGTAGTCTTCA |
| 1 | 72 | Trp | CCA | 75.08 |  | GGAGACTTAGCTCAATGGTAGAGCGCTGGACTCCAAATCCAGCGGTTGCGTGTTCGATTCACGTAGTCTTCA |
| 2 | 72 | Trp | CCA | 56.67 |  | GGGCAATTGGTCTATCAGGTAGGATTCTCGTCTCCAAATCGAGAGATCCAGGTTCGATTCCTGGATTGCCCC |
| 1 | 72 | Trp | CCA | 53.12 |  | GGGCAATTGGTCTATCAGGTAGGATTCTCGTTTCCAAATCGAGAGATCCAGGTTCAATTCCTGGATTGCCCC |
| 1 | 72 | Trp | CCA | 56.99 |  | GGGCAATTGGTCTATTAGGTAGGATTCTCGTCTCCAATTCGAGAGATCCAGGTTCGATTCCTGGATTGCCCC |
| 1 | 72 | Trp | CCA | 58.14 |  | GGGCAATTGGTCTATTAGGTAGGATTCTCGTCTCCAGAGCGAGAGATCCAGGTTCGATTCCTGGATTGCCCC |
| 1 | 83 | Tyr | GTA | 56.24 | 11 | CCAAATTTAGCTCAGTTGGAAGAGCGTCGGACTGTAAagtttgtagtcATCCTTAGGTCCCGGTTCGAACCCGGGAGTTTGGA |
| 1 | 84 | Tyr | GTA | 60.38 | 11 | CCGAACTTAGCTCAGCTGGAAGAGCGTCGGACTGTAAagtttgtagtcATCCGTAGGTCCCCAGTTCGATCCCGGGAGTTCGGA |
| 17 | 84 | Tyr | GTA | 70.2 | 11 | CCGAACTTAGCTCAGTTGGAAGAGCATCGGACTGTAAagtttgtagtcATCCGGTGGTCCCCGGTTCGAACCCGGGAGTTCGGA |
| 21 | 73 | Val | AAC | 79.84 |  | GATTCCTTAGTGTAGTGGTTATCACGTCAGTCTAACACACTGAAGGTCCCGAGTTCAAACCTCGGAGGAATCA |
| 1 | 73 | Val | CAC | 75.86 |  | GATTCCTTAGTGAAGTGGTTATCACGTCAGTCTCACACACTGAAGGTCCCGAGTTCAATCCTCGGAGGAATCA |
| 1 | 83 | Val | CAC | 47.23 | 11 | GTCGGCATGGTGAAGCGGTAATCACGAAGGTATCACGaaggtcttatgAACCTTAATCCTAGGTTCAAACCCTAGTGTCGACG |
| 1 | 73 | Val | CAC | 72.56 |  | GTTCGTTTAGTGTAGTGGTTATCACGTTCCCTTCACACGGGAAAGGCCCCGAGTTCGACCCTCGGAATGAACA |
| 1 | 73 | Val | CAC | 77.61 |  | GTTCGTTTAGTGTAGTGGTTATCACGTTCCCTTCACACGGGAAAGGTCCCGAGTTCAATCCTCGGAATGAACA |
| 1 | 73 | Val | TAC | 72.83 |  | GATTCCTTAGCGTAGTGGTTAGCATGTCAGTCTTACACACTGAAGGTCCCGAGTTCAATCCTCGGAGGAATCA |
| 2 | 73 | Val | TAC | 79.85 |  | GATTCCTTAGTGTAGTGGTTATCACGTCAGTCTTACACACTGAAGGTCCCGAGTTCAATCCTCGGAGGAATCA |
| 3 | 73 | Val | TAC | 78.4 |  | GATTTCTTAGTGTAGTGGTTATCACGTCAGTCTTACACACTGAAGGTCCCGAGTTCAATCCTCGGAGAAATCA |
| 1 | 71 | Undet | ??? | 41.36 |  | ACCAGGATGGTGTAGAGGAAAGCATAGCAGCCGTCCAGCTGCAGACCTGGGTTCAATTCCCAGTCTTGGAA |
| 1 | 101 | Undet | ??? | 49.94 |  | GGCTCAGTGGTGCAGCTGGTAAGCATGCTCGATTTAACGTTCATCCTCCTACATTTAGAAAAGAGTGTCGAGAGACAGAGGTTCGAACCCTCTCTGAGCTA |
| 1 | 71 | Undet | ??? | 39 |  | GTTACTTTGGTCTAATGGTAAAGACGGCTGGGTCCAATCTGCAGATCTGGGTTCGATTCCTAGAAGTAACA |
| 1 | 81 | Leu | AAG | 32.06 |  | GATGGCGTGCCTGAGTGGTCAAAGGGATAAGCGAAGGGCTTGTTGCTCAAAAGCATGTGGGTTCGATTCCCATCGCCATTA |
| 1 | 227 | Leu | CAA | 21.42 | 53 | AATGAAATATTTGAATGGAATAATTGAATATTCAAAagacaaatatggttt … acattttttgcatatATTTTTTGTTTATTCAGGTTCGAAACCTATTTCCATAC |
| 1 | 62 | Leu | CAA | 21.54 |  | GCACGTGTGGCCGAGCGGTCTAAGGCGGCGCACTCAAGTTGCGTTGGAGCGATCCGCGTGAG |
| 1 | 73 | Phe | AAA | 36.59 |  | TCTCGGATAGCTTAGTTGGTTATAGTATGGGTTAAATAACCCAAGATCGAGGGTTCAAGCCCTTCCTGGAGCA |
| 1 | 83 | Val | CAC | 40.33 | 11 | GTCGGCATGGTGAAGTAGTAATCACGAAGGTATCACGaaggtctaataAACCTTAATCCTAGGTTCAAACCCTAGTGTCGACT |

C. Additional non-coding RNA loci detected by genome-wide covariance model search algorithms.

| **ncRNA** | **Copy number** | **Scaffold** | **Position** | **Number of Genbank Entries** |
| --- | --- | --- | --- | --- |
| SRP (7SL) | 3 | 8254688  8254737 | 155342-629  176688-403; 177398-117 | 1 |
| U1 | 6 | 8254650 | 330349-512; 346220-383; 359115-278; 373766-926; 374564-727; 375189-352 | 3-4 |
| U2 | 4 | 8254377  8254824 | 199075-265; 200019-209  48092-7903; 48695-506 | 4-6 |
| U2var | 5 | 8254377 | 204406-594; 205383-571; 206412-600; 207338-526; 208157-345 | 0 |
| U3 | 4 | 8253957  8254479  8254484  8254676 | 67983-67730  40042-39789  82748-82495  70077-69824 | 1-2 |
| U4 | 4 | 8254594 | 336229-358; 337644-779; 338505-640; 339192-327 | 1 |
| U5 | 2 | 8254652 | 481732-620; 482567-455 | 3 |
| U6 | 4 | 8253894 | 88976-873; 89347-244; 90524-423; 91780-677 | 4 |

D. Accession numbers for all *T. thermophila* tRNA genes referred to in Table S6B.

| **Type** | **Anticodon** | **Genbank Accession** |
| --- | --- | --- |
| Ala | CGC | TTHERM_01044480 |
| Ala | TGC | TTHERM_01044450 |
| Ala | AGC | TTHERM_00726190 |
| Ala | AGC | TTHERM_00815160 |
| Ala | AGC | TTHERM_00815190 |
| Ala | AGC | TTHERM_02544190 |
| Ala | AGC | TTHERM_00292210 |
| Ala | AGC | TTHERM_00292230 |
| Ala | AGC | TTHERM_00292240 |
| Ala | AGC | TTHERM_01178690 |
| Ala | AGC | TTHERM_01178730 |
| Ala | AGC | TTHERM_00346820 |
| Ala | AGC | TTHERM_00346830 |
| Ala | AGC | TTHERM_00346850 |
| Ala | AGC | TTHERM_00346860 |
| Ala | AGC | TTHERM_00346870 |
| Ala | AGC | TTHERM_00418390 |
| Ala | AGC | TTHERM_00418420 |
| Ala | AGC | TTHERM_00418440 |
| Ala | AGC | TTHERM_00418460 |
| Ala | AGC | TTHERM_00421030 |
| Ala | AGC | TTHERM_00471250 |
| Ala | AGC | TTHERM_00547990 |
| Ala | AGC | TTHERM_00548250 |
| Ala | AGC | TTHERM_00548260 |
| Ala | AGC | TTHERM_00548000 |
| Ala | AGC | TTHERM_00548010 |
| Ala | AGC | TTHERM_00548180 |
| Ala | AGC | TTHERM_00548200 |
| Ala | AGC | TTHERM_00548210 |
| Ala | AGC | TTHERM_00548220 |
| Ala | AGC | TTHERM_00548230 |
| Ala | AGC | TTHERM_00548240 |
| Ala | TGC | TTHERM_01044450 |
| Ala | TGC | TTHERM_01044540 |
| Ala | TGC | TTHERM_01044550 |
| Ala | TGC | TTHERM_01044460 |
| Ala | TGC | TTHERM_01044470 |
| Ala | TGC | TTHERM_01044490 |
| Ala | TGC | TTHERM_01044500 |
| Ala | TGC | TTHERM_01044510 |
| Ala | TGC | TTHERM_01044520 |
| Ala | TGC | TTHERM_01044530 |
| Ala | TGC | TTHERM_00582120 |
| Arg | CCG | TTHERM_00312250 |
| Arg | TCG | TTHERM_00494310 |
| Arg | TCT | TTHERM_00681810 |
| Arg | ACG | TTHERM_00673310 |
| Arg | ACG | TTHERM_00673320 |
| Arg | ACG | TTHERM_00673330 |
| Arg | ACG | TTHERM_00673340 |
| Arg | ACG | TTHERM_00673350 |
| Arg | ACG | TTHERM_00673370 |
| Arg | ACG | TTHERM_00673380 |
| Arg | ACG | TTHERM_00673390 |
| Arg | CCT | TTHERM_01146070 |
| Arg | CCT | TTHERM_01146080 |
| Arg | TCT | TTHERM_00681810 |
| Arg | TCT | TTHERM_00301980 |
| Arg | TCT | TTHERM_00301990 |
| Arg | TCT | TTHERM_00302000 |
| Arg | TCT | TTHERM_00302020 |
| Arg | TCT | TTHERM_01230050 |
| Arg | TCT | TTHERM_01230060 |
| Arg | TCT | TTHERM_01230070 |
| Arg | TCT | TTHERM_01230080 |
| Arg | TCT | TTHERM_00338130 |
| Arg | TCT | TTHERM_00340220 |
| Arg | TCT | TTHERM_00418020 |
| Arg | TCT | TTHERM_00418060 |
| Arg | TCT | TTHERM_00418410 |
| Arg | TCT | TTHERM_00418480 |
| Arg | TCT | TTHERM_00581620 |
| Arg | TCT | TTHERM_00129080 |
| Arg | TCT | TTHERM_00129100 |
| Arg | TCT | TTHERM_00129110 |
| Arg | TCT | TTHERM_00129130 |
| Arg | TCT | TTHERM_00129140 |
| Arg | TCT | TTHERM_00129150 |
| Arg | TCT | TTHERM_00129170 |
| Arg | TCT | TTHERM_00129190 |
| Arg | TCT | TTHERM_00129200 |
| Asn | ATT | TTHERM_00107050 |
| Asn | GTT | TTHERM_00011060 |
| Asn | GTT | TTHERM_00011060 |
| Asn | GTT | TTHERM_00011070 |
| Asn | GTT | TTHERM_00011080 |
| Asn | GTT | TTHERM_00011090 |
| Asn | GTT | TTHERM_00011100 |
| Asn | GTT | TTHERM_00011110 |
| Asn | GTT | TTHERM_00765160 |
| Asn | GTT | TTHERM_00772050 |
| Asn | GTT | TTHERM_00802380 |
| Asn | GTT | TTHERM_00241560 |
| Asn | GTT | TTHERM_00241570 |
| Asn | GTT | TTHERM_01031240 |
| Asn | GTT | TTHERM_01067000 |
| Asn | GTT | TTHERM_00265130 |
| Asn | GTT | TTHERM_00265160 |
| Asn | GTT | TTHERM_00265170 |
| Asn | GTT | TTHERM_01230140 |
| Asn | GTT | TTHERM_00310610 |
| Asn | GTT | TTHERM_00310630 |
| Asn | GTT | TTHERM_00343660 |
| Asn | GTT | TTHERM_00354660 |
| Asn | GTT | TTHERM_00355930 |
| Asn | GTT | TTHERM_00394680 |
| Asn | GTT | TTHERM_00399230 |
| Asn | GTT | TTHERM_00399250 |
| Asn | GTT | TTHERM_00457090 |
| Asn | GTT | TTHERM_00467820 |
| Asn | GTT | TTHERM_00538780 |
| Asn | GTT | TTHERM_00558170 |
| Asn | GTT | TTHERM_00584830 |
| Asn | GTT | TTHERM_00648700 |
| Asp | GTC | TTHERM_00016310 |
| Asp | GTC | TTHERM_00753270 |
| Asp | GTC | TTHERM_02230820 |
| Asp | GTC | TTHERM_00818300 |
| Asp | GTC | TTHERM_00818360 |
| Asp | GTC | TTHERM_00819460 |
| Asp | GTC | TTHERM_00191570 |
| Asp | GTC | TTHERM_00895860 |
| Asp | GTC | TTHERM_00895880 |
| Asp | GTC | TTHERM_00895890 |
| Asp | GTC | TTHERM_00942760 |
| Asp | GTC | TTHERM_00245760 |
| Asp | GTC | TTHERM_00268180 |
| Asp | GTC | TTHERM_00275820 |
| Asp | GTC | TTHERM_01151510 |
| Asp | GTC | TTHERM_00320100 |
| Asp | GTC | TTHERM_00320140 |
| Asp | GTC | TTHERM_00346720 |
| Asp | GTC | TTHERM_00079500 |
| Asp | GTC | TTHERM_00474770 |
| Asp | GTC | TTHERM_00550930 |
| Asp | GTC | TTHERM_00550960 |
| Asp | GTC | TTHERM_00550980 |
| Asp | GTC | TTHERM_00550990 |
| Asp | GTC | TTHERM_00572210 |
| Asp | GTC | TTHERM_00581520 |
| Asp | GTC | TTHERM_00581530 |
| Asp | GTC | TTHERM_00581540 |
| Asp | GTC | TTHERM_00630250 |
| Asp | GTC | TTHERM_00630290 |
| Asp | GTC | TTHERM_00630390 |
| Asp | GTC | TTHERM_00648710 |
| Cys | GCA | TTHERM_02199760 |
| Cys | GCA | TTHERM_02199760 |
| Cys | GCA | TTHERM_02199770 |
| Cys | GCA | TTHERM_00895980 |
| Cys | GCA | TTHERM_00896000 |
| Cys | GCA | TTHERM_00896010 |
| Cys | GCA | TTHERM_00896020 |
| Cys | GCA | TTHERM_00896030 |
| Cys | GCA | TTHERM_00896040 |
| Cys | GCA | TTHERM_00896050 |
| Cys | GCA | TTHERM_00896060 |
| Cys | GCA | TTHERM_01108730 |
| Cys | GCA | TTHERM_00483690 |
| Cys | GCA | TTHERM_00569360 |
| Cys | GCA | TTHERM_00569370 |
| Cys | GCA | TTHERM_00120840 |
| Cys | GCA | TTHERM_00120850 |
| Cys | GCA | TTHERM_00120860 |
| Cys | GCA | TTHERM_00120870 |
| Gln | TTG | TTHERM_01034370 |
| Gln | TTG | TTHERM_01034380 |
| Gln | TTG | TTHERM_01087760 |
| Gln | TTG | TTHERM_00372370 |
| Gln | TTG | TTHERM_00372390 |
| Gln | TTG | TTHERM_00372400 |
| Gln | TTG | TTHERM_00441890 |
| Gln | TTG | TTHERM_00441920 |
| Gln | TTG | TTHERM_00599690 |
| Gln | TTG | TTHERM_00599710 |
| Gln | TTG | TTHERM_00599720 |
| Gln | TTG | TTHERM_00599730 |
| Gln | TTG | TTHERM_00599740 |
| Glu | CTC | TTHERM_00826930 |
| Glu | TTC | TTHERM_00717740 |
| Glu | CTC | TTHERM_00826930 |
| Glu | CTC | TTHERM_00926930 |
| Glu | CTC | TTHERM_00310600 |
| Glu | CTC | TTHERM_00310830 |
| Glu | CTC | TTHERM_00310840 |
| Glu | CTC | TTHERM_00310850 |
| Glu | CTC | TTHERM_00310860 |
| Glu | TTC | TTHERM_00717740 |
| Glu | TTC | TTHERM_00770860 |
| Glu | TTC | TTHERM_00770900 |
| Glu | TTC | TTHERM_00770920 |
| Glu | TTC | TTHERM_00771940 |
| Glu | TTC | TTHERM_00797850 |
| Glu | TTC | TTHERM_00797860 |
| Glu | TTC | TTHERM_00823560 |
| Glu | TTC | TTHERM_00823570 |
| Glu | TTC | TTHERM_00927020 |
| Glu | TTC | TTHERM_00974170 |
| Glu | TTC | TTHERM_00974180 |
| Glu | TTC | TTHERM_00974190 |
| Glu | TTC | TTHERM_00974200 |
| Glu | TTC | TTHERM_00974210 |
| Glu | TTC | TTHERM_00974220 |
| Glu | TTC | TTHERM_00974230 |
| Glu | TTC | TTHERM_00974240 |
| Glu | TTC | TTHERM_00974250 |
| Glu | TTC | TTHERM_01001310 |
| Glu | TTC | TTHERM_00259440 |
| Glu | TTC | TTHERM_00259480 |
| Glu | TTC | TTHERM_00266220 |
| Glu | TTC | TTHERM_00277450 |
| Glu | TTC | TTHERM_00279730 |
| Glu | TTC | TTHERM_00279740 |
| Glu | TTC | TTHERM_00279760 |
| Glu | TTC | TTHERM_00304230 |
| Glu | TTC | TTHERM_00313860 |
| Glu | TTC | TTHERM_00353390 |
| Glu | TTC | TTHERM_00353400 |
| Glu | TTC | TTHERM_00373740 |
| Glu | TTC | TTHERM_00077740 |
| Glu | TTC | TTHERM_00095420 |
| Glu | TTC | TTHERM_00095430 |
| Glu | TTC | TTHERM_00559730 |
| Glu | TTC | TTHERM_00559740 |
| Gly | CCC | TTHERM_00600060 |
| Gly | TCC | TTHERM_00238670 |
| Gly | TCC | TTHERM_00238670 |
| Gly | GCC | TTHERM_00001100 |
| Gly | GCC | TTHERM_00001110 |
| Gly | GCC | TTHERM_00784750 |
| Gly | GCC | TTHERM_00784780 |
| Gly | GCC | TTHERM_00851660 |
| Gly | GCC | TTHERM_00851670 |
| Gly | GCC | TTHERM_00863820 |
| Gly | GCC | TTHERM_00925500 |
| Gly | GCC | TTHERM_00219550 |
| Gly | GCC | TTHERM_00994070 |
| Gly | GCC | TTHERM_01018360 |
| Gly | GCC | TTHERM_00037520 |
| Gly | GCC | TTHERM_01089050 |
| Gly | GCC | TTHERM_01090080 |
| Gly | GCC | TTHERM_01090100 |
| Gly | GCC | TTHERM_01151470 |
| Gly | GCC | TTHERM_01151490 |
| Gly | GCC | TTHERM_01413090 |
| Gly | GCC | TTHERM_01413100 |
| Gly | GCC | TTHERM_00486080 |
| Gly | GCC | TTHERM_00532420 |
| Gly | GCC | TTHERM_00532430 |
| Gly | GCC | TTHERM_00629790 |
| Gly | GCC | TTHERM_00629820 |
| Gly | GCC | TTHERM_00629830 |
| Gly | GCC | TTHERM_00629870 |
| Gly | GCC | TTHERM_00629880 |
| Gly | GCC | TTHERM_00629900 |
| Gly | GCC | TTHERM_00630070 |
| Gly | GCC | TTHERM_00630080 |
| Gly | GCC | TTHERM_00630090 |
| Gly | TCC | TTHERM_00238670 |
| Gly | TCC | TTHERM_00238680 |
| Gly | TCC | TTHERM_00238690 |
| Gly | TCC | TTHERM_00238700 |
| Gly | TCC | TTHERM_00238710 |
| Gly | TCC | TTHERM_00238720 |
| Gly | TCC | TTHERM_00293390 |
| Gly | TCC | TTHERM_00531950 |
| Gly | TCC | TTHERM_00531960 |
| Gly | TCC | TTHERM_00600040 |
| Gly | TCC | TTHERM_00600050 |
| His | GTG | TTHERM_02564200 |
| His | GTG | TTHERM_02564220 |
| His | GTG | TTHERM_00446160 |
| His | GTG | TTHERM_00446170 |
| His | GTG | TTHERM_00501030 |
| His | GTG | TTHERM_00501040 |
| His | GTG | TTHERM_00501050 |
| His | GTG | TTHERM_00501070 |
| His | GTG | TTHERM_00501080 |
| His | GTG | TTHERM_00502080 |
| His | GTG | TTHERM_00106730 |
| His | GTG | TTHERM_00106740 |
| His | GTG | TTHERM_00106750 |
| His | GTG | TTHERM_00106760 |
| His | GTG | TTHERM_00106770 |
| His | GTG | TTHERM_00106780 |
| Ile | AAT | TTHERM_00821970 |
| Ile | TAT | TTHERM_00856690 |
| Ile | TAT | TTHERM_00856690 |
| Ile | TAT | TTHERM_00856690 |
| Ile | TAT | TTHERM_00856690 |
| Ile | AAT | TTHERM_00821970 |
| Ile | AAT | TTHERM_00821990 |
| Ile | AAT | TTHERM_00822000 |
| Ile | AAT | TTHERM_00822010 |
| Ile | AAT | TTHERM_00192160 |
| Ile | AAT | TTHERM_01206410 |
| Ile | AAT | TTHERM_01206500 |
| Ile | AAT | TTHERM_01206520 |
| Ile | AAT | TTHERM_01206530 |
| Ile | AAT | TTHERM_01206540 |
| Ile | AAT | TTHERM_01206550 |
| Ile | AAT | TTHERM_01206420 |
| Ile | AAT | TTHERM_01206430 |
| Ile | AAT | TTHERM_01206440 |
| Ile | AAT | TTHERM_01206450 |
| Ile | AAT | TTHERM_01206460 |
| Ile | AAT | TTHERM_01206470 |
| Ile | AAT | TTHERM_01206480 |
| Ile | AAT | TTHERM_01206490 |
| Ile | AAT | TTHERM_00305520 |
| Ile | AAT | TTHERM_00305530 |
| Ile | AAT | TTHERM_00305540 |
| Ile | AAT | TTHERM_00305550 |
| Ile | AAT | TTHERM_00305560 |
| Ile | AAT | TTHERM_00338040 |
| Ile | AAT | TTHERM_00338060 |
| Ile | AAT | TTHERM_00433630 |
| Ile | AAT | TTHERM_00560090 |
| Ile | TAT | TTHERM_00856690 |
| Ile | TAT | TTHERM_00856720 |
| Ile | TAT | TTHERM_00856730 |
| Ile | TAT | TTHERM_00856740 |
| Ile | TAT | TTHERM_00310260 |
| Ile | TAT | TTHERM_01307990 |
| Ile | TAT | TTHERM_01308000 |
| Ile | TAT | TTHERM_00440390 |
| Ile | TAT | TTHERM_00440400 |
| Ile | TAT | TTHERM_00440410 |
| Ile | TAT | TTHERM_00630190 |
| Ile | TAT | TTHERM_00630240 |
| Leu | AAG | TTHERM_00850610 |
| Leu | CAA | TTHERM_00393160 |
| Leu | CAA | TTHERM_00393160 |
| Leu | CAA | TTHERM_00393160 |
| Leu | CAG | TTHERM_01114150 |
| Leu | CAG | TTHERM_01114150 |
| Leu | TAA | TTHERM_00014900 |
| Leu | TAA | TTHERM_00014900 |
| Leu | AAG | TTHERM_00850610 |
| Leu | AAG | TTHERM_00992900 |
| Leu | AAG | TTHERM_01048120 |
| Leu | AAG | TTHERM_00265000 |
| Leu | AAG | TTHERM_00265020 |
| Leu | AAG | TTHERM_00265030 |
| Leu | AAG | TTHERM_00265040 |
| Leu | AAG | TTHERM_00265050 |
| Leu | AAG | TTHERM_00265070 |
| Leu | AAG | TTHERM_00265080 |
| Leu | AAG | TTHERM_00338190 |
| Leu | AAG | TTHERM_00338430 |
| Leu | AAG | TTHERM_00558450 |
| Leu | AAG | TTHERM_00644720 |
| Leu | AAG | TTHERM_00655770 |
| Leu | AAG | TTHERM_00655790 |
| Leu | AAG | TTHERM_00655800 |
| Leu | AAG | TTHERM_00655810 |
| Leu | CAA | TTHERM_00393160 |
| Leu | CAA | TTHERM_00393170 |
| Leu | CAA | TTHERM_00393190 |
| Leu | CAA | TTHERM_00393210 |
| Leu | CAA | TTHERM_00393220 |
| Leu | CAA | TTHERM_00471180 |
| Leu | CAA | TTHERM_00471190 |
| Leu | CAA | TTHERM_01934230 |
| Leu | CAA | TTHERM_01934250 |
| Leu | CAA | TTHERM_01934260 |
| Leu | TAA | TTHERM_00014900 |
| Leu | TAA | TTHERM_00753500 |
| Leu | TAA | TTHERM_00753510 |
| Leu | TAA | TTHERM_00753520 |
| Leu | TAA | TTHERM_00753560 |
| Leu | TAA | TTHERM_00808090 |
| Leu | TAA | TTHERM_00809170 |
| Leu | TAA | TTHERM_00842710 |
| Leu | TAA | TTHERM_00227030 |
| Leu | TAA | TTHERM_01230040 |
| Leu | TAA | TTHERM_00319940 |
| Leu | TAA | TTHERM_01347880 |
| Leu | TAA | TTHERM_00374920 |
| Leu | TAA | TTHERM_00462970 |
| Leu | TAA | TTHERM_01813900 |
| Leu | TAA | TTHERM_00576830 |
| Leu | TAA | TTHERM_00576840 |
| Leu | TAA | TTHERM_00576850 |
| Leu | TAA | TTHERM_00576860 |
| Leu | TAA | TTHERM_00585110 |
| Leu | TAA | TTHERM_02039500 |
| Leu | TAG | TTHERM_00859140 |
| Leu | TAG | TTHERM_00859160 |
| Leu | TAG | TTHERM_00859170 |
| Lys | TTT | TTHERM_00005960 |
| Lys | TTT | TTHERM_00005960 |
| Lys | TTT | TTHERM_00005960 |
| Lys | CTT | TTHERM_00709720 |
| Lys | CTT | TTHERM_00721850 |
| Lys | CTT | TTHERM_00721860 |
| Lys | CTT | TTHERM_00777330 |
| Lys | CTT | TTHERM_00777340 |
| Lys | CTT | TTHERM_00777350 |
| Lys | CTT | TTHERM_00155490 |
| Lys | CTT | TTHERM_00155500 |
| Lys | CTT | TTHERM_00823860 |
| Lys | CTT | TTHERM_00823870 |
| Lys | CTT | TTHERM_00856540 |
| Lys | CTT | TTHERM_00856560 |
| Lys | CTT | TTHERM_00939080 |
| Lys | CTT | TTHERM_01085420 |
| Lys | CTT | TTHERM_01085450 |
| Lys | CTT | TTHERM_01085490 |
| Lys | CTT | TTHERM_00259460 |
| Lys | CTT | TTHERM_00259500 |
| Lys | CTT | TTHERM_00275900 |
| Lys | CTT | TTHERM_00277440 |
| Lys | CTT | TTHERM_00051960 |
| Lys | CTT | TTHERM_00353470 |
| Lys | CTT | TTHERM_00069620 |
| Lys | CTT | TTHERM_00415760 |
| Lys | CTT | TTHERM_00415770 |
| Lys | CTT | TTHERM_00466150 |
| Lys | CTT | TTHERM_00466160 |
| Lys | CTT | TTHERM_00466170 |
| Lys | CTT | TTHERM_00519860 |
| Lys | CTT | TTHERM_00129250 |
| Lys | CTT | TTHERM_00129260 |
| Lys | CTT | TTHERM_00129280 |
| Lys | CTT | TTHERM_00639990 |
| Lys | TTT | TTHERM_00005960 |
| Lys | TTT | TTHERM_00005980 |
| Lys | TTT | TTHERM_00006000 |
| Lys | TTT | TTHERM_00822310 |
| Lys | TTT | TTHERM_00822320 |
| Lys | TTT | TTHERM_00243760 |
| Lys | TTT | TTHERM_01122710 |
| Lys | TTT | TTHERM_01122720 |
| Lys | TTT | TTHERM_01165200 |
| Lys | TTT | TTHERM_01165220 |
| Lys | TTT | TTHERM_01293250 |
| Lys | TTT | TTHERM_01399560 |
| Lys | TTT | TTHERM_01399570 |
| Lys | TTT | TTHERM_01399590 |
| Lys | TTT | TTHERM_01399610 |
| Lys | TTT | TTHERM_01399630 |
| Lys | TTT | TTHERM_01415230 |
| Lys | TTT | TTHERM_01415240 |
| Lys | TTT | TTHERM_01498840 |
| Lys | TTT | TTHERM_01498850 |
| Lys | TTT | TTHERM_01520350 |
| Lys | TTT | TTHERM_01545810 |
| Lys | TTT | TTHERM_01545820 |
| Lys | TTT | TTHERM_00497530 |
| Lys | TTT | TTHERM_00106710 |
| Lys | TTT | TTHERM_00561770 |
| Lys | TTT | TTHERM_00561780 |
| Lys | TTT | TTHERM_00590230 |
| Met | CAT | TTHERM_00691120 |
| Met | CAT | TTHERM_00691120 |
| Met | CAT | TTHERM_00691120 |
| Met | CAT | TTHERM_00693050 |
| Met | CAT | TTHERM_00694370 |
| Met | CAT | TTHERM_00713370 |
| Met | CAT | TTHERM_00796710 |
| Met | CAT | TTHERM_00823590 |
| Met | CAT | TTHERM_00912300 |
| Met | CAT | TTHERM_00912310 |
| Met | CAT | TTHERM_00912320 |
| Met | CAT | TTHERM_00912330 |
| Met | CAT | TTHERM_00239110 |
| Met | CAT | TTHERM_00245300 |
| Met | CAT | TTHERM_00275950 |
| Met | CAT | TTHERM_01119450 |
| Met | CAT | TTHERM_00284160 |
| Met | CAT | TTHERM_00284170 |
| Met | CAT | TTHERM_01194850 |
| Met | CAT | TTHERM_01194870 |
| Met | CAT | TTHERM_01194880 |
| Met | CAT | TTHERM_01194890 |
| Met | CAT | TTHERM_01194900 |
| Met | CAT | TTHERM_01194910 |
| Met | CAT | TTHERM_01194920 |
| Met | CAT | TTHERM_01194930 |
| Met | CAT | TTHERM_00058990 |
| Met | CAT | TTHERM_00471590 |
| Met | CAT | TTHERM_00471600 |
| Met | CAT | TTHERM_00476700 |
| Met | CAT | TTHERM_00096710 |
| Met | CAT | TTHERM_00096730 |
| Met | CAT | TTHERM_00096750 |
| Met | CAT | TTHERM_00096760 |
| Met | CAT | TTHERM_00096780 |
| Phe | GAA | TTHERM_00013090 |
| Phe | GAA | TTHERM_00013090 |
| Phe | GAA | TTHERM_00715920 |
| Phe | GAA | TTHERM_00715930 |
| Phe | GAA | TTHERM_00728930 |
| Phe | GAA | TTHERM_00151870 |
| Phe | GAA | TTHERM_00823530 |
| Phe | GAA | TTHERM_00264970 |
| Phe | GAA | TTHERM_01211830 |
| Phe | GAA | TTHERM_00414550 |
| Phe | GAA | TTHERM_00415550 |
| Phe | GAA | TTHERM_00415610 |
| Phe | GAA | TTHERM_00433510 |
| Phe | GAA | TTHERM_00440560 |
| Phe | GAA | TTHERM_00441720 |
| Phe | GAA | TTHERM_00471360 |
| Phe | GAA | TTHERM_00527010 |
| Phe | GAA | TTHERM_00527020 |
| Phe | GAA | TTHERM_00527040 |
| Phe | GAA | TTHERM_00527050 |
| Phe | GAA | TTHERM_00527060 |
| Phe | GAA | TTHERM_00527070 |
| Phe | GAA | TTHERM_00105170 |
| Phe | GAA | TTHERM_00105180 |
| Phe | GAA | TTHERM_00641190 |
| Pro | CGG | TTHERM_00474590 |
| Pro | AGG | TTHERM_00744720 |
| Pro | AGG | TTHERM_00745740 |
| Pro | AGG | TTHERM_00745750 |
| Pro | AGG | TTHERM_00746830 |
| Pro | AGG | TTHERM_00170350 |
| Pro | AGG | TTHERM_00170370 |
| Pro | AGG | TTHERM_00170380 |
| Pro | AGG | TTHERM_01031070 |
| Pro | AGG | TTHERM_01031080 |
| Pro | AGG | TTHERM_01031090 |
| Pro | AGG | TTHERM_01031100 |
| Pro | AGG | TTHERM_01031110 |
| Pro | AGG | TTHERM_00299610 |
| Pro | AGG | TTHERM_00299620 |
| Pro | AGG | TTHERM_00299630 |
| Pro | AGG | TTHERM_00299640 |
| Pro | AGG | TTHERM_00299650 |
| Pro | AGG | TTHERM_00299660 |
| Pro | AGG | TTHERM_00299670 |
| Pro | AGG | TTHERM_00299680 |
| Pro | TGG | TTHERM_01207640 |
| Pro | TGG | TTHERM_00316690 |
| Pro | TGG | TTHERM_00316700 |
| Pro | TGG | TTHERM_00316770 |
| Pro | TGG | TTHERM_00316780 |
| Pro | TGG | TTHERM_00316790 |
| Pro | TGG | TTHERM_00316800 |
| Ser | AGA | TTHERM_00145850 |
| Ser | AGA | TTHERM_00145850 |
| Ser | AGA | TTHERM_00145850 |
| Ser | TGA | TTHERM_00697310 |
| Ser | AGA | TTHERM_00145850 |
| Ser | AGA | TTHERM_00161350 |
| Ser | AGA | TTHERM_00161360 |
| Ser | AGA | TTHERM_00185950 |
| Ser | AGA | TTHERM_00185960 |
| Ser | AGA | TTHERM_00185970 |
| Ser | AGA | TTHERM_00185980 |
| Ser | AGA | TTHERM_00185990 |
| Ser | AGA | TTHERM_00186000 |
| Ser | AGA | TTHERM_00821860 |
| Ser | AGA | TTHERM_01031180 |
| Ser | AGA | TTHERM_01031190 |
| Ser | AGA | TTHERM_01031200 |
| Ser | AGA | TTHERM_01031210 |
| Ser | AGA | TTHERM_01031230 |
| Ser | AGA | TTHERM_00279870 |
| Ser | AGA | TTHERM_00494770 |
| Ser | AGA | TTHERM_00118660 |
| Ser | AGA | TTHERM_00118670 |
| Ser | AGA | TTHERM_00599860 |
| Ser | CGA | TTHERM_01298580 |
| Ser | CGA | TTHERM_00549490 |
| Ser | GCT | TTHERM_00242150 |
| Ser | GCT | TTHERM_01084340 |
| Ser | GCT | TTHERM_01132830 |
| Ser | GCT | TTHERM_01132840 |
| Ser | GCT | TTHERM_01132850 |
| Ser | GCT | TTHERM_01223630 |
| Ser | GCT | TTHERM_00399220 |
| Ser | GCT | TTHERM_00494320 |
| Ser | GCT | TTHERM_00494330 |
| Ser | GCT | TTHERM_00494420 |
| Ser | GCT | TTHERM_00494430 |
| Ser | GCT | TTHERM_00494440 |
| Ser | GCT | TTHERM_00494450 |
| Ser | GCT | TTHERM_00494460 |
| Ser | GCT | TTHERM_00494470 |
| Ser | TGA | TTHERM_00697310 |
| Ser | TGA | TTHERM_00697330 |
| Ser | TGA | TTHERM_00697340 |
| Ser | TGA | TTHERM_00697360 |
| Ser | TGA | TTHERM_00704000 |
| Ser | TGA | TTHERM_01068240 |
| Ser | TGA | TTHERM_01068260 |
| Ser | TGA | TTHERM_01068270 |
| Ser | TGA | TTHERM_00118650 |
| Thr | CGT | TTHERM_00851690 |
| Thr | AGT | TTHERM_00151500 |
| Thr | AGT | TTHERM_00151510 |
| Thr | AGT | TTHERM_00756130 |
| Thr | AGT | TTHERM_00756230 |
| Thr | AGT | TTHERM_00756240 |
| Thr | AGT | TTHERM_00756250 |
| Thr | AGT | TTHERM_00756260 |
| Thr | AGT | TTHERM_00756140 |
| Thr | AGT | TTHERM_00756150 |
| Thr | AGT | TTHERM_00756160 |
| Thr | AGT | TTHERM_00756170 |
| Thr | AGT | TTHERM_00756190 |
| Thr | AGT | TTHERM_00756200 |
| Thr | AGT | TTHERM_00756210 |
| Thr | AGT | TTHERM_00756220 |
| Thr | AGT | TTHERM_00066820 |
| Thr | AGT | TTHERM_00419860 |
| Thr | AGT | TTHERM_00419870 |
| Thr | AGT | TTHERM_00419880 |
| Thr | AGT | TTHERM_00419890 |
| Thr | AGT | TTHERM_00419900 |
| Thr | AGT | TTHERM_00561560 |
| Thr | TGT | TTHERM_01124040 |
| Thr | TGT | TTHERM_01124050 |
| Thr | TGT | TTHERM_01124060 |
| Thr | TGT | TTHERM_01124070 |
| Thr | TGT | TTHERM_01124080 |
| Thr | TGT | TTHERM_01124090 |
| Trp | CCA | TTHERM_00316710 |
| Trp | CCA | TTHERM_00316730 |
| Trp | CCA | TTHERM_00316740 |
| Trp | CCA | TTHERM_00316750 |
| Trp | CCA | TTHERM_00316760 |
| Trp | CCA | TTHERM_00448650 |
| Trp | CCA | TTHERM_00449250 |
| Trp | CCA | TTHERM_00449260 |
| Trp | CCA | TTHERM_00449270 |
| Trp | CCA | TTHERM_00449280 |
| Trp | CCA | TTHERM_00449290 |
| Trp | CCA | TTHERM_00530290 |
| Trp | CCA | TTHERM_00530300 |
| Trp | CCA | TTHERM_00530310 |
| Trp | CCA | TTHERM_00530320 |
| Trp | CCA | TTHERM_00530330 |
| Trp | CCA | TTHERM_00530340 |
| Trp | CCA | TTHERM_00648590 |
| Trp | CCA | TTHERM_00648610 |
| Trp | CCA | TTHERM_00648630 |
| Trp | CCA | TTHERM_00648640 |
| Trp | CCA | TTHERM_00648650 |
| Tyr | GTA | TTHERM_00697100 |
| Tyr | GTA | TTHERM_00841320 |
| Tyr | GTA | TTHERM_00035100 |
| Tyr | GTA | TTHERM_00275960 |
| Tyr | GTA | TTHERM_00275970 |
| Tyr | GTA | TTHERM_00275980 |
| Tyr | GTA | TTHERM_00275990 |
| Tyr | GTA | TTHERM_00276000 |
| Tyr | GTA | TTHERM_00276010 |
| Tyr | GTA | TTHERM_00276020 |
| Tyr | GTA | TTHERM_00283060 |
| Tyr | GTA | TTHERM_00292260 |
| Tyr | GTA | TTHERM_01227830 |
| Tyr | GTA | TTHERM_00335900 |
| Tyr | GTA | TTHERM_00335910 |
| Tyr | GTA | TTHERM_00073210 |
| Tyr | GTA | TTHERM_00107190 |
| Tyr | GTA | TTHERM_00550670 |
| Tyr | GTA | TTHERM_00129480 |
| Undet | ??? | TTHERM_00313850 |
| Val | CAC | TTHERM_01014830 |
| Val | TAC | TTHERM_00717560 |
| Val | AAC | TTHERM_00139670 |
| Val | AAC | TTHERM_00713010 |
| Val | AAC | TTHERM_00713020 |
| Val | AAC | TTHERM_00713030 |
| Val | AAC | TTHERM_00713040 |
| Val | AAC | TTHERM_00713050 |
| Val | AAC | TTHERM_00713060 |
| Val | AAC | TTHERM_00713070 |
| Val | AAC | TTHERM_00713080 |
| Val | AAC | TTHERM_00713090 |
| Val | AAC | TTHERM_00151560 |
| Val | AAC | TTHERM_00756100 |
| Val | AAC | TTHERM_01014810 |
| Val | AAC | TTHERM_01014860 |
| Val | AAC | TTHERM_00283940 |
| Val | AAC | TTHERM_00399470 |
| Val | AAC | TTHERM_00402040 |
| Val | AAC | TTHERM_00414260 |
| Val | AAC | TTHERM_00123650 |
| Val | AAC | TTHERM_00123670 |
| Val | AAC | TTHERM_00666690 |
| Val | TAC | TTHERM_00717560 |
| Val | TAC | TTHERM_00717570 |
| Val | TAC | TTHERM_01014820 |
| Val | TAC | TTHERM_01014840 |
| Val | TAC | TTHERM_01014850 |
| Val | TAC | TTHERM_00048960 |
