# Supplementary material for: Macronuclear Genome Sequence of the Ciliate Tetrahymena thermophila, a Model Eukaryote
Source: PLoS Biol. 2006 Aug 29;4(9):e286. doi: 10.1371/journal.pbio.0040286 (PMC1557398; doi:10.1371/journal.pbio.0040286)
Supplement: Supplementary file 14 [file pbio.0040286.st007.doc]

Table S7: Genes predicted to be highly expressed on the basis of codon usage bias

| **Preliminary TIGR ID** | **GenBank Accession** | **Best BLASTP Hit** | **Evalue** | **Number of ESTs** |
| --- | --- | --- | --- | --- |
| 1074 | TTHERM_00670500 | At5g16070 {Arabidopsis thaliana;} ^\|^GB\|AAM91565.1\|22135966\| | 6.00E-173 | 0 |
| 1206 | TTHERM_00853070 | cathepsin L precursor {Metapenaeus ensis;} | 7.50E-63 | 0 |
| 1213 | TTHERM_00853140 |  |  | 0 |
| 1308 | TTHERM_00780960 | ribosomal protein L27 {Branchiostoma belcheri;} | 7.40E-24 | 0 |
| 1369 | TTHERM_00790790 | histone H2A.1 - Tetrahymena thermophila {Tetrahymena thermop | 9.00E-67 | 77 |
| 1646 | TTHERM_01345770 | erythrocyte binding protein 3 {Plasmodium falciparum;} | 2.80E-27 | 0 |
| 1916 | TTHERM_01113100 | gnb2l1-prov protein {Xenopus laevis;} | 9.40E-72 | 0 |
| 2111 | TTHERM_00657270 | Arginyl-tRNA synthetase (EC 6.1.1.19) (Arginine--tRNA ligase | 6.20E-124 | 6 |
| 2230 | TTHERM_00473300 | putative RNaseIII {Dictyostelium discoideum;} | 5.60E-14 | 1 |
| 2296 | TTHERM_00474970 | 60S acidic ribosomal protein P2. {Podospora anserina;} ^\|^GB | 1.20E-15 | 29 |
| 2313 | TTHERM_00475140 | Protein disulfide isomerase precursor (EC 5.3.4.1) (PDI) (Pr | 2.30E-94 | 19 |
| 2578 | TTHERM_01308050 | Fumarate hydratase, mitochondrial precursor (EC 4.2.1.2) (Fu | 4.10E-174 | 2 |
| 2919 | TTHERM_00694350 | Igr3p {Tetrahymena thermophila;} | 2.70E-74 | 0 |
| 3159 | TTHERM_00535740 | PfMPC {Plasmodium falciparum;} ^\|^GB\|AAN36111.1\|23496553\|AE0 | 1.00E-81 | 229 |
| 3280 | TTHERM_00762890 | 40S ribosomal protein S17 {Lycopersicon esculentum;} | 2.80E-40 | 6 |
| 3322 | TTHERM_00765300 | ribosomal protein S14 {Mus musculus;} | 2.00E-55 | 7 |
| 3393 | TTHERM_00312040 |  |  | 0 |
| 3599 | TTHERM_01085640 | lysozyme murein hydrolase {Aeromonas phage Aeh1;Bacteriophag | 3.00E-11 | 3 |
| 3650 | TTHERM_00655520 | Sod (superoxide dismutase) protein 1 {Caenorhabditis elegans | 8.30E-41 | 2 |
| 3676 | TTHERM_00655820 | Elongation factor 1-alpha (EF-1-alpha) (14-nm filament-assoc | 6.50E-231 | 31 |
| 3732 | TTHERM_00938820 | elongation factor 2 {Tetrahymena thermophila;} | 0 | 0 |
| 3764 | TTHERM_00939150 |  |  | 0 |
| 3815 | TTHERM_00444470 | S-adenosyl methionine synthetase {Phytophthora infestans;} | 1.60E-131 | 38 |
| 3819 | TTHERM_00444510 | putative ribosomal protein L19 {Oryza sativa (japonica culti | 1.90E-56 | 32 |
| 4221 | TTHERM_00492850 | Isoleucyl-tRNA synthetase (EC 6.1.1.5) (Isoleucine--tRNA lig | 0 | 4 |
| 4424 | TTHERM_00773330 | 40s ribosomal protein s13 - fission yeast (Schizosaccharomyc | 6.50E-41 | 0 |
| 4449 | TTHERM_00773580 | Cytochrome c. {Tetrahymena pyriformis;} ^\|^PIR\|A00071\|CCTE c | 3.40E-51 | 19 |
| 4605 | TTHERM_01014750 | dnaK protein [imported] - Brucella melitensis (strain 16M) { | 1.40E-187 | 0 |
| 4648 | TTHERM_00529790 | Citrate synthase, mitochondrial precursor (EC 2.3.3.1) (14 N | 2.50E-251 | 19 |
| 4655 | TTHERM_00529860 | vacuolar ATPase {Paramecium multimicronucleatum;} | 7.20E-216 | 5 |
| 4857 | TTHERM_00283180 | histone H2B.2 - Tetrahymena thermophila {Tetrahymena thermop | 1.50E-57 | 4 |
| 5157 | TTHERM_00621600 | Macronuclear solute carrier homolog CR-MSC. {Sterkiella hist | 2.30E-29 | 140 |
| 5263 | TTHERM_00348510 | Tubulin beta chain. {Tetrahymena thermophila;} ^\|^PIR\|S42519 | 1.10E-242 | 19 |
| 5344 | TTHERM_00353280 | Adenosylhomocysteinase (EC 3.3.1.1) (S-adenosyl-L-homocystei | 1.10E-169 | 7 |
| 5620 | TTHERM_00442200 |  |  | 4 |
| 5965 | TTHERM_00333210 | putative 60S ribosomal protein {Arabidopsis thaliana;} | 9.80E-92 | 39 |
| 6064 | TTHERM_00338230 | cytoplasmic ribosomal protein L18 {Oryza sativa;} | 2.80E-40 | 11 |
| 6073 | TTHERM_00338320 |  |  | 0 |
| 6103 | TTHERM_00339620 | ubiquitin / ribosomal protein CEP52 - Tetrahymena pyriformis | 1.70E-65 | 0 |
| 6193 | TTHERM_00476640 | 60S ribosomal protein L24 {Pagrus major;} | 3.80E-20 | 105 |
| 6263 | TTHERM_00481310 | CG15740-PA {Drosophila melanogaster;} | 3.10E-06 | 5 |
| 6450 | TTHERM_00527180 | granule lattice protein 1 precursor {Tetrahymena thermophila | 4.70E-212 | 15 |
| 6657 | TTHERM_00193740 | 40S ribosomal protein S2 (Strings of pearls protein). {Droso | 2.90E-70 | 64 |
| 6821 | TTHERM_00196360 | hsp60 {Paramecium caudatum;} | 3.40E-74 | 0 |
| 6822 | TTHERM_00196370 | hsp60 {Paramecium caudatum;} | 1.20E-137 | 0 |
| 6860 | TTHERM_00495950 | 23 kDa calcium-binding protein (TCBP-23) [Contains: 12 kDa c | 4.40E-106 | 1 |
| 6953 | TTHERM_00497880 | ribosomal protein L3 {Tetrahymena thermophila;} | 5.20E-213 | 53 |
| 6961 | TTHERM_00497960 | T-complex protein 1, eta subunit (TCP-1-eta) (CCT-eta). {Tet | 1.60E-273 | 27 |
| 6984 | TTHERM_00189170 | histone H4 - Tetrahymena thermophila {Tetrahymena thermophil | 2.80E-49 | 108 |
| 7044 | TTHERM_00918490 | Cytochrome c1, heme protein, mitochondrial precursor. {Neuro | 7.30E-06 | 0 |
| 7128 | TTHERM_00420030 | similar to Emericella nidulans. Phosphoenolpyruvate carboxyk | 4.20E-168 | 10 |
| 7171 | TTHERM_00420460 |  |  | 1 |
| 7230 | TTHERM_00421060 |  |  | 5 |
| 7520 | TTHERM_01194830 | natural killer enhancing factor {Cyprinus carpio;} ^\|^GB\|BAA | 1.20E-62 | 0 |
| 7902 | TTHERM_00558040 | XNop56 protein {Xenopus laevis;} | 3.30E-117 | 18 |
| 7958 | TTHERM_00558620 | Tubulin alpha chain. {Tetrahymena thermophila;} ^\|^GB\|AAA213 | 2.00E-243 | 112 |
| 8077 | TTHERM_00502380 | putative mitochondrial processing peptidase {Arabidopsis tha | 3.10E-57 | 0 |
| 8111 | TTHERM_00502720 |  |  | 3 |
| 8415 | TTHERM_00491100 |  |  | 0 |
| 8418 | TTHERM_00491130 | cell surface immobilization antigen SerH5 {Tetrahymena therm | 9.00E-09 | 0 |
| 8520 | TTHERM_00786930 | nucleosome assembly protein 1-like protein 3 {Nicotiana taba | 4.00E-44 | 11 |
| 8668 | TTHERM_00189180 | histone H3 - Tetrahymena thermophila {Tetrahymena thermophil | 1.30E-65 | 18 |
| 8746 | TTHERM_00190950 | actin {Tetrahymena thermophila;} | 1.10E-203 | 14 |
| 8830 | TTHERM_00191800 | Aconitate hydratase, cytoplasmic (EC 4.2.1.3) (Citrate hydro | 1.20E-300 | 6 |
| 9088 | TTHERM_00859260 | translation elongation factor eEF-1 beta chain - silkworm {B | 6.60E-32 | 129 |
| 9179 | TTHERM_00648580 |  |  | 0 |
| 9396 | TTHERM_00128320 | Cathepsin D precursor (EC 3.4.23.5). {Clupea harengus;} ^\|^G | 1.90E-82 | 6 |
| 9701 | TTHERM_00877050 | peptidylprolyl isomerase (EC 5.2.1.8) CYP5 [similarity] - Ar | 3.00E-59 | 12 |
| 9840 | TTHERM_00268050 | phospholipase A1 {Tetrahymena thermophila;} | 1.10E-77 | 0 |
| 9841 | TTHERM_00268060 | phospholipase A1 {Tetrahymena thermophila;} | 2.10E-76 | 13 |
| 10070 | TTHERM_00802330 | polyubiquitin 5 - Tetrahymena thermophila {Tetrahymena therm | 9.80E-196 | 1 |
| 10091 | TTHERM_00077450 | hypothetical protein, conserved {Plasmodium falciparum 3D7;} | 1.00E-26 | 29 |
| 10151 | TTHERM_00079050 | similar to Dictyostelium discoideum (Slime mold). Prespore-s | 1.70E-11 | 0 |
| 10191 | TTHERM_00079450 | phospholipase A1 {Tetrahymena thermophila;} | 4.50E-81 | 0 |
| 10317 | TTHERM_00378630 |  |  | 0 |
| 10343 | TTHERM_00378890 | granule lattice protein 5 precursor {Tetrahymena thermophila | 3.50E-191 | 16 |
| 10354 | TTHERM_00379000 | epiplasmin C {Tetrahymena pyriformis;} | 1.40E-221 | 8 |
| 10389 | TTHERM_00382320 |  |  | 0 |
| 10390 | TTHERM_00382330 |  |  | 0 |
| 10516 | TTHERM_00571860 | ATP synthase alpha chain heart isoform, mitochondrial precur | 1.30E-190 | 55 |
| 10584 | TTHERM_00641150 | cathepsin B {Bos taurus;} ^\|^GB\|AAA03064.1\|289402\|BOVCATHB c | 1.90E-89 | 13 |
| 10776 | TTHERM_00519810 | MGC52653 protein {Xenopus laevis;} | 1.30E-159 | 1 |
| 10983 | TTHERM_00673440 | hypothetical protein {Lactobacillus johnsonii NCC 533;} | 1.10E-08 | 0 |
| 11127 | TTHERM_00046480 | enolase {Oryza sativa (japonica cultivar-group);} | 1.70E-164 | 121 |
| 11227 | TTHERM_00047480 | 40S ribosomal protein S3A, putative {Plasmodium falciparum 3 | 7.00E-44 | 83 |
| 11339 | TTHERM_00050590 | hypothetical protein MGC75641 {Silurana tropicalis;Xenopus t | 1.70E-120 | 3 |
| 11411 | TTHERM_00052310 | PET9 {Saccharomyces kluyveri;} | 1.60E-106 | 0 |
| 11924 | TTHERM_00016170 | histone H3.3 - Tetrahymena thermophila {Tetrahymena thermoph | 1.00E-65 | 40 |
| 11927 | TTHERM_00016200 | histone H3.3 - Tetrahymena thermophila {Tetrahymena thermoph | 3.10E-64 | 0 |
| 11993 | TTHERM_00022810 | Phosphoenolpyruvate phosphomutase precursor (EC 5.4.2.9)(Pho | 2.30E-148 | 30 |
| 12011 | TTHERM_00023980 | GTP-binding nuclear protein RAN/TC4. {Tetrahymena thermophil | 4.10E-114 | 0 |
| 12378 | TTHERM_00522630 | 40S ribosomal protein S0 (Extracellular matrix receptor prot | 1.50E-41 | 0 |
| 12379 | TTHERM_00522640 | putative 40S ribosomal protein {Oryza sativa (japonica culti | 4.50E-12 | 0 |
| 12437 | TTHERM_00222300 | ribonuclease, RNS1 {Arabidopsis thaliana;} | 9.80E-22 | 0 |
| 12514 | TTHERM_00227040 | FKBP12 {Tetrahymena thermophila;} | 1.20E-55 | 0 |
| 12537 | TTHERM_00227270 | ribosomal protein L22 {Spodoptera frugiperda;} | 8.90E-28 | 32 |
| 12587 | TTHERM_00227770 | putative translation initiation factor 5A2 {Toxoplasma gondi | 4.00E-48 | 131 |
| 12756 | TTHERM_00402120 | Elongation factor 1-gamma (EF-1-gamma) (eEF-1B gamma). {Oryz | 3.60E-47 | 8 |
| 12821 | TTHERM_01339580 |  |  | 0 |
| 12822 | TTHERM_01339590 |  |  | 0 |
| 12854 | TTHERM_00653670 |  |  | 2 |
| 12887 | TTHERM_00654000 |  |  | 0 |
| 13044 | TTHERM_00058590 | fructose-1,6-bisphosphatase {Porteresia coarctata;} | 1.80E-102 | 1 |
| 13179 | TTHERM_00066890 | Vacuolar processing enzyme precursor (EC 3.4.22.-) (VPE) (Pr | 1.20E-66 | 5 |
| 13452 | TTHERM_00365340 | transitional endoplasmic reticulum ATPase - Arabidopsis thal | 1.40E-301 | 2 |
| 13744 | TTHERM_00201730 |  |  | 0 |
| 13911 | TTHERM_00770690 | 10 kDa chaperonin (Protein CPN10) (Protein groES). {Arabidop | 2.90E-22 | 2 |
| 14061 | TTHERM_00426260 | Histidine-rich glycoprotein precursor. {Plasmodium lophurae; | 4.00E-22 | 2 |
| 14117 | TTHERM_00829380 | 60S ribosomal protein L11 (L21). {Tetrahymena thermophila;} | 2.20E-73 | 0 |
| 14204 | TTHERM_00028740 | 60S ribosomal protein L12. {Caenorhabditis briggsae;Caenorha | 5.60E-35 | 0 |
| 14250 | TTHERM_00030190 | malate dehydrogenase {Xanthomonas axonopodis pv. citri str. | 1.80E-102 | 14 |
| 14392 | TTHERM_00035580 | putative integral membrane protein {Phaseolus vulgaris;} | 5.10E-112 | 0 |
| 14441 | TTHERM_00037060 | chaperonin-containing-TCP1 theta subunit {Tetrahymena pyrifo | 6.50E-254 | 2 |
| 14482 | TTHERM_00037470 | Aconitate hydratase, cytoplasmic (EC 4.2.1.3) (Citrate hydro | 5.1e-313 | 1 |
| 14753 | TTHERM_01188360 |  |  | 0 |
| 14804 | TTHERM_01256490 | phospholipase A1 {Tetrahymena thermophila;} | 2.60E-76 | 26 |
| 15252 | TTHERM_00926980 | similar to acetyl-coa acetyltransferase (EC 2.3.1.9) [Schizo | 2.20E-104 | 11 |
| 15314 | TTHERM_01055600 | Ndc1 protein {Tetrahymena thermophila;} | 4.00E-206 | 40 |
| 15525 | TTHERM_00454080 | ribosomal protein S15a, cytosolic - fruit fly (Drosophila me | 4.00E-48 | 0 |
| 15725 | TTHERM_00297140 | 60S ribosomal protein L23. {Caenorhabditis elegans;} ^\|^PIR\| | 3.00E-52 | 63 |
| 15853 | TTHERM_00647470 | cathepsin E protein {Mus musculus;} | 1.20E-64 | 0 |
| 16138 | TTHERM_00683010 | phospholipase A1 {Tetrahymena thermophila;} | 3.40E-170 | 24 |
| 16143 | TTHERM_00683060 | phospholipase A1 {Tetrahymena thermophila;} | 2.10E-97 | 6 |
| 16366 | TTHERM_00085180 | ribosomal protein L32, cytosolic [validated] - rat {Rattus n | 3.70E-38 | 21 |
| 16419 | TTHERM_00086700 | MORN repeat protein {uncultured bacterium 314;} | 1.80E-46 | 0 |
| 16846 | TTHERM_00711860 |  |  | 5 |
| 16866 | TTHERM_00713140 | histamine-releasing factor, putative {Plasmodium falciparum | 5.30E-30 | 5 |
| 17371 | TTHERM_00929590 | similar to nucleolar protein family A, member 1 (H/ACA small | 1.30E-41 | 129 |
| 17540 | TTHERM_00697480 | OSJNBb0070J16.13 {Oryza sativa (japonica cultivar-group);} ^ | 7.00E-12 | 0 |
| 17572 | TTHERM_01299660 | unknown protein {Arabidopsis thaliana;} ^\|^GB\|AAM65457.1\|215 | 1.20E-18 | 18 |
| 17678 | TTHERM_01049200 | glutamate dehydrogenase {Paramecium tetraurelia;} | 1.50E-159 | 0 |
| 18192 | TTHERM_00684790 | Hypothetical protein F27C1.7b {Caenorhabditis elegans;} | 3.30E-21 | 0 |
| 18229 | TTHERM_00686150 | ubiquitin family protein; identical to S. pombe SPAC6G10.11c | 4.90E-27 | 243 |
| 18338 | TTHERM_00992760 | ribosomal protein S20 {Spodoptera frugiperda;} | 4.80E-29 | 0 |
| 18423 | TTHERM_00721150 | 60S ribosomal protein L7-3. {Arabidopsis thaliana;} ^\|^GB\|AA | 1.10E-57 | 0 |
| 18564 | TTHERM_00760790 | hypothetical protein F10M23.360 - Arabidopsis thaliana {Arab | 1.30E-58 | 1 |
| 18668 | TTHERM_00467660 | 40S ribosomal protein S24. {Spodoptera frugiperda;} ^\|^GB\|AA | 3.60E-24 | 0 |
| 19096 | TTHERM_00716240 | hypothetical protein B0250.1 - Caenorhabditis elegans {Caeno | 3.20E-78 | 0 |
| 19176 | TTHERM_00616270 | ubiquinone/menaquinone biosynthesis methyltransferase {Pyroc | 5.70E-10 | 8 |
| 19600 | TTHERM_00486690 |  |  | 1 |
| 19640 | TTHERM_00487090 | fructose-bisphosphate aldolase (EC 4.1.2.13), cytosolic - co | 7.10E-106 | 146 |
| 19662 | TTHERM_00947420 | tropomyosin isoform TMII - Caenorhabditis elegans {Caenorhab | 4.50E-06 | 0 |
| 19700 | TTHERM_00636970 | acidic ribosomal phosphoprotein P0 {Eufolliculina uhligi;} | 5.50E-44 | 0 |
| 20062 | TTHERM_01141570 | Isocitrate lyase (EC 4.1.3.1) (Isocitrase) (Isocitratase) (I | 1.90E-133 | 0 |
| 20190 | TTHERM_01151500 | putative 40S ribosomal protein S3 {Triticum aestivum;} | 1.40E-59 | 7 |
| 20220 | TTHERM_01289110 | 60S ribosomal protein L15-1. {Picea mariana;} ^\|^GB\|AAC32112 | 2.00E-62 | 0 |
| 20309 | TTHERM_00456790 |  |  | 10 |
| 20436 | TTHERM_00823660 |  |  | 6 |
| 20437 | TTHERM_00823670 | 40S ribosomal protein S19S. {Ascaris suum;} ^\|^PIR\|A54581\|A5 | 9.50E-24 | 29 |
| 20442 | TTHERM_00823720 | histone H1, macronuclear - Tetrahymena thermophila {Tetrahym | 1.70E-81 | 39 |
| 20533 | TTHERM_00898280 | Calcium/calmodulin-dependent protein kinase type II delta ch | 1.90E-34 | 0 |
| 20552 | TTHERM_00899460 | 3-ketoacyl-CoA thiolase, peroxisomal precursor (EC 2.3.1.16) | 7.50E-115 | 84 |
| 20689 | TTHERM_00149300 | ribosomal protein S4 {Spodoptera frugiperda;} | 1.20E-76 | 0 |
| 20961 | TTHERM_00316500 | histone H2A.2 - Tetrahymena thermophila {Tetrahymena thermop | 1.20E-64 | 16 |
| 21044 | TTHERM_00317440 | 17-beta-hydroxysteroid dehydrogenase type 4 {Danio rerio;} | 9.10E-106 | 59 |
| 21139 | TTHERM_00777250 |  |  | 8 |
| 21358 | TTHERM_00310540 |  |  | 2 |
| 21410 | TTHERM_00794110 | Isocitrate dehydrogenase [NADP], mitochondrial precursor (EC | 1.90E-152 | 9 |
| 21586 | TTHERM_00411680 | CG15040-PA {Drosophila melanogaster;} | 6.10E-10 | 3 |
| 22023 | TTHERM_01053000 | 60S ribosomal protein L10 (Wilm's tumor suppressor protein h | 3.40E-74 | 494 |
| 22254 | TTHERM_00218270 |  |  | 2 |
| 22306 | TTHERM_00218790 | inorganic pyrophosphatase {Nicotiana tabacum;} | 1.80E-191 | 13 |
| 22853 | TTHERM_00095540 | Dyskerin (Nucleolar protein NAP57). {Rattus norvegicus;} ^\|^ | 3.20E-151 | 3 |
| 22928 | TTHERM_00101290 | cytochrome P450 CYP4AB2 {Solenopsis invicta;} | 5.20E-39 | 5 |
| 23013 | TTHERM_00105110 | heat shock protein {Euplotes eurystomus;} | 8.80E-258 | 19 |
| 23654 | TTHERM_01197120 | eukaryotic translation initiation factor 4A {Toxoplasma gond | 3.40E-123 | 0 |
| 23762 | TTHERM_00660180 | High-mobility-group protein B. {Tetrahymena thermophila;} ^\| | 5.20E-71 | 15 |
| 23890 | TTHERM_00666370 |  |  | 4 |
| 23907 | TTHERM_00666540 | ribosomal protein L21 [imported] - rice {Oryza sativa;} ^\|^G | 9.30E-33 | 109 |
| 23917 | TTHERM_00666640 | 3-hydroxyacyl-CoA dehydrogenase {Bacillus anthracis str. Ame | 2.70E-81 | 4 |
| 24232 | TTHERM_00131110 | ribosomal protein S18 Y57G11C.16 [similarity] - Caenorhabdit | 3.00E-43 | 74 |
| 24318 | TTHERM_00134940 | ribosomal protein L23a {Argopecten irradians;} | 9.90E-29 | 36 |
| 24337 | TTHERM_00136120 | 60S ribosomal protein L6 (YL16-like). {Mesembryanthemum crys | 7.90E-36 | 1 |
| 24687 | TTHERM_00628610 | Thiol protease SEN102 precursor (EC 3.4.22.-). {Hemerocallis | 1.10E-56 | 8 |
| 24772 | TTHERM_00384800 | nucleolar phosphoprotein {Tetrahymena thermophila;} | 6.70E-245 | 8 |
| 24783 | TTHERM_00384910 | centrin {Tetrahymena thermophila;} | 4.30E-83 | 1 |
| 25039 | TTHERM_00592860 |  |  | 0 |
| 25046 | TTHERM_00592930 |  |  | 2 |
| 25049 | TTHERM_00592960 |  |  | 0 |
| 25431 | TTHERM_00734080 | putative equilibrative nucleoside transporter ENT8 {Arabidop | 4.20E-15 | 0 |
| 25448 | TTHERM_00735240 | ADP-ribosylation factor 1 - potato {Solanum tuberosum;} ^\|^G | 1.20E-66 | 61 |
| 25801 | TTHERM_00463490 | 40S ribosomal protein S26. {Sterkiella nova;} ^\|^GB\|CAC34796 | 2.60E-30 | 4 |
| 25891 | TTHERM_00285620 | nascent polypeptide associated complex alpha chain {Oryza sa | 6.20E-20 | 30 |
| 26020 | TTHERM_00290880 | Ubiquitin-conjugating enzyme E2-17 kDa 1 (EC 6.3.2.19) (Ubiq | 3.60E-24 | 9 |
| 26124 | TTHERM_00343440 | putative RNA-binding protein {Oryza sativa (japonica cultiva | 8.90E-22 | 2 |
| 26265 | TTHERM_00346900 | carboxypeptidase-like protein F14D17.80 [imported] - Arabido | 1.60E-78 | 0 |
| 26494 | TTHERM_00433520 | copper-induced metallothionein {Tetrahymena thermophila;} ^\| | 5.50E-67 | 3 |
| 26495 | TTHERM_00433530 | copper-induced metallothionein {Tetrahymena thermophila;} ^\| | 9.00E-67 | 2 |
| 26524 | TTHERM_00433830 |  |  | 1 |
| 26532 | TTHERM_00433910 | bactericidal permeability increasing protein/lipopolysacchar | 2.60E-07 | 0 |
| 26535 | TTHERM_00433940 | Bactericidal/permeability-increasing protein-like 2 precurso | 2.30E-08 | 1 |
| 26542 | TTHERM_00434010 |  |  | 0 |
| 26545 | TTHERM_00434040 | 40S ribosomal protein S12. {Tetrahymena thermophila;} ^\|^PIR | 8.90E-60 | 0 |
| 26548 | TTHERM_00434070 | 40S ribosomal subunit protein S9, putative {Plasmodium falci | 7.60E-54 | 0 |
| 26861 | TTHERM_00705060 |  |  | 0 |
| 26867 | TTHERM_00705120 |  |  | 0 |
| 26886 | TTHERM_00706300 | RPS16 {Saccharomyces kluyveri;} | 4.80E-29 | 0 |
| 27021 | TTHERM_00943010 | 60S ribosomal subunit protein L31, putative {Plasmodium falc | 2.70E-26 | 23 |
| 27151 | TTHERM_00114360 | Seryl-tRNA synthetase, cytoplasmic (EC 6.1.1.11) (Serine--tR | 3.50E-119 | 22 |
| 27180 | TTHERM_00118610 | Hypothetical protein Y69A2AR.18a {Caenorhabditis elegans;} | 4.40E-42 | 2 |
| 27610 | TTHERM_01004930 | SI:dZ150I12.2 (novel protein similar to human proliferation- | 7.10E-79 | 0 |
| 27639 | TTHERM_01005220 | glucosamine--fructose-6-phosphate aminotransferase, putative | 2.00E-110 | 0 |
| 27643 | TTHERM_01005260 | beta-hydroxybutyrate dehydrogenase {Azospirillum brasilense; | 6.10E-77 | 5 |
| 27769 | TTHERM_01018520 | granule lattice protein 5 precursor {Tetrahymena thermophila | 1.50E-137 | 0 |
| 27771 | TTHERM_01018540 | granule lattice protein 5 precursor {Tetrahymena thermophila | 4.10E-165 | 1 |
| 27859 | TTHERM_00559980 |  |  | 6 |
| 28005 | TTHERM_00633360 | histone H2B.1 - Tetrahymena thermophila {Tetrahymena thermop | 1.90E-57 | 4 |
| 28119 | TTHERM_01386050 | 40S ribosome protein S5 {Griffithsia japonica;} | 3.30E-60 | 41 |
| 28303 | TTHERM_01276400 | cysteine proteinase homolog {Naegleria fowleri;} | 1.40E-70 | 3 |
| 28394 | TTHERM_00158040 | ran binding protein 1 {Plasmodium falciparum 3D7;} ^\|^GB\|CAD | 2.50E-29 | 2 |
| 28442 | TTHERM_00158520 | hsp82 heat shock protein {Tetrahymena thermophila;} | 0 | 0 |
| 28493 | TTHERM_00161010 | nucleoside diphosphate kinase {Aspergillus fumigatus;} | 9.50E-56 | 27 |
| 28498 | TTHERM_00161060 | 40S ribosomal protein S12. {Hordeum vulgare;} ^\|^GB\|AAD39838 | 5.00E-18 | 29 |
| 28505 | TTHERM_00161130 | cathepsin L {Stylonychia lemnae;} | 4.00E-64 | 17 |
| 29011 | TTHERM_00577240 | putative 60S ribosomal protein L10A (RPL10aC) {Oryza sativa | 4.40E-58 | 126 |
| 29117 | TTHERM_00864870 | hypothetical protein APE2147 - Aeropyrum pernix (strain K1) | 7.80E-26 | 12 |
| 29178 | TTHERM_00550700 | unknown protein {Arabidopsis thaliana;} ^\|^GB\|AAK59549.1\|143 | 2.00E-96 | 0 |
| 29220 | TTHERM_00551160 | glyceraldehyde-3-phosphate dehydrogenase {Tetrahymena thermo | 2.00E-159 | 18 |
| 29794 | TTHERM_00171850 | ER-type hsp70 {Paramecium caudatum;} | 1.30E-232 | 4 |
| 29845 | TTHERM_00185230 | Triosephosphate isomerase (EC 5.3.1.1) (TIM). {Schistosoma m | 5.30E-62 | 126 |
| 30010 | TTHERM_00813040 | Unknown (protein for MGC:66194) {Danio rerio;} | 3.10E-49 | 0 |
| 30035 | TTHERM_01128560 | ribosomal protein S10 {Crassostrea gigas;} | 1.80E-15 | 0 |
| 30046 | TTHERM_01129660 | hypothetical protein F37C12.4 - Caenorhabditis elegans {Caen | 2.40E-18 | 50 |
| 30119 | TTHERM_01146030 | putative catalase {Tetrahymena thermophila;} | 4.40E-265 | 6 |
| 30285 | TTHERM_00329800 | guanylate kinase {Aspergillus fumigatus;} | 1.40E-09 | 9 |
