# Supplementary material for: Macronuclear Genome Sequence of the Ciliate Tetrahymena thermophila, a Model Eukaryote
Source: PLoS Biol. 2006 Aug 29;4(9):e286. doi: 10.1371/journal.pbio.0040286 (PMC1557398; doi:10.1371/journal.pbio.0040286)
Supplement: Supplementary file 15 [file pbio.0040286.st008.doc]

Table S8: Likely mitochondrion-derived genes from the *T. thermophila* macronuclear genome, identified as being related to those from alpha proteobacteria or mitochondria.

| **Gene ID** | **GenBank Accession** | **Best Genbank hit** |
| --- | --- | --- |
| 1066 | TTHERM_00670420 | conserved hypothetical protein {Wolbachia endosymbiont of Drosophila melanogaster} |
| 1072 | TTHERM_00670480 | Mitochondrial processing peptidase beta subunit, mitochondrialprecursor {Neurospora crassa} |
| 2578 | TTHERM_01308050 | Fumarate hydratase, mitochondrial precursor {Rattus norvegicus} |
| 3333 | TTHERM_00766400 | proteinase I {Brucella melitensis} |
| 3389 | TTHERM_00312000 | unknown {Phytophthora infestans} |
| 4132 | TTHERM_00585260 | ATP synthase beta chain, mitochondrial precursor {Rattus norvegicus} |
| 4449 | TTHERM_00773580 | Cytochrome c {Tetrahymena pyriformis} |
| 4597 | TTHERM_01014670 | 5-aminolevulinic acid synthase {Rickettsia prowazekii} |
| 4694 | TTHERM_00530250 | Aldh3a2 protein {Mus musculus} |
| 4738 | TTHERM_00530750 | dihydrolipoamide S-acetyltransferase (E2 component of pyruvate dehydrogenase complex) {Mus musculus} |
| 4833 | TTHERM_00279940 | Pseudouridine synthase {Rhodopseudomonas palustris } |
| 5302 | TTHERM_00348900 | putative DNA alkylation repair enzyme {Bdellovibrio bacteriovorus} |
| 5438 | TTHERM_00438810 | Hypothetical 46.7 kDa protein in HOR7-COX7 intergenic region. {Saccharomyces cerevisiae} |
| 5593 | TTHERM_00441930 | selenium binding protein 1 {Mus musculus} |
| 5778 | TTHERM_00578850 | AT19154p {Drosophila melanogaster} |
| 6008 | TTHERM_00335630 | subunit NUHM of protein NADH:Ubiquinone Oxidoreductase (Complex I) {Yarrowia lipolytica} |
| 6123 | TTHERM_00339820 | methylenetetrahydrofolate dehydrogenase {Aquifex aeolicus} |
| 6286 | TTHERM_00483520 | hypothetical protein {Agrobacterium tumefaciens} |
| 6606 | TTHERM_00193230 | Pyruvate dehydrogenase E1 component alpha subunit, mitochondrialprecursor {Solanum tuberosum} |
| 6675 | TTHERM_00193910 | putative NADH dehydrogenase {Oryza sativa} |
| 6710 | TTHERM_00194260 | NADH dehydrogenase (ubiquinone) Fe-S protein 1 {Mus musculus} |
| 6941 | TTHERM_00497760 | unknown {Phytophthora infestans} |
| 7045 | TTHERM_00918500 | AT13706p {Drosophila melanogaster} |
| 7192 | TTHERM_00420670 | Tetratricopeptide repeat family protein {Fusobacterium nucleatum} |
| 7582 | TTHERM_00678180 | conserved CBS domain multi-pass transmembrane protein {Cryptosporidium parvum} |
| 7889 | TTHERM_00557910 | hypothetical protein {Caenorhabditis elegans} |
| 8277 | TTHERM_00979780 | Pyruvate dehydrogenase E1 component beta subunit, mitochondrialprecursor {Arabidopsis thaliana} |
| 8552 | TTHERM_00787250 | glutamate dehydrogenase {Paramecium tetraurelia} |
| 10314 | TTHERM_00378600 | Testis-specific gene A2 (Male meiotic metaphase chromosome-associatedacidic protein) (Meichroacidin). {Homo sapiens} |
| 10478 | TTHERM_00569500 | Brefeldin A-inhibited guanine nucleotide-exchange protein 2 {Homo sapiens} |
| 10516 | TTHERM_00571860 | ATP synthase alpha chain heart isoform, mitochondrial precursor {Bos taurus} |
| 11064 | TTHERM_00043870 | dihydrolipoamide dehydrogenase precursor {Lycopersicon esculentum} |
| 11216 | TTHERM_00047370 | succinate-semialdehyde dehydrogenase (NADPH+) (ssdH) {Agrobacterium tumefaciens} |
| 11414 | TTHERM_00052340 | hypothetical protein {Arabidopsis thaliana} |
| 11537 | TTHERM_00001300 | hypothetical protein {Schizosaccharomyces pombe} |
| 11971 | TTHERM_00019620 | TPR domain/sulfotransferase domain protein {uncultured bacterium 560} |
| 12855 | TTHERM_00653680 | AT17730p {Drosophila melanogaster} |
| 13452 | TTHERM_00365340 | transitional endoplasmic reticulum ATPase {Arabidopsis thaliana} |
| 13530 | TTHERM_00415580 | ATP-binding protein of ABC transporter {Nostoc sp. PCC 7120} |
| 13690 | TTHERM_00198220 | p450 enzyme precursor {Rhodnius prolixus} |
| 14352 | TTHERM_00035180 | Probable acetoacetate decarboxylase 1 {Bradyrhizobium japonicum} |
| 14517 | TTHERM_00038820 | DKFZP586B1621 protein {Homo sapiens} |
| 14996 | TTHERM_00541500 | ABC transporter, ATP-binding protein {Chlorobium tepidum TLS} |
| 15268 | TTHERM_00927150 | hypothetical protein, conserved {Plasmodium falciparum 3D7} |
| 15270 | TTHERM_00927170 | hypothetical protein, conserved {Plasmodium falciparum 3D7} |
| 15574 | TTHERM_00294640 | NADH-ubiquinone oxidoreductase 23 kDa subunit, mitochondrial precursor {Arabidopsis thaliana} |
| 15618 | TTHERM_00295080 | AT5g13430/T22N19_80 {Arabidopsis thaliana} |
| 16425 | TTHERM_00086760 | heat shock protein GrpE {Brucella suis 1330} |
| 17666 | TTHERM_01048080 | patatin b1 precursor {Rickettsia conorii} |
| 17669 | TTHERM_01048110 | glutamate dehydrogenase {Paramecium tetraurelia} |
| 17678 | TTHERM_01049200 | glutamate dehydrogenase {Paramecium tetraurelia} |
| 17781 | TTHERM_00836690 | Mitochondrial processing peptidase alpha subunit, mitochondrialprecursor {Solanum tuberosum} |
| 18455 | TTHERM_00721470 | unnamed protein product {Arabidopsis thaliana} |
| 18956 | TTHERM_00242190 | GTP-binding protein engA. {Brucella melitensis} |
| 19077 | TTHERM_00716050 | coproporphyrinogen oxidase {Homo sapiens} |
| 19744 | TTHERM_00637410 | cytochrome c oxidase assembly protein COX11 {Homo sapiens} |
| 19960 | TTHERM_00534030 | heat shock protein HslV {Wolbachia endosymbiont of Drosophila melanogaster} |
| 20301 | TTHERM_00456710 | 23S ribsomal RNA methyltransferase {Brucella melitensis} |
| 20825 | TTHERM_00151670 | probable Zinc-binding dehydrogenases {Leptospira interrogans serovar Lai str. 56601} |
| 21101 | TTHERM_00775880 | immobilization antigen LD {Tetrahymena thermophila} |
| 21804 | TTHERM_01135020 | H1 immobilization antigen {Tetrahymena thermophila} |
| 21960 | TTHERM_00895780 | SEC7-related protein {Paramecium tetraurelia} |
| 22100 | TTHERM_00600370 | hypothetical protein {Mesorhizobium loti} |
| 22464 | TTHERM_00470500 | cytohesin-like protein, possible {Cryptosporidium parvum} |
| 22682 | TTHERM_01034460 | 50S ribosomal protein L20 {Bradyrhizobium japonicum} |
| 23179 | TTHERM_00689830 | probable thiophene and furan oxidation protein thdF {Agrobacterium tumefaciens} |
| 23805 | TTHERM_00661600 | WD-domain containing protein {Methanosarcina acetivorans} |
| 23899 | TTHERM_00666460 | possible GTPases {Rhodopseudomonas palustris} |
| 24050 | TTHERM_01090200 | hypothetical protein {Rickettsia prowazekii} |
| 24290 | TTHERM_00133670 | ubiquinone/menaquinone biosynthesis methlytransferase ubiE {Rhodopseudomonas palustris} |
| 24986 | TTHERM_00997610 | Tryptophanyl-tRNA synthetase {Sinorhizobium meliloti} |
| 25099 | TTHERM_00595440 | immobilization antigen LA {Tetrahymena thermophila} |
| 25164 | TTHERM_00941580 | conserved hypothetical protein {Bordetella pertussis} |
| 25535 | TTHERM_00784310 | LD45826p {Drosophila melanogaster} |
| 25561 | TTHERM_00784570 | similar to Arabidopsis thaliana {Dictyostelium discoideum} |
| 25767 | TTHERM_00463150 | mitochondrial processing peptidase alpha-chain precursor {Dactylis glomerata} |
| 25999 | TTHERM_00290670 | Hydroxyacid oxidase 1 {Homo sapiens} |
| 26526 | TTHERM_00433850 | hypothetical protein {Caenorhabditis elegans} |
| 26713 | TTHERM_00145050 | probable Zinc-binding dehydrogenases {Leptospira interrogans} |
| 26715 | TTHERM_00145070 | probable Zinc-binding dehydrogenases {Leptospira interrogans} |
| 26716 | TTHERM_00145080 | NADH oxidoreductase {Leptospira interrogans} |
| 26751 | TTHERM_00145430 | conserved hypothetical protein {Coxiella burnetii} |
| 26839 | TTHERM_00146320 | hypothetical protein {Oryza sativa} |
| 30216 | TTHERM_00327130 | hypothetical protein {Escherichia coli} |
| 21918 | TTHERM_01609750 | Hypothetical WD-repeat protein {Nostoc sp. PCC 7120} |
| 15650 | TTHERM_00295400 | Glucose inhibited division protein A {Brucella melitensis} |
| 28163 | TTHERM_00975330 | ATP synthase mitochondrial F1 complex assembly factor 2 {Homo sapiens} |
| 27180 | TTHERM_00118610 | Hypothetical protein {Caenorhabditis elegans} |
| 27184 | TTHERM_00118680 | ABC transporter {Dictyostelium discoideum} |
| 28082 | TTHERM_01107330 | putative MAPK activating protein {Homo sapiens} |
| 28655 | TTHERM_00372410 | probable Zinc-binding dehydrogenases {Leptospira interrogans} |
