# Supplementary material for: Macronuclear Genome Sequence of the Ciliate Tetrahymena thermophila, a Model Eukaryote
Source: PLoS Biol. 2006 Aug 29;4(9):e286. doi: 10.1371/journal.pbio.0040286 (PMC1557398; doi:10.1371/journal.pbio.0040286)
Supplement: Supplementary file 16 [file pbio.0040286.st009.doc]

Table S9: Scaffolds with similarity to members of the *mariner*/*Tc1*/*IS630* superfamily in TBlastN searches. The asterisks indicates TEs shown to be micronucleus-limited by Southern blot analysis (Karrer and Sun, unpublished results). ITRs = inverted terminal repeats.

| **Scaffold #** | **Length (bp)** | **ITRs** | **Element** | **MIC copy number** |
| --- | --- | --- | --- | --- |
| 8253880 | 122320 |  |  |  |
| 8254015 * | 1483 | Both present | Tc1-like | 6 - 10 |
| 8254016 | 1477 |  |  |  |
| 8254036 * | 2940 | Both present | Tc1-like | 14 - 20 |
| 8254071 | 4294 |  |  |  |
| 8254087 | 1960 |  |  |  |
| 8254089 | 2100 |  |  |  |
| 8254091 | 1366 |  |  |  |
| 8254093 | 1868 | Possibly one | Tc1-like |  |
| 8254137 | 2766 |  |  |  |
| 8254227 | 1603 |  |  |  |
| 8254234 | 1569 |  |  |  |
| 8254311 | 1695 | Both present | Tc1-like |  |
| 8254320 | 2574 | One present | mariner-like |  |
| 8254324 | 1795 |  |  |  |
| 8254328 | 1541 |  |  |  |
| 8254611 | 521720 |  |  |  |
| 8254665 | 877599 |  |  |  |
| 8254666 | 596578 |  |  |  |
| 8254667 | 356833 |  |  |  |
| 8254839 | 1393 |  |  |  |
| 8255011 | 1402 |  |  |  |
| 8255124 | 1420 |  |  |  |
| 8255194 | 1340 |  |  |  |
| 8255210 | 1194 |  |  |  |
| 8255246 | 1046 | One present | mariner-like |  |
| 8255314 | 1076 |  |  |  |
| 8255399 | 1042 |  |  |  |
| 8255480 | 1121 |  |  |  |
| 8255512 | 1424 |  |  |  |
| 8255643 | 1125 |  |  |  |
| 8255680 | 1312 |  |  |  |
| 8255708 | 1395 |  |  |  |

NOTE: matches to large assemblies do not seem to correspond to complete transposases, although they may belong to more divergent elements.
