# Supplementary material for: Macronuclear Genome Sequence of the Ciliate Tetrahymena thermophila, a Model Eukaryote
Source: PLoS Biol. 2006 Aug 29;4(9):e286. doi: 10.1371/journal.pbio.0040286 (PMC1557398; doi:10.1371/journal.pbio.0040286)
Supplement: Supplementary file 17 [file pbio.0040286.st010.doc]

Table S10: Recent gene duplications

Serine Protease

1012-TTHERM_02290890

1218-TTHERM_00853190

1409-TTHERM_01144940

1410-TTHERM_01144950

1412-TTHERM_01144970

1413-TTHERM_01144980

1488-TTHERM_01540710

1490-TTHERM_01540730

1627-TTHERM_01364620

1871-TTHERM_01691290

2559-TTHERM_01337410

2678-TTHERM_01075660

2926-TTHERM_00694430

2927-TTHERM_00694440

2952-TTHERM_00691140

3378-TTHERM_01466260

3523-TTHERM_00313360

3526-TTHERM_00313390

3528-TTHERM_00313410

3582-TTHERM_01085460

3661-TTHERM_00655630

4184-TTHERM_00492480

4378-TTHERM_00925710

4897-TTHERM_00283580

4900-TTHERM_00283610

4932-TTHERM_00283930

4934-TTHERM_00283960

4935-TTHERM_00283970

5151-TTHERM_00621540

5201-TTHERM_01088000

5279-TTHERM_00348670

5538-TTHERM_01009800

5539-TTHERM_01009810

5540-TTHERM_01009820

5541-TTHERM_01009830

5544-TTHERM_01009860

5545-TTHERM_01009870

5553-TTHERM_01009950

5631-TTHERM_00442310

5738-TTHERM_00578450

5817-TTHERM_00579240

5818-TTHERM_00579250

5884-TTHERM_00951940

6236-TTHERM_00478070

6237-TTHERM_00478080

6239-TTHERM_00479090

6253-TTHERM_00481210

6269-TTHERM_00481370

6272-TTHERM_00482390

6348-TTHERM_00525110

6438-TTHERM_00527000

6513-TTHERM_00629910

6515-TTHERM_00629930

6573-TTHERM_00630600

6744-TTHERM_00194600

6747-TTHERM_00194630

6995-TTHERM_01749600

7541-TTHERM_01532560

7612-TTHERM_00678480

7646-TTHERM_00860400

7647-TTHERM_00860410

7649-TTHERM_00860430

7650-TTHERM_00860440

7651-TTHERM_00860450

7652-TTHERM_00860460

7656-TTHERM_00860500

7697-TTHERM_01642990

7717-TTHERM_01081650

7720-TTHERM_01081680

7728-TTHERM_01081760

7744-TTHERM_00821840

7917-TTHERM_00558200

7963-TTHERM_00558670

8242-TTHERM_02528180

8250-TTHERM_01169410

8261-TTHERM_01169520

8290-TTHERM_00979910

8291-TTHERM_00979920

8292-TTHERM_00979930

8293-TTHERM_00979940

8294-TTHERM_00979950

8295-TTHERM_00979960

8296-TTHERM_00980960

8360-TTHERM_00490550

8424-TTHERM_00491190

8426-TTHERM_00491210

8457-TTHERM_00826790

8558-TTHERM_00787310

8653-TTHERM_00189030

8782-TTHERM_00191310

8784-TTHERM_00191330

8844-TTHERM_00191940

9170-TTHERM_01628910

9484-TTHERM_00129320

9504-TTHERM_00129530

9505-TTHERM_00129540

9506-TTHERM_00129550

9510-TTHERM_00129590

9514-TTHERM_00129630

9535-TTHERM_00129840

9536-TTHERM_00129850

9537-TTHERM_00129860

9867-TTHERM_00268330

9868-TTHERM_00268340

9869-TTHERM_00268350

9873-TTHERM_00270370

9880-TTHERM_00274400

9883-TTHERM_00274430

9887-TTHERM_00274470

10047-TTHERM_00077010

10048-TTHERM_00077020

10089-TTHERM_00077430

10556-TTHERM_00638880

10558-TTHERM_00638900

10559-TTHERM_00638910

10561-TTHERM_00639920

10608-TTHERM_00643380

10699-TTHERM_00509140

10772-TTHERM_00519770

10832-TTHERM_01030020

10833-TTHERM_01030030

11062-TTHERM_00043850

11083-TTHERM_00045050

11164-TTHERM_00046850

11219-TTHERM_00047400

11220-TTHERM_00047410

11490-TTHERM_01373860

11491-TTHERM_01374860

11492-TTHERM_01374870

11493-TTHERM_01374880

11585-TTHERM_00002770

11587-TTHERM_00003780

11675-TTHERM_00008650

11692-TTHERM_00009810

11694-TTHERM_00009830

11698-TTHERM_00009870

11699-TTHERM_00009880

11899-TTHERM_00014930

11900-TTHERM_00014940

11901-TTHERM_00014950

12202-TTHERM_00540440

12442-TTHERM_00222350

12444-TTHERM_00222370

12448-TTHERM_00222410

12450-TTHERM_00223420

12455-TTHERM_00224460

12456-TTHERM_00224470

12461-TTHERM_00224520

12462-TTHERM_00224530

12463-TTHERM_00224540

12464-TTHERM_00224550

12578-TTHERM_00227680

12724-TTHERM_00400800

12976-TTHERM_00053950

13005-TTHERM_00057210

13006-TTHERM_00058210

13007-TTHERM_00058220

13008-TTHERM_00058230

13010-TTHERM_00058250

13135-TTHERM_00059510

13136-TTHERM_00059520

13137-TTHERM_00059530

13138-TTHERM_00059540

13140-TTHERM_00060550

13281-TTHERM_00070910

13491-TTHERM_02047520

13549-TTHERM_00415800

13552-TTHERM_00415830

13554-TTHERM_00417830

13555-TTHERM_00417840

13556-TTHERM_00417850

13557-TTHERM_00417860

13561-TTHERM_00417900

13562-TTHERM_00417910

13568-TTHERM_00417970

13570-TTHERM_00417990

13571-TTHERM_00418000

13819-TTHERM_00209410

13883-TTHERM_01237350

13888-TTHERM_01237400

13974-TTHERM_00423410

13975-TTHERM_00424410

13977-TTHERM_00424430

13978-TTHERM_00424440

13986-TTHERM_00424520

13989-TTHERM_00424550

13990-TTHERM_00424560

13991-TTHERM_00424570

13992-TTHERM_00424580

14018-TTHERM_00425830

14091-TTHERM_00427550

14241-TTHERM_00030100

14242-TTHERM_00030110

14245-TTHERM_00030140

14247-TTHERM_00030160

14459-TTHERM_00037240

14460-TTHERM_00037250

14495-TTHERM_00037610

14499-TTHERM_00037650

14561-TTHERM_00039260

14629-TTHERM_00884630

14645-TTHERM_00885810

14706-TTHERM_00746890

14727-TTHERM_01357370

14729-TTHERM_01358380

14871-TTHERM_00699750

14875-TTHERM_00699790

14964-TTHERM_00873660

14965-TTHERM_00873670

14998-TTHERM_00541520

14999-TTHERM_00541530

15000-TTHERM_00541540

15001-TTHERM_00541550

15002-TTHERM_00541560

15003-TTHERM_00542560

15004-TTHERM_00543560

15005-TTHERM_00543570

15006-TTHERM_00543580

15100-TTHERM_00549590

15191-TTHERM_01176660

15192-TTHERM_01178650

15193-TTHERM_01178660

15194-TTHERM_01178670

15195-TTHERM_01178680

15197-TTHERM_01178710

15198-TTHERM_01178720

15199-TTHERM_01178750

15347-TTHERM_01399660

15605-TTHERM_00294950

15626-TTHERM_00295160

15880-TTHERM_02640280

15910-TTHERM_00319900

16032-TTHERM_00323130

16033-TTHERM_00323140

16380-TTHERM_00085320

16381-TTHERM_00085330

16382-TTHERM_00085340

16447-TTHERM_00086980

16541-TTHERM_00092870

16549-TTHERM_00093940

17006-TTHERM_01603650

17181-TTHERM_01002610

17182-TTHERM_01002620

17462-TTHERM_00695660

17860-TTHERM_01082860

18289-TTHERM_00756180

18293-TTHERM_00756300

18296-TTHERM_00756330

18449-TTHERM_00721410

18766-TTHERM_01436770

18937-TTHERM_00241990

19228-TTHERM_00298260

19243-TTHERM_00298410

19309-TTHERM_00300140

19310-TTHERM_00300150

19350-TTHERM_00300550

19401-TTHERM_00302090

19427-TTHERM_01346830

19430-TTHERM_01346860

19432-TTHERM_01346880

19486-TTHERM_01485660

19504-TTHERM_01267140

19721-TTHERM_00637180

20348-TTHERM_00458180

20349-TTHERM_00458190

20350-TTHERM_00458200

20351-TTHERM_00458210

20367-TTHERM_00459360

20368-TTHERM_00459370

20568-TTHERM_01098980

21054-TTHERM_00318530

21146-TTHERM_00777320

21152-TTHERM_00778400

21228-TTHERM_00304220

21229-TTHERM_00304240

21237-TTHERM_00304320

21297-TTHERM_00309920

21298-TTHERM_00309930

21445-TTHERM_00794460

21559-TTHERM_00411410

21615-TTHERM_00411970

21707-TTHERM_01495760

21708-TTHERM_01495770

21737-TTHERM_00976510

21766-TTHERM_01418330

21767-TTHERM_01419330

21768-TTHERM_01419340

21790-TTHERM_01325790

21935-TTHERM_01476540

21968-TTHERM_00895870

21970-TTHERM_00895910

21974-TTHERM_00895950

22037-TTHERM_01510180

22038-TTHERM_01510190

22052-TTHERM_00599850

22058-TTHERM_00599920

22212-TTHERM_00215880

22422-TTHERM_00220940

22425-TTHERM_00220970

22772-TTHERM_01675220

22783-TTHERM_01212880

22832-TTHERM_00937730

22871-TTHERM_00096720

22872-TTHERM_00096740

22876-TTHERM_00096810

22877-TTHERM_00097810

22878-TTHERM_00097820

22879-TTHERM_00098820

22880-TTHERM_00099820

22882-TTHERM_00099840

22889-TTHERM_00099910

22893-TTHERM_00099950

22894-TTHERM_00099960

22895-TTHERM_00099970

22898-TTHERM_00100000

22900-TTHERM_00100020

22901-TTHERM_00100030

22904-TTHERM_00100060

22906-TTHERM_00100080

22952-TTHERM_00101530

22958-TTHERM_00102580

22959-TTHERM_00102590

22960-TTHERM_00102600

22961-TTHERM_00102610

22962-TTHERM_00102620

22963-TTHERM_00102630

22964-TTHERM_00102640

22965-TTHERM_00102650

22967-TTHERM_00102670

22968-TTHERM_00102680

22969-TTHERM_00102690

22971-TTHERM_00102710

23265-TTHERM_00982970

23266-TTHERM_00983970

23972-TTHERM_01321560

23976-TTHERM_01321600

23980-TTHERM_01321640

24033-TTHERM_01089010

24093-TTHERM_00586810

24206-TTHERM_01006490

24208-TTHERM_01006510

24210-TTHERM_01006530

24262-TTHERM_00133390

24322-TTHERM_00134980

24324-TTHERM_00135990

24345-TTHERM_00136200

24740-TTHERM_01102690

24866-TTHERM_00389700

25123-TTHERM_00597660

25124-TTHERM_00598660

25165-TTHERM_00941590

25166-TTHERM_00941600

25167-TTHERM_00941610

25625-TTHERM_00581870

25627-TTHERM_00581890

25978-TTHERM_00289470

25979-TTHERM_00289480

26189-TTHERM_00344100

26190-TTHERM_00344110

26192-TTHERM_00344130

26199-TTHERM_00344200

26279-TTHERM_00752110

26342-TTHERM_01159890

26380-TTHERM_01601640

26381-TTHERM_01602640

26898-TTHERM_00706420

26899-TTHERM_00706430

26900-TTHERM_00706440

26902-TTHERM_00706460

26903-TTHERM_00707460

26904-TTHERM_00707470

26916-TTHERM_00709560

26998-TTHERM_00942780

27000-TTHERM_00942800

27002-TTHERM_00942820

27003-TTHERM_00942830

27006-TTHERM_00942860

27007-TTHERM_00942870

27158-TTHERM_00115420

27159-TTHERM_00115430

27162-TTHERM_00115460

27164-TTHERM_00115480

27166-TTHERM_00115500

27167-TTHERM_00115510

27169-TTHERM_00115530

27170-TTHERM_00115540

27171-TTHERM_00116540

27172-TTHERM_00117540

27257-TTHERM_00122420

27312-TTHERM_00123980

27437-TTHERM_01460110

27601-TTHERM_00357150

27717-TTHERM_02596230

27838-TTHERM_00559770

27839-TTHERM_00559780

27845-TTHERM_00559840

27851-TTHERM_00559900

27976-TTHERM_00633070

28133-TTHERM_01713380

28238-TTHERM_01520330

28241-TTHERM_01520370

28269-TTHERM_01598640

28312-TTHERM_00153240

28314-TTHERM_00155240

28316-TTHERM_00155260

28320-TTHERM_00155300

28358-TTHERM_00156690

28447-TTHERM_00160550

28735-TTHERM_01545790

28738-TTHERM_01545840

28797-TTHERM_01026340

28864-TTHERM_01214990

29278-TTHERM_01600640

29557-TTHERM_01297400

29602-TTHERM_01393280

29603-TTHERM_01393290

29604-TTHERM_01393300

29718-TTHERM_00166110

30093-TTHERM_00944150

30116-TTHERM_02159680

30194-TTHERM_00326910

30195-TTHERM_00326920

Hypothetical Protein

1154-TTHERM_01362540

1443-TTHERM_00971730

1457-TTHERM_00971870

1458-TTHERM_00971880

1460-TTHERM_00971900

1894-TTHERM_01105020

2083-TTHERM_01080410

2377-TTHERM_00243820

2387-TTHERM_00243920

2509-TTHERM_00247140

2929-TTHERM_00694460

2932-TTHERM_00694490

2935-TTHERM_00694520

2985-TTHERM_00691470

3123-TTHERM_00535380

3287-TTHERM_00762960

3291-TTHERM_00763000

3308-TTHERM_00765150

3313-TTHERM_00765210

3315-TTHERM_00765230

3608-TTHERM_01085730

4094-TTHERM_00584870

4096-TTHERM_00584890

4713-TTHERM_00530500

4908-TTHERM_00283690

4957-TTHERM_00284210

5045-TTHERM_01046980

5049-TTHERM_01047020

5216-TTHERM_00348040

5523-TTHERM_00440690

5752-TTHERM_00578590

6011-TTHERM_00335660

6013-TTHERM_00335680

6118-TTHERM_00339770

6214-TTHERM_00476860

6354-TTHERM_00525170

6357-TTHERM_00526190

6467-TTHERM_00527350

6470-TTHERM_00527380

6505-TTHERM_00629770

6534-TTHERM_00630150

6575-TTHERM_00630620

6937-TTHERM_00497720

6938-TTHERM_00497730

6940-TTHERM_00497750

7531-TTHERM_01529550

7554-TTHERM_00676910

7772-TTHERM_00822170

7774-TTHERM_00822190

7828-TTHERM_00989380

8170-TTHERM_00757740

8203-TTHERM_00759060

8204-TTHERM_00759070

8206-TTHERM_00759090

8282-TTHERM_00979830

8387-TTHERM_00490820

8443-TTHERM_01401750

8446-TTHERM_01401780

8452-TTHERM_00826740

8455-TTHERM_00826770

8500-TTHERM_00827230

8503-TTHERM_00827260

8602-TTHERM_00188520

8607-TTHERM_00188570

8609-TTHERM_00188590

8870-TTHERM_01149270

9034-TTHERM_00566940

9159-TTHERM_01379950

9334-TTHERM_00125720

9573-TTHERM_01035620

9669-TTHERM_01322640

9672-TTHERM_01322670

9676-TTHERM_01323700

9716-TTHERM_00878190

9829-TTHERM_00267940

9859-TTHERM_00268250

9931-TTHERM_00275930

9964-TTHERM_00073220

9966-TTHERM_00073240

9968-TTHERM_00074250

10037-TTHERM_00075920

10040-TTHERM_00075950

10044-TTHERM_00076980

10600-TTHERM_00641320

10866-TTHERM_00717700

10867-TTHERM_00717710

10902-TTHERM_00718070

10904-TTHERM_00718090

11499-TTHERM_01404840

11505-TTHERM_01405890

11553-TTHERM_00001460

11555-TTHERM_00001480

11559-TTHERM_00001520

11563-TTHERM_00001560

11567-TTHERM_00002590

11569-TTHERM_00002610

11802-TTHERM_00011960

12066-TTHERM_01395380

12254-TTHERM_00854290

12489-TTHERM_00225790

12893-TTHERM_00654060

12897-TTHERM_00654100

12898-TTHERM_00654110

12909-TTHERM_00654220

13429-TTHERM_00363130

13430-TTHERM_00363140

13431-TTHERM_00363150

13432-TTHERM_00363160

13433-TTHERM_00363170

13444-TTHERM_00364270

13446-TTHERM_00364290

13448-TTHERM_00364310

13465-TTHERM_00365470

13469-TTHERM_00365510

13494-TTHERM_00414160

13497-TTHERM_00414190

13499-TTHERM_00414210

13694-TTHERM_00198260

13698-TTHERM_00198300

13703-TTHERM_00198350

13707-TTHERM_00198390

13711-TTHERM_00198430

13713-TTHERM_00198450

13722-TTHERM_00198540

13870-TTHERM_00214870

13964-TTHERM_00423310

14233-TTHERM_00030020

14316-TTHERM_00031840

14502-TTHERM_00037680

14605-TTHERM_00042670

14618-TTHERM_00884520

14652-TTHERM_00885880

14654-TTHERM_00885900

14655-TTHERM_00886900

14656-TTHERM_00886910

14669-TTHERM_00738560

14671-TTHERM_00738580

14699-TTHERM_00745820

14712-TTHERM_00748930

14714-TTHERM_00748950

14716-TTHERM_00748970

14901-TTHERM_00701040

14902-TTHERM_00701050

14906-TTHERM_00701090

15145-TTHERM_01013360

15148-TTHERM_01013390

15155-TTHERM_01273240

15160-TTHERM_01273290

15165-TTHERM_01275320

15697-TTHERM_00295870

15711-TTHERM_00296010

15713-TTHERM_00296030

15715-TTHERM_00297040

15716-TTHERM_00297050

15720-TTHERM_00297090

15896-TTHERM_01313370

15902-TTHERM_01317390

15904-TTHERM_01317410

15905-TTHERM_01317420

15962-TTHERM_00320450

15967-TTHERM_00320500

16702-TTHERM_02214810

17026-TTHERM_01586460

17401-TTHERM_00930880

17402-TTHERM_00930890

17602-TTHERM_01473440

17764-TTHERM_01526450

18015-TTHERM_01164110

18251-TTHERM_00755740

18279-TTHERM_00756020

18280-TTHERM_00756030

18463-TTHERM_00721550

18506-TTHERM_00760210

18508-TTHERM_00760230

18510-TTHERM_00760250

18720-TTHERM_00469180

18861-TTHERM_00239230

18863-TTHERM_00239250

18953-TTHERM_00242160

19079-TTHERM_00716070

19143-TTHERM_00615940

19210-TTHERM_00616610

19211-TTHERM_00616620

19212-TTHERM_00616630

19484-TTHERM_01125340

19499-TTHERM_01266100

20556-TTHERM_00899500

20774-TTHERM_00151130

20931-TTHERM_00316200

21124-TTHERM_00777100

21125-TTHERM_00777110

21129-TTHERM_00777150

21133-TTHERM_00777190

21135-TTHERM_00777210

21138-TTHERM_00777240

21305-TTHERM_00310000

21308-TTHERM_00310030

21311-TTHERM_00310060

21723-TTHERM_01433610

21788-TTHERM_01325770

21832-TTHERM_00724670

21938-TTHERM_00895560

22017-TTHERM_01052940

22162-TTHERM_00901670

22164-TTHERM_00901690

22168-TTHERM_00901730

22170-TTHERM_00901750

22173-TTHERM_00901780

22180-TTHERM_00903830

22279-TTHERM_00218520

22695-TTHERM_00800260

23035-TTHERM_00105350

23039-TTHERM_00105390

23040-TTHERM_00105400

23211-TTHERM_00796700

23218-TTHERM_00797770

23248-TTHERM_00798090

23291-TTHERM_00985210

23293-TTHERM_00985230

23297-TTHERM_00986260

23323-TTHERM_00890110

23719-TTHERM_00912120

23722-TTHERM_00912150

23724-TTHERM_00912170

23727-TTHERM_00912200

23729-TTHERM_00912220

23835-TTHERM_00663880

23943-TTHERM_00666910

23984-TTHERM_01547860

24035-TTHERM_01089030

24038-TTHERM_01089070

24040-TTHERM_01090090

24044-TTHERM_01090140

24047-TTHERM_01090170

24050-TTHERM_01090200

24099-TTHERM_00588850

24221-TTHERM_01553980

24222-TTHERM_01553990

24953-TTHERM_00849520

24954-TTHERM_00850520

25103-TTHERM_00595480

25109-TTHERM_00595540

25112-TTHERM_00596560

25116-TTHERM_00596600

25120-TTHERM_00597630

25121-TTHERM_00597640

25122-TTHERM_00597650

25327-TTHERM_00253440

25328-TTHERM_00253450

25622-TTHERM_00581840

25663-TTHERM_00582270

25706-TTHERM_00460550

25876-TTHERM_00285470

25914-TTHERM_00286840

25917-TTHERM_00286870

26065-TTHERM_00998860

26087-TTHERM_00999080

26244-TTHERM_00346630

26245-TTHERM_00346640

26247-TTHERM_00346660

26249-TTHERM_00346680

26251-TTHERM_00346700

26254-TTHERM_00346740

26820-TTHERM_00146130

26823-TTHERM_00146160

26825-TTHERM_00146180

26826-TTHERM_00146190

27100-TTHERM_00112860

27102-TTHERM_00112880

27104-TTHERM_00112900

27896-TTHERM_00561350

28136-TTHERM_00972990

28139-TTHERM_00973020

28140-TTHERM_00973030

28143-TTHERM_00974050

28145-TTHERM_00974070

28172-TTHERM_00975420

28212-TTHERM_01389210

28213-TTHERM_01389220

28218-TTHERM_01389270

28670-TTHERM_00372560

28679-TTHERM_00372650

28682-TTHERM_00372680

29049-TTHERM_00845800

29050-TTHERM_00845810

29124-TTHERM_00864940

29127-TTHERM_00864970

29131-TTHERM_00865010

29221-TTHERM_00551170

29223-TTHERM_00551190

29236-TTHERM_00554290

29291-TTHERM_00258260

29295-TTHERM_00259290

29674-TTHERM_01198170

29678-TTHERM_01201180

29685-TTHERM_01204220

29953-TTHERM_00810490

29956-TTHERM_00810520

29960-TTHERM_00811550

29964-TTHERM_00811590

29968-TTHERM_00812620

30014-TTHERM_00813080

30198-TTHERM_00326950

30303-TTHERM_00329980

K+channel Protein

1076-TTHERM_00670520

1077-TTHERM_00670530

1353-TTHERM_00790630

1354-TTHERM_00790640

1356-TTHERM_00790660

1679-TTHERM_01262810

2397-TTHERM_00245010

2426-TTHERM_00245310

2432-TTHERM_00245370

2433-TTHERM_00245380

2434-TTHERM_00245390

2435-TTHERM_00245400

2555-TTHERM_01337370

2651-TTHERM_01016170

2966-TTHERM_00691280

3417-TTHERM_00312300

3470-TTHERM_00312830

3471-TTHERM_00312840

3473-TTHERM_00312860

3808-TTHERM_00444400

4062-TTHERM_00584540

4118-TTHERM_00585120

4155-TTHERM_00585490

4289-TTHERM_00494610

4415-TTHERM_00773240

4521-TTHERM_01106070

5298-TTHERM_00348860

5453-TTHERM_00438960

5454-TTHERM_00438970

5456-TTHERM_00438990

5628-TTHERM_00442280

6256-TTHERM_00481240

6336-TTHERM_01353180

6337-TTHERM_01353190

6339-TTHERM_01353210

6413-TTHERM_00526750

6702-TTHERM_00194180

6704-TTHERM_00194200

6779-TTHERM_00195940

6780-TTHERM_00195950

6781-TTHERM_00195960

7057-TTHERM_00919610

7105-TTHERM_00419750

7163-TTHERM_00420380

7452-TTHERM_00624640

7831-TTHERM_00989410

7881-TTHERM_00557830

7892-TTHERM_00557940

7918-TTHERM_00558210

8140-TTHERM_01068280

8196-TTHERM_00758990

8197-TTHERM_00759000

8198-TTHERM_00759010

8201-TTHERM_00759040

8202-TTHERM_00759050

8463-TTHERM_00826850

8804-TTHERM_00191530

8857-TTHERM_00192070

8965-TTHERM_00564270

9195-TTHERM_00648810

9491-TTHERM_00129390

9493-TTHERM_00129410

9976-TTHERM_00074330

9977-TTHERM_00074340

10053-TTHERM_00077070

10094-TTHERM_00077480

10374-TTHERM_00382170

10856-TTHERM_00717600

10857-TTHERM_00717610

10858-TTHERM_00717620

10859-TTHERM_00717630

10861-TTHERM_00717650

10862-TTHERM_00717660

10863-TTHERM_00717670

10972-TTHERM_00672260

11528-TTHERM_00001210

11680-TTHERM_00008700

11834-TTHERM_00013280

12150-TTHERM_00538930

12256-TTHERM_00854310

12260-TTHERM_00854350

12535-TTHERM_00227250

12685-TTHERM_00399410

13053-TTHERM_00058680

13270-TTHERM_00070800

13273-TTHERM_00070830

13439-TTHERM_00363230

13636-TTHERM_00197680

13822-TTHERM_00209440

13930-TTHERM_00770890

13931-TTHERM_00770910

13932-TTHERM_00770930

13945-TTHERM_00772070

13996-TTHERM_00424620

13997-TTHERM_00424630

13998-TTHERM_00424640

14086-TTHERM_00427500

14087-TTHERM_00427510

14088-TTHERM_00427520

14089-TTHERM_00427530

14090-TTHERM_00427540

14202-TTHERM_00028720

14292-TTHERM_00031600

14293-TTHERM_00031610

14294-TTHERM_00031620

14466-TTHERM_00037310

14660-TTHERM_00886950

14890-TTHERM_00700930

15664-TTHERM_00295540

15710-TTHERM_00296000

15972-TTHERM_00321540

16097-TTHERM_00680610

16099-TTHERM_00680630

16100-TTHERM_00680640

16102-TTHERM_00680660

16127-TTHERM_00681910

16133-TTHERM_00682960

16304-TTHERM_00083570

16305-TTHERM_00083580

16306-TTHERM_00083590

16307-TTHERM_00083600

16308-TTHERM_00083610

17210-TTHERM_01002900

17434-TTHERM_01079190

17519-TTHERM_00697230

17844-TTHERM_01277490

17847-TTHERM_01277520

17848-TTHERM_01277530

17957-TTHERM_01109870

17961-TTHERM_01109910

18217-TTHERM_00686030

18235-TTHERM_00686210

18361-TTHERM_00993000

18436-TTHERM_00721280

18437-TTHERM_00721290

18480-TTHERM_00721720

18527-TTHERM_00760420

18529-TTHERM_00760440

18570-TTHERM_00760850

18736-TTHERM_00469340

18749-TTHERM_01326850

18900-TTHERM_00241620

19063-TTHERM_00715890

19168-TTHERM_00616190

19899-TTHERM_00532410

20500-TTHERM_01250070

20502-TTHERM_01250090

20708-TTHERM_00149490

20824-TTHERM_00151660

20831-TTHERM_00151730

20834-TTHERM_00151760

20968-TTHERM_00316570

21164-TTHERM_00922850

21249-TTHERM_00305430

21281-TTHERM_00307780

21282-TTHERM_00307790

21295-TTHERM_00309900

21348-TTHERM_00310440

21363-TTHERM_00310590

21485-TTHERM_00407700

21691-TTHERM_01295350

21692-TTHERM_01295360

21721-TTHERM_01433590

21753-TTHERM_00977660

21757-TTHERM_00977700

21758-TTHERM_00977710

21881-TTHERM_00726150

21886-TTHERM_00726210

21889-TTHERM_00726240

22089-TTHERM_00600260

22272-TTHERM_00218450

22305-TTHERM_00218780

22470-TTHERM_00470560

22556-TTHERM_00471460

22558-TTHERM_00471480

23157-TTHERM_00688620

23334-TTHERM_00891210

23404-TTHERM_00808080

23412-TTHERM_00809160

23416-TTHERM_00809210

23417-TTHERM_00809220

23418-TTHERM_00809230

23430-TTHERM_00809350

23432-TTHERM_00809370

23564-TTHERM_00393410

23741-TTHERM_00913370

23839-TTHERM_00663920

24178-TTHERM_00591630

24196-TTHERM_01006390

24457-TTHERM_00138300

24582-TTHERM_01121620

24583-TTHERM_01121630

24683-TTHERM_00628570

24704-TTHERM_01184150

24712-TTHERM_01184230

24976-TTHERM_00997510

24978-TTHERM_00997530

24979-TTHERM_00997540

24980-TTHERM_00997550

24983-TTHERM_00997580

24984-TTHERM_00997590

25318-TTHERM_00252360

25321-TTHERM_00252390

25538-TTHERM_00784340

25589-TTHERM_00581460

25590-TTHERM_00581470

25925-TTHERM_00287940

26177-TTHERM_00343980

26178-TTHERM_00343990

26179-TTHERM_00344000

26263-TTHERM_00346880

26410-TTHERM_00429690

26720-TTHERM_00145120

26721-TTHERM_00145130

26726-TTHERM_00145180

27271-TTHERM_00123550

27619-TTHERM_01005020

27624-TTHERM_01005070

27782-TTHERM_01050390

28423-TTHERM_00158330

28557-TTHERM_00161670

28570-TTHERM_00161800

29183-TTHERM_00550750

29184-TTHERM_00550760

29186-TTHERM_00550780

29187-TTHERM_00550790

29404-TTHERM_00263390

29405-TTHERM_00263400

29413-TTHERM_00263480

29698-TTHERM_00163930

29699-TTHERM_00163940

29701-TTHERM_00164950

29702-TTHERM_00164960

29703-TTHERM_00164970

29704-TTHERM_00164980

29705-TTHERM_00164990

29706-TTHERM_00165000

29816-TTHERM_00180980

30217-TTHERM_00327140

interaptin

1816-TTHERM_01042000

2396-TTHERM_00244010

2399-TTHERM_00245030

2400-TTHERM_00245040

2401-TTHERM_00245050

3593-TTHERM_01085580

3628-TTHERM_00655300

3636-TTHERM_00655380

3637-TTHERM_00655390

4073-TTHERM_00584650

4167-TTHERM_00492310

4325-TTHERM_01142700

4679-TTHERM_00530100

4680-TTHERM_00530110

4681-TTHERM_00530120

4977-TTHERM_00954010

4978-TTHERM_00954020

4979-TTHERM_00954030

4981-TTHERM_00954050

4982-TTHERM_00954060

4984-TTHERM_00954080

4985-TTHERM_00954090

4986-TTHERM_00954100

4987-TTHERM_00954110

4988-TTHERM_00954120

4990-TTHERM_00954140

4992-TTHERM_00954160

5418-TTHERM_00437620

5556-TTHERM_01009980

5557-TTHERM_01009990

5873-TTHERM_00951830

5874-TTHERM_00951840

5876-TTHERM_00951860

5877-TTHERM_00951870

5879-TTHERM_00951890

5880-TTHERM_00951900

5945-TTHERM_01084260

6453-TTHERM_00527210

6454-TTHERM_00527220

6455-TTHERM_00527230

6456-TTHERM_00527240

6578-TTHERM_00630650

6962-TTHERM_00497970

6963-TTHERM_00497980

7615-TTHERM_00678510

7648-TTHERM_00860420

7653-TTHERM_00860470

7654-TTHERM_00860480

7725-TTHERM_01081730

8187-TTHERM_00758900

8188-TTHERM_00758910

8189-TTHERM_00758920

8190-TTHERM_00758930

8213-TTHERM_00759160

8325-TTHERM_00488220

8326-TTHERM_00488230

8327-TTHERM_00488240

8329-TTHERM_00488260

8330-TTHERM_00488270

8331-TTHERM_00488280

8397-TTHERM_00490920

8797-TTHERM_00191460

8798-TTHERM_00191470

9982-TTHERM_00074390

9983-TTHERM_00074400

9984-TTHERM_00074410

9986-TTHERM_00074430

9991-TTHERM_00075470

9993-TTHERM_00075490

9994-TTHERM_00075500

9995-TTHERM_00075510

9997-TTHERM_00075530

10078-TTHERM_00077320

10079-TTHERM_00077330

10574-TTHERM_00640060

10575-TTHERM_00640070

10576-TTHERM_00641070

10890-TTHERM_00717950

10892-TTHERM_00717970

11589-TTHERM_00004790

11590-TTHERM_00004800

12157-TTHERM_00539000

12158-TTHERM_00539010

12159-TTHERM_00540010

12160-TTHERM_00540020

12161-TTHERM_00540030

12162-TTHERM_00540040

12501-TTHERM_00225910

12503-TTHERM_00225930

12504-TTHERM_00225940

12507-TTHERM_00225970

12508-TTHERM_00225980

12509-TTHERM_00225990

12592-TTHERM_00227820

12595-TTHERM_00227850

12696-TTHERM_00399530

12702-TTHERM_00399590

12734-TTHERM_00401890

12736-TTHERM_00401910

12737-TTHERM_00401920

13192-TTHERM_00068030

13193-TTHERM_00068040

13194-TTHERM_00068050

13391-TTHERM_00361760

13395-TTHERM_00361800

13454-TTHERM_00365360

14041-TTHERM_00426060

14235-TTHERM_00030040

14479-TTHERM_00037440

14480-TTHERM_00037450

14737-TTHERM_01415160

14956-TTHERM_00872590

14957-TTHERM_00872600

15343-TTHERM_01399600

15364-TTHERM_01330010

15366-TTHERM_01330030

15367-TTHERM_01330040

15368-TTHERM_01330050

15789-TTHERM_00646830

15790-TTHERM_00646840

15854-TTHERM_00647480

15855-TTHERM_00647490

15934-TTHERM_00320170

15977-TTHERM_00321590

15981-TTHERM_00321630

15987-TTHERM_00321690

15988-TTHERM_00321700

15989-TTHERM_00321710

16049-TTHERM_00324440

16050-TTHERM_00324450

16051-TTHERM_00324460

16279-TTHERM_00083320

16280-TTHERM_00083330

16452-TTHERM_00088020

17179-TTHERM_01002590

17490-TTHERM_00696930

18060-TTHERM_00723000

18648-TTHERM_00467460

19122-TTHERM_00614740

19124-TTHERM_00614760

19126-TTHERM_00614780

20049-TTHERM_01141440

20052-TTHERM_01141470

20053-TTHERM_01141480

20059-TTHERM_01141540

20061-TTHERM_01141560

20297-TTHERM_00455680

20300-TTHERM_00456700

20828-TTHERM_00151700

20922-TTHERM_00316110

20923-TTHERM_00316120

22384-TTHERM_00219570

22864-TTHERM_00095650

22870-TTHERM_00096700

22953-TTHERM_00101540

22954-TTHERM_00101550

22956-TTHERM_00102560

22957-TTHERM_00102570

23389-TTHERM_00807930

23449-TTHERM_00391220

23451-TTHERM_00391240

23453-TTHERM_00391260

24192-TTHERM_01006350

24193-TTHERM_01006360

24253-TTHERM_00131320

24537-TTHERM_00835220

24541-TTHERM_00835260

24958-TTHERM_00850560

24998-TTHERM_00997730

25000-TTHERM_00997750

25030-TTHERM_00592770

25579-TTHERM_00784760

25582-TTHERM_00784800

25609-TTHERM_00581710

25668-TTHERM_00582320

25670-TTHERM_00582340

25671-TTHERM_00582350

25672-TTHERM_00582360

25673-TTHERM_00582370

25674-TTHERM_00582380

25675-TTHERM_00582390

25676-TTHERM_00582400

26202-TTHERM_00344230

26437-TTHERM_00429970

27292-TTHERM_00123780

27293-TTHERM_00123790

27294-TTHERM_00123800

27954-TTHERM_00632850

27979-TTHERM_00633100

27980-TTHERM_00633110

28609-TTHERM_00370930

28611-TTHERM_00370950

28612-TTHERM_00370960

28613-TTHERM_00370970

28614-TTHERM_00370980

28699-TTHERM_00373850

28921-TTHERM_00574320

29082-TTHERM_00848110

29252-TTHERM_00554450

29253-TTHERM_00554460

29257-TTHERM_00554500

29259-TTHERM_00554520

29261-TTHERM_00554540

29264-TTHERM_00554570

29832-TTHERM_00181140

29833-TTHERM_00181150

29834-TTHERM_00182150

29835-TTHERM_00185130

30066-TTHERM_01467290

30271-TTHERM_00328670

30272-TTHERM_00328680

interaptin

1200-TTHERM_00853010

2174-TTHERM_00658890

2185-TTHERM_00659000

2336-TTHERM_00475370

2821-TTHERM_01100300

2822-TTHERM_01100310

2823-TTHERM_01100320

2824-TTHERM_01100330

2825-TTHERM_01100340

2826-TTHERM_01100350

2924-TTHERM_00694410

2925-TTHERM_00694420

3507-TTHERM_00313200

3508-TTHERM_00313210

3509-TTHERM_00313220

3510-TTHERM_00313230

3512-TTHERM_00313250

3513-TTHERM_00313260

3538-TTHERM_00313510

3640-TTHERM_00655420

3641-TTHERM_00655430

5740-TTHERM_00578470

5790-TTHERM_00578970

5792-TTHERM_00578990

5918-TTHERM_01044690

5919-TTHERM_01044700

7002-TTHERM_01156800

7114-TTHERM_00419840

7254-TTHERM_00727530

7282-TTHERM_00727810

8127-TTHERM_01068120

9102-TTHERM_00859400

10232-TTHERM_00079870

10692-TTHERM_00509070

10874-TTHERM_00717790

10875-TTHERM_00717800

10932-TTHERM_01133970

11597-TTHERM_00004870

11598-TTHERM_00004880

11798-TTHERM_00011920

12656-TTHERM_00398100

12657-TTHERM_00398110

12658-TTHERM_00398120

12659-TTHERM_00398130

12660-TTHERM_00398140

12661-TTHERM_00399140

12662-TTHERM_00399150

12663-TTHERM_00399160

12672-TTHERM_00399280

12673-TTHERM_00399290

12674-TTHERM_00399300

12677-TTHERM_00399330

12790-TTHERM_00405430

13041-TTHERM_00058560

13042-TTHERM_00058570

13529-TTHERM_00415570

13824-TTHERM_00209460

13827-TTHERM_00210480

14258-TTHERM_00030270

14259-TTHERM_00030280

14305-TTHERM_00031730

14548-TTHERM_00039130

14549-TTHERM_00039140

14550-TTHERM_00039150

14551-TTHERM_00039160

14552-TTHERM_00039170

14553-TTHERM_00039180

14554-TTHERM_00039190

15137-TTHERM_01013280

15138-TTHERM_01013290

15751-TTHERM_01001320

15752-TTHERM_01001330

16566-TTHERM_00094110

16880-TTHERM_00713280

16881-TTHERM_00713290

16882-TTHERM_00713300

16883-TTHERM_00713310

16884-TTHERM_00713320

17205-TTHERM_01002850

17458-TTHERM_00695620

18326-TTHERM_00992640

18327-TTHERM_00992650

18328-TTHERM_00992660

18329-TTHERM_00992670

18955-TTHERM_00242180

18956-TTHERM_00242190

18957-TTHERM_00242200

19944-TTHERM_00532880

19945-TTHERM_00532890

20483-TTHERM_02245830

21591-TTHERM_00411730

21627-TTHERM_00412090

21629-TTHERM_00412110

23420-TTHERM_00809250

23421-TTHERM_00809260

23424-TTHERM_00809290

23427-TTHERM_00809320

23428-TTHERM_00809330

23843-TTHERM_00663960

24098-TTHERM_00587850

24145-TTHERM_00590310

24758-TTHERM_00384660

26052-TTHERM_00292200

27035-TTHERM_00109240

27038-TTHERM_00109270

27041-TTHERM_00111280

27042-TTHERM_00112280

27452-TTHERM_00354650

27455-TTHERM_00354690

27545-TTHERM_00355590

27720-TTHERM_02242830

28421-TTHERM_00158310

28984-TTHERM_00576970

29110-TTHERM_00863800

29836-TTHERM_00185140

29838-TTHERM_00185160

29920-TTHERM_00186040

30055-TTHERM_01129750

FLJ00180 protein

1659-TTHERM_01232230

1792-TTHERM_01038790

2579-TTHERM_01308060

2665-TTHERM_01580350

2667-TTHERM_01580370

2706-TTHERM_01453050

3082-TTHERM_01451020

3933-TTHERM_01434640

4375-TTHERM_00925680

4377-TTHERM_00925700

4740-TTHERM_00530770

4806-TTHERM_00278640

4974-TTHERM_00953980

4975-TTHERM_00953990

4989-TTHERM_00954130

5035-TTHERM_01046880

5857-TTHERM_00949690

5871-TTHERM_00951810

5872-TTHERM_00951820

5999-TTHERM_00334540

6577-TTHERM_00630640

6580-TTHERM_00630670

7011-TTHERM_01156890

7302-TTHERM_00729010

7632-TTHERM_01369750

7681-TTHERM_00862730

7812-TTHERM_01117360

7845-TTHERM_00991530

7852-TTHERM_00991600

8307-TTHERM_01472400

8439-TTHERM_01557020

8901-TTHERM_01251280

9130-TTHERM_01309070

9649-TTHERM_00820630

9955-TTHERM_00072130

10022-TTHERM_00075770

10080-TTHERM_00077340

10265-TTHERM_01397460

10266-TTHERM_01397470

11395-TTHERM_00052150

11541-TTHERM_00001340

11964-TTHERM_00016580

12077-TTHERM_01577290

12218-TTHERM_01336330

12285-TTHERM_00856600

12722-TTHERM_00400780

13647-TTHERM_00197790

13826-TTHERM_00209480

13962-TTHERM_00423290

14476-TTHERM_00037410

14601-TTHERM_00041640

14695-TTHERM_00745780

14776-TTHERM_01118370

14857-TTHERM_00698620

14930-TTHERM_01542770

14967-TTHERM_00873690

14976-TTHERM_00874770

14977-TTHERM_00874780

14978-TTHERM_00874790

14982-TTHERM_00875820

14988-TTHERM_00875880

15205-TTHERM_01442810

15224-TTHERM_01062860

15356-TTHERM_01465230

16540-TTHERM_00092860

16859-TTHERM_00711990

16937-TTHERM_01093640

16990-TTHERM_01260670

17442-TTHERM_01079270

17480-TTHERM_00695840

17904-TTHERM_00932060

18577-TTHERM_01182010

19009-TTHERM_01573200

19016-TTHERM_01318420

19027-TTHERM_01319520

19491-TTHERM_01485710

19805-TTHERM_01054110

19910-TTHERM_00532540

20028-TTHERM_00994320

20519-TTHERM_00898140

20539-TTHERM_00898340

21291-TTHERM_00309860

21645-TTHERM_00956500

21646-TTHERM_00956510

21696-TTHERM_01359430

21731-TTHERM_00976450

21784-TTHERM_01324740

21785-TTHERM_01325740

21966-TTHERM_00895840

22721-TTHERM_00802510

22820-TTHERM_00936620

23222-TTHERM_00797810

23313-TTHERM_00888030

23534-TTHERM_00393060

23878-TTHERM_00666250

25194-TTHERM_01449000

25387-TTHERM_00257010

25453-TTHERM_00735290

25455-TTHERM_00735310

25494-TTHERM_01209750

26097-TTHERM_01420340

27416-TTHERM_00611560

27693-TTHERM_01074590

27761-TTHERM_01018440

27956-TTHERM_00632870

28043-TTHERM_00635720

28047-TTHERM_00635760

28581-TTHERM_00370650

28809-TTHERM_01026460

28823-TTHERM_01166320

29597-TTHERM_01246770

29605-TTHERM_01393310

30128-TTHERM_01147130

30283-TTHERM_00329780

hypothetical protein

1181-TTHERM_00852820

2276-TTHERM_00474760

2464-TTHERM_00245690

2672-TTHERM_01075600

3911-TTHERM_00446440

4082-TTHERM_00584740

4084-TTHERM_00584760

5479-TTHERM_00439220

6197-TTHERM_00476680

6873-TTHERM_00497070

6874-TTHERM_00497080

7338-TTHERM_01244550

7694-TTHERM_01347940

8281-TTHERM_00979820

8285-TTHERM_00979860

9217-TTHERM_00649030

9219-TTHERM_00649050

9509-TTHERM_00129580

9515-TTHERM_00129640

9539-TTHERM_00129880

9785-TTHERM_00266500

9866-TTHERM_00268320

9925-TTHERM_00275860

10812-TTHERM_01028830

10814-TTHERM_01028850

10839-TTHERM_00717410

10840-TTHERM_00717420

10841-TTHERM_00717430

10842-TTHERM_00717440

10843-TTHERM_00717450

11017-TTHERM_00675760

11089-TTHERM_00046100

11705-TTHERM_00010930

11706-TTHERM_00010940

11867-TTHERM_00013610

11871-TTHERM_00013650

12194-TTHERM_00540360

12197-TTHERM_00540390

12198-TTHERM_00540400

12842-TTHERM_00652560

12843-TTHERM_00652570

12958-TTHERM_00053770

13043-TTHERM_00058580

13075-TTHERM_00058900

13268-TTHERM_00070780

13650-TTHERM_00197820

13651-TTHERM_00197830

13742-TTHERM_00201710

14149-TTHERM_00833660

14593-TTHERM_00041560

14823-TTHERM_01226690

14825-TTHERM_01227700

15900-TTHERM_01317370

15954-TTHERM_00320370

15956-TTHERM_00320390

15957-TTHERM_00320400

15958-TTHERM_00320410

15959-TTHERM_00320420

16004-TTHERM_00322850

16008-TTHERM_00322890

16057-TTHERM_00324520

16058-TTHERM_00324530

17503-TTHERM_00697060

18557-TTHERM_00760720

18559-TTHERM_00760740

18560-TTHERM_00760750

19266-TTHERM_00299710

19414-TTHERM_01285840

19416-TTHERM_01285860

19417-TTHERM_01285870

19418-TTHERM_01285880

19419-TTHERM_01285890

19447-TTHERM_01264990

19448-TTHERM_01265000

19450-TTHERM_01265020

20122-TTHERM_00703460

20884-TTHERM_01589520

21382-TTHERM_00310810

21486-TTHERM_00408700

21738-TTHERM_00976520

21971-TTHERM_00895920

22134-TTHERM_00600710

23934-TTHERM_00666820

24638-TTHERM_00627130

24679-TTHERM_00628530

24680-TTHERM_00628540

25685-TTHERM_00583480

26070-TTHERM_00998910

26623-TTHERM_00141180

26624-TTHERM_00141190

26625-TTHERM_00141200

26626-TTHERM_00142200

26627-TTHERM_00142210

26628-TTHERM_00142220

26631-TTHERM_00142250

26632-TTHERM_00142260

27034-TTHERM_00108240

27036-TTHERM_00109250

27037-TTHERM_00109260

27415-TTHERM_00610560

28317-TTHERM_00155270

28318-TTHERM_00155280

28909-TTHERM_00573210

28910-TTHERM_00573220

29088-TTHERM_00848170

29089-TTHERM_00848180

29090-TTHERM_00848190

29753-TTHERM_00170450

29754-TTHERM_00170460

30163-TTHERM_00325610

cysteine proteinase

1206-TTHERM_00853070

1247-TTHERM_02592230

3001-TTHERM_00691630

4392-TTHERM_00925850

4394-TTHERM_00925870

4729-TTHERM_00530660

5481-TTHERM_00439240

5482-TTHERM_00439250

6274-TTHERM_00483400

6275-TTHERM_00483410

6344-TTHERM_00525070

6517-TTHERM_00629950

7899-TTHERM_00558010

9463-TTHERM_00128990

9464-TTHERM_00129000

9465-TTHERM_00129010

9718-TTHERM_00878210

9721-TTHERM_00879230

9723-TTHERM_00879250

9725-TTHERM_00879270

9726-TTHERM_00879280

9727-TTHERM_00879290

9728-TTHERM_00879300

9732-TTHERM_00881320

9733-TTHERM_00881330

9737-TTHERM_00881370

9738-TTHERM_00881380

9739-TTHERM_00881390

9741-TTHERM_00881410

9742-TTHERM_00881420

9744-TTHERM_00881440

9745-TTHERM_00881450

9840-TTHERM_00268050

9841-TTHERM_00268060

10029-TTHERM_00075840

10034-TTHERM_00075890

10184-TTHERM_00079380

10191-TTHERM_00079450

10200-TTHERM_00079550

10203-TTHERM_00079580

10205-TTHERM_00079600

10206-TTHERM_00079610

10207-TTHERM_00079620

10208-TTHERM_00079630

10209-TTHERM_00079640

10210-TTHERM_00079650

11826-TTHERM_00013200

13257-TTHERM_00069680

13595-TTHERM_00418260

14804-TTHERM_01256490

14807-TTHERM_01256520

14974-TTHERM_00874750

15946-TTHERM_00320290

16138-TTHERM_00683010

16139-TTHERM_00683020

16143-TTHERM_00683060

16245-TTHERM_00842600

17099-TTHERM_01354260

17100-TTHERM_01354270

18272-TTHERM_00755950

18516-TTHERM_00760310

19864-TTHERM_00532060

21700-TTHERM_01359470

21701-TTHERM_01359480

21954-TTHERM_00895720

22705-TTHERM_00802340

23778-TTHERM_00660340

23779-TTHERM_00660350

23780-TTHERM_00660360

23782-TTHERM_00660380

23783-TTHERM_00660390

23785-TTHERM_00660410

23786-TTHERM_00660420

23788-TTHERM_00660440

23789-TTHERM_00660450

24088-TTHERM_00586760

24687-TTHERM_00628610

25447-TTHERM_00735230

27689-TTHERM_01074550

28717-TTHERM_00375030

29570-TTHERM_01008660

30108-TTHERM_00945290

30109-TTHERM_00945300

interaptin

3083-TTHERM_01452020

4051-TTHERM_01592570

4052-TTHERM_01592580

4384-TTHERM_00925770

10941-TTHERM_00671950

14126-TTHERM_00829470

14127-TTHERM_00829480

14128-TTHERM_00829490

14131-TTHERM_00830510

14132-TTHERM_00831510

14133-TTHERM_00831520

14134-TTHERM_00831530

14136-TTHERM_00831550

14137-TTHERM_00831560

14138-TTHERM_00831570

14143-TTHERM_00832610

14144-TTHERM_00833610

14147-TTHERM_00833640

14161-TTHERM_00833780

14162-TTHERM_00833790

14163-TTHERM_00833800

14164-TTHERM_00833810

14165-TTHERM_00833820

14544-TTHERM_00039090

14759-TTHERM_01188420

14942-TTHERM_00866510

14944-TTHERM_00866530

14960-TTHERM_00873620

16792-TTHERM_02540180

17399-TTHERM_00930860

17871-TTHERM_01082970

18130-TTHERM_01579310

18588-TTHERM_01183110

18589-TTHERM_01183120

18590-TTHERM_01183130

18591-TTHERM_01183140

23239-TTHERM_00798000

23240-TTHERM_00798010

24521-TTHERM_00835060

24522-TTHERM_00835070

24523-TTHERM_00835080

24524-TTHERM_00835090

24744-TTHERM_01102730

24745-TTHERM_01102740

24746-TTHERM_01102750

24747-TTHERM_01102760

24748-TTHERM_01102770

24749-TTHERM_01102780

27747-TTHERM_01018290

27748-TTHERM_01018300

27749-TTHERM_01018310

27750-TTHERM_01018320

28069-TTHERM_02539180

28283-TTHERM_01217230

28284-TTHERM_01217240

29655-TTHERM_00965460

29657-TTHERM_00965480

29658-TTHERM_00966480

29659-TTHERM_00966490

29660-TTHERM_00966500

29661-TTHERM_00966510

29662-TTHERM_00966520

29663-TTHERM_00967520

29664-TTHERM_00967530

29665-TTHERM_00967540

29666-TTHERM_00967550

29667-TTHERM_00967560

29668-TTHERM_00967570

interaptin

2474-TTHERM_00245800

2475-TTHERM_00245810

2479-TTHERM_00245850

2483-TTHERM_00245890

2485-TTHERM_00245910

2486-TTHERM_00246910

2488-TTHERM_00246930

2491-TTHERM_00246960

2493-TTHERM_00246980

2497-TTHERM_00247020

2498-TTHERM_00247030

2500-TTHERM_00247050

2501-TTHERM_00247060

2502-TTHERM_00247070

2503-TTHERM_00247080

2515-TTHERM_00247200

2516-TTHERM_00248200

2518-TTHERM_00248220

2519-TTHERM_00248230

2520-TTHERM_00248240

2521-TTHERM_00248250

2522-TTHERM_00248260

2523-TTHERM_00248270

2524-TTHERM_00248280

2525-TTHERM_00248290

2526-TTHERM_00248300

2528-TTHERM_00248320

5068-TTHERM_00617740

5069-TTHERM_00617750

8510-TTHERM_00785840

14749-TTHERM_01188320

15213-TTHERM_01060770

18550-TTHERM_00760650

18553-TTHERM_00760680

18554-TTHERM_00760690

18573-TTHERM_01180980

18574-TTHERM_01181980

18976-TTHERM_00242390

20521-TTHERM_00898160

23060-TTHERM_00105600

23061-TTHERM_00105610

23063-TTHERM_00105630

27444-TTHERM_00354580

27921-TTHERM_00561610

29390-TTHERM_00263250

29392-TTHERM_00263270

29394-TTHERM_00263290

29395-TTHERM_00263300

29469-TTHERM_00265110

29471-TTHERM_00265140

30086-TTHERM_00944080

hypothetical protein

2605-TTHERM_01685280

2815-TTHERM_01438780

2817-TTHERM_01440780

3357-TTHERM_01615780

4505-TTHERM_01378930

5060-TTHERM_01461130

6314-TTHERM_01424450

6997-TTHERM_01156750

6998-TTHERM_01156760

8908-TTHERM_01683250

10885-TTHERM_00717900

10886-TTHERM_00717910

12085-TTHERM_01670170

12227-TTHERM_01263930

12230-TTHERM_01263960

13310-TTHERM_01170560

13336-TTHERM_00359230

13347-TTHERM_00360330

13371-TTHERM_00361560

13374-TTHERM_00361590

14779-TTHERM_01119390

14780-TTHERM_01119400

14781-TTHERM_01119410

14782-TTHERM_01119420

15778-TTHERM_01365630

17269-TTHERM_01709380

17855-TTHERM_01627910

17858-TTHERM_01082840

19025-TTHERM_01319500

19028-TTHERM_01319530

19409-TTHERM_01725470

21147-TTHERM_00777360

21155-TTHERM_00778430

21704-TTHERM_01361490

23086-TTHERM_00106920

23995-TTHERM_01153610

23998-TTHERM_01153640

24009-TTHERM_01155730

24213-TTHERM_01006560

25202-TTHERM_01356330

25205-TTHERM_01356360

26273-TTHERM_00752050

26387-TTHERM_01565120

28067-TTHERM_01410020

28099-TTHERM_01331050

28243-TTHERM_01569160

29103-TTHERM_01445920

29554-TTHERM_01296380

29634-TTHERM_00963270

30140-TTHERM_01148240

histidine kinase DhkL

3209-TTHERM_00537230

4106-TTHERM_00584990

4112-TTHERM_00585050

4145-TTHERM_00585390

4146-TTHERM_00585400

6390-TTHERM_00526520

6570-TTHERM_00630570

6574-TTHERM_00630610

7076-TTHERM_00920790

7183-TTHERM_00420580

7820-TTHERM_00989300

7838-TTHERM_00989480

7982-TTHERM_00499390

7983-TTHERM_00499400

8067-TTHERM_00502280

8442-TTHERM_01401740

10844-TTHERM_00717460

10845-TTHERM_00717470

10868-TTHERM_00717720

12346-TTHERM_00522310

13539-TTHERM_00415680

13760-TTHERM_00202880

14296-TTHERM_00031640

14557-TTHERM_00039220

14574-TTHERM_00040380

15301-TTHERM_01055470

15795-TTHERM_00646890

15969-TTHERM_00320520

16249-TTHERM_00842640

16839-TTHERM_00711790

17200-TTHERM_01002800

17202-TTHERM_01002820

17203-TTHERM_01002830

17211-TTHERM_01002910

17212-TTHERM_01002920

18664-TTHERM_00467620

19275-TTHERM_00299800

19671-TTHERM_00947510

19862-TTHERM_00532040

21169-TTHERM_00922900

22059-TTHERM_00599930

22136-TTHERM_00600730

22236-TTHERM_00216110

22277-TTHERM_00218500

25684-TTHERM_00583470

28479-TTHERM_00160870

28692-TTHERM_00373780

30008-TTHERM_00813020

proprotein convertase

1993-TTHERM_02383080

2073-TTHERM_02598230

5044-TTHERM_01046970

5048-TTHERM_01047010

8453-TTHERM_00826750

9035-TTHERM_00566950

9670-TTHERM_01322650

9674-TTHERM_01322690

9678-TTHERM_01323720

9680-TTHERM_01323740

10036-TTHERM_00075910

10039-TTHERM_00075940

10043-TTHERM_00076970

11502-TTHERM_01405860

13466-TTHERM_00365480

13470-TTHERM_00365520

15146-TTHERM_01013370

15696-TTHERM_00295860

15899-TTHERM_01317360

16182-TTHERM_01718400

21719-TTHERM_01433570

21939-TTHERM_00895570

22169-TTHERM_00901740

22171-TTHERM_00901760

22175-TTHERM_00901800

22182-TTHERM_00903850

22282-TTHERM_00218550

22757-TTHERM_01781750

23717-TTHERM_00911110

23720-TTHERM_00912130

23723-TTHERM_00912160

23725-TTHERM_00912180

23728-TTHERM_00912210

23942-TTHERM_00666900

23988-TTHERM_01547900

25664-TTHERM_00582280

26066-TTHERM_00998870

29290-TTHERM_00258250

29294-TTHERM_00259280

29677-TTHERM_01201170

29686-TTHERM_01204230

29950-TTHERM_00810460

29954-TTHERM_00810500

29957-TTHERM_00810530

29961-TTHERM_00811560

29967-TTHERM_00812610

29969-TTHERM_00812630

hypothetical protein

2288-TTHERM_00474890

2775-TTHERM_00732540

3256-TTHERM_01043230

3742-TTHERM_00938920

3845-TTHERM_00444770

3973-TTHERM_00825090

4333-TTHERM_01142780

5074-TTHERM_00617800

5597-TTHERM_00441970

5667-TTHERM_00442670

7433-TTHERM_00624450

8672-TTHERM_00189220

10852-TTHERM_00717540

11099-TTHERM_00046200

11241-TTHERM_00047620

11376-TTHERM_00051950

11435-TTHERM_00052550

12045-TTHERM_00024320

13612-TTHERM_00418490

14454-TTHERM_00037190

15378-TTHERM_00448590

15820-TTHERM_00647140

15832-TTHERM_00647260

16571-TTHERM_00094160

17907-TTHERM_00933080

18267-TTHERM_00755900

18631-TTHERM_00467290

19090-TTHERM_00716180

19567-TTHERM_00486360

19750-TTHERM_00637470

20449-TTHERM_00823790

20643-TTHERM_00148840

20962-TTHERM_00316510

21227-TTHERM_00304210

21750-TTHERM_00977630

22606-TTHERM_00471980

23004-TTHERM_00105020

24865-TTHERM_00389690

26719-TTHERM_00145110

27215-TTHERM_00121010

27867-TTHERM_00560060

28375-TTHERM_00157850

29730-TTHERM_00170190

Probable serine/threonine-protein kinase

1990-TTHERM_01693300

3066-TTHERM_01582400

4498-TTHERM_01376880

4501-TTHERM_01377900

5174-TTHERM_01086730

5844-TTHERM_01498820

8114-TTHERM_01742570

9595-TTHERM_00815090

11466-TTHERM_01138230

12223-TTHERM_01611750

12644-TTHERM_00236260

13106-TTHERM_00059220

13633-TTHERM_00197650

13816-TTHERM_00209380

14173-TTHERM_01695310

16201-TTHERM_01640980

17699-TTHERM_02293890

18245-TTHERM_01620830

19406-TTHERM_01948320

19991-TTHERM_01388120

19995-TTHERM_01388160

20038-TTHERM_00994420

20891-TTHERM_01414120

21480-TTHERM_01618810

21673-TTHERM_00958760

21676-TTHERM_00958790

21728-TTHERM_00976420

22155-TTHERM_00900600

22632-TTHERM_01656030

22799-TTHERM_00935420

24183-TTHERM_01689280

24609-TTHERM_02013450

25184-TTHERM_01515340

26369-TTHERM_01644000

26374-TTHERM_01663090

26388-TTHERM_01565130

27656-TTHERM_01494740

27999-TTHERM_00633300

28253-TTHERM_01428470

29096-TTHERM_01564120

29524-TTHERM_01504060

29587-TTHERM_01246670

29589-TTHERM_01246690

hypothetical protein

9340-TTHERM_00125780

9341-TTHERM_00125790

9342-TTHERM_00125800

9343-TTHERM_00125810

9344-TTHERM_00125820

9345-TTHERM_00125830

9346-TTHERM_00126830

9347-TTHERM_00126840

9348-TTHERM_00126850

9349-TTHERM_00126860

9350-TTHERM_00126870

9351-TTHERM_00126880

9352-TTHERM_00126890

9353-TTHERM_00126900

9355-TTHERM_00126920

9554-TTHERM_00130030

9557-TTHERM_00130060

9558-TTHERM_00130070

9918-TTHERM_00275770

9921-TTHERM_00275800

9923-TTHERM_00275830

10913-TTHERM_01130780

10914-TTHERM_01130790

10916-TTHERM_01131800

10917-TTHERM_01131810

10918-TTHERM_01131820

10919-TTHERM_01132820

16045-TTHERM_00324390

19799-TTHERM_01224660

19800-TTHERM_01225660

19801-TTHERM_01225670

27788-TTHERM_01050450

27790-TTHERM_01050470

27791-TTHERM_01050480

27792-TTHERM_01050490

27793-TTHERM_01050500

27795-TTHERM_01050520

27801-TTHERM_01050580

27823-TTHERM_01539690

27824-TTHERM_01539700

27825-TTHERM_01539710

ABC transporter AbcG1

4433-TTHERM_00773420

5830-TTHERM_00580360

7447-TTHERM_00624590

12889-TTHERM_00654020

13609-TTHERM_00418430

14309-TTHERM_00031770

14311-TTHERM_00031790

14312-TTHERM_00031800

14313-TTHERM_00031810

14314-TTHERM_00031820

14315-TTHERM_00031830

14320-TTHERM_00032870

14321-TTHERM_00032880

14322-TTHERM_00032890

14324-TTHERM_00033900

14325-TTHERM_00033910

14326-TTHERM_00034910

14327-TTHERM_00034920

14329-TTHERM_00034940

14361-TTHERM_00035270

14362-TTHERM_00035280

14364-TTHERM_00035300

14366-TTHERM_00035320

14367-TTHERM_00035330

14369-TTHERM_00035350

14370-TTHERM_00035360

14372-TTHERM_00035380

14373-TTHERM_00035390

14374-TTHERM_00035400

14376-TTHERM_00035420

14377-TTHERM_00035430

14378-TTHERM_00035440

16889-TTHERM_00713380

17183-TTHERM_01002630

17184-TTHERM_01002640

21402-TTHERM_00794030

21525-TTHERM_00409090

22070-TTHERM_00600070

30039-TTHERM_01128600

hypothetical protein

3931-TTHERM_01434620

3932-TTHERM_01434630

3934-TTHERM_01434650

3935-TTHERM_01434660

3936-TTHERM_01434670

5547-TTHERM_01009890

5717-TTHERM_01372810

5719-TTHERM_01372830

7364-TTHERM_00622770

8186-TTHERM_00758890

14891-TTHERM_00700940

14892-TTHERM_00700950

16599-TTHERM_01349970

17123-TTHERM_01306890

17124-TTHERM_01306900

18139-TTHERM_01400690

18140-TTHERM_01400700

18142-TTHERM_01400720

19974-TTHERM_01406910

19975-TTHERM_01406920

19978-TTHERM_01407940

21998-TTHERM_01051760

25867-TTHERM_00285380

26173-TTHERM_00343940

27139-TTHERM_00113250

30219-TTHERM_00327160

30220-TTHERM_00327170

30221-TTHERM_00327180

30223-TTHERM_00327200

30225-TTHERM_00327220

30226-TTHERM_00327230

30227-TTHERM_00327240

30228-TTHERM_00327250

cytochrome P450 monooxygenase

1727-TTHERM_00754730

3537-TTHERM_00313500

3814-TTHERM_00444460

4881-TTHERM_00283420

5090-TTHERM_00620930

7634-TTHERM_01369770

9540-TTHERM_00129890

10727-TTHERM_00516350

12513-TTHERM_00227020

13688-TTHERM_00198200

13689-TTHERM_00198210

13690-TTHERM_00198220

13691-TTHERM_00198230

13700-TTHERM_00198320

13702-TTHERM_00198340

13725-TTHERM_00200550

13729-TTHERM_00201580

13734-TTHERM_00201630

15468-TTHERM_00449550

16089-TTHERM_01398470

17281-TTHERM_01280630

18075-TTHERM_00723150

18915-TTHERM_00241770

20495-TTHERM_01250020

20537-TTHERM_00898320

22916-TTHERM_00101170

22928-TTHERM_00101290

24401-TTHERM_00137750

24596-TTHERM_01122770

24597-TTHERM_01122780

26335-TTHERM_00754700

26338-TTHERM_01698320

27099-TTHERM_00112850

29883-TTHERM_00185610

hypothetical protein

3931-TTHERM_01434620

3932-TTHERM_01434630

3934-TTHERM_01434650

3935-TTHERM_01434660

3936-TTHERM_01434670

5547-TTHERM_01009890

5717-TTHERM_01372810

5719-TTHERM_01372830

7364-TTHERM_00622770

8186-TTHERM_00758890

14891-TTHERM_00700940

14892-TTHERM_00700950

16599-TTHERM_01349970

17123-TTHERM_01306890

17124-TTHERM_01306900

18139-TTHERM_01400690

18140-TTHERM_01400700

18142-TTHERM_01400720

19974-TTHERM_01406910

19975-TTHERM_01406920

19978-TTHERM_01407940

21998-TTHERM_01051760

25867-TTHERM_00285380

26173-TTHERM_00343940

27139-TTHERM_00113250

30219-TTHERM_00327160

30220-TTHERM_00327170

30221-TTHERM_00327180

30223-TTHERM_00327200

30225-TTHERM_00327220

30226-TTHERM_00327230

30227-TTHERM_00327240

30228-TTHERM_00327250

MORN repeat protein

3128-TTHERM_00535430

3285-TTHERM_00762940

3342-TTHERM_00766490

3988-TTHERM_00825240

4268-TTHERM_00494340

4482-TTHERM_01248870

5936-TTHERM_01084170

6203-TTHERM_00476750

6646-TTHERM_00193630

6793-TTHERM_00196080

8993-TTHERM_00564550

9011-TTHERM_00566710

9050-TTHERM_00857860

10081-TTHERM_00077350

10140-TTHERM_00078940

10304-TTHERM_00378500

12188-TTHERM_00540300

15304-TTHERM_01055500

15823-TTHERM_00647170

16938-TTHERM_01093650

18017-TTHERM_01164130

18207-TTHERM_00685930

19680-TTHERM_00947600

21087-TTHERM_00318860

21838-TTHERM_00724730

22699-TTHERM_00801290

24128-TTHERM_00590130

24269-TTHERM_00133460

24427-TTHERM_00138010

24774-TTHERM_00384820

25138-TTHERM_00940320

27098-TTHERM_00112840

27446-TTHERM_00354600

Hypothetical WD-repeat protein alr3466

2471-TTHERM_00245770

5483-TTHERM_00439260

7085-TTHERM_01422370

7086-TTHERM_01422380

9200-TTHERM_00648860

9208-TTHERM_00648940

10328-TTHERM_00378740

12527-TTHERM_00227170

12567-TTHERM_00227570

12723-TTHERM_00400790

12785-TTHERM_00405380

13306-TTHERM_01170520

14398-TTHERM_00035640

14683-TTHERM_00741670

16098-TTHERM_00680620

17395-TTHERM_00930820

21918-TTHERM_01609750

22624-TTHERM_01351030

23496-TTHERM_00392680

23805-TTHERM_00661600

23999-TTHERM_01154640

24214-TTHERM_01006570

24215-TTHERM_01006580

24216-TTHERM_01006590

24217-TTHERM_01006600

24676-TTHERM_00628500

25854-TTHERM_00285250

26126-TTHERM_00343460

28630-TTHERM_00371140

ATP-binding cassette transporter C4

4097-TTHERM_00584900

4855-TTHERM_00283160

5518-TTHERM_00440640

5520-TTHERM_00440660

5543-TTHERM_01009850

6238-TTHERM_00479080

6569-TTHERM_00630560

6571-TTHERM_00630580

7464-TTHERM_00624760

7508-TTHERM_01194710

8514-TTHERM_00785880

12021-TTHERM_00024080

14732-TTHERM_01358410

16172-TTHERM_00683350

19332-TTHERM_00300370

23750-TTHERM_00916430

23803-TTHERM_00661580

23804-TTHERM_00661590

25052-TTHERM_00592990

25054-TTHERM_00593010

25055-TTHERM_00593020

26059-TTHERM_00998800

27917-TTHERM_00561570

27919-TTHERM_00561590

28576-TTHERM_00161860

28914-TTHERM_00574250

29472-TTHERM_00265150

immobilization antigen LD

2359-TTHERM_01894110

4835-TTHERM_00280970

5188-TTHERM_01087870

5190-TTHERM_01087890

5191-TTHERM_01087900

5192-TTHERM_01087910

5193-TTHERM_01087920

6521-TTHERM_00629990

10679-TTHERM_00506960

15073-TTHERM_00548320

15074-TTHERM_00548330

15075-TTHERM_00548340

15077-TTHERM_00548360

15079-TTHERM_00548380

15406-TTHERM_00448880

16650-TTHERM_02573220

19461-TTHERM_01125110

19462-TTHERM_01125120

19841-TTHERM_00531810

23339-TTHERM_00891260

23341-TTHERM_00891280

23347-TTHERM_00892330

23349-TTHERM_00892350

23352-TTHERM_00894360

23354-TTHERM_00894380

23355-TTHERM_00894390

23772-TTHERM_00660280

interaptin

2453-TTHERM_00245580

2454-TTHERM_00245590

2456-TTHERM_00245610

2460-TTHERM_00245650

2916-TTHERM_00694320

4068-TTHERM_00584600

4525-TTHERM_01106110

7741-TTHERM_00821810

7742-TTHERM_00821820

7743-TTHERM_00821830

11551-TTHERM_00001440

11566-TTHERM_00002580

11570-TTHERM_00002620

11571-TTHERM_00002630

11572-TTHERM_00002640

11577-TTHERM_00002690

11579-TTHERM_00002710

20023-TTHERM_00994270

20025-TTHERM_00994290

20027-TTHERM_00994310

20029-TTHERM_00994330

20030-TTHERM_00994340

22223-TTHERM_00215990

28450-TTHERM_00160580

28451-TTHERM_00160590

hypothetical protein

8157-TTHERM_00757610

8158-TTHERM_00757620

8160-TTHERM_00757640

8162-TTHERM_00757660

8163-TTHERM_00757670

8164-TTHERM_00757680

8166-TTHERM_00757700

8167-TTHERM_00757710

8168-TTHERM_00757720

8207-TTHERM_00759100

8209-TTHERM_00759120

8211-TTHERM_00759140

8214-TTHERM_00759170

11460-TTHERM_02463150

20047-TTHERM_01141420

20048-TTHERM_01141430

20050-TTHERM_01141450

20051-TTHERM_01141460

23362-TTHERM_00894460

23364-TTHERM_00894480

23366-TTHERM_00894500

23368-TTHERM_00894520

24716-TTHERM_01186250

27666-TTHERM_01070360

30114-TTHERM_02471150

interaptin

2804-TTHERM_00732830

2833-TTHERM_01100420

4550-TTHERM_01179810

6912-TTHERM_00497460

8802-TTHERM_00191510

8803-TTHERM_00191520

11787-TTHERM_00011810

13243-TTHERM_00069530

13245-TTHERM_00069550

13246-TTHERM_00069560

13247-TTHERM_00069570

13456-TTHERM_00365380

13460-TTHERM_00365420

13521-TTHERM_00414480

13576-TTHERM_00418070

13900-TTHERM_00769590

13901-TTHERM_00770590

13919-TTHERM_00770770

15534-TTHERM_01519330

16141-TTHERM_00683040

21010-TTHERM_00317100

26112-TTHERM_00343320

27547-TTHERM_00355610

27599-TTHERM_00357130

27868-TTHERM_00560070

glutathione transferase

6541-TTHERM_00630260

6542-TTHERM_00630270

8392-TTHERM_00490870

8393-TTHERM_00490880

8394-TTHERM_00490890

8395-TTHERM_00490900

9902-TTHERM_00274620

10446-TTHERM_00569160

10450-TTHERM_00569200

10451-TTHERM_00569210

12787-TTHERM_00405400

12791-TTHERM_00405440

12792-TTHERM_00405450

12794-TTHERM_00405470

12797-TTHERM_00405500

13830-TTHERM_00211500

13831-TTHERM_00211510

20212-TTHERM_01289030

21957-TTHERM_00895750

21958-TTHERM_00895760

21959-TTHERM_00895770

27338-TTHERM_00602860

27339-TTHERM_00602870

30316-TTHERM_00332090

hypothetical protein

4046-TTHERM_01497800

4966-TTHERM_01312330

4970-TTHERM_01312370

5558-TTHERM_01010000

5559-TTHERM_01010010

5562-TTHERM_01010040

7548-TTHERM_01536640

7723-TTHERM_01081710

7731-TTHERM_01081790

8223-TTHERM_01076840

8225-TTHERM_01076860

8227-TTHERM_01076880

8407-TTHERM_00491020

12918-TTHERM_01486710

12919-TTHERM_01488700

13687-TTHERM_00198190

15176-TTHERM_01292210

17064-TTHERM_01855030

19454-TTHERM_01265060

20204-TTHERM_01585460

28250-TTHERM_01543790

29576-TTHERM_01008720

29578-TTHERM_01008740

29582-TTHERM_01008780

J immobilization antigen

8430-TTHERM_00491250

8431-TTHERM_00491260

8432-TTHERM_00491270

8433-TTHERM_00491280

9202-TTHERM_00648880

9203-TTHERM_00648890

13982-TTHERM_00424480

13984-TTHERM_00424500

19838-TTHERM_01700340

21099-TTHERM_00775860

21100-TTHERM_00775870

21102-TTHERM_00775890

21104-TTHERM_00775910

22815-TTHERM_00935580

24070-TTHERM_00586580

27355-TTHERM_00606990

27357-TTHERM_00607010-

28032-TTHERM_00633630-

28033-TTHERM_00633640-

28035-TTHERM_00634650-

28037-TTHERM_00634670-

28038-TTHERM_00634680-

28040-TTHERM_00635690-

28042-TTHERM_00635710-

hypothetical protein

16711-TTHERM_02477160

27365-TTHERM_00607090

27366-TTHERM_00607100

27368-TTHERM_00607120

27381-TTHERM_00607250

27382-TTHERM_00607260

27385-TTHERM_00607290

27386-TTHERM_00607300

27387-TTHERM_00607310

27389-TTHERM_00609310

27390-TTHERM_00609320

27391-TTHERM_00609330

27392-TTHERM_00609340

27394-TTHERM_00609360

27396-TTHERM_00609380

27398-TTHERM_00609400

27400-TTHERM_00609420

27403-TTHERM_00609450

27404-TTHERM_00609460

27407-TTHERM_00609490

27408-TTHERM_00609500

27412-TTHERM_00610530

27413-TTHERM_00610540

28745-TTHERM_01021930

hypothetical protein (multi-domain)

2142-TTHERM_00657580

5007-TTHERM_00954310

6306-TTHERM_00484720

7409-TTHERM_00624210

10617-TTHERM_00643470

11484-TTHERM_01140390

11486-TTHERM_01140410

12559-TTHERM_00227490

12560-TTHERM_00227500

12561-TTHERM_00227510

12563-TTHERM_00227530

13378-TTHERM_00361630

13379-TTHERM_00361640

18199-TTHERM_00685850

21007-TTHERM_00317070

21184-TTHERM_00923050

22786-TTHERM_01213900

23098-TTHERM_00107040

23450-TTHERM_00391230

25583-TTHERM_00784810

26473-TTHERM_00431320

29766-TTHERM_00170580

29767-TTHERM_00170590

membrane transporter

2419-TTHERM_00245230

2421-TTHERM_00245250

2423-TTHERM_00245270

2424-TTHERM_00245280

2425-TTHERM_00245290

2843-TTHERM_01100520

9120-TTHERM_01207670

9121-TTHERM_01207680

9306-TTHERM_00125440

11039-TTHERM_01284730

11040-TTHERM_01284740

22115-TTHERM_00600520

23251-TTHERM_00798120

23253-TTHERM_00798140

23254-TTHERM_00798150

23255-TTHERM_00798160

23256-TTHERM_00798170

23257-TTHERM_00798180

23258-TTHERM_00799180

23829-TTHERM_00663820

24339-TTHERM_00136140

24343-TTHERM_00136180

26852-TTHERM_00146450

hypothetical protein

6307-TTHERM_00484730

7926-TTHERM_00558290

10819-TTHERM_01029890

10820-TTHERM_01029900

13076-TTHERM_00058910

14227-TTHERM_00029960

14229-TTHERM_00029980

21408-TTHERM_00794090

27304-TTHERM_00123900

27305-TTHERM_00123910

27306-TTHERM_00123920

27307-TTHERM_00123930

27308-TTHERM_00123940

27309-TTHERM_00123950

27310-TTHERM_00123960

27311-TTHERM_00123970

28582-TTHERM_00370660

29339-TTHERM_00260760

29343-TTHERM_00261790

29344-TTHERM_00261800

29346-TTHERM_00261820

29347-TTHERM_00261830

hypothetical protein

1372-TTHERM_00790820

1373-TTHERM_00790830

1569-TTHERM_01298560

2680-TTHERM_01075680

4174-TTHERM_00492380

5354-TTHERM_00353380

5355-TTHERM_00353410

6627-TTHERM_00193440

7344-TTHERM_01245600

11609-TTHERM_00006010

13231-TTHERM_00069410

16003-TTHERM_00322840

18684-TTHERM_00467830

19292-TTHERM_00299970

19514-TTHERM_00485820

23815-TTHERM_00661700

24094-TTHERM_00586820

25462-TTHERM_00735380

25463-TTHERM_00735390

26837-TTHERM_00146300

28504-TTHERM_00161120

28644-TTHERM_00371280

hypothetical protein

15279-TTHERM_00927260

15280-TTHERM_00927270

15282-TTHERM_00927290

15283-TTHERM_00927300

15284-TTHERM_00927310

15286-TTHERM_00927330

15287-TTHERM_00927340

15288-TTHERM_00927350

15289-TTHERM_00927360

15290-TTHERM_00927370

15291-TTHERM_00927380

15292-TTHERM_00927390

15293-TTHERM_00927400

17989-TTHERM_01027600

17991-TTHERM_01027620

17992-TTHERM_01027630

17993-TTHERM_01027640

20624-TTHERM_00147660

20625-TTHERM_00147670

20626-TTHERM_00147680

20627-TTHERM_00148680

21397-TTHERM_00793980

liver stage antigen

1316-TTHERM_00781040

3864-TTHERM_00445950

6933-TTHERM_00497680

7116-TTHERM_00419910

9374-TTHERM_00127110

11192-TTHERM_00047130

11357-TTHERM_00051760

11940-TTHERM_00016340

11979-TTHERM_00019700

12313-TTHERM_00521980

12759-TTHERM_00402150

15736-TTHERM_01000170

18009-TTHERM_01164050

20256-TTHERM_00455270

20376-TTHERM_00459450

20579-TTHERM_01099090

22931-TTHERM_00101320

25603-TTHERM_00581650

25829-TTHERM_00463770

26475-TTHERM_00431340

27926-TTHERM_00561660

28522-TTHERM_00161300

GP63-4 protein

4291-TTHERM_00494630

9043-TTHERM_00857790

9384-TTHERM_00127210

18490-TTHERM_00721820

20610-TTHERM_00147520

25973-TTHERM_00289410

25974-TTHERM_00289430

25982-TTHERM_00289510

25983-TTHERM_00289520

25984-TTHERM_00289530

25985-TTHERM_00289540

25986-TTHERM_00289550

25987-TTHERM_00289560

25988-TTHERM_00289570

25990-TTHERM_00289590

25991-TTHERM_00289600

25992-TTHERM_00289610

25993-TTHERM_00290610

25995-TTHERM_00290630

26990-TTHERM_00942690

27090-TTHERM_00112760

27324-TTHERM_02182710

interaptin

1199-TTHERM_00853000

1201-TTHERM_00853020

1204-TTHERM_00853050

1761-TTHERM_01243500

3477-TTHERM_00312900

5954-TTHERM_01084360

7168-TTHERM_00420430

8794-TTHERM_00191430

15215-TTHERM_01060790

16165-TTHERM_00683280

18090-TTHERM_00723300

19197-TTHERM_00616480

19338-TTHERM_00300430

21245-TTHERM_00305390

27192-TTHERM_00118760

27193-TTHERM_00118770

27194-TTHERM_00118780

27195-TTHERM_00119780

27196-TTHERM_00120780

27197-TTHERM_00120790

28626-TTHERM_00371100

proprotein convertase aPC6C isoform

1885-TTHERM_01104930

3455-TTHERM_00312680

5754-TTHERM_00578610

6602-TTHERM_00193190

10639-TTHERM_00644680

16805-TTHERM_02146650

19787-TTHERM_00637840

21161-TTHERM_00921830

21162-TTHERM_00922830

21163-TTHERM_00922840

23858-TTHERM_01597630

28640-TTHERM_00371240

29202-TTHERM_00550950

29207-TTHERM_00551030

30088-TTHERM_00944100

30090-TTHERM_00944120

30091-TTHERM_00944130

30092-TTHERM_00944140

30094-TTHERM_00944160

30095-TTHERM_00945160

30096-TTHERM_00945170

hypothetical protein -

4047-TTHERM_01497810

4965-TTHERM_01312320

4968-TTHERM_01312350

5560-TTHERM_01010020

5563-TTHERM_01010050

5568-TTHERM_01081830

7352-TTHERM_00622650

7549-TTHERM_01536650

7722-TTHERM_01081700

7730-TTHERM_01081780

8224-TTHERM_01076850

8226-TTHERM_01076870

8228-TTHERM_01076890

8406-TTHERM_00491010

12920-TTHERM_01488710

15175-TTHERM_01292200

20599-TTHERM_01099290

29575-TTHERM_01008710

29581-TTHERM_01008770

Putative triacylglycerol lipase -

1972-TTHERM_01092360

3000-TTHERM_00691620

4346-TTHERM_00925380

5334-TTHERM_00352190

6681-TTHERM_00193970

6742-TTHERM_00194580

6743-TTHERM_00194590

7257-TTHERM_00727560

7410-TTHERM_00624220

9529-TTHERM_00129780

10880-TTHERM_00717850

11574-TTHERM_00002660

15930-TTHERM_00320120

15931-TTHERM_00320130

15933-TTHERM_00320160

15937-TTHERM_00320200

15940-TTHERM_00320230

18201-TTHERM_00685870

23946-TTHERM_00666940

26560-TTHERM_00434190

K+ channel, beta subunit-

2077-TTHERM_01080350

2078-TTHERM_01080360

2079-TTHERM_01080370

6061-TTHERM_00338200

17180-TTHERM_01002600

19233-TTHERM_00298310

19357-TTHERM_00300620

19358-TTHERM_00300630

19359-TTHERM_00300640

20164-TTHERM_00703880

21528-TTHERM_00409120

23688-TTHERM_00962000

23690-TTHERM_00962020

23692-TTHERM_00962040

23693-TTHERM_00962050

23694-TTHERM_00962060

23695-TTHERM_00962070

23697-TTHERM_00962090

29624-TTHERM_01311290

hypothetical protein-

6114-TTHERM_00339730

6115-TTHERM_00339740

6116-TTHERM_00339750

6117-TTHERM_00339760

8629-TTHERM_00188790

14924-TTHERM_00702260

15099-TTHERM_00549580

15801-TTHERM_00646950

18603-TTHERM_00465000

18604-TTHERM_00465010

23343-TTHERM_00891300

27470-TTHERM_00354840

29003-TTHERM_00577160

29004-TTHERM_00577170

29006-TTHERM_00577190

29007-TTHERM_00577200

29008-TTHERM_00577210

29009-TTHERM_00577220

hypothetical protein-

4436-TTHERM_00773450

4437-TTHERM_00773460

7556-TTHERM_00676930

10028-TTHERM_00075830

10766-TTHERM_00518720

13279-TTHERM_00070890

13590-TTHERM_00418210

16925-TTHERM_01093520

16997-TTHERM_01260740

16998-TTHERM_01260750

19039-TTHERM_00715650

19724-TTHERM_00637210

20852-TTHERM_00151940

21304-TTHERM_00309990

21649-TTHERM_00957530

25821-TTHERM_00463690

25869-TTHERM_00285400

30388-TTHERM_01444900

unknown-

11547-TTHERM_00001400

11549-TTHERM_00001420

11550-TTHERM_00001430

13879-TTHERM_01234340

17597-TTHERM_02533180

18223-TTHERM_00686090

21775-TTHERM_01606700

21934-TTHERM_01476530

25139-TTHERM_00940330

26406-TTHERM_00429650

26922-TTHERM_00709620

27885-TTHERM_00561240

27886-TTHERM_00561250

27887-TTHERM_00561260

27888-TTHERM_00561270

29283-TTHERM_01478570

30003-TTHERM_00812970

AtABCA1 -

2737-TTHERM_01417320

2911-TTHERM_00693280

2912-TTHERM_00693290

2913-TTHERM_00693300

2914-TTHERM_00694300

2915-TTHERM_00694310

6219-TTHERM_00476910

6227-TTHERM_00476990

7051-TTHERM_00919550

7052-TTHERM_00919560

11753-TTHERM_00011470

13817-TTHERM_00209390

19382-TTHERM_00301860

19383-TTHERM_00301870

19935-TTHERM_00532790

20535-TTHERM_00898300

24453-TTHERM_00138260

solute carrier family 22 member 5 -

4494-TTHERM_01248990

4950-TTHERM_00284120

5425-TTHERM_00437690

5432-TTHERM_00437760

5433-TTHERM_00437770

7609-TTHERM_00678450

8475-TTHERM_00826980

8716-TTHERM_00190650

8845-TTHERM_00191950

8846-TTHERM_00191960

12487-TTHERM_00225770

15118-TTHERM_01012100

16933-TTHERM_01093600

23550-TTHERM_00393270

24469-TTHERM_00138420

25886-TTHERM_00285570

27458-TTHERM_00354720

Bile pigment transporter 1-

5861-TTHERM_00951710

5863-TTHERM_00951730

5867-TTHERM_00951770

5870-TTHERM_00951800

6356-TTHERM_00526180

12359-TTHERM_00522440

13506-TTHERM_00414290

14840-TTHERM_01227870

15921-TTHERM_00320020

15922-TTHERM_00320030

17875-TTHERM_01083010

26187-TTHERM_00344080

26201-TTHERM_00344220

26221-TTHERM_00346400

26252-TTHERM_00346710

26997-TTHERM_00942770

29642-TTHERM_00964340

long chain acyl-CoA synthetase 7 -

3058-TTHERM_01124100

5324-TTHERM_00349120

7600-TTHERM_00678360

7601-TTHERM_00678370

10395-TTHERM_00382380

11451-TTHERM_00052710

15237-TTHERM_01066950

16278-TTHERM_00083310

20498-TTHERM_01250050

27116-TTHERM_00113020

28711-TTHERM_00374970

28715-TTHERM_00375010

29936-TTHERM_00187190

29938-TTHERM_00187210

29940-TTHERM_00187230

29944-TTHERM_00187270

hypothetical protein-

13833-TTHERM_00211530

13834-TTHERM_00211540

13835-TTHERM_00212540

15932-TTHERM_00320150

26208-TTHERM_00344290

26209-TTHERM_00344300

26210-TTHERM_00344310

26211-TTHERM_00344320

26212-TTHERM_00344330

26213-TTHERM_00344340

26214-TTHERM_00344350

26215-TTHERM_00344360

26217-TTHERM_00344380

26218-TTHERM_00344390

26219-TTHERM_00345390

26220-TTHERM_00346390

hypothetical protein-

6600-TTHERM_00193170

7009-TTHERM_01156870

12210-TTHERM_01335260

13884-TTHERM_01237360

14985-TTHERM_00875850

16544-TTHERM_00092900

16784-TTHERM_02162690

21922-TTHERM_01583440

23375-TTHERM_00805810

23666-TTHERM_00959800

24090-TTHERM_00586780

27670-TTHERM_01071390

28207-TTHERM_02222810

28747-TTHERM_01021950

30395-TTHERM_01594600

hypothetical protein-

3405-TTHERM_00312170

13396-TTHERM_00361810

16325-TTHERM_00083780

16328-TTHERM_00083810

16329-TTHERM_00083820

16330-TTHERM_00083830

16331-TTHERM_00083840

16332-TTHERM_00083850

16333-TTHERM_00083860

16334-TTHERM_00083870

16335-TTHERM_00083880

16338-TTHERM_00083910

16340-TTHERM_00083930

16341-TTHERM_00083940

29994-TTHERM_00812880

hypothetical protein-

1499-TTHERM_01394350

1736-TTHERM_01462150

4465-TTHERM_00773740

6834-TTHERM_00196490

7805-TTHERM_01117290

8896-TTHERM_01251230

9129-TTHERM_01309060

10204-TTHERM_00079590

19948-TTHERM_00533910

20494-TTHERM_01250010

20504-TTHERM_01250110

20506-TTHERM_01250130

22978-TTHERM_00102780

25266-TTHERM_00249860

28084-TTHERM_01107350

TPR domain/sulfotransferase domain protein -

2139-TTHERM_00657550

2178-TTHERM_00658930

7896-TTHERM_00557980

8628-TTHERM_00188780

9596-TTHERM_00815100

9597-TTHERM_00815110

10244-TTHERM_00079990

11820-TTHERM_00013140

11971-TTHERM_00019620

12298-TTHERM_00856770

14171-TTHERM_00833880

23384-TTHERM_00806890

23480-TTHERM_00391530

24175-TTHERM_00591600

26843-TTHERM_00146360

hypothetical protein LOC340351 -

3643-TTHERM_00655450

5794-TTHERM_00579010

6465-TTHERM_00527330

7286-TTHERM_00727850

8130-TTHERM_01068150

9020-TTHERM_00566800

13894-TTHERM_00767550

16134-TTHERM_00682970

16281-TTHERM_00083340

17365-TTHERM_00929530

19094-TTHERM_00716220

20975-TTHERM_00316640

29218-TTHERM_00551140

29906-TTHERM_00185840

30315-TTHERM_00332080

P-type ATPase -

2406-TTHERM_00245100

7213-TTHERM_00420880

7215-TTHERM_00420900

8876-TTHERM_01149330

8879-TTHERM_01150350

9853-TTHERM_00268190

10625-TTHERM_00643550

10682-TTHERM_00508970

10684-TTHERM_00508990

11282-TTHERM_00049030

13143-TTHERM_00061570

13457-TTHERM_00365390

13463-TTHERM_00365450

14887-TTHERM_00700900

27566-TTHERM_00355800

carboxypeptidase-like protein-

15272-TTHERM_00927190

15273-TTHERM_00927200

15274-TTHERM_00927210

15275-TTHERM_00927220

15410-TTHERM_00448920

15411-TTHERM_00448930

15416-TTHERM_00448980

20066-TTHERM_01141610

25236-TTHERM_00249550

25237-TTHERM_00249560

25238-TTHERM_00249570

25249-TTHERM_00249680

25252-TTHERM_00249710

25261-TTHERM_00249800

26265-TTHERM_00346900

rac-beta serine/threonine protein kinase, putative -

3020-TTHERM_00691820

4011-TTHERM_00825470

6284-TTHERM_00483500

9468-TTHERM_00129040

10726-TTHERM_00516340

14576-TTHERM_00040400

18079-TTHERM_00723190

19190-TTHERM_00616410

19624-TTHERM_00486930

20748-TTHERM_00149880

21909-TTHERM_00726440

23891-TTHERM_00666380

24302-TTHERM_00134780

29158-TTHERM_00865280

Proprotein convertase-

2729-TTHERM_01205390

15830-TTHERM_00647240

17676-TTHERM_01049180

18066-TTHERM_00723060-

18744-TTHERM_01326800-

22247-TTHERM_00217210-

22249-TTHERM_00218220-

23337-TTHERM_00891240-

23587-TTHERM_00394630-

23642-TTHERM_01197000-

23643-TTHERM_01197010-

28866-TTHERM_01215010-

28868-TTHERM_01215030-

29327-TTHERM_00259650-

hypothetical protein -

3246-TTHERM_01043130

3247-TTHERM_01043140

3248-TTHERM_01043150

8016-TTHERM_00499730

8017-TTHERM_00500730

9548-TTHERM_00129970

23819-TTHERM_00661740

23821-TTHERM_00662750

23822-TTHERM_00662760

23823-TTHERM_00663760

23825-TTHERM_00663780

23826-TTHERM_00663790

23827-TTHERM_00663800

23828-TTHERM_00663810

hypothetical protein -

25513-TTHERM_00782110

25514-TTHERM_00782120

25516-TTHERM_00782140

25517-TTHERM_00782150

25518-TTHERM_00782160

25519-TTHERM_00782170

25520-TTHERM_00782180

25522-TTHERM_00783190

25524-TTHERM_00783210

25525-TTHERM_00783220

25528-TTHERM_00784240

25529-TTHERM_00784250

25530-TTHERM_00784260

25531-TTHERM_00784270

hypothetical protein -

2606-TTHERM_01686280

2813-TTHERM_01437770

2816-TTHERM_01439780

4504-TTHERM_01378920

4513-TTHERM_01612750

12084-TTHERM_01670160

13312-TTHERM_01171570

13313-TTHERM_01171580

14778-TTHERM_01119380

16743-TTHERM_02247830

17268-TTHERM_01709370

18149-TTHERM_01562110

23997-TTHERM_01153630

29633-TTHERM_00963260

LD24679p-

6140-TTHERM_00339990

6176-TTHERM_00476470

6367-TTHERM_00526290

6368-TTHERM_00526300

6369-TTHERM_00526310

6371-TTHERM_00526330

6372-TTHERM_00526340

6934-TTHERM_00497690

9767-TTHERM_00266330

9890-TTHERM_00274500

9892-TTHERM_00274520

16596-TTHERM_01348950

19673-TTHERM_00947530

21995-TTHERM_01051730

hypothetical protein-

1005-TTHERM_01876070

1524-TTHERM_01918160

2052-TTHERM_01862040

5894-TTHERM_01719430

6594-TTHERM_01825940

7082-TTHERM_02164690

7699-TTHERM_02310920

16690-TTHERM_02451140

16790-TTHERM_02374060

17223-TTHERM_01877070

17766-TTHERM_01989400

22452-TTHERM_01878070

25488-TTHERM_01720430

27441-TTHERM_01857040

hypothetical protein-

2930-TTHERM_00694470

12039-TTHERM_00024260

13244-TTHERM_00069540

14531-TTHERM_00038960

14532-TTHERM_00038970

16250-TTHERM_00842650

16583-TTHERM_00094280

20868-TTHERM_00152100

23651-TTHERM_01197090

25537-TTHERM_00784330

27918-TTHERM_00561580

28472-TTHERM_00160800

28473-TTHERM_00160810

28474-TTHERM_00160820

hypothetical protein-

1024-TTHERM_01672190

1387-TTHERM_00790970

1456-TTHERM_00971860

1607-TTHERM_01538660

2005-TTHERM_01708370

2028-TTHERM_01527450

2032-TTHERM_01527490

13869-TTHERM_00214860

15784-TTHERM_00645790

17606-TTHERM_01473480

25118-TTHERM_00596620

25125-TTHERM_00598670

26402-TTHERM_00429610

26569-TTHERM_01533570

probable Zinc-binding dehydrogenases -

6904-TTHERM_00497380

18876-TTHERM_00239380

20825-TTHERM_00151670

21034-TTHERM_00317340

26712-TTHERM_00145040

26713-TTHERM_00145050

26714-TTHERM_00145060

26715-TTHERM_00145070

26716-TTHERM_00145080

26717-TTHERM_00145090

26729-TTHERM_00145210

26730-TTHERM_00145220

28655-TTHERM_00372410

hypothetical protein-

23870-TTHERM_00666170

23871-TTHERM_00666180

25630-TTHERM_00581920

25631-TTHERM_00581930

25632-TTHERM_00581940

25633-TTHERM_00581950

25636-TTHERM_00581980

25637-TTHERM_00581990

25638-TTHERM_00582000

25639-TTHERM_00582010

25640-TTHERM_00582020

25641-TTHERM_00582030

25642-TTHERM_00582040

hypothetical protein-

18494-TTHERM_00721880

18495-TTHERM_00721890

18496-TTHERM_00721900

18497-TTHERM_00721910

24967-TTHERM_00996430

24968-TTHERM_00996440

24969-TTHERM_00996450

24970-TTHERM_00997450

24972-TTHERM_00997470

24973-TTHERM_00997480

24974-TTHERM_00997490

28915-TTHERM_00574260

28916-TTHERM_00574270

glutathione S-transferase -

9528-TTHERM_00129770

10734-TTHERM_00516420

10736-TTHERM_00516440

10741-TTHERM_00518470

10975-TTHERM_00672290

23802-TTHERM_00661570

23807-TTHERM_00661620

23808-TTHERM_00661630

23809-TTHERM_00661640

23810-TTHERM_00661650

23811-TTHERM_00661660

29717-TTHERM_00166100

29720-TTHERM_00169100

hypothetical protein-

2132-TTHERM_00657480

2379-TTHERM_00243840

2380-TTHERM_00243850

2381-TTHERM_00243860

2383-TTHERM_00243880

18306-TTHERM_00756430

18983-TTHERM_00242460

19177-TTHERM_00616280

24281-TTHERM_00133580

25975-TTHERM_00289440

26352-TTHERM_01159990

28366-TTHERM_00156770

28531-TTHERM_00161410

cell surface immobilization antigen-

7962-TTHERM_00558660

8355-TTHERM_00489510

16022-TTHERM_00323030

27344-TTHERM_00602920

27345-TTHERM_00602930

27351-TTHERM_00606950

27352-TTHERM_00606960

29745-TTHERM_00170340

29746-TTHERM_00170360

29750-TTHERM_00170420

29751-TTHERM_00170430

29752-TTHERM_00170440

29874-TTHERM_00185520

hypothetical protein-

1345-TTHERM_00790550

5401-TTHERM_00437450

5402-TTHERM_00437460

5403-TTHERM_00437470

5404-TTHERM_00437480

5405-TTHERM_00437490

5406-TTHERM_00437500

8766-TTHERM_00191150

10534-TTHERM_00572040

11496-TTHERM_02444130

19942-TTHERM_00532860

23730-TTHERM_00912230

26051-TTHERM_00292190

hypothetical protein-

1873-TTHERM_01103820

1876-TTHERM_01104840

1877-TTHERM_01104850

1878-TTHERM_01104860

1879-TTHERM_01104870

23861-TTHERM_00666080

23862-TTHERM_00666090

23863-TTHERM_00666100

23864-TTHERM_00666110

23865-TTHERM_00666120

23866-TTHERM_00666130

23868-TTHERM_00666150

23869-TTHERM_00666160

lysosomal acid phosphatase -

4649-TTHERM_00529800

4954-TTHERM_00284180

5281-TTHERM_00348690

9236-TTHERM_00649220

11169-TTHERM_00046900

12989-TTHERM_00056060

13533-TTHERM_00415620

14646-TTHERM_00885820

17397-TTHERM_00930840

17398-TTHERM_00930850

18037-TTHERM_01193550

24995-TTHERM_00997700

26216-TTHERM_00344370

hypothetical protein -

6945-TTHERM_00497800

11721-TTHERM_00011150

11722-TTHERM_00011160

12902-TTHERM_00654150

13116-TTHERM_00059320

16850-TTHERM_00711900

17300-TTHERM_01220360

19246-TTHERM_00298440

19466-TTHERM_01125160

22654-TTHERM_01031130

27175-TTHERM_00117570

28575-TTHERM_00161850

29850-TTHERM_00185280

unconventional myosin heavy chain-

6689-TTHERM_00194050

9585-TTHERM_01035740

9972-TTHERM_00074290

11126-TTHERM_00046470

13089-TTHERM_00059050

13970-TTHERM_00423370

15526-TTHERM_00454090

22140-TTHERM_00600770

22141-TTHERM_00600780

25768-TTHERM_00463160

27057-TTHERM_00112430

30235-TTHERM_00327320

TPR repeat -

7000-TTHERM_01156780

7001-TTHERM_01156790

10746-TTHERM_00518520

12290-TTHERM_00856650

15917-TTHERM_00319980

16906-TTHERM_00713550

16913-TTHERM_00713620

18205-TTHERM_00685910

18399-TTHERM_01229030

21153-TTHERM_00778410

27335-TTHERM_00601840

29129-TTHERM_00864990

kazal-type proteinase inhibitor 1 -

20419-TTHERM_00823450

20420-TTHERM_00823460

20421-TTHERM_00823470

20422-TTHERM_00823480

20423-TTHERM_00823490

20424-TTHERM_00823500

20425-TTHERM_00823510

24628-TTHERM_00627030

24630-TTHERM_00627050

24632-TTHERM_00627070

24633-TTHERM_00627080

25085-TTHERM_00594310

interaptin-

2418-TTHERM_00245220

5314-TTHERM_00349020

6362-TTHERM_00526240

16023-TTHERM_00323040

16567-TTHERM_00094120

22065-TTHERM_00599990

23579-TTHERM_00394550

25864-TTHERM_00285350

29179-TTHERM_00550710

29317-TTHERM_00259550

29318-TTHERM_00259560

29323-TTHERM_00259610

hypothetical protein-

4836-TTHERM_00280980

5389-TTHERM_00437330

5391-TTHERM_00437350

6907-TTHERM_00497410

9196-TTHERM_00648820

9197-TTHERM_00648830

9198-TTHERM_00648840

9199-TTHERM_00648850

13845-TTHERM_00213630

13846-TTHERM_00213640

13853-TTHERM_00214700

15935-TTHERM_00320180

MGC68953 protein -

8377-TTHERM_00490720

9532-TTHERM_00129810

12767-TTHERM_00404210

13756-TTHERM_00202840

17080-TTHERM_01512220

23419-TTHERM_00809240

23422-TTHERM_00809270

23425-TTHERM_00809300

23426-TTHERM_00809310

23429-TTHERM_00809340

25855-TTHERM_00285260

26206-TTHERM_00344270

hypothetical protein -

10290-TTHERM_00377370

10292-TTHERM_00377390

10293-TTHERM_00377400

10295-TTHERM_00378410

10297-TTHERM_00378430

10299-TTHERM_00378450

10300-TTHERM_00378460

10301-TTHERM_00378470

23473-TTHERM_00391460

23474-TTHERM_00391470

23476-TTHERM_00391490

29552-TTHERM_01711380

hypothetical protein -

7021-TTHERM_01416280

9547-TTHERM_00129960

9549-TTHERM_00129980

10397-TTHERM_00382400

12015-TTHERM_00024020

14979-TTHERM_00874800

19142-TTHERM_00615930

20396-TTHERM_01108650

20398-TTHERM_01108670

20400-TTHERM_01108690

24732-TTHERM_01101620

24735-TTHERM_01102640

ATP-dependent DNA helicase -

2748-TTHERM_00730290

2749-TTHERM_00730300

8358-TTHERM_00490530

8359-TTHERM_00490540

8361-TTHERM_00490560

9428-TTHERM_00128640

9429-TTHERM_00128650

10989-TTHERM_00673500

10990-TTHERM_00674500

14123-TTHERM_00829440

23316-TTHERM_00888060

29119-TTHERM_00864890

interaptin-

7330-TTHERM_01247780

7331-TTHERM_01247790

7332-TTHERM_01247800

7333-TTHERM_01247810

7334-TTHERM_01247820

7335-TTHERM_01247830

20544-TTHERM_00899380

28749-TTHERM_01021970

28753-TTHERM_01023000

28755-TTHERM_01023020

28757-TTHERM_01023040

28759-TTHERM_01023060

Chitin synthase 1 -

5611-TTHERM_00442110

7770-TTHERM_00822150

11616-TTHERM_00006080

11770-TTHERM_00011640

22060-TTHERM_00599940

24314-TTHERM_00134900

24315-TTHERM_00134910

27117-TTHERM_00113030

27118-TTHERM_00113040

27119-TTHERM_00113050

27120-TTHERM_00113060

27123-TTHERM_00113090

putative equilibrative nucleoside transporter-

1448-TTHERM_00971780

5038-TTHERM_01046910

12016-TTHERM_00024030

20605-TTHERM_00147470

22206-TTHERM_00907060

22207-TTHERM_00907070

22443-TTHERM_00221150

25431-TTHERM_00734080

25823-TTHERM_00463710

27911-TTHERM_00561500

28333-TTHERM_00155430

28334-TTHERM_00155440

MORN repeat protein-

4100-TTHERM_00584930

5921-TTHERM_01044720

6067-TTHERM_00338260

10582-TTHERM_00641130

11228-TTHERM_00047490

19713-TTHERM_00637100

19714-TTHERM_00637110

19715-TTHERM_00637120

19717-TTHERM_00637140

27254-TTHERM_00122390

27557-TTHERM_00355710

30291-TTHERM_00329860

hypothetical protein-

1156-TTHERM_01362560

6587-TTHERM_00630740

15353-TTHERM_01463220

16971-TTHERM_01605680

20087-TTHERM_01929220

23669-TTHERM_00959830

25482-TTHERM_00737560

27691-TTHERM_01074570

27765-TTHERM_01018480

28054-TTHERM_00635830

hypothetical protein-

5386-TTHERM_00437300

9414-TTHERM_00128500

9420-TTHERM_00128560

9423-TTHERM_00128590

12699-TTHERM_00399560

12701-TTHERM_00399580

12705-TTHERM_00399620

12709-TTHERM_00399660

12712-TTHERM_00399690

12714-TTHERM_00399710

12718-TTHERM_00400740

12720-TTHERM_00400760

interaptin-

1753-TTHERM_01243420

1791-TTHERM_01037790

9608-TTHERM_00816230

9609-TTHERM_00816240

9610-TTHERM_00816250

12930-TTHERM_01697320

14756-TTHERM_01188390

20894-TTHERM_01414150

22204-TTHERM_00905060

25437-TTHERM_00734140

25440-TTHERM_00735160

hypothetical protein-

1216-TTHERM_00853170

2093-TTHERM_01080510

4196-TTHERM_00492600

4198-TTHERM_00492620

7584-TTHERM_00678200

7585-TTHERM_00678210

10399-TTHERM_00382420

19236-TTHERM_00298340

20366-TTHERM_00459350

23276-TTHERM_00985060

23277-TTHERM_00985070

Probable cation-transporting ATPase-

7990-TTHERM_00499470

18435-TTHERM_00721270

18737-TTHERM_00469350

22075-TTHERM_00600120

23742-TTHERM_00913380

23743-TTHERM_00913390

25251-TTHERM_00249700

25253-TTHERM_00249720

25257-TTHERM_00249760

25260-TTHERM_00249790

25262-TTHERM_00249810

serine/threonine-protein kinase Nek1, putative -

3630-TTHERM_00655320

15445-TTHERM_00449320

15446-TTHERM_00449330

15448-TTHERM_00449350

15449-TTHERM_00449360

15450-TTHERM_00449370

15451-TTHERM_00449380

15452-TTHERM_00449390

15453-TTHERM_00449400

19066-TTHERM_00715940

21061-TTHERM_00318600

hypothetical protein-

1874-TTHERM_01104820

2323-TTHERM_00475240

2324-TTHERM_00475250

2326-TTHERM_00475270

2327-TTHERM_00475280

2328-TTHERM_00475290

15016-TTHERM_00543680

15019-TTHERM_00543710

15020-TTHERM_00543720

19872-TTHERM_00532140

21743-TTHERM_00977560

hypothetical protein-

2684-TTHERM_01075720

2687-TTHERM_01075750

7715-TTHERM_01081630

8332-TTHERM_00488290

13683-TTHERM_00198150

14702-TTHERM_00746850

18411-TTHERM_01230170

18412-TTHERM_01230180

26200-TTHERM_00344210

29558-TTHERM_01297410

29571-TTHERM_01008670

hypothetical protein-

8400-TTHERM_00490950

8401-TTHERM_00490960

8402-TTHERM_00490970

8403-TTHERM_00490980

8405-TTHERM_00491000

22414-TTHERM_00220860

22415-TTHERM_00220870

22416-TTHERM_00220880

22417-TTHERM_00220890

22418-TTHERM_00220900

22419-TTHERM_00220910

hypothetical protein-

3812-TTHERM_00444440

7664-TTHERM_00861570

7665-TTHERM_00861580

7667-TTHERM_00861600

7668-TTHERM_00861610

7670-TTHERM_00861630

7671-TTHERM_00861640

7672-TTHERM_00861650

7673-TTHERM_00861660

15088-TTHERM_00549460

Probable cation-transporting ATPase-

13738-TTHERM_00201670

13743-TTHERM_00201720

16005-TTHERM_00322860

21194-TTHERM_00923150

21196-TTHERM_00923170

23916-TTHERM_00666630

26761-TTHERM_00145530

28937-TTHERM_00575470

28938-TTHERM_00575480

28941-TTHERM_00575510

29468-TTHERM_00265100

immobilization antigen LA -

13409-TTHERM_00361940

13413-TTHERM_00362970

13415-TTHERM_00362990

13416-TTHERM_00363000

13417-TTHERM_00363010

13418-TTHERM_00363020

13419-TTHERM_00363030

13420-TTHERM_00363040

13421-TTHERM_00363050

13422-TTHERM_00363060

13423-TTHERM_00363070

Tap1p-

3207-TTHERM_00537210

3208-TTHERM_00537220

3787-TTHERM_00444190

7624-TTHERM_00678600

12804-TTHERM_00405570

12806-TTHERM_00405590

13617-TTHERM_00418540

14750-TTHERM_01188330

14751-TTHERM_01188340

29796-TTHERM_00171870

30180-TTHERM_00326770

Potential phospholipid-transporting ATPase IA -

3040-TTHERM_01123860

15586-TTHERM_00294760

17831-TTHERM_00840160

19038-TTHERM_00715640

21581-TTHERM_00411630

21697-TTHERM_01359440

21759-TTHERM_00977720

22094-TTHERM_00600310

23235-TTHERM_00797960

23236-TTHERM_00797970

30159-TTHERM_00325570

unknown protein -

4114-TTHERM_00585070

4115-TTHERM_00585080

4116-TTHERM_00585090

7185-TTHERM_00420600

11414-TTHERM_00052340

15417-TTHERM_00448990

24078-TTHERM_00586660

24079-TTHERM_00586670

24080-TTHERM_00586680

24085-TTHERM_00586730

27612-TTHERM_01004950

FLJ00180 protein -

12233-TTHERM_01263990

13323-TTHERM_01174650

13367-TTHERM_00361520

15779-TTHERM_01365640

23667-TTHERM_00959810

24005-TTHERM_01155690

24007-TTHERM_01155710

25503-TTHERM_01211820

25504-TTHERM_01211840

28064-TTHERM_01409990

cell surface immobilization antigen-

5534-TTHERM_01431570

21013-TTHERM_00317130

21809-TTHERM_01135070

21810-TTHERM_01135080

21813-TTHERM_01135110

21820-TTHERM_01137160

21822-TTHERM_01137180

25418-TTHERM_00733950

28779-TTHERM_01026160

28780-TTHERM_01026170

calcium-dependent protein kinase-a -

2016-TTHERM_01333220

2623-TTHERM_01015890

2675-TTHERM_01075630

5812-TTHERM_00579190

8052-TTHERM_00502130

10025-TTHERM_00075800

16214-TTHERM_00841290

19915-TTHERM_00532590

22258-TTHERM_00218310

28485-TTHERM_00160930

hypothetical protein -

9369-TTHERM_00127060

11684-TTHERM_00008740

12476-TTHERM_00225660

12477-TTHERM_00225670

12478-TTHERM_00225680

12479-TTHERM_00225690

12480-TTHERM_00225700

12481-TTHERM_00225710

21471-TTHERM_01514310

22146-TTHERM_00600830

O-linked GlcNAc transferase -

3427-TTHERM_00312400

3429-TTHERM_00312420

3433-TTHERM_00312460

3434-TTHERM_00312470

5431-TTHERM_00437750

9747-TTHERM_00881470

14767-TTHERM_01192460

14768-TTHERM_01192470

17511-TTHERM_00697150

19950-TTHERM_00533930

serine proteinase inhibitor -

2992-TTHERM_00691540

2993-TTHERM_00691550

3382-TTHERM_00311930

5001-TTHERM_00954250

5002-TTHERM_00954260

5003-TTHERM_00954270

5998-TTHERM_00334530

12163-TTHERM_00540050

14511-TTHERM_00037770

14512-TTHERM_00037780

KIAA1062 protein -

3182-TTHERM_00535970

4616-TTHERM_00529470

7682-TTHERM_00862740

9250-TTHERM_00649360

9271-TTHERM_00125090

16025-TTHERM_00323060

16027-TTHERM_00323080

22270-TTHERM_00218430

26842-TTHERM_00146350

28048-TTHERM_00635770

ABC transporter-

8100-TTHERM_00502610

8102-TTHERM_00502630

13971-TTHERM_00423380

13972-TTHERM_00423390

16326-TTHERM_00083790

18204-TTHERM_00685900

18884-TTHERM_00240450

18888-TTHERM_00241480

20186-TTHERM_01151440

20187-TTHERM_01151450

interaptin-

4419-TTHERM_00773280

25354-TTHERM_00255690

25355-TTHERM_00255700

25359-TTHERM_00256730

25362-TTHERM_00256760

25365-TTHERM_00256790

25366-TTHERM_00256800

25369-TTHERM_00256830

25371-TTHERM_00256850

25375-TTHERM_00256890

hypothetical protein-

16478-TTHERM_00090260

16480-TTHERM_00090280

16484-TTHERM_00090320

16486-TTHERM_00090340

16488-TTHERM_00090360

16489-TTHERM_00090370

16492-TTHERM_00090400

16496-TTHERM_00090440

16498-TTHERM_00091450

16501-TTHERM_00091480

gp91phox-

10704-TTHERM_00510180

10705-TTHERM_00510190

10706-TTHERM_00512180

10707-TTHERM_00513180

10709-TTHERM_00513200

10710-TTHERM_00513210

10711-TTHERM_00514210

10712-TTHERM_00515210

18053-TTHERM_00722930

interaptin-

4931-TTHERM_00283920

16083-TTHERM_01403830

19353-TTHERM_00300580

20138-TTHERM_00703620

25360-TTHERM_00256740

25361-TTHERM_00256750

25372-TTHERM_00256860

25373-TTHERM_00256870

25894-TTHERM_00285650

hypothetical protein-

12857-TTHERM_00653700

12858-TTHERM_00653710

12859-TTHERM_00653720

12860-TTHERM_00653730

12861-TTHERM_00653740

22966-TTHERM_00102660

25656-TTHERM_00582200

26952-TTHERM_01094900

26954-TTHERM_01097890

CG12194-PA -

5767-TTHERM_00578740

5768-TTHERM_00578750

15298-TTHERM_01055440

18092-TTHERM_00723320

18093-TTHERM_00723330

19584-TTHERM_00486530

24496-TTHERM_00139690

24498-TTHERM_00139710

27640-TTHERM_01005230

hypothetical protein-

1939-TTHERM_01382990

2856-TTHERM_01590530

6853-TTHERM_01701350

8268-TTHERM_01535640

16967-TTHERM_02217810

18391-TTHERM_01228950

22191-TTHERM_00903940

24001-TTHERM_01154660

24710-TTHERM_01184210

T15D22.4 protein-

3403-TTHERM_00312150

4186-TTHERM_00492500

4457-TTHERM_00773660

4458-TTHERM_00773670

5071-TTHERM_00617770

5073-TTHERM_00617790

8795-TTHERM_00191440

13401-TTHERM_00361860

13406-TTHERM_00361910

hypothetical protein-

4797-TTHERM_00277560

8880-TTHERM_01150360

9092-TTHERM_00859300

13727-TTHERM_00200570

14465-TTHERM_00037300

15441-TTHERM_00449230

18969-TTHERM_00242320

18985-TTHERM_00242480

27909-TTHERM_00561480

hypothetical protein-

1615-TTHERM_02608240

1776-TTHERM_02175710

2076-TTHERM_01080340

7542-TTHERM_01532570

8218-TTHERM_02555200

16635-TTHERM_02422110

21644-TTHERM_00956490

23963-TTHERM_02646280

29494-TTHERM_02111590

hypothetical protein-

2084-TTHERM_01080420

8283-TTHERM_00979840

8601-TTHERM_00188510

8603-TTHERM_00188530

8606-TTHERM_00188560

8608-TTHERM_00188580

25329-TTHERM_00253460

28955-TTHERM_00576640

28960-TTHERM_00576690

hypothetical protein-

14406-TTHERM_00035720

14411-TTHERM_00035770

14412-TTHERM_00035780

14414-TTHERM_00035800

14415-TTHERM_00036800

14416-TTHERM_00036810

14421-TTHERM_00036860

14422-TTHERM_00036870

14423-TTHERM_00036880

hypothetical protein-

4759-TTHERM_00277160

5599-TTHERM_00441990

5902-TTHERM_01044420

7274-TTHERM_00727730

14837-TTHERM_01227840

22707-TTHERM_00802360

22709-TTHERM_00802390

23554-TTHERM_00393310

24396-TTHERM_00137700

Interaptin-

6584-TTHERM_00630710

22194-TTHERM_00903970

22197-TTHERM_00904000

22198-TTHERM_00904010

22199-TTHERM_00904020

22200-TTHERM_00904030

22201-TTHERM_00904040

22202-TTHERM_00904050

22203-TTHERM_00904060

D-alanyl-D-alanine carboxypeptidase -

1271-TTHERM_00780590

2797-TTHERM_00732760

4422-TTHERM_00773310

11037-TTHERM_01284710

12153-TTHERM_00538960

12323-TTHERM_00522080

19249-TTHERM_00298470

21202-TTHERM_00923230

23150-TTHERM_00688550

Interaptin -

10272-TTHERM_00376200

10273-TTHERM_00377200

10274-TTHERM_00377210

12539-TTHERM_00227290

12545-TTHERM_00227350

12552-TTHERM_00227420

12553-TTHERM_00227430

14097-TTHERM_00427610

29428-TTHERM_00263630

protein tyrosine kinase 1 -

1405-TTHERM_01144900

2469-TTHERM_00245740

8589-TTHERM_00188390

10831-TTHERM_01030010

19380-TTHERM_00301840

19674-TTHERM_00947540

23433-TTHERM_00809380

24670-TTHERM_00628440

interaptin-

1932-TTHERM_01390280

3362-TTHERM_01287940

18131-TTHERM_01579320

18320-TTHERM_01257650

18323-TTHERM_01259660

23445-TTHERM_01370770

23446-TTHERM_01371770

23447-TTHERM_01371780

hypothetical protein -

5139-TTHERM_00621420

14005-TTHERM_00424710

23589-TTHERM_00394650

29859-TTHERM_00185370

29861-TTHERM_00185390

29862-TTHERM_00185400

29863-TTHERM_00185410

29864-TTHERM_00185420

Tlr 4Rp protein -

1717-TTHERM_01797840

2053-TTHERM_01862050

7013-TTHERM_01875060

16787-TTHERM_02184720

16978-TTHERM_02010440

17072-TTHERM_02041500

17586-TTHERM_01896110

23966-TTHERM_02195740

hypothetical protein-

1492-TTHERM_02339990

1493-TTHERM_02340000

4050-TTHERM_01592560

4509-TTHERM_01684280

12951-TTHERM_01272230

14853-TTHERM_02636280

14935-TTHERM_00866440

25218-TTHERM_02279870

hypothetical protein-

4545-TTHERM_01179760

14486-TTHERM_00037510

15264-TTHERM_00927110

15266-TTHERM_00927130

15267-TTHERM_00927140

17533-TTHERM_00697410

23287-TTHERM_00985170

23288-TTHERM_00985180

hexose transporter -

14021-TTHERM_00425860

19475-TTHERM_01125250

19476-TTHERM_01125260

19477-TTHERM_01125270

19478-TTHERM_01125280

19479-TTHERM_01125290

24250-TTHERM_00131290

28372-TTHERM_00157820

probable carbonic anhdrase -

7924-TTHERM_00558270

12913-TTHERM_00654260

14994-TTHERM_00541480

28698-TTHERM_00373840

28702-TTHERM_00374870

28703-TTHERM_00374880

29427-TTHERM_00263620

29432-TTHERM_00263670

hypothetical protein-

7819-TTHERM_00989290

13334-TTHERM_00359210

25199-TTHERM_01356300

25200-TTHERM_01356310

25206-TTHERM_01356370

26270-TTHERM_00751030

26272-TTHERM_00752040

26383-TTHERM_02530180

guanylyl cyclase -

2222-TTHERM_00473220

2286-TTHERM_00474870

2329-TTHERM_00475300

5690-TTHERM_00442900

7005-TTHERM_01156830

11100-TTHERM_00046210

15107-TTHERM_00549660

23898-TTHERM_00666450

related to elongation factor-2 kinase-

6257-TTHERM_00481250

6258-TTHERM_00481260

7218-TTHERM_00420930

15769-TTHERM_01001500

17440-TTHERM_01079250

24053-TTHERM_01090230

25428-TTHERM_00734050

29299-TTHERM_00259330

interaptin-

6032-TTHERM_00335870

12907-TTHERM_00654200

13236-TTHERM_00069460

13509-TTHERM_00414360

13525-TTHERM_00414520

24941-TTHERM_00849400

24944-TTHERM_00849430

24945-TTHERM_00849440

immobilization antigen LD -

1375-TTHERM_00790850

1377-TTHERM_00790870

1379-TTHERM_00790890

1380-TTHERM_00790900

1381-TTHERM_00790910

10798-TTHERM_01523410

13407-TTHERM_00361920

29714-TTHERM_00166070

PACE4 -

13695-TTHERM_00198270

13699-TTHERM_00198310

13705-TTHERM_00198370

13708-TTHERM_00198400

13715-TTHERM_00198470

13723-TTHERM_00200530

24045-TTHERM_01090150

25101-TTHERM_00595460

Tubulin--tyrosine ligase -

7407-TTHERM_00624190

13589-TTHERM_00418200

19263-TTHERM_00299600

20714-TTHERM_00149550

21494-TTHERM_00408780

25002-TTHERM_00997770

25570-TTHERM_00784660

26863-TTHERM_00705080

Unknown -

2968-TTHERM_00691300

18196-TTHERM_00685820

18197-TTHERM_00685830

19665-TTHERM_00947450

19672-TTHERM_00947520

20858-TTHERM_00152000

20859-TTHERM_00152010

20860-TTHERM_00152020

deliriumA-

2944-TTHERM_01574240

7488-TTHERM_01469310

12832-TTHERM_01342660

16191-TTHERM_01456060

16920-TTHERM_01572200

21802-TTHERM_01716400

24030-TTHERM_02119600

30069-TTHERM_01468310

type 1 inositol 1,4,5-trisphosphate receptor-

3277-TTHERM_00762860

7148-TTHERM_00420230

8236-TTHERM_01076970

9276-TTHERM_00125140

11367-TTHERM_00051860

15033-TTHERM_00545830

19903-TTHERM_00532470

24975-TTHERM_00997500

hypothetical protein-

1846-TTHERM_02105570

2585-TTHERM_01662070

3348-TTHERM_01911150

16773-TTHERM_02272860

17146-TTHERM_01732490

26973-TTHERM_01479590

29076-TTHERM_00847060

29077-TTHERM_00848060
