# Supplementary material for: Macronuclear Genome Sequence of the Ciliate Tetrahymena thermophila, a Model Eukaryote
Source: PLoS Biol. 2006 Aug 29;4(9):e286. doi: 10.1371/journal.pbio.0040286 (PMC1557398; doi:10.1371/journal.pbio.0040286)
Supplement: Supplementary file 18 [file pbio.0040286.st011.doc]

Table S11. Expanded versions of Tables 5-8, including TIGR and Genbank IDs for all the identified genes.

A. *Tetrahymena* Kinases. TIGR and Genbank IDs are presented for all kinases referred to in Table 5, along with their classification to kinase Group, Family, and sometimes Subfamily. Families that are unique to *T. thermophila* are provisionally placed in the "Other" group and labeled with a letter-digit combination (A1, E2 etc.). This count and classification are preliminary; many kinases had weak or fragmentary homology to known kinases, and 100 additional very short fragments were omitted from this count.

| **Preliminary TIGR ID** | **GB_accession** | **Group** | **Family** | **Subfamily** |
| --- | --- | --- | --- | --- |
| 1560 | TTHERM_01298470 | AGC | AKT |  |
| 3020 | TTHERM_00691820 | AGC | AKT |  |
| 3983 | TTHERM_00825190 | AGC | AKT |  |
| 5269 | TTHERM_00348570 | AGC | AKT |  |
| 5665 | TTHERM_00442650 | AGC | AKT |  |
| 6414 | TTHERM_00526760 | AGC | AKT |  |
| 14576 | TTHERM_00040400 | AGC | AKT |  |
| 18079 | TTHERM_00723190 | AGC | AKT |  |
| 18101 | TTHERM_00723410 | AGC | AKT |  |
| 19784 | TTHERM_00637810 | AGC | AKT |  |
| 20271 | TTHERM_00455420 | AGC | AKT |  |
| 20554 | TTHERM_00899480 | AGC | AKT |  |
| 23891 | TTHERM_00666380 | AGC | AKT |  |
| 1697 | TTHERM_01126380 | AGC | AktR |  |
| 2238 | TTHERM_00474370 | AGC | AktR |  |
| 3866 | TTHERM_00445970 | AGC | AktR |  |
| 6284 | TTHERM_00483500 | AGC | AktR |  |
| 7169 | TTHERM_00420440 | AGC | AktR |  |
| 7480 | TTHERM_00624920 | AGC | AktR |  |
| 8040 | TTHERM_00500960 | AGC | AktR |  |
| 9468 | TTHERM_00129040 | AGC | AktR |  |
| 15295 | TTHERM_01055410 | AGC | AktR |  |
| 15633 | TTHERM_00295230 | AGC | AktR |  |
| 15961 | TTHERM_00320440 | AGC | AktR |  |
| 19624 | TTHERM_00486930 | AGC | AktR |  |
| 21909 | TTHERM_00726440 | AGC | AktR |  |
| 23531 | TTHERM_00393030 | AGC | AktR |  |
| 23592 | TTHERM_00394690 | AGC | AktR |  |
| 25761 | TTHERM_00463090 | AGC | AktR |  |
| 26010 | TTHERM_00290780 | AGC | AktR |  |
| 26293 | TTHERM_00752250 | AGC | AktR |  |
| 26470 | TTHERM_00431290 | AGC | AktR |  |
| 26471 | TTHERM_00431300 | AGC | AktR |  |
| 9978 | TTHERM_00074350 | AGC | MAST |  |
| 15619 | TTHERM_00295090 | AGC | MAST |  |
| 19957 | TTHERM_00534000 | AGC | MAST |  |
| 3690 | TTHERM_00655960 | AGC | NDR |  |
| 4868 | TTHERM_00283290 | AGC | NDR |  |
| 14389 | TTHERM_00035550 | AGC | NDR |  |
| 18168 | TTHERM_00684550 | AGC | NDR |  |
| 20817 | TTHERM_00151590 | AGC | NDR |  |
| 8643 | TTHERM_00188930 | AGC | PDK1 |  |
| 2171 | TTHERM_00658860 | AGC | PKA |  |
| 17649 | TTHERM_01020820 | AGC | PKA |  |
| 18333 | TTHERM_00992710 | AGC | PKA |  |
| 26009 | TTHERM_00290770 | AGC | PKA |  |
| 26485 | TTHERM_00433420 | AGC | PKA |  |
| 4011 | TTHERM_00825470 | AGC | RSK | RSK |
| 11909 | TTHERM_00016020 | AGC | RSK | RSK |
| 19190 | TTHERM_00616410 | AGC | RSK | Unclassified |
| 29158 | TTHERM_00865280 | AGC | RSK | Unclassified |
| 2547 | TTHERM_00248510 | AGC | SGK |  |
| 10726 | TTHERM_00516340 | AGC | SGK |  |
| 21414 | TTHERM_00794150 | Atypical | A6 |  |
| 30099 | TTHERM_00945200 | Atypical | ABC1 | ABC1-A |
| 14263 | TTHERM_00030320 | Atypical | ABC1 | ABC1-B |
| 17637 | TTHERM_01020700 | Atypical | ABC1 | ABC1-B |
| 23657 | TTHERM_01197150 | Atypical | ABC1 | ABC1-B |
| 6257 | TTHERM_00481250 | Atypical | Alpha |  |
| 6258 | TTHERM_00481260 | Atypical | Alpha |  |
| 7218 | TTHERM_00420930 | Atypical | Alpha |  |
| 10338 | TTHERM_00378840 | Atypical | Alpha |  |
| 15769 | TTHERM_01001500 | Atypical | Alpha |  |
| 15771 | TTHERM_01001520 | Atypical | Alpha |  |
| 24053 | TTHERM_01090230 | Atypical | Alpha |  |
| 25428 | TTHERM_00734050 | Atypical | Alpha |  |
| 29299 | TTHERM_00259330 | Atypical | Alpha |  |
| 1075 | TTHERM_00670510 | Atypical | HisK |  |
| 2892 | TTHERM_00693090 | Atypical | HisK |  |
| 3209 | TTHERM_00537230 | Atypical | HisK |  |
| 3312 | TTHERM_00765200 | Atypical | HisK |  |
| 4106 | TTHERM_00584990 | Atypical | HisK |  |
| 4112 | TTHERM_00585050 | Atypical | HisK |  |
| 4145 | TTHERM_00585390 | Atypical | HisK |  |
| 4146 | TTHERM_00585400 | Atypical | HisK |  |
| 4594 | TTHERM_01014640 | Atypical | HisK |  |
| 5265 | TTHERM_00348530 | Atypical | HisK |  |
| 5266 | TTHERM_00348540 | Atypical | HisK |  |
| 5989 | TTHERM_00334440 | Atypical | HisK |  |
| 6037 | TTHERM_00335940 | Atypical | HisK |  |
| 6390 | TTHERM_00526520 | Atypical | HisK |  |
| 6570 | TTHERM_00630570 | Atypical | HisK |  |
| 6574 | TTHERM_00630610 | Atypical | HisK |  |
| 6859 | TTHERM_00495940 | Atypical | HisK |  |
| 6877 | TTHERM_00497110 | Atypical | HisK |  |
| 7076 | TTHERM_00920790 | Atypical | HisK |  |
| 7183 | TTHERM_00420580 | Atypical | HisK |  |
| 7820 | TTHERM_00989300 | Atypical | HisK |  |
| 7838 | TTHERM_00989480 | Atypical | HisK |  |
| 7982 | TTHERM_00499390 | Atypical | HisK |  |
| 7983 | TTHERM_00499400 | Atypical | HisK |  |
| 8067 | TTHERM_00502280 | Atypical | HisK |  |
| 8442 | TTHERM_01401740 | Atypical | HisK |  |
| 10472 | TTHERM_00569440 | Atypical | HisK |  |
| 10844 | TTHERM_00717460 | Atypical | HisK |  |
| 10845 | TTHERM_00717470 | Atypical | HisK |  |
| 10868 | TTHERM_00717720 | Atypical | HisK |  |
| 11424 | TTHERM_00052440 | Atypical | HisK |  |
| 11754 | TTHERM_00011480 | Atypical | HisK |  |
| 12346 | TTHERM_00522310 | Atypical | HisK |  |
| 12411 | TTHERM_00522960 | Atypical | HisK |  |
| 12733 | TTHERM_00401880 | Atypical | HisK |  |
| 13282 | TTHERM_00070920 | Atypical | HisK |  |
| 13283 | TTHERM_00070930 | Atypical | HisK |  |
| 13303 | TTHERM_00071130 | Atypical | HisK |  |
| 13539 | TTHERM_00415680 | Atypical | HisK |  |
| 13760 | TTHERM_00202880 | Atypical | HisK |  |
| 14296 | TTHERM_00031640 | Atypical | HisK |  |
| 14557 | TTHERM_00039220 | Atypical | HisK |  |
| 14574 | TTHERM_00040380 | Atypical | HisK |  |
| 15301 | TTHERM_01055470 | Atypical | HisK |  |
| 15306 | TTHERM_01055520 | Atypical | HisK |  |
| 15326 | TTHERM_01055720 | Atypical | HisK |  |
| 15614 | TTHERM_00295040 | Atypical | HisK |  |
| 15795 | TTHERM_00646890 | Atypical | HisK |  |
| 15969 | TTHERM_00320520 | Atypical | HisK |  |
| 16029 | TTHERM_00323100 | Atypical | HisK |  |
| 16246 | TTHERM_00842610 | Atypical | HisK |  |
| 16249 | TTHERM_00842640 | Atypical | HisK |  |
| 16839 | TTHERM_00711790 | Atypical | HisK |  |
| 17177 | TTHERM_01002570 | Atypical | HisK |  |
| 17200 | TTHERM_01002800 | Atypical | HisK |  |
| 17202 | TTHERM_01002820 | Atypical | HisK |  |
| 17203 | TTHERM_01002830 | Atypical | HisK |  |
| 17204 | TTHERM_01002840 | Atypical | HisK |  |
| 17211 | TTHERM_01002910 | Atypical | HisK |  |
| 17212 | TTHERM_01002920 | Atypical | HisK |  |
| 18664 | TTHERM_00467620 | Atypical | HisK |  |
| 19275 | TTHERM_00299800 | Atypical | HisK |  |
| 19671 | TTHERM_00947510 | Atypical | HisK |  |
| 19862 | TTHERM_00532040 | Atypical | HisK |  |
| 19891 | TTHERM_00532330 | Atypical | HisK |  |
| 21169 | TTHERM_00922900 | Atypical | HisK |  |
| 21980 | TTHERM_00896090 | Atypical | HisK |  |
| 22059 | TTHERM_00599930 | Atypical | HisK |  |
| 22113 | TTHERM_00600500 | Atypical | HisK |  |
| 22114 | TTHERM_00600510 | Atypical | HisK |  |
| 22136 | TTHERM_00600730 | Atypical | HisK |  |
| 22236 | TTHERM_00216110 | Atypical | HisK |  |
| 22277 | TTHERM_00218500 | Atypical | HisK |  |
| 22512 | TTHERM_00470980 | Atypical | HisK |  |
| 23233 | TTHERM_00797940 | Atypical | HisK |  |
| 25571 | TTHERM_00784670 | Atypical | HisK |  |
| 25684 | TTHERM_00583470 | Atypical | HisK |  |
| 26487 | TTHERM_00433440 | Atypical | HisK |  |
| 26654 | TTHERM_00142480 | Atypical | HisK |  |
| 28479 | TTHERM_00160870 | Atypical | HisK |  |
| 28692 | TTHERM_00373780 | Atypical | HisK |  |
| 29191 | TTHERM_00550830 | Atypical | HisK |  |
| 30008 | TTHERM_00813020 | Atypical | HisK |  |
| 29569 | TTHERM_01008650 | Atypical | PIKK | ATR |
| 13773 | TTHERM_00203010 | Atypical | PIKK | DNAPK |
| 20618 | TTHERM_00147600 | Atypical | PIKK | FRAP |
| 20619 | TTHERM_00147610 | Atypical | PIKK | FRAP |
| 11147 | TTHERM_00046680 | Atypical | RIO | RIO1 |
| 27105 | TTHERM_00112910 | Atypical | RIO | RIO2 |
| 6605 | TTHERM_00193220 | Atypical | TAF1 |  |
| 1303 | TTHERM_00780910 | CAMK | CAMK1 |  |
| 7380 | TTHERM_00622930 | CAMK | CAMK1 |  |
| 10284 | TTHERM_00377310 | CAMK | CAMK1 |  |
| 11728 | TTHERM_00011220 | CAMK | CAMK1 |  |
| 13265 | TTHERM_00070750 | CAMK | CAMK1 |  |
| 22258 | TTHERM_00218310 | CAMK | CAMK1 |  |
| 25228 | TTHERM_01344710 | CAMK | CAMK1 |  |
| 1110 | TTHERM_00670860 | CAMK | CAMKL | AMPK |
| 4451 | TTHERM_00773600 | CAMK | CAMKL | AMPK |
| 7432 | TTHERM_00624440 | CAMK | CAMKL | AMPK |
| 11543 | TTHERM_00001360 | CAMK | CAMKL | AMPK |
| 20428 | TTHERM_00823550 | CAMK | CAMKL | AMPK |
| 21336 | TTHERM_00310320 | CAMK | CAMKL | AMPK |
| 26498 | TTHERM_00433560 | CAMK | CAMKL | AMPK |
| 5439 | TTHERM_00438820 | CAMK | CAMKL | MARK |
| 11331 | TTHERM_00050510 | CAMK | CAMKL | MARK |
| 11795 | TTHERM_00011890 | CAMK | CAMKL | MARK |
| 12692 | TTHERM_00399490 | CAMK | CAMKL | MARK |
| 14385 | TTHERM_00035510 | CAMK | CAMKL | MARK |
| 20667 | TTHERM_00149080 | CAMK | CAMKL | MARK |
| 20819 | TTHERM_00151610 | CAMK | CAMKL | MARK |
| 23838 | TTHERM_00663910 | CAMK | CAMKL | MARK |
| 26669 | TTHERM_00143620 | CAMK | CAMKL | MARK |
| 28996 | TTHERM_00577090 | CAMK | CAMKL | MARK |
| 5359 | TTHERM_00353450 | CAMK | CAMKL | Unclassified |
| 10181 | TTHERM_00079350 | CAMK | CAMKL | Unclassified |
| 14487 | TTHERM_00037530 | CAMK | CAMKL |  |
| 9913 | TTHERM_00275720 | CAMK | CAMK-Tt |  |
| 9914 | TTHERM_00275730 | CAMK | CAMK-Tt |  |
| 19367 | TTHERM_00301710 | CAMK | CAMK-Tt |  |
| 25722 | TTHERM_00460710 | CAMK | CAMK-Tt |  |
| 27757 | TTHERM_01018400 | CAMK | CAMK-Tt |  |
| 5683 | TTHERM_00442830 | CAMK | CAMK-Unique | |
| 23071 | TTHERM_00106700 | CAMK | CAMK-Unique | |
| 2016 | TTHERM_01333220 | CAMK | CDPK |  |
| 2173 | TTHERM_00658880 | CAMK | CDPK |  |
| 2675 | TTHERM_01075630 | CAMK | CDPK |  |
| 4576 | TTHERM_01014460 | CAMK | CDPK |  |
| 5812 | TTHERM_00579190 | CAMK | CDPK |  |
| 7382 | TTHERM_00622950 | CAMK | CDPK |  |
| 7386 | TTHERM_00622990 | CAMK | CDPK |  |
| 8052 | TTHERM_00502130 | CAMK | CDPK |  |
| 10025 | TTHERM_00075800 | CAMK | CDPK |  |
| 11744 | TTHERM_00011380 | CAMK | CDPK |  |
| 12974 | TTHERM_00053930 | CAMK | CDPK |  |
| 16214 | TTHERM_00841290 | CAMK | CDPK |  |
| 18013 | TTHERM_01164090 | CAMK | CDPK |  |
| 19206 | TTHERM_00616570 | CAMK | CDPK |  |
| 19915 | TTHERM_00532590 | CAMK | CDPK |  |
| 22011 | TTHERM_01052880 | CAMK | CDPK |  |
| 22014 | TTHERM_01052910 | CAMK | CDPK |  |
| 22015 | TTHERM_01052920 | CAMK | CDPK |  |
| 24111 | TTHERM_00589960 | CAMK | CDPK |  |
| 28485 | TTHERM_00160930 | CAMK | CDPK |  |
| 3738 | TTHERM_00938880 | CK1 | CK1 |  |
| 3897 | TTHERM_00446300 | CK1 | CK1 |  |
| 4547 | TTHERM_01179780 | CK1 | CK1 |  |
| 5508 | TTHERM_00440530 | CK1 | CK1 |  |
| 5908 | TTHERM_01044590 | CK1 | CK1 |  |
| 7080 | TTHERM_00920830 | CK1 | CK1 |  |
| 7132 | TTHERM_00420070 | CK1 | CK1 |  |
| 7563 | TTHERM_00677990 | CK1 | CK1 |  |
| 8104 | TTHERM_00502650 | CK1 | CK1 |  |
| 8793 | TTHERM_00191420 | CK1 | CK1 |  |
| 10222 | TTHERM_00079770 | CK1 | CK1 |  |
| 13675 | TTHERM_00198070 | CK1 | CK1 |  |
| 18951 | TTHERM_00242130 | CK1 | CK1 |  |
| 20116 | TTHERM_00703400 | CK1 | CK1 |  |
| 22792 | TTHERM_01213960 | CK1 | CK1 |  |
| 23028 | TTHERM_00105280 | CK1 | CK1 |  |
| 25400 | TTHERM_00257140 | CK1 | CK1 |  |
| 26759 | TTHERM_00145510 | CK1 | CK1 |  |
| 29795 | TTHERM_00171860 | CK1 | CK1 |  |
| 6129 | TTHERM_00339880 | CMGC | CDK | CDC2 |
| 6298 | TTHERM_00483640 | CMGC | CDK | CDC2 |
| 9119 | TTHERM_01207660 | CMGC | CDK | CDC2 |
| 9560 | TTHERM_01035490 | CMGC | CDK | CDC2 |
| 21071 | TTHERM_00318700 | CMGC | CDK | CDC2 |
| 21599 | TTHERM_00411810 | CMGC | CDK | CDC2 |
| 21600 | TTHERM_00411820 | CMGC | CDK | CDC2 |
| 23583 | TTHERM_00394590 | CMGC | CDK | CDC2 |
| 25533 | TTHERM_00784290 | CMGC | CDK | CDC2 |
| 25907 | TTHERM_00286770 | CMGC | CDK | CDC2 |
| 26482 | TTHERM_00433390 | CMGC | CDK | CDC2 |
| 11773 | TTHERM_00011670 | CMGC | CDK | CRK7 |
| 13176 | TTHERM_00066860 | CMGC | CDK | PITSLRE |
| 2101 | TTHERM_01080590 | CMGC | CDK | Unclassified |
| 7414 | TTHERM_00624260 | CMGC | CDKL |  |
| 10873 | TTHERM_00717780 | CMGC | CDKL |  |
| 17811 | TTHERM_00837980 | CMGC | CDKL |  |
| 29899 | TTHERM_00185770 | CMGC | CDKL |  |
| 15737 | TTHERM_01000180 | CMGC | CK2 |  |
| 28298 | TTHERM_01276350 | CMGC | CK2 |  |
| 22369 | TTHERM_00219410 | CMGC | CLK |  |
| 22349 | TTHERM_00219210 | CMGC | DYRK | DYRK1 |
| 5306 | TTHERM_00348940 | CMGC | DYRK | DYRK2 |
| 5356 | TTHERM_00353420 | CMGC | DYRK | DYRK2 |
| 5655 | TTHERM_00442550 | CMGC | DYRK | DYRK2 |
| 10580 | TTHERM_00641110 | CMGC | DYRK | DYRK2 |
| 22423 | TTHERM_00220950 | CMGC | DYRK | DYRK2 |
| 15575 | TTHERM_00294650 | CMGC | DYRK | PRP4 |
| 2636 | TTHERM_01016020 | CMGC | GSK |  |
| 5825 | TTHERM_00579320 | CMGC | GSK |  |
| 7876 | TTHERM_00557780 | CMGC | GSK |  |
| 19912 | TTHERM_00532560 | CMGC | GSK |  |
| 19923 | TTHERM_00532670 | CMGC | GSK |  |
| 19924 | TTHERM_00532680 | CMGC | GSK |  |
| 4173 | TTHERM_00492370 | CMGC | MAPK | ERK |
| 6784 | TTHERM_00195990 | CMGC | MAPK | ERK |
| 13785 | TTHERM_00204120 | CMGC | MAPK | ERK |
| 18504 | TTHERM_00760190 | CMGC | MAPK | ERK |
| 18512 | TTHERM_00760270 | CMGC | MAPK | ERK |
| 18725 | TTHERM_00469230 | CMGC | MAPK | ERK |
| 23186 | TTHERM_00689900 | CMGC | MAPK | ERK |
| 3210 | TTHERM_00537240 | CMGC | MAPK | ERK7 |
| 27541 | TTHERM_00355550 | CMGC | MAPK | ERK7 |
| 28942 | TTHERM_00575520 | CMGC | MAPK | ERK7 |
| 11145 | TTHERM_00046660 | CMGC | MAPK | Unclassified |
| 16370 | TTHERM_00085220 | CMGC | MAPK | Unclassified |
| 23757 | TTHERM_00660130 | CMGC | MAPK | Unclassified |
| 28107 | TTHERM_01332120 | CMGC | MAPK | Unclassified |
| 7690 | TTHERM_01347900 | CMGC | RCK |  |
| 9133 | TTHERM_01309100 | CMGC | RCK |  |
| 9821 | TTHERM_00267860 | CMGC | RCK |  |
| 13065 | TTHERM_00058800 | CMGC | RCK |  |
| 15513 | TTHERM_00450990 | CMGC | RCK |  |
| 26702 | TTHERM_00144940 | CMGC | RCK |  |
| 27248 | TTHERM_00122330 | CMGC | RCK |  |
| 28968 | TTHERM_00576770 | CMGC | RCK |  |
| 3884 | TTHERM_00446150 | CMGC | SRPK |  |
| 13234 | TTHERM_00069440 | CMGC | SRPK |  |
| 22244 | TTHERM_00217180 | CMGC | SRPK |  |
| 1167 | TTHERM_00851680 | CMGC | Unique |  |
| 13031 | TTHERM_00058460 | Other | A1 |  |
| 13032 | TTHERM_00058470 | Other | A1 |  |
| 19713 | TTHERM_00637100 | Other | A1 |  |
| 19714 | TTHERM_00637110 | Other | A1 |  |
| 19716 | TTHERM_00637130 | Other | A1 |  |
| 19717 | TTHERM_00637140 | Other | A1 |  |
| 3376 | TTHERM_01466240 | Other | A2 |  |
| 7617 | TTHERM_00678530 | Other | A2 |  |
| 11712 | TTHERM_00011000 | Other | A2 |  |
| 12283 | TTHERM_00856580 | Other | A2 |  |
| 12290 | TTHERM_00856650 | Other | A2 |  |
| 13750 | TTHERM_00202780 | Other | A2 |  |
| 14530 | TTHERM_00038950 | Other | A2 |  |
| 16906 | TTHERM_00713550 | Other | A2 |  |
| 16913 | TTHERM_00713620 | Other | A2 |  |
| 22851 | TTHERM_00095520 | Other | A2 |  |
| 22852 | TTHERM_00095530 | Other | A2 |  |
| 23053 | TTHERM_00105530 | Other | A2 |  |
| 25405 | TTHERM_00257190 | Other | A2 |  |
| 27335 | TTHERM_00601840 | Other | A2 |  |
| 27990 | TTHERM_00633210 | Other | A2 |  |
| 1609 | TTHERM_01538680 | Other | A3 |  |
| 4613 | TTHERM_01639960 | Other | A3 |  |
| 10899 | TTHERM_00718040 | Other | A3 |  |
| 10908 | TTHERM_00720110 | Other | A3 |  |
| 16375 | TTHERM_00085270 | Other | A4 |  |
| 16382 | TTHERM_00085340 | Other | A4 |  |
| 16383 | TTHERM_00085350 | Other | A4 |  |
| 16384 | TTHERM_00085360 | Other | A4 |  |
| 16385 | TTHERM_00085370 | Other | A4 |  |
| 18266 | TTHERM_00755890 | Other | A4 |  |
| 4078 | TTHERM_00584700 | Other | A5 |  |
| 7557 | TTHERM_00676940 | Other | A5 |  |
| 8254 | TTHERM_01169450 | Other | A5 |  |
| 10631 | TTHERM_00644600 | Other | A5 |  |
| 13181 | TTHERM_00066910 | Other | A5 |  |
| 18782 | TTHERM_00237380 | Other | A5 |  |
| 19604 | TTHERM_00486730 | Other | A5 |  |
| 22050 | TTHERM_00599830 | Other | A5 |  |
| 29759 | TTHERM_00170510 | Other | A5 |  |
| 30307 | TTHERM_00330020 | Other | A5 |  |
| 2641 | TTHERM_01016070 | Other | A6 |  |
| 5409 | TTHERM_00437530 | Other | A6 |  |
| 5517 | TTHERM_00440630 | Other | A6 |  |
| 8975 | TTHERM_00564370 | Other | A6 |  |
| 8976 | TTHERM_00564380 | Other | A6 |  |
| 19056 | TTHERM_00715820 | Other | A6 |  |
| 21621 | TTHERM_00412030 | Other | A6 |  |
| 22526 | TTHERM_00471120 | Other | A6 |  |
| 24780 | TTHERM_00384880 | Other | A6 |  |
| 2721 | TTHERM_01205310 | Other | A7 |  |
| 2963 | TTHERM_00691250 | Other | A7 |  |
| 3172 | TTHERM_00535870 | Other | A7 |  |
| 6866 | TTHERM_00497000 | Other | A7 |  |
| 6891 | TTHERM_00497250 | Other | A7 |  |
| 9865 | TTHERM_00268310 | Other | A7 |  |
| 11522 | TTHERM_00001150 | Other | A7 |  |
| 14731 | TTHERM_01358400 | Other | A7 |  |
| 19451 | TTHERM_01265030 | Other | A7 |  |
| 21201 | TTHERM_00923220 | Other | A7 |  |
| 28806 | TTHERM_01026430 | Other | A7 |  |
| 7343 | TTHERM_01244600 | Other | A8 |  |
| 9287 | TTHERM_00125250 | Other | A8 |  |
| 9756 | TTHERM_00266210 | Other | A8 |  |
| 9946 | TTHERM_01496790 | Other | A8 |  |
| 10482 | TTHERM_00569540 | Other | A8 |  |
| 13474 | TTHERM_00366550 | Other | A8 |  |
| 13483 | TTHERM_00369610 | Other | A8 |  |
| 16356 | TTHERM_00085080 | Other | A8 |  |
| 18442 | TTHERM_00721340 | Other | A8 |  |
| 22398 | TTHERM_00220700 | Other | A8 |  |
| 26895 | TTHERM_00706390 | Other | A8 |  |
| 6861 | TTHERM_00495960 | Other | A9 |  |
| 12433 | TTHERM_00222260 | Other | A9 |  |
| 13125 | TTHERM_00059410 | Other | A9 |  |
| 20983 | TTHERM_00316830 | Other | A9 |  |
| 23520 | TTHERM_00392920 | Other | A9 |  |
| 24862 | TTHERM_00389660 | Other | A9 |  |
| 5973 | TTHERM_00334280 | Other | Aur |  |
| 9766 | TTHERM_00266320 | Other | Aur |  |
| 12754 | TTHERM_00402100 | Other | Aur |  |
| 13285 | TTHERM_00070950 | Other | Aur |  |
| 13287 | TTHERM_00070970 | Other | Aur |  |
| 15246 | TTHERM_00926910 | Other | Aur |  |
| 16602 | TTHERM_01350000 | Other | Aur |  |
| 18172 | TTHERM_00684590 | Other | Aur |  |
| 19498 | TTHERM_01266090 | Other | Aur |  |
| 19636 | TTHERM_00487050 | Other | Aur |  |
| 22429 | TTHERM_00221010 | Other | Aur |  |
| 25157 | TTHERM_00941510 | Other | Aur |  |
| 26118 | TTHERM_00343380 | Other | Aur |  |
| 26122 | TTHERM_00343420 | Other | Aur |  |
| 9435 | TTHERM_00128710 | Other | B1 |  |
| 9436 | TTHERM_00128720 | Other | B1 |  |
| 9437 | TTHERM_00128730 | Other | B1 |  |
| 9438 | TTHERM_00128740 | Other | B1 |  |
| 19333 | TTHERM_00300380 | Other | B1 |  |
| 20853 | TTHERM_00151950 | Other | B1 |  |
| 20855 | TTHERM_00151970 | Other | B1 |  |
| 20856 | TTHERM_00151980 | Other | B1 |  |
| 6306 | TTHERM_00484720 | Other | B2 |  |
| 11484 | TTHERM_01140390 | Other | B2 |  |
| 11486 | TTHERM_01140410 | Other | B2 |  |
| 18199 | TTHERM_00685850 | Other | B2 |  |
| 21007 | TTHERM_00317070 | Other | B2 |  |
| 23098 | TTHERM_00107040 | Other | B2 |  |
| 25583 | TTHERM_00784810 | Other | B2 |  |
| 26473 | TTHERM_00431320 | Other | B2 |  |
| 8814 | TTHERM_00191640 | Other | B3 |  |
| 8815 | TTHERM_00191650 | Other | B3 |  |
| 8817 | TTHERM_00191670 | Other | B3 |  |
| 8818 | TTHERM_00191680 | Other | B3 |  |
| 4601 | TTHERM_01014710 | Other | Bud32 |  |
| 1114 | TTHERM_00670900 | Other | C1 |  |
| 3202 | TTHERM_00537160 | Other | C1 |  |
| 4107 | TTHERM_00585000 | Other | C1 |  |
| 20533 | TTHERM_00898280 | Other | C1 |  |
| 22427 | TTHERM_00220990 | Other | C1 |  |
| 27962 | TTHERM_00632930 | Other | C1 |  |
| 6314 | TTHERM_01424450 | Other | C2 |  |
| 12227 | TTHERM_01263930 | Other | C2 |  |
| 12230 | TTHERM_01263960 | Other | C2 |  |
| 12233 | TTHERM_01263990 | Other | C2 |  |
| 13367 | TTHERM_00361520 | Other | C2 |  |
| 13371 | TTHERM_00361560 | Other | C2 |  |
| 19025 | TTHERM_01319500 | Other | C2 |  |
| 24009 | TTHERM_01155730 | Other | C2 |  |
| 25503 | TTHERM_01211820 | Other | C2 |  |
| 25504 | TTHERM_01211840 | Other | C2 |  |
| 27697 | TTHERM_01483630 | Other | C2 |  |
| 28067 | TTHERM_01410020 | Other | C2 |  |
| 9189 | TTHERM_00648750 | Other | C3 |  |
| 9190 | TTHERM_00648760 | Other | C3 |  |
| 15975 | TTHERM_00321570 | Other | C3 |  |
| 15976 | TTHERM_00321580 | Other | C3 |  |
| 26110 | TTHERM_00343300 | Other | C3 |  |
| 6826 | TTHERM_00196410 | Other | C4 |  |
| 11348 | TTHERM_00051670 | Other | C4 |  |
| 11769 | TTHERM_00011630 | Other | C4 |  |
| 14048 | TTHERM_00426130 | Other | C4 |  |
| 22331 | TTHERM_00219030 | Other | C4 |  |
| 27883 | TTHERM_00561220 | Other | C4 |  |
| 2009 | TTHERM_01333150 | Other | C5 |  |
| 2011 | TTHERM_01333170 | Other | C5 |  |
| 2942 | TTHERM_01574220 | Other | C5 |  |
| 3081 | TTHERM_01451010 | Other | C5 |  |
| 5526 | TTHERM_01661070 | Other | C5 |  |
| 7492 | TTHERM_01469350 | Other | C5 |  |
| 7706 | TTHERM_01474480 | Other | C5 |  |
| 12088 | TTHERM_01327890 | Other | C5 |  |
| 15362 | TTHERM_01329990 | Other | C5 |  |
| 16546 | TTHERM_00092920 | Other | C5 |  |
| 18047 | TTHERM_01638940 | Other | C5 |  |
| 18408 | TTHERM_01230130 | Other | C5 |  |
| 24721 | TTHERM_01187290 | Other | C5 |  |
| 25224 | TTHERM_01344670 | Other | C5 |  |
| 28272 | TTHERM_01889100 | Other | C5 |  |
| 3630 | TTHERM_00655320 | Other | C6 |  |
| 10855 | TTHERM_00717590 | Other | C6 |  |
| 15445 | TTHERM_00449320 | Other | C6 |  |
| 15446 | TTHERM_00449330 | Other | C6 |  |
| 15448 | TTHERM_00449350 | Other | C6 |  |
| 15449 | TTHERM_00449360 | Other | C6 |  |
| 15450 | TTHERM_00449370 | Other | C6 |  |
| 15451 | TTHERM_00449380 | Other | C6 |  |
| 15452 | TTHERM_00449390 | Other | C6 |  |
| 15453 | TTHERM_00449400 | Other | C6 |  |
| 19066 | TTHERM_00715940 | Other | C6 |  |
| 21061 | TTHERM_00318600 | Other | C6 |  |
| 1990 | TTHERM_01693300 | Other | C7 |  |
| 3066 | TTHERM_01582400 | Other | C7 |  |
| 4498 | TTHERM_01376880 | Other | C7 |  |
| 4501 | TTHERM_01377900 | Other | C7 |  |
| 5174 | TTHERM_01086730 | Other | C7 |  |
| 5844 | TTHERM_01498820 | Other | C7 |  |
| 6993 | TTHERM_02370050 | Other | C7 |  |
| 8114 | TTHERM_01742570 | Other | C7 |  |
| 9595 | TTHERM_00815090 | Other | C7 |  |
| 11466 | TTHERM_01138230 | Other | C7 |  |
| 12223 | TTHERM_01611750 | Other | C7 |  |
| 12241 | TTHERM_01558040 | Other | C7 |  |
| 12644 | TTHERM_00236260 | Other | C7 |  |
| 13106 | TTHERM_00059220 | Other | C7 |  |
| 13633 | TTHERM_00197650 | Other | C7 |  |
| 13816 | TTHERM_00209380 | Other | C7 |  |
| 14173 | TTHERM_01695310 | Other | C7 |  |
| 14925 | TTHERM_00702270 | Other | C7 |  |
| 16201 | TTHERM_01640980 | Other | C7 |  |
| 17306 | TTHERM_01220420 | Other | C7 |  |
| 17307 | TTHERM_01220430 | Other | C7 |  |
| 17699 | TTHERM_02293890 | Other | C7 |  |
| 18245 | TTHERM_01620830 | Other | C7 |  |
| 19406 | TTHERM_01948320 | Other | C7 |  |
| 19991 | TTHERM_01388120 | Other | C7 |  |
| 19995 | TTHERM_01388160 | Other | C7 |  |
| 20038 | TTHERM_00994420 | Other | C7 |  |
| 20891 | TTHERM_01414120 | Other | C7 |  |
| 21480 | TTHERM_01618810 | Other | C7 |  |
| 21673 | TTHERM_00958760 | Other | C7 |  |
| 21676 | TTHERM_00958790 | Other | C7 |  |
| 21728 | TTHERM_00976420 | Other | C7 |  |
| 21795 | TTHERM_01541750 | Other | C7 |  |
| 22155 | TTHERM_00900600 | Other | C7 |  |
| 22632 | TTHERM_01656030 | Other | C7 |  |
| 22799 | TTHERM_00935420 | Other | C7 |  |
| 24183 | TTHERM_01689280 | Other | C7 |  |
| 24609 | TTHERM_02013450 | Other | C7 |  |
| 25184 | TTHERM_01515340 | Other | C7 |  |
| 26369 | TTHERM_01644000 | Other | C7 |  |
| 26374 | TTHERM_01663090 | Other | C7 |  |
| 26388 | TTHERM_01565130 | Other | C7 |  |
| 27530 | TTHERM_00355440 | Other | C7 |  |
| 27656 | TTHERM_01494740 | Other | C7 |  |
| 27999 | TTHERM_00633300 | Other | C7 |  |
| 28253 | TTHERM_01428470 | Other | C7 |  |
| 29096 | TTHERM_01564120 | Other | C7 |  |
| 29524 | TTHERM_01504060 | Other | C7 |  |
| 29587 | TTHERM_01246670 | Other | C7 |  |
| 29589 | TTHERM_01246690 | Other | C7 |  |
| 6998 | TTHERM_01156760 | Other | C8 |  |
| 2605 | TTHERM_01685280 | Other | C8 |  |
| 2815 | TTHERM_01438780 | Other | C8 |  |
| 2817 | TTHERM_01440780 | Other | C8 |  |
| 3357 | TTHERM_01615780 | Other | C8 |  |
| 4505 | TTHERM_01378930 | Other | C8 |  |
| 5060 | TTHERM_01461130 | Other | C8 |  |
| 6997 | TTHERM_01156750 | Other | C8 |  |
| 12085 | TTHERM_01670170 | Other | C8 |  |
| 13310 | TTHERM_01170560 | Other | C8 |  |
| 13323 | TTHERM_01174650 | Other | C8 |  |
| 13336 | TTHERM_00359230 | Other | C8 |  |
| 13347 | TTHERM_00360330 | Other | C8 |  |
| 13374 | TTHERM_00361590 | Other | C8 |  |
| 14779 | TTHERM_01119390 | Other | C8 |  |
| 14781 | TTHERM_01119410 | Other | C8 |  |
| 14782 | TTHERM_01119420 | Other | C8 |  |
| 15779 | TTHERM_01365640 | Other | C8 |  |
| 15780 | TTHERM_01365650 | Other | C8 |  |
| 17855 | TTHERM_01627910 | Other | C8 |  |
| 17858 | TTHERM_01082840 | Other | C8 |  |
| 19028 | TTHERM_01319530 | Other | C8 |  |
| 19409 | TTHERM_01725470 | Other | C8 |  |
| 21155 | TTHERM_00778430 | Other | C8 |  |
| 21704 | TTHERM_01361490 | Other | C8 |  |
| 22826 | TTHERM_00937670 | Other | C8 |  |
| 22828 | TTHERM_00937690 | Other | C8 |  |
| 23667 | TTHERM_00959810 | Other | C8 |  |
| 23995 | TTHERM_01153610 | Other | C8 |  |
| 23998 | TTHERM_01153640 | Other | C8 |  |
| 24005 | TTHERM_01155690 | Other | C8 |  |
| 24007 | TTHERM_01155710 | Other | C8 |  |
| 24213 | TTHERM_01006560 | Other | C8 |  |
| 25202 | TTHERM_01356330 | Other | C8 |  |
| 25205 | TTHERM_01356360 | Other | C8 |  |
| 26273 | TTHERM_00752050 | Other | C8 |  |
| 26387 | TTHERM_01565120 | Other | C8 |  |
| 28064 | TTHERM_01409990 | Other | C8 |  |
| 28099 | TTHERM_01331050 | Other | C8 |  |
| 28243 | TTHERM_01569160 | Other | C8 |  |
| 29103 | TTHERM_01445920 | Other | C8 |  |
| 29554 | TTHERM_01296380 | Other | C8 |  |
| 29634 | TTHERM_00963270 | Other | C8 |  |
| 30140 | TTHERM_01148240 | Other | C8 |  |
| 7318 | TTHERM_00729170 | Other | CDC7 |  |
| 1168 | TTHERM_00852690 | Other | D |  |
| 1573 | TTHERM_01298610 | Other | D |  |
| 1649 | TTHERM_01345800 | Other | D |  |
| 1794 | TTHERM_01038810 | Other | D |  |
| 2219 | TTHERM_00473190 | Other | D |  |
| 2788 | TTHERM_00732670 | Other | D |  |
| 2882 | TTHERM_00692980 | Other | D |  |
| 3331 | TTHERM_00765390 | Other | D |  |
| 3335 | TTHERM_00766420 | Other | D |  |
| 3472 | TTHERM_00312850 | Other | D |  |
| 3618 | TTHERM_01435710 | Other | D |  |
| 5379 | TTHERM_00436240 | Other | D |  |
| 5809 | TTHERM_00579160 | Other | D |  |
| 5902 | TTHERM_01044420 | Other | D |  |
| 5990 | TTHERM_00334450 | Other | D |  |
| 6373 | TTHERM_00526350 | Other | D |  |
| 6536 | TTHERM_00630170 | Other | D |  |
| 6762 | TTHERM_00194780 | Other | D |  |
| 6910 | TTHERM_00497440 | Other | D |  |
| 7237 | TTHERM_00421130 | Other | D |  |
| 7291 | TTHERM_00728890 | Other | D |  |
| 7361 | TTHERM_00622740 | Other | D |  |
| 7400 | TTHERM_00624120 | Other | D |  |
| 7412 | TTHERM_00624240 | Other | D |  |
| 7504 | TTHERM_01194670 | Other | D |  |
| 7644 | TTHERM_02472150 | Other | D |  |
| 7789 | TTHERM_00822360 | Other | D |  |
| 7822 | TTHERM_00989320 | Other | D |  |
| 8053 | TTHERM_00502140 | Other | D |  |
| 8538 | TTHERM_00787110 | Other | D |  |
| 8547 | TTHERM_00787200 | Other | D |  |
| 8761 | TTHERM_00191100 | Other | D |  |
| 8837 | TTHERM_00191870 | Other | D |  |
| 8908 | TTHERM_01683250 | Other | D |  |
| 9066 | TTHERM_00858020 | Other | D |  |
| 9300 | TTHERM_00125380 | Other | D |  |
| 9755 | TTHERM_00266200 | Other | D |  |
| 10000 | TTHERM_00075560 | Other | D |  |
| 10243 | TTHERM_00079980 | Other | D |  |
| 10245 | TTHERM_00080000 | Other | D |  |
| 10479 | TTHERM_00569510 | Other | D |  |
| 10505 | TTHERM_00571750 | Other | D |  |
| 11112 | TTHERM_00046330 | Other | D |  |
| 11215 | TTHERM_00047360 | Other | D |  |
| 11408 | TTHERM_00052280 | Other | D |  |
| 11644 | TTHERM_00006360 | Other | D |  |
| 12052 | TTHERM_00024390 | Other | D |  |
| 12145 | TTHERM_00538880 | Other | D |  |
| 12475 | TTHERM_00225650 | Other | D |  |
| 12538 | TTHERM_00227280 | Other | D |  |
| 12856 | TTHERM_00653690 | Other | D |  |
| 13052 | TTHERM_00058670 | Other | D |  |
| 13217 | TTHERM_00069270 | Other | D |  |
| 13258 | TTHERM_00069690 | Other | D |  |
| 13276 | TTHERM_00070860 | Other | D |  |
| 13389 | TTHERM_00361740 | Other | D |  |
| 13752 | TTHERM_00202800 | Other | D |  |
| 13938 | TTHERM_00771990 | Other | D |  |
| 14297 | TTHERM_00031650 | Other | D |  |
| 14332 | TTHERM_00034970 | Other | D |  |
| 14732 | TTHERM_01358410 | Other | D |  |
| 14780 | TTHERM_01119400 | Other | D |  |
| 14836 | TTHERM_01227810 | Other | D |  |
| 15035 | TTHERM_00545850 | Other | D |  |
| 15587 | TTHERM_00294770 | Other | D |  |
| 15732 | TTHERM_00297210 | Other | D |  |
| 15764 | TTHERM_01001450 | Other | D |  |
| 15778 | TTHERM_01365630 | Other | D |  |
| 15974 | TTHERM_00321560 | Other | D |  |
| 16552 | TTHERM_00093970 | Other | D |  |
| 16697 | TTHERM_02510180 | Other | D |  |
| 17269 | TTHERM_01709380 | Other | D |  |
| 17295 | TTHERM_01220310 | Other | D |  |
| 17356 | TTHERM_00929440 | Other | D |  |
| 17452 | TTHERM_00695560 | Other | D |  |
| 17457 | TTHERM_00695610 | Other | D |  |
| 17505 | TTHERM_00697080 | Other | D |  |
| 17861 | TTHERM_01082870 | Other | D |  |
| 17985 | TTHERM_01027560 | Other | D |  |
| 18020 | TTHERM_01164160 | Other | D |  |
| 18036 | TTHERM_01193540 | Other | D |  |
| 18054 | TTHERM_00722940 | Other | D |  |
| 18056 | TTHERM_00722960 | Other | D |  |
| 18517 | TTHERM_00760320 | Other | D |  |
| 18735 | TTHERM_00469330 | Other | D |  |
| 18785 | TTHERM_00237410 | Other | D |  |
| 18786 | TTHERM_00237420 | Other | D |  |
| 18946 | TTHERM_00242080 | Other | D |  |
| 19053 | TTHERM_00715790 | Other | D |  |
| 19106 | TTHERM_00716340 | Other | D |  |
| 19298 | TTHERM_00300030 | Other | D |  |
| 20266 | TTHERM_00455370 | Other | D |  |
| 20272 | TTHERM_00455430 | Other | D |  |
| 20431 | TTHERM_00823610 | Other | D |  |
| 20457 | TTHERM_00823890 | Other | D |  |
| 20607 | TTHERM_00147490 | Other | D |  |
| 20738 | TTHERM_00149780 | Other | D |  |
| 21075 | TTHERM_00318740 | Other | D |  |
| 21888 | TTHERM_00726230 | Other | D |  |
| 22063 | TTHERM_00599970 | Other | D |  |
| 22078 | TTHERM_00600150 | Other | D |  |
| 22110 | TTHERM_00600470 | Other | D |  |
| 22219 | TTHERM_00215950 | Other | D |  |
| 22735 | TTHERM_00803640 | Other | D |  |
| 23019 | TTHERM_00105190 | Other | D |  |
| 23282 | TTHERM_00985120 | Other | D |  |
| 23775 | TTHERM_00660310 | Other | D |  |
| 23882 | TTHERM_00666290 | Other | D |  |
| 24136 | TTHERM_00590210 | Other | D |  |
| 24200 | TTHERM_01006430 | Other | D |  |
| 24300 | TTHERM_00133770 | Other | D |  |
| 24856 | TTHERM_00388610 | Other | D |  |
| 24858 | TTHERM_00388630 | Other | D |  |
| 25269 | TTHERM_00250880 | Other | D |  |
| 25399 | TTHERM_00257130 | Other | D |  |
| 25478 | TTHERM_00736530 | Other | D |  |
| 25731 | TTHERM_00461790 | Other | D |  |
| 25965 | TTHERM_00289330 | Other | D |  |
| 26359 | TTHERM_01161050 | Other | D |  |
| 26360 | TTHERM_01162050 | Other | D |  |
| 26549 | TTHERM_00434080 | Other | D |  |
| 26552 | TTHERM_00434110 | Other | D |  |
| 26582 | TTHERM_00140770 | Other | D |  |
| 26666 | TTHERM_00143590 | Other | D |  |
| 26670 | TTHERM_00143630 | Other | D |  |
| 26699 | TTHERM_00144910 | Other | D |  |
| 26940 | TTHERM_01094780 | Other | D |  |
| 27040 | TTHERM_00109290 | Other | D |  |
| 27042 | TTHERM_00112280 | Other | D |  |
| 27070 | TTHERM_00112560 | Other | D |  |
| 27370 | TTHERM_00607140 | Other | D |  |
| 27482 | TTHERM_00354960 | Other | D |  |
| 27698 | TTHERM_01483640 | Other | D |  |
| 27908 | TTHERM_00561470 | Other | D |  |
| 28415 | TTHERM_00158250 | Other | D |  |
| 28529 | TTHERM_00161390 | Other | D |  |
| 28969 | TTHERM_00576780 | Other | D |  |
| 28991 | TTHERM_00577040 | Other | D |  |
| 29013 | TTHERM_00577260 | Other | D |  |
| 29028 | TTHERM_00577410 | Other | D |  |
| 29059 | TTHERM_00845900 | Other | D |  |
| 29190 | TTHERM_00550820 | Other | D |  |
| 29314 | TTHERM_00259520 | Other | D |  |
| 29366 | TTHERM_00263010 | Other | D |  |
| 30107 | TTHERM_00945280 | Other | D |  |
| 30187 | TTHERM_00326840 | Other | D |  |
| 30298 | TTHERM_00329930 | Other | D |  |
| 1365 | TTHERM_00790750 | Other | E1 |  |
| 1931 | TTHERM_01390270 | Other | E1 |  |
| 1932 | TTHERM_01390280 | Other | E1 |  |
| 2142 | TTHERM_00657580 | Other | E1 |  |
| 3360 | TTHERM_01286930 | Other | E1 |  |
| 3362 | TTHERM_01287940 | Other | E1 |  |
| 4854 | TTHERM_00283150 | Other | E1 |  |
| 4856 | TTHERM_00283170 | Other | E1 |  |
| 5007 | TTHERM_00954310 | Other | E1 |  |
| 5185 | TTHERM_01087840 | Other | E1 |  |
| 6000 | TTHERM_00334560 | Other | E1 |  |
| 7296 | TTHERM_00728950 | Other | E1 |  |
| 10617 | TTHERM_00643470 | Other | E1 |  |
| 10618 | TTHERM_00643480 | Other | E1 |  |
| 10885 | TTHERM_00717900 | Other | E1 |  |
| 10886 | TTHERM_00717910 | Other | E1 |  |
| 12019 | TTHERM_00024060 | Other | E1 |  |
| 12559 | TTHERM_00227490 | Other | E1 |  |
| 12560 | TTHERM_00227500 | Other | E1 |  |
| 12561 | TTHERM_00227510 | Other | E1 |  |
| 12562 | TTHERM_00227520 | Other | E1 |  |
| 12563 | TTHERM_00227530 | Other | E1 |  |
| 12577 | TTHERM_00227670 | Other | E1 |  |
| 13378 | TTHERM_00361630 | Other | E1 |  |
| 13379 | TTHERM_00361640 | Other | E1 |  |
| 13472 | TTHERM_00365540 | Other | E1 |  |
| 13476 | TTHERM_00367560 | Other | E1 |  |
| 13478 | TTHERM_00368570 | Other | E1 |  |
| 13479 | TTHERM_00368580 | Other | E1 |  |
| 13481 | TTHERM_00369590 | Other | E1 |  |
| 14911 | TTHERM_00701140 | Other | E1 |  |
| 14987 | TTHERM_00875870 | Other | E1 |  |
| 18319 | TTHERM_01257640 | Other | E1 |  |
| 18320 | TTHERM_01257650 | Other | E1 |  |
| 18323 | TTHERM_01259660 | Other | E1 |  |
| 18324 | TTHERM_01259670 | Other | E1 |  |
| 19121 | TTHERM_00614730 | Other | E1 |  |
| 19947 | TTHERM_00533900 | Other | E1 |  |
| 19973 | TTHERM_01406900 | Other | E1 |  |
| 20471 | TTHERM_00824030 | Other | E1 |  |
| 21069 | TTHERM_00318680 | Other | E1 |  |
| 21147 | TTHERM_00777360 | Other | E1 |  |
| 21184 | TTHERM_00923050 | Other | E1 |  |
| 22157 | TTHERM_00900620 | Other | E1 |  |
| 22159 | TTHERM_00901630 | Other | E1 |  |
| 22786 | TTHERM_01213900 | Other | E1 |  |
| 22819 | TTHERM_00936610 | Other | E1 |  |
| 22827 | TTHERM_00937680 | Other | E1 |  |
| 23086 | TTHERM_00106920 | Other | E1 |  |
| 23445 | TTHERM_01370770 | Other | E1 |  |
| 23446 | TTHERM_01371770 | Other | E1 |  |
| 23447 | TTHERM_01371780 | Other | E1 |  |
| 23450 | TTHERM_00391230 | Other | E1 |  |
| 24612 | TTHERM_01446960 | Other | E1 |  |
| 24905 | TTHERM_00390090 | Other | E1 |  |
| 25435 | TTHERM_00734120 | Other | E1 |  |
| 26270 | TTHERM_00751030 | Other | E1 |  |
| 28065 | TTHERM_01410000 | Other | E1 |  |
| 28605 | TTHERM_00370890 | Other | E1 |  |
| 29766 | TTHERM_00170580 | Other | E1 |  |
| 29767 | TTHERM_00170590 | Other | E1 |  |
| 1090 | TTHERM_00670660 | Other | E2 | E2a |
| 2256 | TTHERM_00474550 | Other | E2 | E2a |
| 2623 | TTHERM_01015890 | Other | E2 | E2a |
| 3709 | TTHERM_00656150 | Other | E2 | E2a |
| 4137 | TTHERM_00585310 | Other | E2 | E2a |
| 4750 | TTHERM_00277070 | Other | E2 | E2a |
| 6436 | TTHERM_00526980 | Other | E2 | E2a |
| 7304 | TTHERM_00729030 | Other | E2 | E2a |
| 8030 | TTHERM_00500860 | Other | E2 | E2a |
| 9801 | TTHERM_00266660 | Other | E2 | E2a |
| 10066 | TTHERM_00077200 | Other | E2 | E2a |
| 10118 | TTHERM_00077720 | Other | E2 | E2a |
| 10195 | TTHERM_00079490 | Other | E2 | E2a |
| 11510 | TTHERM_00000020 | Other | E2 | E2a |
| 11511 | TTHERM_00000030 | Other | E2 | E2a |
| 12415 | TTHERM_00523000 | Other | E2 | E2a |
| 13229 | TTHERM_00069390 | Other | E2 | E2a |
| 14827 | TTHERM_01227720 | Other | E2 | E2a |
| 15541 | TTHERM_00293310 | Other | E2 | E2a |
| 17681 | TTHERM_01049230 | Other | E2 | E2a |
| 23204 | TTHERM_00690080 | Other | E2 | E2a |
| 23205 | TTHERM_00690090 | Other | E2 | E2a |
| 23956 | TTHERM_00668030 | Other | E2 | E2a |
| 27560 | TTHERM_00355740 | Other | E2 | E2a |
| 27680 | TTHERM_01071490 | Other | E2 | E2a |
| 5330 | TTHERM_00351160 | Other | E2 | E2b |
| 5677 | TTHERM_00442770 | Other | E2 | E2b |
| 6653 | TTHERM_00193700 | Other | E2 | E2b |
| 8041 | TTHERM_00500970 | Other | E2 | E2b |
| 11132 | TTHERM_00046530 | Other | E2 | E2b |
| 11603 | TTHERM_00005920 | Other | E2 | E2b |
| 11605 | TTHERM_00005940 | Other | E2 | E2b |
| 11833 | TTHERM_00013270 | Other | E2 | E2b |
| 18124 | TTHERM_00723640 | Other | E2 | E2b |
| 23052 | TTHERM_00105520 | Other | E2 | E2b |
| 28438 | TTHERM_00158480 | Other | E2 | E2b |
| 2332 | TTHERM_00475330 | Other | E2 | E2c |
| 9454 | TTHERM_00128900 | Other | E2 | E2c |
| 19629 | TTHERM_00486980 | Other | E2 | E2c |
| 19630 | TTHERM_00486990 | Other | E2 | E2c |
| 20139 | TTHERM_00703630 | Other | E2 | E2c |
| 20144 | TTHERM_00703680 | Other | E2 | E2c |
| 21266 | TTHERM_00307630 | Other | E2 | E2c |
| 28437 | TTHERM_00158470 | Other | E2 | E2c |
| 2224 | TTHERM_00473240 | Other | E2 |  |
| 3655 | TTHERM_00655570 | Other | E2 |  |
| 3710 | TTHERM_00656160 | Other | E2 |  |
| 3995 | TTHERM_00825310 | Other | E2 |  |
| 4069 | TTHERM_00584610 | Other | E2 |  |
| 4090 | TTHERM_00584820 | Other | E2 |  |
| 4439 | TTHERM_00773480 | Other | E2 |  |
| 4723 | TTHERM_00530600 | Other | E2 |  |
| 5444 | TTHERM_00438870 | Other | E2 |  |
| 5498 | TTHERM_00440430 | Other | E2 |  |
| 5656 | TTHERM_00442560 | Other | E2 |  |
| 5692 | TTHERM_00442920 | Other | E2 |  |
| 5729 | TTHERM_01513280 | Other | E2 |  |
| 6435 | TTHERM_00526970 | Other | E2 |  |
| 7231 | TTHERM_00421070 | Other | E2 |  |
| 7312 | TTHERM_00729110 | Other | E2 |  |
| 7413 | TTHERM_00624250 | Other | E2 |  |
| 7935 | TTHERM_00558380 | Other | E2 |  |
| 8635 | TTHERM_00188850 | Other | E2 |  |
| 8897 | TTHERM_01251240 | Other | E2 |  |
| 9999 | TTHERM_00075550 | Other | E2 |  |
| 10107 | TTHERM_00077610 | Other | E2 |  |
| 10523 | TTHERM_00571930 | Other | E2 |  |
| 10685 | TTHERM_00509000 | Other | E2 |  |
| 10871 | TTHERM_00717760 | Other | E2 |  |
| 11236 | TTHERM_00047570 | Other | E2 |  |
| 11509 | TTHERM_00000010 | Other | E2 |  |
| 12851 | TTHERM_00653640 | Other | E2 |  |
| 13486 | TTHERM_00369640 | Other | E2 |  |
| 15045 | TTHERM_00547930 | Other | E2 |  |
| 15686 | TTHERM_00295760 | Other | E2 |  |
| 15767 | TTHERM_01001480 | Other | E2 |  |
| 16220 | TTHERM_00841360 | Other | E2 |  |
| 17684 | TTHERM_01049260 | Other | E2 |  |
| 18188 | TTHERM_00684750 | Other | E2 |  |
| 19068 | TTHERM_00715960 | Other | E2 |  |
| 19112 | TTHERM_00716400 | Other | E2 |  |
| 19522 | TTHERM_00485900 | Other | E2 |  |
| 19573 | TTHERM_00486420 | Other | E2 |  |
| 19962 | TTHERM_00534050 | Other | E2 |  |
| 20262 | TTHERM_00455330 | Other | E2 |  |
| 20331 | TTHERM_00457010 | Other | E2 |  |
| 20456 | TTHERM_00823880 | Other | E2 |  |
| 20643 | TTHERM_00148840 | Other | E2 |  |
| 20748 | TTHERM_00149880 | Other | E2 |  |
| 22235 | TTHERM_00216100 | Other | E2 |  |
| 22340 | TTHERM_00219120 | Other | E2 |  |
| 22504 | TTHERM_00470900 | Other | E2 |  |
| 22937 | TTHERM_00101380 | Other | E2 |  |
| 24195 | TTHERM_01006380 | Other | E2 |  |
| 24302 | TTHERM_00134780 | Other | E2 |  |
| 24495 | TTHERM_00139680 | Other | E2 |  |
| 26140 | TTHERM_00343600 | Other | E2 |  |
| 26746 | TTHERM_00145380 | Other | E2 |  |
| 27742 | TTHERM_01017250 | Other | E2 |  |
| 27964 | TTHERM_00632950 | Other | E2 |  |
| 28547 | TTHERM_00161570 | Other | E2 |  |
| 28647 | TTHERM_00371310 | Other | E2 |  |
| 28946 | TTHERM_00575560 | Other | E2 |  |
| 29162 | TTHERM_00865320 | Other | E2 |  |
| 5140 | TTHERM_00621430 | Other | E3 |  |
| 5142 | TTHERM_00621450 | Other | E3 |  |
| 7517 | TTHERM_01194800 | Other | E3 |  |
| 9015 | TTHERM_00566750 | Other | E3 |  |
| 10582 | TTHERM_00641130 | Other | E3 |  |
| 14039 | TTHERM_00426040 | Other | E3 |  |
| 3011 | TTHERM_00691730 | Other | E4 |  |
| 5933 | TTHERM_01084140 | Other | E4 |  |
| 20648 | TTHERM_00148890 | Other | E4 |  |
| 24353 | TTHERM_00136280 | Other | E4 |  |
| 10110 | TTHERM_00077640 | Other | E5 |  |
| 13023 | TTHERM_00058380 | Other | E5 |  |
| 13025 | TTHERM_00058400 | Other | E5 |  |
| 13026 | TTHERM_00058410 | Other | E5 |  |
| 13028 | TTHERM_00058430 | Other | E5 |  |
| 13029 | TTHERM_00058440 | Other | E5 |  |
| 13030 | TTHERM_00058450 | Other | E5 |  |
| 13033 | TTHERM_00058480 | Other | E5 |  |
| 19549 | TTHERM_00486180 | Other | E5 |  |
| 4866 | TTHERM_00283270 | Other | E6 |  |
| 6544 | TTHERM_00630300 | Other | E6 |  |
| 9395 | TTHERM_00128310 | Other | E6 |  |
| 10501 | TTHERM_00571710 | Other | E6 |  |
| 14484 | TTHERM_00037490 | Other | E6 |  |
| 14485 | TTHERM_00037500 | Other | E6 |  |
| 14879 | TTHERM_00699830 | Other | E6 |  |
| 18715 | TTHERM_00469130 | Other | E6 |  |
| 23558 | TTHERM_00393350 | Other | E6 |  |
| 26506 | TTHERM_00433650 | Other | E6 |  |
| 26507 | TTHERM_00433660 | Other | E6 |  |
| 13784 | TTHERM_00204110 | Other | E7 |  |
| 17883 | TTHERM_01083090 | Other | E7 |  |
| 27893 | TTHERM_00561320 | Other | E7 |  |
| 27894 | TTHERM_00561330 | Other | E7 |  |
| 5505 | TTHERM_00440500 | Other | E8 |  |
| 5506 | TTHERM_00440510 | Other | E8 |  |
| 22407 | TTHERM_00220790 | Other | E8 |  |
| 7212 | TTHERM_00420870 | Other | E9 |  |
| 9974 | TTHERM_00074310 | Other | E9 |  |
| 15671 | TTHERM_00295610 | Other | E9 |  |
| 6855 | TTHERM_00495900 | Other | F1 |  |
| 29238 | TTHERM_00554310 | Other | F1 |  |
| 2548 | TTHERM_00248520 | Other | F2 |  |
| 29450 | TTHERM_00264840 | Other | F2 |  |
| 10577 | TTHERM_00641080 | Other | IRE |  |
| 22589 | TTHERM_00471810 | Other | NAK |  |
| 26298 | TTHERM_00753300 | Other | NAK |  |
| 1405 | TTHERM_01144900 | Other | NEK |  |
| 1924 | TTHERM_01114180 | Other | NEK |  |
| 2122 | TTHERM_00657380 | Other | NEK |  |
| 2218 | TTHERM_00473180 | Other | NEK |  |
| 2469 | TTHERM_00245740 | Other | NEK |  |
| 3425 | TTHERM_00312380 | Other | NEK |  |
| 3979 | TTHERM_00825150 | Other | NEK |  |
| 4647 | TTHERM_00529780 | Other | NEK |  |
| 6348 | TTHERM_00525110 | Other | NEK |  |
| 7740 | TTHERM_00821800 | Other | NEK |  |
| 8589 | TTHERM_00188390 | Other | NEK |  |
| 10831 | TTHERM_01030010 | Other | NEK |  |
| 13547 | TTHERM_00415780 | Other | NEK |  |
| 15435 | TTHERM_00449170 | Other | NEK |  |
| 15734 | TTHERM_01000150 | Other | NEK |  |
| 17542 | TTHERM_00697500 | Other | NEK |  |
| 17999 | TTHERM_01027700 | Other | NEK |  |
| 18448 | TTHERM_00721400 | Other | NEK |  |
| 18676 | TTHERM_00467740 | Other | NEK |  |
| 19380 | TTHERM_00301840 | Other | NEK |  |
| 19674 | TTHERM_00947540 | Other | NEK |  |
| 20296 | TTHERM_00455670 | Other | NEK |  |
| 20809 | TTHERM_00151480 | Other | NEK |  |
| 21625 | TTHERM_00412070 | Other | NEK |  |
| 21626 | TTHERM_00412080 | Other | NEK |  |
| 22242 | TTHERM_00216170 | Other | NEK |  |
| 23433 | TTHERM_00809380 | Other | NEK |  |
| 24670 | TTHERM_00628440 | Other | NEK |  |
| 24902 | TTHERM_00390060 | Other | NEK |  |
| 24991 | TTHERM_00997660 | Other | NEK |  |
| 26810 | TTHERM_00146030 | Other | NEK |  |
| 27114 | TTHERM_00113000 | Other | NEK |  |
| 28433 | TTHERM_00158430 | Other | NEK |  |
| 28502 | TTHERM_00161100 | Other | NEK |  |
| 29340 | TTHERM_00260770 | Other | NEK |  |
| 29341 | TTHERM_00260780 | Other | NEK |  |
| 29473 | TTHERM_00265180 | Other | NEK |  |
| 29897 | TTHERM_00185750 | Other | NEK |  |
| 11434 | TTHERM_00052540 | Other | PEK | GCN2 |
| 18670 | TTHERM_00467680 | Other | PEK | GCN2 |
| 21870 | TTHERM_00726040 | Other | PEK | GCN2 |
| 6287 | TTHERM_00483530 | Other | PEK | Unclassified |
| 5034 | TTHERM_01046870 | Other | PLK |  |
| 8238 | TTHERM_01076990 | Other | PLK |  |
| 8829 | TTHERM_00191790 | Other | PLK |  |
| 18358 | TTHERM_00992970 | Other | PLK |  |
| 18380 | TTHERM_01443850 | Other | PLK |  |
| 21183 | TTHERM_00923040 | Other | PLK |  |
| 22503 | TTHERM_00470890 | Other | PLK |  |
| 3125 | TTHERM_00535400 | Other | SCY1 |  |
| 4269 | TTHERM_00494350 | Other | TLK |  |
| 19858 | TTHERM_00532000 | Other | TLK |  |
| 1348 | TTHERM_00790580 | Other | ULK |  |
| 1349 | TTHERM_00790590 | Other | ULK |  |
| 1429 | TTHERM_00969600 | Other | ULK |  |
| 1439 | TTHERM_00971680 | Other | ULK |  |
| 1647 | TTHERM_01345780 | Other | ULK |  |
| 2103 | TTHERM_01080610 | Other | ULK |  |
| 2836 | TTHERM_01100450 | Other | ULK |  |
| 3032 | TTHERM_00691940 | Other | ULK |  |
| 3104 | TTHERM_00535190 | Other | ULK |  |
| 3833 | TTHERM_00444650 | Other | ULK |  |
| 4111 | TTHERM_00585040 | Other | ULK |  |
| 5362 | TTHERM_00353490 | Other | ULK |  |
| 5380 | TTHERM_00436250 | Other | ULK |  |
| 5688 | TTHERM_00442880 | Other | ULK |  |
| 6347 | TTHERM_00525100 | Other | ULK |  |
| 6603 | TTHERM_00193200 | Other | ULK |  |
| 7377 | TTHERM_00622900 | Other | ULK |  |
| 8181 | TTHERM_00758840 | Other | ULK |  |
| 8636 | TTHERM_00188860 | Other | ULK |  |
| 8696 | TTHERM_00189460 | Other | ULK |  |
| 9457 | TTHERM_00128930 | Other | ULK |  |
| 10447 | TTHERM_00569170 | Other | ULK |  |
| 11042 | TTHERM_01284760 | Other | ULK |  |
| 11253 | TTHERM_00047740 | Other | ULK |  |
| 11660 | TTHERM_00007510 | Other | ULK |  |
| 13838 | TTHERM_00213560 | Other | ULK |  |
| 14572 | TTHERM_00040360 | Other | ULK |  |
| 15010 | TTHERM_00543620 | Other | ULK |  |
| 15044 | TTHERM_00547920 | Other | ULK |  |
| 15069 | TTHERM_00548280 | Other | ULK |  |
| 15675 | TTHERM_00295650 | Other | ULK |  |
| 15705 | TTHERM_00295950 | Other | ULK |  |
| 15762 | TTHERM_01001430 | Other | ULK |  |
| 15836 | TTHERM_00647300 | Other | ULK |  |
| 15837 | TTHERM_00647310 | Other | ULK |  |
| 16588 | TTHERM_00094330 | Other | ULK |  |
| 18315 | TTHERM_00756520 | Other | ULK |  |
| 18701 | TTHERM_00468000 | Other | ULK |  |
| 18780 | TTHERM_00237360 | Other | ULK |  |
| 19558 | TTHERM_00486270 | Other | ULK |  |
| 19707 | TTHERM_00637040 | Other | ULK |  |
| 20769 | TTHERM_00151080 | Other | ULK |  |
| 20902 | TTHERM_00314920 | Other | ULK |  |
| 21109 | TTHERM_00775960 | Other | ULK |  |
| 21913 | TTHERM_00726480 | Other | ULK |  |
| 22048 | TTHERM_00599810 | Other | ULK |  |
| 22049 | TTHERM_00599820 | Other | ULK |  |
| 24363 | TTHERM_00136380 | Other | ULK |  |
| 24887 | TTHERM_00389910 | Other | ULK |  |
| 24896 | TTHERM_00390000 | Other | ULK |  |
| 26436 | TTHERM_00429960 | Other | ULK |  |
| 26742 | TTHERM_00145340 | Other | ULK |  |
| 27664 | TTHERM_01070340 | Other | ULK |  |
| 28079 | TTHERM_01107300 | Other | ULK |  |
| 28933 | TTHERM_00575430 | Other | ULK |  |
| 29387 | TTHERM_00263220 | Other | ULK |  |
| 30213 | TTHERM_00327100 | Other | ULK |  |
| 1648 | TTHERM_01345790 | Other | Unique |  |
| 4546 | TTHERM_01179770 | Other | Unique |  |
| 8550 | TTHERM_00787230 | Other | Unique |  |
| 8760 | TTHERM_00191090 | Other | Unique |  |
| 9187 | TTHERM_00648730 | Other | Unique |  |
| 9404 | TTHERM_00128400 | Other | Unique |  |
| 10356 | TTHERM_00379020 | Other | Unique |  |
| 10969 | TTHERM_00672230 | Other | Unique |  |
| 11313 | TTHERM_00049340 | Other | Unique |  |
| 12278 | TTHERM_00856510 | Other | Unique |  |
| 13600 | TTHERM_00418310 | Other | Unique |  |
| 14663 | TTHERM_00886980 | Other | Unique |  |
| 15034 | TTHERM_00545840 | Other | Unique |  |
| 21877 | TTHERM_00726110 | Other | Unique |  |
| 23577 | TTHERM_00394530 | Other | Unique |  |
| 25339 | TTHERM_00254550 | Other | Unique |  |
| 27115 | TTHERM_00113010 | Other | Unique |  |
| 27542 | TTHERM_00355560 | Other | Unique |  |
| 15015 | TTHERM_00543670 | Other | VPS15 |  |
| 18894 | TTHERM_00241540 | Other | Wee |  |
| 26815 | TTHERM_00146080 | Other | Wee |  |
| 8961 | TTHERM_00564230 | Other | Wnk |  |
| 17822 | TTHERM_00840070 | Other | Wnk |  |
| 4322 | TTHERM_01142670 | STE | STE11 |  |
| 6622 | TTHERM_00193390 | STE | STE11 |  |
| 7147 | TTHERM_00420220 | STE | STE11 |  |
| 11359 | TTHERM_00051780 | STE | STE11 |  |
| 15684 | TTHERM_00295740 | STE | STE11 |  |
| 18758 | TTHERM_01502010 | STE | STE11 |  |
| 23105 | TTHERM_00107120 | STE | STE11 |  |
| 29763 | TTHERM_00170550 | STE | STE11 |  |
| 1462 | TTHERM_00971920 | STE | STE20 | MST |
| 5838 | TTHERM_00580440 | STE | STE20 | MST |
| 17909 | TTHERM_00933100 | STE | STE20 | MST |
| 29596 | TTHERM_01246760 | STE | STE20 | MST |
| 19075 | TTHERM_00716030 | STE | STE20 | PAKA |
| 25909 | TTHERM_00286790 | STE | STE20 | PAKA |
| 17911 | TTHERM_00933120 | STE | STE20 | YSK |
| 1557 | TTHERM_01298440 | STE | STE7 |  |
| 1909 | TTHERM_01111050 | STE | STE7 |  |
| 12277 | TTHERM_00856500 | STE | STE7 |  |
| 26687 | TTHERM_00143800 | STE | STE7 |  |
| 5755 | TTHERM_00578620 | TKL | TKL |  |
| 6778 | TTHERM_00195930 | TKL | TKL |  |
| 12330 | TTHERM_00522150 | TKL | TKL |  |
| 14057 | TTHERM_00426220 | TKL | TKL |  |
| 26581 | TTHERM_00140760 | TKL | TKL |  |

⨪

B. ID numbers for all *T. thermophila* membrane transporter-encoding genes referred to in Table 6, with their families and predicted substrates.

| **Preliminary TIGR ID** | **GB_ACCESSION** | **Transporter family** | **Predicted Substrate/function** |
| --- | --- | --- | --- |
| 1076 | TTHERM_00670520 | VIC | potassium ion channel |
| 1077 | TTHERM_00670530 | VIC | potassium ion channel |
| 1178 | TTHERM_00852790 | MFS | organic cation/carnitine |
| 1263 | TTHERM_00780510 | P-ATPase | calcium ion |
| 1353 | TTHERM_00790630 | VIC | potassium ion channel |
| 1354 | TTHERM_00790640 | VIC | potassium ion channel |
| 1356 | TTHERM_00790660 | VIC | potassium ion channel |
| 1448 | TTHERM_00971780 | ENT | nucleoside |
| 1679 | TTHERM_01262810 | VIC | potassium ion channel |
| 1704 | TTHERM_01127440 | CPA1 | sodium ion/proton |
| 1706 | TTHERM_01127460 | CPA1 | sodium ion/proton |
| 1707 | TTHERM_01127470 | CPA1 | sodium ion/proton |
| 1708 | TTHERM_01127480 | CPA1 | sodium ion/proton |
| 1711 | TTHERM_01127510 | CPA1 | sodium ion/proton |
| 1712 | TTHERM_01127520 | CPA1 | sodium ion/proton |
| 1834 | TTHERM_02139630 | MC | ADP/ATP |
| 1970 | TTHERM_01092340 | MOP | multidrug efflux |
| 2086 | TTHERM_01080440 | Hsp70 | heat shock protein 70 homolog |
| 2087 | TTHERM_01080450 | MFS | ? |
| 2134 | TTHERM_00657500 | VIC | potassium ion channel |
| 2220 | TTHERM_00473200 | MFS | hexose |
| 2222 | TTHERM_00473220 | P-ATPase | phospholipid (fused with guanylyl cyclase) |
| 2258 | TTHERM_00474570 | MC | ? |
| 2267 | TTHERM_00474670 | MC | aspartate/glutamate (Ca2+activated) |
| 2272 | TTHERM_00474720 | P-ATPase | phospholipid |
| 2363 | TTHERM_00243670 | ABC | multispecific organic anion/multidrug (ABCC) |
| 2397 | TTHERM_00245010 | VIC | potassium ion channel |
| 2406 | TTHERM_00245100 | P-ATPase | sodium/potassium ion |
| 2417 | TTHERM_00245210 | GPH | sugar |
| 2419 | TTHERM_00245230 | MFS | oxalate/formate? |
| 2421 | TTHERM_00245250 | MFS | oxalate/formate? |
| 2423 | TTHERM_00245270 | MFS | oxalate/formate? |
| 2424 | TTHERM_00245280 | MFS | oxalate/formate? |
| 2425 | TTHERM_00245290 | MFS | oxalate/formate? |
| 2426 | TTHERM_00245310 | VIC | potassium ion channel |
| 2432 | TTHERM_00245370 | VIC | potassium ion channel |
| 2433 | TTHERM_00245380 | VIC | potassium ion channel |
| 2435 | TTHERM_00245400 | VIC | potassium ion channel |
| 2468 | TTHERM_00245730 | MFS | feline leukemia virus subgroup C receptor FLVCR homolog |
| 2555 | TTHERM_01337370 | VIC | potassium ion channel |
| 2651 | TTHERM_01016170 | VIC | potassium ion channel |
| 2724 | TTHERM_01205340 | RIR-CaC | IP3 receptor calcium ion channel |
| 2737 | TTHERM_01417320 | ABC | ? (ABCA) |
| 2766 | TTHERM_00732450 | MFS | feline leukemia virus subgroup C receptor FLVCR homolog |
| 2842 | TTHERM_01100510 | MOP | multidrug efflux |
| 2843 | TTHERM_01100520 | MFS | oxalate/formate |
| 2844 | TTHERM_01100530 | MOP | multidrug efflux |
| 2845 | TTHERM_01100540 | MOP | multidrug efflux |
| 2911 | TTHERM_00693280 | ABC | ? (ABCA) |
| 2912 | TTHERM_00693290 | ABC | ? (ABCA) |
| 2913 | TTHERM_00693300 | ABC | ? (ABCA) |
| 2914 | TTHERM_00694300 | ABC | ? (ABCA) |
| 2915 | TTHERM_00694310 | ABC | ? (ABCA) |
| 2948 | TTHERM_00691090 | DMT | drug/metabolite? |
| 2966 | TTHERM_00691280 | VIC | potassium ion channel |
| 3010 | TTHERM_00691720 | Nramp | metal ion |
| 3018 | TTHERM_00691800 | MFS | hexose |
| 3023 | TTHERM_00691850 | MC | ? |
| 3040 | TTHERM_01123860 | P-ATPase | phospholipid |
| 3097 | TTHERM_00535120 | MFS | hexose |
| 3156 | TTHERM_00535710 | P-ATPase | calcium ion |
| 3158 | TTHERM_00535730 | MC | phosphate |
| 3159 | TTHERM_00535740 | MC | phosphate |
| 3182 | TTHERM_00535970 | ABC | ? (ABCA) |
| 3187 | TTHERM_00537010 | VIC | potassium ion channel |
| 3207 | TTHERM_00537210 | AAAP | amino acid |
| 3208 | TTHERM_00537220 | AAAP | amino acid |
| 3276 | TTHERM_00762850 | RIR-CaC | IP3 receptor calcium ion channel |
| 3277 | TTHERM_00762860 | RIR-CaC | IP3 receptor calcium ion channel |
| 3417 | TTHERM_00312300 | VIC | potassium ion channel |
| 3470 | TTHERM_00312830 | VIC | potassium ion channel |
| 3471 | TTHERM_00312840 | VIC | potassium ion channel |
| 3473 | TTHERM_00312860 | VIC | potassium ion channel |
| 3524 | TTHERM_00313370 | CaCA | sodium ion:calcium/potassium ion |
| 3525 | TTHERM_00313380 | CaCA | sodium ion:calcium/potassium ion |
| 3678 | TTHERM_00655840 | CDF | zinc ion efflux |
| 3727 | TTHERM_00938770 | CDF | zinc ion efflux |
| 3787 | TTHERM_00444190 | AAAP | amino acid |
| 3798 | TTHERM_00444300 | VIC | potassium ion channel |
| 3808 | TTHERM_00444400 | VIC | potassium ion channel |
| 3874 | TTHERM_00446050 | CPA1 | sodium ion/proton |
| 4014 | TTHERM_00825500 | AAAP | amino acid |
| 4062 | TTHERM_00584540 | VIC | potassium ion channel |
| 4097 | TTHERM_00584900 | ABC | multidrug (ABCC) |
| 4118 | TTHERM_00585120 | VIC | potassium ion channel |
| 4132 | TTHERM_00585260 | F-ATPase | protons (mitochondrial) |
| 4148 | TTHERM_00585420 | Annexin | Annexin VII homolog |
| 4155 | TTHERM_00585490 | VIC | potassium ion channel |
| 4289 | TTHERM_00494610 | VIC | potassium ion channel |
| 4299 | TTHERM_00494710 | MC | uncoupling protein homolog |
| 4300 | TTHERM_00494720 | MC | 2-oxoglutarate/malate |
| 4415 | TTHERM_00773240 | VIC | potassium ion channel |
| 4433 | TTHERM_00773420 | ABC | White protein homolog (ABCG) |
| 4453 | TTHERM_00773620 | MFS | feline leukemia virus subgroup C receptor FLVCR homolog |
| 4454 | TTHERM_00773630 | MFS | feline leukemia virus subgroup C receptor FLVCR homolog |
| 4455 | TTHERM_00773640 | MFS | feline leukemia virus subgroup C receptor FLVCR homolog |
| 4472 | TTHERM_00773810 | H+PPase | proton |
| 4494 | TTHERM_01248990 | MFS | organic cation |
| 4544 | TTHERM_01179750 | MFS | oxalate/formate? |
| 4548 | TTHERM_01179790 | VIC | potassium ion channel |
| 4585 | TTHERM_01014550 | MC | ? |
| 4601 | TTHERM_01014710 | F-ATPase | protons (vacuolar) |
| 4604 | TTHERM_01014740 | Hsp70 | heat shock protein 70 homolog |
| 4605 | TTHERM_01014750 | Hsp70 | heat shock protein 70 homolog |
| 4616 | TTHERM_00529470 | ABC | ? (ABCA) |
| 4655 | TTHERM_00529860 | F-ATPase | protons (vacuolar) |
| 4661 | TTHERM_00529920 | P-ATPase | copper ion |
| 4662 | TTHERM_00529930 | P-ATPase | copper ion |
| 4736 | TTHERM_00530730 | MC | ? |
| 4758 | TTHERM_00277150 | AAAP | amino acid |
| 4759 | TTHERM_00277160 | AAAP | amino acid |
| 4825 | TTHERM_00279850 | VIC | potassium ion channel |
| 4829 | TTHERM_00279900 | VIC | potassium ion channel/adenylyl cyclase fusion protein |
| 4831 | TTHERM_00279920 | VIC | potassium ion channel/adenylyl cyclase fusion protein |
| 4855 | TTHERM_00283160 | ABC | multidrug (ABCC) |
| 4901 | TTHERM_00283620 | MFS | multidrug efflux? |
| 4933 | TTHERM_00283950 | DMT | drug/metabolite? |
| 4950 | TTHERM_00284120 | MFS | organic cation |
| 5038 | TTHERM_01046910 | ENT | nucleoside |
| 5125 | TTHERM_00621280 | CPA1 | sodium ion/proton |
| 5127 | TTHERM_00621300 | Annexin | Annexin VII homolog |
| 5157 | TTHERM_00621600 | MC | 2-oxoglutarate/malate |
| 5219 | TTHERM_00348070 | VIC | potassium ion channel |
| 5298 | TTHERM_00348860 | VIC | potassium ion channel |
| 5328 | TTHERM_00351140 | Hsp70 | heat shock protein 70 homolog |
| 5425 | TTHERM_00437690 | MFS | organic cation |
| 5432 | TTHERM_00437760 | MFS | organic anion |
| 5433 | TTHERM_00437770 | MFS | organic cation |
| 5438 | TTHERM_00438810 | DMT | drug/metabolite? |
| 5453 | TTHERM_00438960 | VIC | potassium ion channel |
| 5454 | TTHERM_00438970 | VIC | potassium ion channel |
| 5456 | TTHERM_00438990 | VIC | potassium ion channel |
| 5477 | TTHERM_00439200 | MFS | feline leukemia virus subgroup C receptor FLVCR homolog |
| 5479 | TTHERM_00439220 | MFS | feline leukemia virus subgroup C receptor FLVCR homolog |
| 5518 | TTHERM_00440640 | ABC | multidrug (ABCC) |
| 5520 | TTHERM_00440660 | ABC | multidrug (ABCC) |
| 5543 | TTHERM_01009850 | ABC | multidrug (ABCC) |
| 5582 | TTHERM_00441800 | VIC | potassium ion channel |
| 5599 | TTHERM_00441990 | AAAP | amino acid |
| 5628 | TTHERM_00442280 | VIC | potassium ion channel |
| 5689 | TTHERM_00442890 | P-ATPase | phospholipid (fused with guanylyl cyclase) |
| 5690 | TTHERM_00442900 | P-ATPase | phospholipid (fused with guanylyl cyclase) |
| 5711 | TTHERM_00443110 | MFS | glycerol 3-phosphate |
| 5797 | TTHERM_00579040 | PCC | Polycystin 2 homolog |
| 5830 | TTHERM_00580360 | ABC | White protein homolog (ABCG) |
| 5861 | TTHERM_00951710 | ABC | multidrug (ABCC) |
| 5863 | TTHERM_00951730 | ABC | multispecific organic anion/multidrug (ABCC) |
| 5867 | TTHERM_00951770 | ABC | multidrug/bile salt (ABCB) |
| 5870 | TTHERM_00951800 | ABC | multidrug/bile salt (ABCB) |
| 5899 | TTHERM_01044390 | Hsp70 | heat shock protein 70 homolog |
| 5951 | TTHERM_01084320 | ABC | multidrug (P-glycoprotein homolog) (ABCB) |
| 5955 | TTHERM_01084370 | P-ATPase | calcium ion |
| 6005 | TTHERM_00334610 | VIC | potassium ion channel |
| 6105 | TTHERM_00339640 | F-ATPase | protons (vacuolar) |
| 6162 | TTHERM_00340210 | MFS | organic cation/carnitine |
| 6219 | TTHERM_00476910 | ABC | ? (ABCA) |
| 6227 | TTHERM_00476990 | ABC | ? (ABCA) |
| 6238 | TTHERM_00479080 | ABC | multidrug (ABCC) |
| 6256 | TTHERM_00481240 | VIC | potassium ion channel |
| 6293 | TTHERM_00483590 | MPT | inner membrane translocase (Tim17) |
| 6336 | TTHERM_01353180 | VIC | potassium ion channel |
| 6337 | TTHERM_01353190 | VIC | potassium ion channel |
| 6339 | TTHERM_01353210 | VIC | potassium ion channel |
| 6356 | TTHERM_00526180 | ABC | multidrug (ABCC) |
| 6411 | TTHERM_00526730 | AAAP | amino acid |
| 6413 | TTHERM_00526750 | VIC | potassium ion channel |
| 6430 | TTHERM_00526920 | CHR | chromate ion |
| 6569 | TTHERM_00630560 | ABC | multidrug (ABCC) |
| 6571 | TTHERM_00630580 | ABC | multidrug (ABCC) |
| 6649 | TTHERM_00193660 | F-ATPase | protons (vacuolar) |
| 6702 | TTHERM_00194180 | VIC | potassium ion channel |
| 6704 | TTHERM_00194200 | VIC | potassium ion channel |
| 6721 | TTHERM_00194370 | RIR-CaC | IP3 receptor calcium ion channel |
| 6779 | TTHERM_00195940 | VIC | potassium ion channel |
| 6780 | TTHERM_00195950 | VIC | potassium ion channel |
| 6781 | TTHERM_00195960 | VIC | potassium ion channel |
| 6872 | TTHERM_00497060 | CDF | zinc ion efflux |
| 6875 | TTHERM_00497090 | CDF | heavy metal ion efflux |
| 6924 | TTHERM_00497590 | PiT | phosphate |
| 6947 | TTHERM_00497820 | VIC | potassium ion channel/adenylyl cyclase fusion protein |
| 6955 | TTHERM_00497900 | P-ATPase | phospholipid |
| 7004 | TTHERM_01156820 | P-ATPase | phospholipid (fused with guanylyl cyclase) |
| 7005 | TTHERM_01156830 | P-ATPase | phospholipid (fused with guanylyl cyclase) |
| 7027 | TTHERM_01338460 | VIC | potassium ion channel |
| 7029 | TTHERM_01338480 | VIC | potassium ion channel |
| 7031 | TTHERM_01338500 | VIC | potassium ion channel |
| 7051 | TTHERM_00919550 | ABC | ? (ABCA) |
| 7052 | TTHERM_00919560 | ABC | ? (ABCA) |
| 7057 | TTHERM_00919610 | VIC | potassium ion channel |
| 7102 | TTHERM_00419720 | GUP | glycerol |
| 7105 | TTHERM_00419750 | VIC | potassium ion channel |
| 7127 | TTHERM_00420020 | MC | carnitine/acylcarnitine |
| 7148 | TTHERM_00420230 | RIR-CaC | IP3 receptor calcium ion channel |
| 7163 | TTHERM_00420380 | VIC | potassium ion channel |
| 7166 | TTHERM_00420410 | ABC | TAP-family protein (ABCB) |
| 7207 | TTHERM_00420820 | VIC | small-conductance calcium-activated potassium ion channel |
| 7213 | TTHERM_00420880 | P-ATPase | sodium/potassium ion |
| 7215 | TTHERM_00420900 | P-ATPase | sodium/potassium ion |
| 7274 | TTHERM_00727730 | AAAP | amino acid |
| 7293 | TTHERM_00728910 | ABC | ? (ABCA) |
| 7447 | TTHERM_00624590 | ABC | White protein homolog (ABCG) |
| 7452 | TTHERM_00624640 | VIC | potassium ion channel |
| 7463 | TTHERM_00624750 | MFS | feline leukemia virus subgroup C receptor FLVCR homolog |
| 7464 | TTHERM_00624760 | ABC | multidrug (ABCC) |
| 7470 | TTHERM_00624820 | AAAP | amino acid |
| 7508 | TTHERM_01194710 | ABC | multidrug (ABCC) |
| 7595 | TTHERM_00678310 | NSS | sodium ion/GABA |
| 7596 | TTHERM_00678320 | NSS | sodium ion/proline |
| 7609 | TTHERM_00678450 | MFS | organic cation |
| 7624 | TTHERM_00678600 | AAAP | amino acid |
| 7682 | TTHERM_00862740 | ABC | ? (ABCA) |
| 7693 | TTHERM_01347930 | MFS | glycerol-3-phosphate |
| 7746 | TTHERM_00821870 | F-ATPase | protons (vacuolar) |
| 7780 | TTHERM_00822250 | MC | ADP/ATP |
| 7831 | TTHERM_00989410 | VIC | potassium ion channel |
| 7834 | TTHERM_00989440 | P-ATPase | sodium/potassium ion |
| 7881 | TTHERM_00557830 | VIC | potassium ion channel |
| 7892 | TTHERM_00557940 | VIC | potassium ion channel |
| 7918 | TTHERM_00558210 | VIC | potassium ion channel |
| 7919 | TTHERM_00558220 | ABC | multidrug (ABCC) |
| 7920 | TTHERM_00558230 | ABC | multidrug (ABCC) |
| 7941 | TTHERM_00558440 | Hsp70 | heat shock protein 70 homolog |
| 7949 | TTHERM_00558530 | VIC | potassium ion channel |
| 7990 | TTHERM_00499470 | P-ATPase | cation |
| 8036 | TTHERM_00500920 | Amt | CO2 (Rhesus glycoprotein homolog)? |
| 8100 | TTHERM_00502610 | ABC | multidrug (P-glycoprotein homolog) (ABCB) |
| 8102 | TTHERM_00502630 | ABC | multidrug/bile salt (ABCB) |
| 8119 | TTHERM_01068040 | Annexin | Annexin VII homolog |
| 8140 | TTHERM_01068280 | VIC | potassium ion channel |
| 8153 | TTHERM_00757570 | MFS | multidrug efflux |
| 8196 | TTHERM_00758990 | VIC | potassium ion channel |
| 8197 | TTHERM_00759000 | VIC | potassium ion channel |
| 8198 | TTHERM_00759010 | VIC | potassium ion channel |
| 8201 | TTHERM_00759040 | VIC | potassium ion channel |
| 8202 | TTHERM_00759050 | VIC | potassium ion channel |
| 8236 | TTHERM_01076970 | RIR-CaC | IP3 receptor calcium ion channel |
| 8408 | TTHERM_00491030 | MFS | organic anion |
| 8459 | TTHERM_00826810 | VIC | potassium ion channel |
| 8463 | TTHERM_00826850 | VIC | potassium ion channel |
| 8475 | TTHERM_00826980 | MFS | organic anion |
| 8511 | TTHERM_00785850 | VIC | calcium ion channel |
| 8514 | TTHERM_00785880 | ABC | multidrug (ABCC) |
| 8516 | TTHERM_00785900 | VIC | calcium ion channel |
| 8517 | TTHERM_00785910 | VIC | calcium ion channel |
| 8560 | TTHERM_00787330 | MFS | hexose |
| 8594 | TTHERM_00188440 | ABC | ? (ABCA) |
| 8597 | TTHERM_00188470 | MC | ? |
| 8600 | TTHERM_00188500 | MFS | glucose |
| 8624 | TTHERM_00188740 | VIC | potassium ion channel |
| 8660 | TTHERM_00189100 | ABC | ? (ABCA) |
| 8716 | TTHERM_00190650 | MFS | organic cation/carnitine |
| 8773 | TTHERM_00191220 | MFS | glycerol-3-phosphate |
| 8804 | TTHERM_00191530 | VIC | potassium ion channel |
| 8810 | TTHERM_00191600 | MFS | acetyl-CoA |
| 8813 | TTHERM_00191630 | MFS | hexose |
| 8845 | TTHERM_00191950 | MFS | organic cation/carnitine |
| 8846 | TTHERM_00191960 | MFS | organic cation/carnitine |
| 8857 | TTHERM_00192070 | VIC | potassium ion channel |
| 8876 | TTHERM_01149330 | P-ATPase | sodium/potassium ion |
| 8879 | TTHERM_01150350 | P-ATPase | sodium/potassium ion |
| 8927 | TTHERM_00563890 | CPA1 | sodium ion/proton |
| 8950 | TTHERM_00564120 | ABC | long-chain fatty acid (ALDP homolog) (ABCD) |
| 8965 | TTHERM_00564270 | VIC | potassium ion channel |
| 8970 | TTHERM_00564320 | CDF | zinc ion efflux |
| 9120 | TTHERM_01207670 | MFS | oxalate/formate? |
| 9121 | TTHERM_01207680 | MFS | oxalate/formate? |
| 9195 | TTHERM_00648810 | VIC | potassium ion channel |
| 9228 | TTHERM_00649140 | P-ATPase | phospholipid |
| 9235 | TTHERM_00649210 | MC | folate |
| 9250 | TTHERM_00649360 | ABC | ? (ABCA) |
| 9271 | TTHERM_00125090 | ABC | ? (ABCA) |
| 9276 | TTHERM_00125140 | RIR-CaC | IP3 receptor calcium ion channel |
| 9306 | TTHERM_00125440 | MFS | oxalate/formate? |
| 9321 | TTHERM_00125590 | MC | ADP/ATP |
| 9326 | TTHERM_00125640 | Hsp70 | heat shock protein 70 homolog |
| 9408 | TTHERM_00128440 | VIC | potassium ion channel |
| 9491 | TTHERM_00129390 | VIC | potassium ion channel |
| 9493 | TTHERM_00129410 | VIC | potassium ion channel |
| 9689 | TTHERM_00876930 | DMT | drug/metabolite? |
| 9691 | TTHERM_00876950 | DMT | drug/metabolite? |
| 9692 | TTHERM_00876960 | DMT | drug/metabolite? |
| 9792 | TTHERM_00266570 | F-ATPase | protons (vacuolar) |
| 9796 | TTHERM_00266610 | P-ATPase | sodium/potassium ion |
| 9837 | TTHERM_00268020 | MOP | multidrug efflux |
| 9844 | TTHERM_00268090 | P-ATPase | calcium ion |
| 9853 | TTHERM_00268190 | P-ATPase | sodium/potassium ion |
| 9934 | TTHERM_00276040 | MC | ? |
| 9976 | TTHERM_00074330 | VIC | potassium ion channel |
| 9977 | TTHERM_00074340 | VIC | potassium ion channel |
| 9990 | TTHERM_00075460 | VIC | potassium ion channel |
| 10053 | TTHERM_00077070 | VIC | potassium ion channel |
| 10054 | TTHERM_00077080 | MC | RNA splicing protein MRS4 homolog |
| 10093 | TTHERM_00077470 | VIC | potassium ion channel |
| 10094 | TTHERM_00077480 | VIC | potassium ion channel |
| 10096 | TTHERM_00077500 | VIC | potassium ion channel |
| 10097 | TTHERM_00077510 | VIC | potassium ion channel |
| 10138 | TTHERM_00078920 | CPA1 | sodium ion/proton |
| 10216 | TTHERM_00079710 | MFS | organic cation |
| 10302 | TTHERM_00378480 | VIC | low-voltage activated calcium ion channel |
| 10374 | TTHERM_00382170 | VIC | potassium ion channel |
| 10516 | TTHERM_00571860 | F-ATPase | protons (mitochondrial) |
| 10621 | TTHERM_00643510 | GIC | glutamate gated ion channel ionotropic kainate |
| 10625 | TTHERM_00643550 | P-ATPase | sodium/potassium ion |
| 10665 | TTHERM_00503850 | DMT | drug/metabolite? |
| 10682 | TTHERM_00508970 | P-ATPase | sodium/potassium ion |
| 10684 | TTHERM_00508990 | P-ATPase | sodium/potassium ion |
| 10704 | TTHERM_00510180 | CytB | NADPH oxidase homolog |
| 10706 | TTHERM_00512180 | CytB | NADPH oxidase homolog |
| 10707 | TTHERM_00513180 | CytB | NADPH oxidase homolog |
| 10709 | TTHERM_00513200 | CytB | NADPH oxidase homolog |
| 10710 | TTHERM_00513210 | CytB | NADPH oxidase homolog |
| 10711 | TTHERM_00514210 | CytB | NADPH oxidase homolog |
| 10712 | TTHERM_00515210 | CytB | NADH/NADPH thyroid oxidase homolog |
| 10716 | TTHERM_00515250 | MOP | multidrug efflux |
| 10718 | TTHERM_00515270 | CPA1 | sodium ion/proton |
| 10856 | TTHERM_00717600 | VIC | potassium ion channel |
| 10857 | TTHERM_00717610 | VIC | potassium ion channel |
| 10858 | TTHERM_00717620 | VIC | potassium ion channel |
| 10859 | TTHERM_00717630 | VIC | potassium ion channel |
| 10860 | TTHERM_00717640 | ABC | ? (ABCA) |
| 10861 | TTHERM_00717650 | VIC | potassium ion channel |
| 10862 | TTHERM_00717660 | VIC | potassium ion channel |
| 10863 | TTHERM_00717670 | VIC | potassium ion channel |
| 10887 | TTHERM_00717920 | VIC | potassium ion channel |
| 10972 | TTHERM_00672260 | VIC | potassium ion channel |
| 10973 | TTHERM_00672270 | RND | Niemann-Pick C1 protein homolog |
| 11012 | TTHERM_00675710 | VIC | T-type calcium ion channel |
| 11039 | TTHERM_01284730 | MFS | oxalate/formate? |
| 11040 | TTHERM_01284740 | MFS | oxalate/formate? |
| 11071 | TTHERM_00043940 | PPI | peroxisome assembly protein PEX12 homolog |
| 11186 | TTHERM_00047070 | F-ATPase | protons (vacuolar) |
| 11193 | TTHERM_00047140 | MFS | ? |
| 11251 | TTHERM_00047720 | ABC | ? (ABCA) |
| 11265 | TTHERM_00048850 | MOP | multidrug efflux |
| 11282 | TTHERM_00049030 | P-ATPase | sodium/potassium ion |
| 11367 | TTHERM_00051860 | RIR-CaC | IP3 receptor calcium ion channel |
| 11411 | TTHERM_00052310 | MC | ADP/ATP |
| 11426 | TTHERM_00052460 | F-ATPase | protons (vacuolar) |
| 11527 | TTHERM_00001200 | MFS | hexose |
| 11528 | TTHERM_00001210 | VIC | potassium ion channel |
| 11632 | TTHERM_00006240 | ABC | multispecific organic anion/multidrug (ABCC) |
| 11636 | TTHERM_00006280 | MFS | ? |
| 11637 | TTHERM_00006290 | MFS | ? |
| 11680 | TTHERM_00008700 | VIC | potassium ion channel |
| 11729 | TTHERM_00011230 | VIC | potassium ion channel |
| 11753 | TTHERM_00011470 | ABC | ? (ABCA) |
| 11834 | TTHERM_00013280 | VIC | potassium ion channel |
| 12016 | TTHERM_00024030 | ENT | nucleoside |
| 12021 | TTHERM_00024080 | ABC | multidrug (ABCC) |
| 12143 | TTHERM_00538860 | RIR-CaC | IP3 receptor calcium ion channel |
| 12144 | TTHERM_00538870 | RIR-CaC | IP3 receptor calcium ion channel |
| 12150 | TTHERM_00538930 | VIC | potassium ion channel |
| 12183 | TTHERM_00540250 | MFS | glucose |
| 12184 | TTHERM_00540260 | MFS | glucose |
| 12189 | TTHERM_00540310 | LCT | Mannose-P-dolichol utilization defect 1 protein homolog |
| 12256 | TTHERM_00854310 | VIC | potassium ion channel |
| 12260 | TTHERM_00854350 | VIC | potassium ion channel |
| 12268 | TTHERM_00854430 | P-ATPase | sodium/potassium ion |
| 12269 | TTHERM_00855430 | P-ATPase | sodium/potassium ion |
| 12270 | TTHERM_00856430 | P-ATPase | sodium/potassium ion |
| 12271 | TTHERM_00856440 | P-ATPase | sodium/potassium ion |
| 12345 | TTHERM_00522300 | ABC | lipoprotein release? |
| 12352 | TTHERM_00522370 | RIR-CaC | IP3 receptor calcium ion channel |
| 12358 | TTHERM_00522430 | P-ATPase | calcium ion |
| 12359 | TTHERM_00522440 | ABC | multidrug (ABCC) |
| 12361 | TTHERM_00522460 | P-ATPase | calcium ion |
| 12472 | TTHERM_00225620 | MFS | acetyl-CoA |
| 12487 | TTHERM_00225770 | MFS | organic anion |
| 12496 | TTHERM_00225860 | Annexin | Annexin VII homolog |
| 12497 | TTHERM_00225870 | Annexin | Annexin VII homolog |
| 12535 | TTHERM_00227250 | VIC | potassium ion channel |
| 12671 | TTHERM_00399270 | AAAP | amino acid |
| 12685 | TTHERM_00399410 | VIC | potassium ion channel |
| 12687 | TTHERM_00399430 | Hsp70 | heat shock protein homolog |
| 12730 | TTHERM_00401850 | DMT | drug/metabolite? |
| 12801 | TTHERM_00405540 | MPT | inner membrane translocase TIM22 |
| 12804 | TTHERM_00405570 | AAAP | amino acid |
| 12806 | TTHERM_00405590 | AAAP | amino acid |
| 12849 |  | F-ATPase | protons (vacuolar) |
| 12889 | TTHERM_00654020 | ABC | White protein homolog (ABCG) |
| 13040 | TTHERM_00058550 | Hsp70 | heat shock protein 70 homolog |
| 13053 | TTHERM_00058680 | VIC | potassium ion channel |
| 13121 | TTHERM_00059370 | H+PPase | proton |
| 13126 | TTHERM_00059420 | MFS | organic cation/carnitine |
| 13143 | TTHERM_00061570 | P-ATPase | sodium/potassium ion |
| 13269 | TTHERM_00070790 | VIC | potassium ion channel |
| 13270 | TTHERM_00070800 | VIC | potassium ion channel |
| 13273 | TTHERM_00070830 | VIC | potassium ion channel |
| 13280 | TTHERM_00070900 | ABC | multidrug (P-glycoprotein homolog) (ABCB) |
| 13319 | TTHERM_01171640 | MFS | acetyl-CoA |
| 13437 | TTHERM_00363210 | MC | ADP/ATP |
| 13439 | TTHERM_00363230 | VIC | potassium ion channel |
| 13457 | TTHERM_00365390 | P-ATPase | sodium/potassium ion |
| 13463 | TTHERM_00365450 | P-ATPase | sodium/potassium ion |
| 13506 | TTHERM_00414290 | ABC | multidrug (ABCC) |
| 13527 | TTHERM_00414540 | ABC | multidrug (P-glycoprotein homolog) (ABCB) |
| 13530 | TTHERM_00415580 | ABC | multidrug (P-glycoprotein homolog) (ABCB) |
| 13537 | TTHERM_00415660 | MFS | ? |
| 13609 | TTHERM_00418430 | ABC | White protein homolog (ABCG) |
| 13617 | TTHERM_00418540 | AAAP | amino acid |
| 13636 | TTHERM_00197680 | VIC | potassium ion channel |
| 13738 | TTHERM_00201670 | P-ATPase | cation |
| 13743 | TTHERM_00201720 | P-ATPase | cation |
| 13789 | TTHERM_00204160 | VIC | potassium ion channel |
| 13817 | TTHERM_00209390 | ABC | ? (ABCA) |
| 13822 | TTHERM_00209440 | VIC | potassium ion channel |
| 13863 | TTHERM_00214800 | MOP | multidrug efflux |
| 13920 | TTHERM_00770780 | MPT | outer membrane translocase Tom70 |
| 13931 | TTHERM_00770910 | VIC | potassium ion channel |
| 13932 | TTHERM_00770930 | VIC | potassium ion channel |
| 13937 | TTHERM_00771980 | PPI | peroxisomal targeting signal 1 receptor |
| 13945 | TTHERM_00772070 | VIC | potassium ion channel |
| 13971 | TTHERM_00423380 | ABC | multidrug (P-glycoprotein homolog) (ABCB) |
| 13972 | TTHERM_00423390 | ABC | multidrug (P-glycoprotein homolog) (ABCB) |
| 13996 | TTHERM_00424620 | VIC | potassium ion channel |
| 13997 | TTHERM_00424630 | VIC | potassium ion channel |
| 13998 | TTHERM_00424640 | VIC | potassium ion channel |
| 14021 | TTHERM_00425860 | MFS | hexose |
| 14086 | TTHERM_00427500 | VIC | potassium ion channel |
| 14087 | TTHERM_00427510 | VIC | potassium ion channel |
| 14088 | TTHERM_00427520 | VIC | potassium ion channel |
| 14089 | TTHERM_00427530 | VIC | potassium ion channel |
| 14090 | TTHERM_00427540 | VIC | potassium ion channel |
| 14191 | TTHERM_00028610 | VIC | sodium/calcium ion channel |
| 14193 | TTHERM_00028630 | VIC | sodium ion channel |
| 14202 | TTHERM_00028720 | VIC | potassium ion channel |
| 14292 | TTHERM_00031600 | VIC | potassium ion channel |
| 14293 | TTHERM_00031610 | VIC | potassium ion channel |
| 14294 | TTHERM_00031620 | VIC | potassium ion channel |
| 14309 | TTHERM_00031770 | ABC | White protein homolog (ABCG) |
| 14311 | TTHERM_00031790 | ABC | White protein homolog (ABCG) |
| 14312 | TTHERM_00031800 | ABC | White protein homolog (ABCG) |
| 14313 | TTHERM_00031810 | ABC | White protein homolog (ABCG) |
| 14314 | TTHERM_00031820 | ABC | White protein homolog (ABCG) |
| 14315 | TTHERM_00031830 | ABC | White protein homolog (ABCG) |
| 14320 | TTHERM_00032870 | ABC | White protein homolog (ABCG) |
| 14321 | TTHERM_00032880 | ABC | White protein homolog (ABCG) |
| 14322 | TTHERM_00032890 | ABC | White protein homolog (ABCG) |
| 14324 | TTHERM_00033900 | ABC | White protein homolog (ABCG) |
| 14325 | TTHERM_00033910 | ABC | White protein homolog (ABCG) |
| 14326 | TTHERM_00034910 | ABC | White protein homolog (ABCG) |
| 14327 | TTHERM_00034920 | ABC | White protein homolog (ABCG) |
| 14329 | TTHERM_00034940 | ABC | White protein homolog (ABCG) |
| 14361 | TTHERM_00035270 | ABC | White protein homolog (ABCG) |
| 14362 | TTHERM_00035280 | ABC | White protein homolog (ABCG) |
| 14364 | TTHERM_00035300 | ABC | White protein homolog (ABCG) |
| 14366 | TTHERM_00035320 | ABC | White protein homolog (ABCG) |
| 14367 | TTHERM_00035330 | ABC | White protein homolog (ABCG) |
| 14369 | TTHERM_00035350 | ABC | White protein homolog (ABCG) |
| 14370 | TTHERM_00035360 | ABC | White protein homolog (ABCG) |
| 14372 | TTHERM_00035380 | ABC | White protein homolog (ABCG) |
| 14373 | TTHERM_00035390 | ABC | ? (ABCG) |
| 14374 | TTHERM_00035400 | ABC | ? (ABCG) |
| 14376 | TTHERM_00035420 | ABC | White protein homolog (ABCG) |
| 14377 | TTHERM_00035430 | ABC | White protein homolog (ABCG) |
| 14378 | TTHERM_00035440 | ABC | White protein homolog (ABCG) |
| 14391 | TTHERM_00035570 | IISP | protein translocase (Sec61 alpha homolog) |
| 14392 | TTHERM_00035580 | IISP | protein translocase (Sec61 alpha homolog) |
| 14396 | TTHERM_00035620 | MC | ? |
| 14466 | TTHERM_00037310 | VIC | potassium ion channel |
| 14478 | TTHERM_00037430 | ABC | multidrug (P-glycoprotein homolog) (ABCB) |
| 14497 | TTHERM_00037630 | MC | ADP/ATP |
| 14498 | TTHERM_00037640 | MC | ADP/ATP |
| 14529 | TTHERM_00038940 | MFS | feline leukemia virus subgroup C receptor FLVCR homolog |
| 14585 | TTHERM_00041480 | VIC | small-conductance calcium-activated potassium ion channel |
| 14637 | TTHERM_00885700 | RIR-CaC | IP3 receptor calcium ion channel |
| 14648 | TTHERM_00885840 | VIC | potassium ion channel |
| 14660 | TTHERM_00886950 | VIC | potassium ion channel |
| 14732 | TTHERM_01358410 | ABC | multidrug (ABCC) |
| 14750 | TTHERM_01188330 | AAAP | amino acid |
| 14751 | TTHERM_01188340 | AAAP | amino acid |
| 14837 | TTHERM_01227840 | AAAP | amino acid |
| 14840 | TTHERM_01227870 | ABC | multidrug (ABCC) |
| 14887 | TTHERM_00700900 | P-ATPase | sodium/potassium ion |
| 14890 | TTHERM_00700930 | VIC | potassium ion channel |
| 14895 | TTHERM_00700980 | MOP | multidrug efflux |
| 14898 | TTHERM_00701010 | MOP | multidrug efflux |
| 14996 | TTHERM_00541500 | ABC | mitochondrial ATM1 homolog (ABCB)? |
| 14997 | TTHERM_00541510 | ABC | multidrug (P-glycoprotein homolog) (ABCB) |
| 15033 | TTHERM_00545830 | RIR-CaC | IP3 receptor calcium ion channel |
| 15108 | TTHERM_00549670 | P-ATPase | phospholipid (fused with guanylyl cyclase) |
| 15118 | TTHERM_01012100 | MFS | organic cation |
| 15276 | TTHERM_00927230 | DMT | drug/metabolite? |
| 15281 | TTHERM_00927280 | VIC | large conductance calcium activated potassium ion channel |
| 15467 | TTHERM_00449540 | P-ATPase | phospholipid (fused with guanylyl cyclase) |
| 15523 | TTHERM_00454060 | RIR-CaC | IP3 receptor calcium ion channel |
| 15586 | TTHERM_00294760 | P-ATPase | phospholipid |
| 15632 | TTHERM_00295220 | RIR-CaC | IP3 receptor calcium ion channel |
| 15634 | TTHERM_00295240 | AAAP | amino acid |
| 15652 | TTHERM_00295420 | DMT | drug/metabolite? |
| 15664 | TTHERM_00295540 | VIC | potassium ion channel |
| 15693 | TTHERM_00295830 | MFS | monosaccharide |
| 15698 | TTHERM_00295880 | MFS | monosaccharide |
| 15699 | TTHERM_00295890 | MFS | hexose |
| 15710 | TTHERM_00296000 | VIC | potassium ion channel |
| 15796 | TTHERM_00646900 | MC | ? |
| 15844 | TTHERM_00647380 | ZIP | zinc ion |
| 15849 | TTHERM_00647430 | VIC | potassium/sodium hyperpolarization-activated cyclic nucleotide-gated ion channel |
| 15921 | TTHERM_00320020 | ABC | multispecific organic anion/multidrug (ABCC) |
| 15922 | TTHERM_00320030 | ABC | multispecific organic anion/multidrug (ABCC) |
| 15972 | TTHERM_00321540 | VIC | potassium ion channel |
| 16005 | TTHERM_00322860 | P-ATPase | cation |
| 16025 | TTHERM_00323060 | ABC | ? (ABCA) |
| 16027 | TTHERM_00323080 | ABC | ? (ABCA) |
| 16097 | TTHERM_00680610 | VIC | potassium ion channel |
| 16099 | TTHERM_00680630 | VIC | potassium ion channel |
| 16100 | TTHERM_00680640 | VIC | potassium ion channel |
| 16101 | TTHERM_00680650 | Nramp | metal ion |
| 16102 | TTHERM_00680660 | VIC | potassium ion channel |
| 16127 | TTHERM_00681910 | VIC | cGMP-gated potassium ion channel |
| 16133 | TTHERM_00682960 | VIC | cGMP-gated potassium ion channel |
| 16154 | TTHERM_00683170 | VIC | potassium ion channel |
| 16172 | TTHERM_00683350 | ABC | multidrug (ABCC) |
| 16268 | TTHERM_00082220 | MPT | inner membrane translocase (Tim8) |
| 16304 | TTHERM_00083570 | VIC | potassium ion channel |
| 16305 | TTHERM_00083580 | VIC | potassium ion channel |
| 16306 | TTHERM_00083590 | VIC | potassium ion channel |
| 16307 | TTHERM_00083600 | VIC | potassium ion channel |
| 16308 | TTHERM_00083610 | VIC | potassium ion channel |
| 16326 | TTHERM_00083790 | ABC | multidrug (P-glycoprotein homolog) (ABCB) |
| 16601 | TTHERM_01349990 | VIC | potassium ion channel |
| 16737 | TTHERM_02440130 | P-ATPase | sodium/potassium ion |
| 16838 | TTHERM_00711780 | MC | ADP/ATP |
| 16889 | TTHERM_00713380 | ABC | White protein homolog (ABCG) |
| 16898 | TTHERM_00713470 | MC | 2-oxoglutarate/malate |
| 16922 | TTHERM_01093490 | RIR-CaC | IP3 receptor calcium ion channel |
| 16933 | TTHERM_01093600 | MFS | organic cation |
| 17183 | TTHERM_01002630 | ABC | ? (ABCG) |
| 17184 | TTHERM_01002640 | ABC | ? (ABCG) |
| 17210 | TTHERM_01002900 | VIC | potassium ion channel |
| 17434 | TTHERM_01079190 | VIC | cGMP-gated potassium ion channel |
| 17444 | TTHERM_01079290 | RIR-CaC | IP3 receptor calcium ion channel |
| 17488 | TTHERM_00696910 | P-ATPase | cation |
| 17500 | TTHERM_00697030 | Nramp | metal ion |
| 17519 | TTHERM_00697230 | VIC | potassium ion channel |
| 17672 | TTHERM_01048150 | VIC | potassium ion channel |
| 17689 | TTHERM_01049310 | MFS | hexose |
| 17787 | TTHERM_00836750 | ABC | long-chain fatty acid (ALDP homolog) (ABCD) |
| 17804 | TTHERM_00837910 | VIC | potassium ion channel |
| 17821 | TTHERM_00840060 | P-ATPase | sodium/potassium ion |
| 17831 | TTHERM_00840160 | P-ATPase | phospholipid |
| 17844 | TTHERM_01277490 | VIC | potassium ion channel |
| 17846 | TTHERM_01277510 | VIC | potassium ion channel |
| 17847 | TTHERM_01277520 | VIC | potassium ion channel |
| 17848 | TTHERM_01277530 | VIC | potassium ion channel |
| 17875 | TTHERM_01083010 | ABC | bile pigment (ABCC) |
| 17890 | TTHERM_00931920 | VIC | small conductance calcium-activated potassium ion channel |
| 17917 | TTHERM_00933180 | CPA1 | sodium ion/proton |
| 17957 | TTHERM_01109870 | VIC | potassium ion channel |
| 17958 | TTHERM_01109880 | VIC | potassium ion channel |
| 17961 | TTHERM_01109910 | VIC | potassium ion channel |
| 17962 | TTHERM_01109920 | VIC | potassium ion channel |
| 18001 | TTHERM_01027720 | VIC | potassium ion channel/adenylyl cyclase fusion protein |
| 18052 | TTHERM_00722920 | P-ATPase | calcium ion |
| 18053 | TTHERM_00722930 | CytB | NADPH oxidase homolog |
| 18082 | TTHERM_00723220 | VIC | calcium-activated potassium ion channel |
| 18092 | TTHERM_00723320 | MFS | nitrate? |
| 18093 | TTHERM_00723330 | MFS | nitrate? |
| 18156 | TTHERM_00684430 | ABC | mitochondrial ATM1 homolog (ABCB) |
| 18180 | TTHERM_00684670 | MFS | multidrug efflux |
| 18181 | TTHERM_00684680 | MOP | multidrug efflux |
| 18187 | TTHERM_00684740 | P-ATPase | phospholipid |
| 18192 | TTHERM_00684790 | F-ATPase | protons (mitochondrial) |
| 18204 | TTHERM_00685900 | ABC | multidrug (P-glycoprotein homolog) (ABCB) |
| 18217 | TTHERM_00686030 | VIC | potassium ion channel |
| 18231 | TTHERM_00686170 | MC | aspartate/glutamate (Ca2+activated) |
| 18235 | TTHERM_00686210 | VIC | potassium ion channel |
| 18361 | TTHERM_00993000 | VIC | potassium ion channel |
| 18377 | TTHERM_01443820 | MFS | ? |
| 18435 | TTHERM_00721270 | P-ATPase | cation |
| 18436 | TTHERM_00721280 | VIC | potassium ion channel |
| 18437 | TTHERM_00721290 | VIC | potassium ion channel |
| 18446 | TTHERM_00721380 | VIC | potassium ion channel |
| 18447 | TTHERM_00721390 | VIC | potassium ion channel |
| 18472 | TTHERM_00721640 | MC | 2-oxoglutarate/malate |
| 18480 | TTHERM_00721720 | VIC | potassium ion channel |
| 18520 | TTHERM_00760350 | LCT | Cystinosin homolog |
| 18526 | TTHERM_00760410 | GUP | glycerol |
| 18527 | TTHERM_00760420 | VIC | potassium ion channel |
| 18528 | TTHERM_00760430 | MC | ADP/ATP |
| 18529 | TTHERM_00760440 | VIC | potassium ion channel |
| 18570 | TTHERM_00760850 | VIC | potassium ion channel |
| 18637 | TTHERM_00467350 | MFS | multidrug efflux? |
| 18703 | TTHERM_00468020 | AAAP | amino acid |
| 18721 | TTHERM_00469190 | MC | folate |
| 18736 | TTHERM_00469340 | VIC | potassium ion channel |
| 18737 | TTHERM_00469350 | P-ATPase | cation |
| 18749 | TTHERM_01326850 | VIC | potassium ion channel |
| 18764 | TTHERM_01436750 | MFS | feline leukemia virus subgroup C receptor FLVCR homolog |
| 18851 | TTHERM_00239130 | DMT | UDP-galactose |
| 18884 | TTHERM_00240450 | ABC | multidrug (P-glycoprotein homolog) (ABCB) |
| 18888 | TTHERM_00241480 | ABC | multidrug (P-glycoprotein homolog) (ABCB) |
| 18900 | TTHERM_00241620 | VIC | potassium ion channel |
| 18917 | TTHERM_00241790 | P-ATPase | calcium ion |
| 18968 | TTHERM_00242310 | MFS | ? |
| 19038 | TTHERM_00715640 | P-ATPase | phospholipid |
| 19063 | TTHERM_00715890 | VIC | potassium ion channel |
| 19167 | TTHERM_00616180 | ABC | glutathione S-conjugate (ABCC) |
| 19168 | TTHERM_00616190 | VIC | potassium ion channel |
| 19200 | TTHERM_00616510 | VIC | potassium ion channel |
| 19299 | TTHERM_00300040 | MFS | ? |
| 19302 | TTHERM_00300070 | VIC | potassium ion channel |
| 19318 | TTHERM_00300230 | AAAP | amino acid |
| 19332 | TTHERM_00300370 | ABC | multidrug (ABCC) |
| 19382 | TTHERM_00301860 | ABC | ? (ABCA) |
| 19383 | TTHERM_00301870 | ABC | ? (ABCA) |
| 19391 | TTHERM_00301950 | MFS | sugar? |
| 19399 | TTHERM_00302070 | VIC | potassium ion channel |
| 19475 | TTHERM_01125250 | MFS | hexose |
| 19476 | TTHERM_01125260 | MFS | hexose |
| 19477 | TTHERM_01125270 | MFS | hexose |
| 19478 | TTHERM_01125280 | MFS | hexose |
| 19479 | TTHERM_01125290 | MFS | hexose |
| 19537 | TTHERM_00486050 | MFS | glycerol-3-phosphate |
| 19584 | TTHERM_00486530 | MFS | ? |
| 19589 | TTHERM_00486580 | DMT | drug/metabolite? |
| 19620 | TTHERM_00486890 | VIC | potassium ion channel/adenylyl cyclase fusion protein |
| 19644 | TTHERM_00487130 | VIC | potassium ion channel/adenylyl cyclase fusion protein |
| 19723 | TTHERM_00637200 | GPH | sugar |
| 19774 | TTHERM_00637710 | MC | ADP/ATP |
| 19843 | TTHERM_00531830 | VIC | sodium ion channel |
| 19871 | TTHERM_00532130 | MOP | multidrug efflux |
| 19882 | TTHERM_00532240 | MOP | multidrug efflux |
| 19893 | TTHERM_00532350 | VIC | potassium ion channel |
| 19899 | TTHERM_00532410 | VIC | potassium ion channel |
| 19903 | TTHERM_00532470 | RIR-CaC | IP3 receptor calcium ion channel |
| 19930 | TTHERM_00532740 | AAAP | amino acid |
| 19935 | TTHERM_00532790 | ABC | ? (ABCA) |
| 19938 | TTHERM_00532820 | MFS | glucose |
| 19941 | TTHERM_00532850 | MFS | glucose |
| 20031 | TTHERM_00994350 | P-ATPase | phospholipid |
| 20186 | TTHERM_01151440 | ABC | multidrug/bile salt (ABCB) |
| 20187 | TTHERM_01151450 | ABC | multidrug (P-glycoprotein homolog) (ABCB) |
| 20321 | TTHERM_00456910 | MFS | ? |
| 20372 | TTHERM_00459410 | MFS | hexose |
| 20427 | TTHERM_00823540 | ABC | multidrug (bacterial homolog) |
| 20464 | TTHERM_00823960 | MFS | glucose |
| 20500 | TTHERM_01250070 | VIC | potassium ion channel |
| 20502 | TTHERM_01250090 | VIC | potassium ion channel |
| 20535 | TTHERM_00898300 | ABC | ? (ABCA) |
| 20605 | TTHERM_00147470 | ENT | nucleoside |
| 20676 | TTHERM_00149170 | VIC | sodium ion channel |
| 20708 | TTHERM_00149490 | VIC | potassium ion channel |
| 20788 | TTHERM_00151270 | MC | uncoupling protein homolog |
| 20824 | TTHERM_00151660 | VIC | potassium ion channel |
| 20831 | TTHERM_00151730 | VIC | potassium ion channel |
| 20834 | TTHERM_00151760 | VIC | potassium ion channel |
| 20901 | TTHERM_00314910 | MOP | multidrug efflux |
| 20938 | TTHERM_00316270 | MFS | feline leukemia virus subgroup C receptor FLVCR homolog |
| 20942 | TTHERM_00316310 | CPA1 | sodium ion/proton |
| 20968 | TTHERM_00316570 | VIC | potassium ion channel |
| 20974 | TTHERM_00316630 | P-ATPase | phospholipid |
| 20994 | TTHERM_00316940 | MOP | multidrug efflux |
| 21055 | TTHERM_00318540 | MFS | glucose |
| 21091 | TTHERM_00318900 | MFS | sugar? |
| 21164 | TTHERM_00922850 | VIC | potassium ion channel |
| 21194 | TTHERM_00923150 | P-ATPase | cation |
| 21195 | TTHERM_00923160 | P-ATPase | cation |
| 21196 | TTHERM_00923170 | P-ATPase | cation |
| 21242 | TTHERM_00304370 | MFS | glycerol-3-phosphate? |
| 21249 | TTHERM_00305430 | VIC | potassium ion channel |
| 21281 | TTHERM_00307780 | VIC | potassium ion channel |
| 21282 | TTHERM_00307790 | VIC | potassium ion channel |
| 21288 | TTHERM_00309830 | DMT | drug/metabolite? |
| 21295 | TTHERM_00309900 | VIC | potassium ion channel |
| 21347 | TTHERM_00310430 | VIC | cGMP-gated cation channel |
| 21348 | TTHERM_00310440 | VIC | potassium ion channel |
| 21354 | TTHERM_00310500 | VIC | potassium ion channel |
| 21363 | TTHERM_00310590 | VIC | potassium ion channel |
| 21377 | TTHERM_00310760 | DMT | drug/metabolite? |
| 21383 | TTHERM_00310820 | MC | uncoupling protein homolog |
| 21400 | TTHERM_00794010 | MOP | multidrug efflux |
| 21402 | TTHERM_00794030 | ABC | ? (ABCG) |
| 21485 | TTHERM_00407700 | VIC | potassium ion channel |
| 21525 | TTHERM_00409090 | ABC | White protein homolog (ABCG) |
| 21581 | TTHERM_00411630 | P-ATPase | phospholipid |
| 21623 | TTHERM_00412050 | MFS | hexose |
| 21691 | TTHERM_01295350 | VIC | potassium ion channel |
| 21692 | TTHERM_01295360 | VIC | potassium ion channel |
| 21697 | TTHERM_01359440 | P-ATPase | phospholipid |
| 21721 | TTHERM_01433590 | VIC | potassium ion channel |
| 21753 | TTHERM_00977660 | VIC | potassium ion channel |
| 21756 | TTHERM_00977690 | VIC | potassium ion channel |
| 21757 | TTHERM_00977700 | VIC | potassium ion channel |
| 21758 | TTHERM_00977710 | VIC | potassium ion channel |
| 21759 | TTHERM_00977720 | P-ATPase | phospholipid |
| 21833 | TTHERM_00724680 | ZIP | zinc ion |
| 21861 | TTHERM_00725950 | MFS | ? |
| 21881 | TTHERM_00726150 | VIC | potassium ion channel |
| 21886 | TTHERM_00726210 | VIC | potassium ion channel |
| 21889 | TTHERM_00726240 | VIC | potassium ion channel |
| 21910 | TTHERM_00726450 | MC | ? |
| 21944 | TTHERM_00895620 | Hsp70 | heat shock protein 70 homolog |
| 22070 | TTHERM_00600070 | ABC | White protein homolog (ABCG) |
| 22075 | TTHERM_00600120 | P-ATPase | cation |
| 22089 | TTHERM_00600260 | VIC | potassium ion channel |
| 22094 | TTHERM_00600310 | P-ATPase | phospholipid |
| 22115 | TTHERM_00600520 | MFS | ? |
| 22206 | TTHERM_00907060 | ENT | nucleoside |
| 22207 | TTHERM_00907070 | ENT | nucleoside |
| 22260 | TTHERM_00218330 | F-ATPase | protons (vacuolar) |
| 22270 | TTHERM_00218430 | ABC | ? (ABCA) |
| 22272 | TTHERM_00218450 | VIC | potassium ion channel |
| 22305 | TTHERM_00218780 | VIC | potassium ion channel |
| 22306 | TTHERM_00218790 | H+PPase | proton |
| 22316 | TTHERM_00218890 | NSS | sodium ion/GABA neurotransmitter |
| 22341 | TTHERM_00219130 | MFS | multidrug efflux? |
| 22354 | TTHERM_00219260 | CaCA | sodium ion:calcium/potassium ion |
| 22381 | TTHERM_00219530 | ABC | multispecific organic anion/multidrug (ABCC) |
| 22402 | TTHERM_00220740 | MC | ADP/ATP |
| 22438 | TTHERM_00221100 | MC | uncoupling protein homolog |
| 22443 | TTHERM_00221150 | ENT | nucleoside |
| 22469 | TTHERM_00470550 | P-ATPase | phospholipid |
| 22470 | TTHERM_00470560 | VIC | potassium ion channel |
| 22490 | TTHERM_00470760 | VIC | potassium ion channel |
| 22513 | TTHERM_00470990 | Hsp70 | heat shock protein homolog |
| 22556 | TTHERM_00471460 | VIC | potassium ion channel |
| 22558 | TTHERM_00471480 | VIC | potassium ion channel |
| 22565 | TTHERM_00471550 | VIC | potassium ion channel |
| 22604 | TTHERM_00471960 | ArsAB | arsenite (ArsA) |
| 22707 | TTHERM_00802360 | AAAP | amino acid |
| 22709 | TTHERM_00802390 | AAAP | amino acid |
| 22715 | TTHERM_00802450 | MC | tricarboxylate |
| 22728 | TTHERM_00803570 | ABC | multidrug (P-glycoprotein homolog) (ABCB) |
| 22729 | TTHERM_00803580 | VIC | potassium ion channel |
| 22988 | TTHERM_00103870 | VIC | potassium ion channel |
| 23013 | TTHERM_00105110 | Hsp70 | heat shock protein 70 homolog |
| 23032 | TTHERM_00105320 | F-ATPase | protons (vacuolar) |
| 23074 | TTHERM_00106800 | F-ATPase | protons (vacuolar) |
| 23125 | TTHERM_00688300 | Hsp70 | heat shock protein homolog |
| 23145 | TTHERM_00688500 | P-ATPase | calcium ion |
| 23157 | TTHERM_00688620 | VIC | potassium ion channel |
| 23185 | TTHERM_00689890 | VIC | potassium ion channel |
| 23233 | TTHERM_00797940 | P-ATPase | phospholipid |
| 23235 | TTHERM_00797960 | P-ATPase | phospholipid |
| 23236 | TTHERM_00797970 | P-ATPase | phospholipid |
| 23251 | TTHERM_00798120 | MFS | oxalate/formate? |
| 23254 | TTHERM_00798150 | MFS | oxalate/formate? |
| 23255 | TTHERM_00798160 | MFS | oxalate/formate? |
| 23256 | TTHERM_00798170 | MFS | oxalate/formate? |
| 23257 | TTHERM_00798180 | MFS | oxalate/formate? |
| 23258 | TTHERM_00799180 | MFS | oxalate/formate? |
| 23334 | TTHERM_00891210 | VIC | potassium ion channel |
| 23394 | TTHERM_00807980 | CPA1 | sodium ion/proton |
| 23404 | TTHERM_00808080 | VIC | potassium ion channel |
| 23412 | TTHERM_00809160 | VIC | potassium ion channel |
| 23415 | TTHERM_00809200 | VIC | potassium ion channel |
| 23416 | TTHERM_00809210 | VIC | potassium ion channel |
| 23417 | TTHERM_00809220 | VIC | potassium ion channel |
| 23418 | TTHERM_00809230 | VIC | potassium ion channel |
| 23430 | TTHERM_00809350 | VIC | potassium ion channel |
| 23432 | TTHERM_00809370 | VIC | potassium ion channel |
| 23507 | TTHERM_00392790 | H+PPase | proton |
| 23510 | TTHERM_00392820 | GIC | glutamate gated ion channel |
| 23550 | TTHERM_00393270 | MFS | organic cation/carnitine |
| 23564 | TTHERM_00393410 | VIC | potassium ion channel |
| 23573 | TTHERM_00394490 | MC | folate |
| 23627 | TTHERM_00397020 | VIC | potassium ion channel |
| 23630 | TTHERM_00397050 | VIC | potassium ion channel |
| 23731 | TTHERM_00912240 | ABC | multispecific organic anion/multidrug (ABCC) |
| 23732 | TTHERM_00912250 | ABC | multispecific organic anion/multidrug (ABCC) |
| 23734 | TTHERM_00912270 | ABC | multispecific organic anion/multidrug (ABCC) |
| 23741 | TTHERM_00913370 | VIC | potassium ion channel |
| 23742 | TTHERM_00913380 | P-ATPase | cation |
| 23743 | TTHERM_00913390 | P-ATPase | cation |
| 23750 | TTHERM_00916430 | ABC | multidrug (ABCC) |
| 23752 | TTHERM_00917440 | ABC | multispecific organic anion/multidrug (ABCC) |
| 23753 | TTHERM_00917450 | ABC | multispecific organic anion/multidrug (ABCC) |
| 23795 | TTHERM_00661500 | MC | ADP/ATP |
| 23803 | TTHERM_00661580 | ABC | multidrug (ABCC) |
| 23804 | TTHERM_00661590 | ABC | multidrug (ABCC) |
| 23829 | TTHERM_00663820 | MFS | oxalate/formate? |
| 23839 | TTHERM_00663920 | VIC | potassium ion channel |
| 23845 | TTHERM_00663980 | MTC | tricarboxylates |
| 23883 | TTHERM_00666300 | NSCC2 | translocation protein SEC62 homolog |
| 23916 | TTHERM_00666630 | P-ATPase | cation |
| 24151 | TTHERM_00590370 | CaCA | sodium ion/calcium ion |
| 24169 | TTHERM_00591540 | MC | ? |
| 24178 | TTHERM_00591630 | VIC | potassium ion channel |
| 24196 | TTHERM_01006390 | VIC | potassium ion channel |
| 24247 | TTHERM_00131260 | MFS | glucose |
| 24248 | TTHERM_00131270 | MFS | sugar |
| 24250 | TTHERM_00131290 | MFS | hexose |
| 24252 | TTHERM_00131310 | MFS | sugar |
| 24325 | TTHERM_00136000 | ABC | multidrug (P-glycoprotein homolog) (ABCB) |
| 24339 | TTHERM_00136140 | MFS | oxalate/formate? |
| 24343 | TTHERM_00136180 | MFS | oxalate/formate? |
| 24395 | TTHERM_00137690 | VIC | calcium-activated potassium ion channel |
| 24411 | TTHERM_00137850 | ABC | multidrug (ABCC) |
| 24453 | TTHERM_00138260 | ABC | ? (ABCA) |
| 24457 | TTHERM_00138300 | VIC | potassium ion channel |
| 24469 | TTHERM_00138420 | MFS | organic cation |
| 24483 | TTHERM_00138560 | RIR-CaC | IP3 receptor calcium ion channel |
| 24496 | TTHERM_00139690 | MFS | ? |
| 24498 | TTHERM_00139710 | MFS | ? |
| 24582 | TTHERM_01121620 | VIC | potassium ion channel |
| 24583 | TTHERM_01121630 | VIC | potassium ion channel |
| 24641 | TTHERM_00627160 | VIC | pot/sodium hyperpolarization-activated cyclic nucleotide-gated ion channel |
| 24642 | TTHERM_00627170 | MC | carnitine/acylcarnitine |
| 24643 | TTHERM_00627180 | MC | carnitine/acylcarnitine |
| 24644 | TTHERM_00627190 | MC | carnitine/acylcarnitine |
| 24683 | TTHERM_00628570 | VIC | potassium ion channel |
| 24684 |  | RIR-CaC | IP3 receptor calcium ion channel |
| 24686 | TTHERM_00628600 | F-ATPase | protons (vacuolar) |
| 24704 | TTHERM_01184150 | VIC | potassium ion channel |
| 24712 | TTHERM_01184230 | VIC | potassium ion channel |
| 24802 | TTHERM_00387080 | P-ATPase | calcium ion |
| 24838 | TTHERM_00388430 | CPA1 | sodium ion/proton |
| 24904 | TTHERM_00390080 | FBT | folate/pteridine? |
| 24917 | TTHERM_00390210 | MFS | feline leukemia virus subgroup C receptor FLVCR homolog |
| 24975 | TTHERM_00997500 | RIR-CaC | IP3 receptor calcium ion channel |
| 24976 | TTHERM_00997510 | VIC | potassium ion channel |
| 24978 | TTHERM_00997530 | VIC | potassium ion channel |
| 24979 | TTHERM_00997540 | VIC | potassium ion channel |
| 24980 | TTHERM_00997550 | VIC | potassium ion channel |
| 24983 | TTHERM_00997580 | VIC | potassium ion channel |
| 24984 | TTHERM_00997590 | VIC | potassium ion channel |
| 24985 | TTHERM_00997600 | VIC | potassium ion channel |
| 25052 | TTHERM_00592990 | ABC | multidrug (ABCC) |
| 25054 | TTHERM_00593010 | ABC | multispecific organic anion/multidrug (ABCC) |
| 25055 | TTHERM_00593020 | ABC | multispecific organic anion/multidrug (ABCC) |
| 25063 | TTHERM_00594090 | ABC | multidrug (ABCC) |
| 25155 | TTHERM_00941490 | AAAP | amino acid |
| 25187 | TTHERM_01821940 | P-ATPase | sodium/potassium ion |
| 25251 | TTHERM_00249700 | P-ATPase | cation |
| 25253 | TTHERM_00249720 | P-ATPase | cation |
| 25257 | TTHERM_00249760 | P-ATPase | cation |
| 25260 | TTHERM_00249790 | P-ATPase | cation |
| 25262 | TTHERM_00249810 | P-ATPase | cation |
| 25299 | TTHERM_00251180 | ZIP | zinc ion |
| 25300 | TTHERM_00251190 | ZIP | zinc ion |
| 25305 | TTHERM_00251240 | ZIP | zinc ion |
| 25318 | TTHERM_00252360 | VIC | potassium ion channel |
| 25320 | TTHERM_00252380 | VIC | potassium ion channel |
| 25321 | TTHERM_00252390 | VIC | potassium ion channel |
| 25323 | TTHERM_00252410 | VIC | potassium ion channel |
| 25431 | TTHERM_00734080 | ENT | nucleoside |
| 25458 | TTHERM_00735340 | H+PPase | proton |
| 25538 | TTHERM_00784340 | VIC | potassium ion channel |
| 25569 | TTHERM_00784650 | CDF | cation efflux |
| 25589 | TTHERM_00581460 | VIC | potassium ion channel |
| 25590 | TTHERM_00581470 | VIC | potassium ion channel |
| 25599 | TTHERM_00581590 | VIC | potassium ion channel |
| 25794 | TTHERM_00463420 | F-ATPase | protons (vacuolar) |
| 25802 | TTHERM_00463500 | ABC | TAP-family protein (ABCB) |
| 25823 | TTHERM_00463710 | ENT | nucleoside |
| 25831 | TTHERM_00463790 | P-ATPase | calcium ion |
| 25833 | TTHERM_00463810 | MC | ADP/ATP |
| 25886 | TTHERM_00285570 | MFS | organic anion |
| 25925 | TTHERM_00287940 | VIC | potassium ion channel |
| 26059 | TTHERM_00998800 | ABC | multispecific organic anion/multidrug (ABCC) |
| 26061 | TTHERM_00998820 | MC | ? |
| 26083 | TTHERM_00999040 | CPA1 | sodium ion/proton |
| 26130 | TTHERM_00343500 | IISP | Signal recognition particle (SRP54) |
| 26142 | TTHERM_00343620 | Annexin | Annexin VII homolog |
| 26143 | TTHERM_00343630 | Annexin | Annexin VII homolog |
| 26177 | TTHERM_00343980 | VIC | potassium ion channel |
| 26178 | TTHERM_00343990 | VIC | potassium ion channel |
| 26187 | TTHERM_00344080 | ABC | glutathione-conjugate (ABCC) |
| 26201 | TTHERM_00344220 | ABC | multispecific organic anion/multidrug (ABCC) |
| 26221 | TTHERM_00346400 | ABC | multispecific organic anion/multidrug (ABCC) |
| 26252 | TTHERM_00346710 | ABC | multispecific organic anion/multidrug (ABCC) |
| 26263 | TTHERM_00346880 | VIC | potassium ion channel |
| 26282 | TTHERM_00752140 | MFS | multidrug efflux? |
| 26410 | TTHERM_00429690 | VIC | potassium ion channel |
| 26413 | TTHERM_00429720 | P-ATPase | phospholipid (fused with guanylyl cyclase) |
| 26490 | TTHERM_00433470 | F-ATPase | protons (vacuolar) |
| 26583 | TTHERM_00140780 | MFS | ? |
| 26630 | TTHERM_00142240 | IISP | Signal recognition particle (SRP54) |
| 26720 | TTHERM_00145120 | VIC | potassium ion channel |
| 26721 | TTHERM_00145130 | VIC | potassium ion channel |
| 26722 | TTHERM_00145140 | VIC | potassium ion channel |
| 26723 | TTHERM_00145150 | VIC | potassium ion channel |
| 26728 | TTHERM_00145200 | MFS | sugar |
| 26748 | TTHERM_00145400 | CaCA | sodium ion/calcium/potassium ion |
| 26761 | TTHERM_00145530 | P-ATPase | cation |
| 26804 | TTHERM_00145970 | RIR-CaC | IP3 receptor calcium ion channel |
| 26842 | TTHERM_00146350 | ABC | ? (ABCA) |
| 26852 | TTHERM_00146450 | MFS | oxalate/formate? |
| 26892 | TTHERM_00706360 | ABC | multidrug (ABCC) |
| 26925 | TTHERM_00709650 | DMT | drug/metabolite? |
| 26997 | TTHERM_00942770 | ABC | multidrug (ABCC) |
| 27014 | TTHERM_00942940 | MC | peroxisome membrane protein homolog |
| 27061 | TTHERM_00112470 | F-ATPase | protons (vacuolar) |
| 27140 | TTHERM_00113260 | MFS | glycerol-3-phosphate |
| 27154 | TTHERM_00114390 | VIC | potassium ion channel |
| 27175 | TTHERM_00117570 | DMT | UDP-N-acetylglucosamine |
| 27180 | TTHERM_00118610 | F-ATPase | protons (mitochondrial) |
| 27252 | TTHERM_00122370 | VIC | calcium ion channel |
| 27255 | TTHERM_00122400 | VIC | calcium ion channel |
| 27256 | TTHERM_00122410 | VIC | calcium ion channel |
| 27258 | TTHERM_00122430 | VIC | calcium ion channel |
| 27271 | TTHERM_00123550 | VIC | potassium ion channel |
| 27273 | TTHERM_00123570 | VIC | potassium ion channel |
| 27286 | TTHERM_00123720 | P-ATPase | phospholipid |
| 27360 | TTHERM_00607040 | VIC | potassium ion channel |
| 27363 | TTHERM_00607070 | VIC | potassium ion channel |
| 27364 | TTHERM_00607080 | VIC | potassium ion channel |
| 27451 | TTHERM_00354640 | RIR-CaC | IP3 receptor calcium ion channel |
| 27458 | TTHERM_00354720 | MFS | organic cation |
| 27492 | TTHERM_00355060 | MFS | sugar |
| 27493 | TTHERM_00355070 | MFS | glucose |
| 27559 | TTHERM_00355730 | VIC | calcium ion channel |
| 27566 | TTHERM_00355800 | P-ATPase | sodium/potassium ion |
| 27619 | TTHERM_01005020 | VIC | potassium ion channel |
| 27624 | TTHERM_01005070 | VIC | potassium ion channel |
| 27638 | TTHERM_01005210 | F-ATPase | protons (vacuolar) |
| 27752 | TTHERM_01018340 | MC | carnitine/acylcarnitine |
| 27782 | TTHERM_01050390 | VIC | potassium ion channel |
| 27911 | TTHERM_00561500 | ENT | nucleoside |
| 27917 | TTHERM_00561570 | ABC | multidrug (ABCC) |
| 27919 | TTHERM_00561590 | ABC | multidrug (ABCC) |
| 27920 | TTHERM_00561600 | ABC | White protein homolog (ABCG) |
| 27930 | TTHERM_00561700 | DMT | UDP-N-acetylglucosamine |
| 28048 | TTHERM_00635770 | ABC | ? (ABCA) |
| 28102 | TTHERM_01332070 | F-ATPase | protons (vacuolar) |
| 28167 | TTHERM_00975370 | VIC | large-conductance calcium-activated potassium ion channel |
| 28333 | TTHERM_00155430 | ENT | nucleoside |
| 28334 | TTHERM_00155440 | ENT | nucleoside |
| 28372 | TTHERM_00157820 | MFS | glucose |
| 28423 | TTHERM_00158330 | VIC | potassium ion channel |
| 28440 | TTHERM_00158500 | VIC | potassium ion channel |
| 28487 | TTHERM_00160950 | ABC | multispecific organic anion/multidrug (ABCC) |
| 28496 | TTHERM_00161040 | MFS | glucose |
| 28514 | TTHERM_00161220 | SulP | sulfate |
| 28517 | TTHERM_00161250 | F-ATPase | protons (vacuolar) |
| 28518 | TTHERM_00161260 | F-ATPase | protons (vacuolar) |
| 28557 | TTHERM_00161670 | VIC | potassium ion channel |
| 28571 | TTHERM_00161810 | VIC | potassium ion channel |
| 28576 | TTHERM_00161860 | ABC | multidrug (ABCC) |
| 28590 | TTHERM_00370740 | RIR-CaC | IP3 receptor calcium ion channel |
| 28660 | TTHERM_00372460 | P-ATPase | phospholipid |
| 28667 | TTHERM_00372530 | F-ATPase | protons (vacuolar) |
| 28676 | TTHERM_00372620 | ABC | multidrug (ABCC) |
| 28678 | TTHERM_00372640 | ABC | multidrug (ABCC) |
| 28710 | TTHERM_00374960 | MC | aspartate/glutamate (Ca2+activated) |
| 28819 | TTHERM_01166280 | P-ATPase | phospholipid |
| 28914 | TTHERM_00574250 | ABC | multidrug (ABCC) |
| 28932 | TTHERM_00575420 | MC | ADP/ATP |
| 28937 | TTHERM_00575470 | P-ATPase | cation |
| 28938 | TTHERM_00575480 | P-ATPase | cation |
| 28941 | TTHERM_00575510 | P-ATPase | cation |
| 29171 | TTHERM_00865410 | F-ATPase | protons (mitochondrial) |
| 29172 | TTHERM_00865420 | F-ATPase | protons (mitochondrial) |
| 29183 | TTHERM_00550750 | VIC | potassium ion channel |
| 29184 | TTHERM_00550760 | VIC | potassium ion channel |
| 29186 | TTHERM_00550780 | VIC | potassium ion channel |
| 29187 | TTHERM_00550790 | VIC | potassium ion channel |
| 29197 | TTHERM_00550890 | ABC | TAP-family protein (ABCB) |
| 29198 | TTHERM_00550900 | ABC | TAP-family protein (ABCB) |
| 29306 | TTHERM_00259400 | MC | dicarboxylate/tricarboxylate |
| 29404 | TTHERM_00263390 | VIC | potassium ion channel |
| 29405 | TTHERM_00263400 | VIC | potassium ion channel |
| 29410 | TTHERM_00263450 | VIC | potassium ion channel |
| 29412 | TTHERM_00263470 | VIC | potassium ion channel |
| 29414 | TTHERM_00263490 | VIC | potassium ion channel |
| 29415 | TTHERM_00263500 | VIC | potassium ion channel |
| 29417 | TTHERM_00263520 | VIC | potassium ion channel |
| 29422 | TTHERM_00263570 | VIC | potassium ion channel |
| 29468 | TTHERM_00265100 | P-ATPase | cation |
| 29472 | TTHERM_00265150 | ABC | multidrug (ABCC) |
| 29642 | TTHERM_00964340 | ABC | multispecific organic anion/multidrug (ABCC) |
| 29698 | TTHERM_00163930 | VIC | potassium ion channel |
| 29699 | TTHERM_00163940 | VIC | potassium ion channel |
| 29701 | TTHERM_00164950 | VIC | potassium ion channel |
| 29702 | TTHERM_00164960 | VIC | potassium ion channel |
| 29703 | TTHERM_00164970 | VIC | potassium ion channel |
| 29704 | TTHERM_00164980 | VIC | potassium ion channel |
| 29705 | TTHERM_00164990 | VIC | potassium ion channel |
| 29706 | TTHERM_00165000 | VIC | potassium ion channel |
| 29794 | TTHERM_00171850 | Hsp70 | heat shock protein 70 homolog |
| 29796 | TTHERM_00171870 | AAAP | amino acid |
| 29816 | TTHERM_00180980 | VIC | potassium ion channel |
| 29842 | TTHERM_00185200 | IISP | Signal recognition particle receptor alpha subunit |
| 29926 | TTHERM_00187090 | VIC | large-conductance calcium-activated potassium ion channel |
| 29927 | TTHERM_00187100 | VIC | large-conductance calcium-activated potassium ion channel |
| 30039 | TTHERM_01128600 | ABC | White protein homolog (ABCG) |
| 30159 | TTHERM_00325570 | P-ATPase | phospholipid |
| 30164 | TTHERM_00325620 | DMT | drug/metabolite? |
| 30165 | TTHERM_00325630 | DMT | drug/metabolite? |
| 30175 | TTHERM_00326720 | VIC | small-conductance calcium-activated potassium ion channel |
| 30180 | TTHERM_00326770 | AAAP | amino acid |
| 30201 | TTHERM_00326980 | ZIP | zinc ion |
| 30202 | TTHERM_00326990 | ZIP | zinc ion |
| 30217 | TTHERM_00327140 | VIC | potassium ion channel |
| 30236 | TTHERM_00327330 | VIC | potassium ion channel |
| 30326 | TTHERM_00332190 | FBT | folate/pteridine |
| 30386 | TTHERM_01444880 | MFS | organic cation? |

C. ID numbers for all *T. thermophila* protease-encoding genes referred to in Table 7.

| **Preliminary TIGR Gene ID** | **GB_ACCESSION** | **Catalytic Class** | **Protease family/ nomenclature** |
| --- | --- | --- | --- |
| 1444 | TTHERM_00971740 | **Aspartic** | A1 (pepsin family) |
| 3901 | TTHERM_00446340 |  |  |
| 5393 | TTHERM_00437370 |  |  |
| 5395 | TTHERM_00437390 |  |  |
| 5506 | TTHERM_00440510 |  |  |
| 7404 | TTHERM_00624160 |  |  |
| 7618 | TTHERM_00678540 |  |  |
| 8710 | TTHERM_00189600 |  |  |
| 8771 | TTHERM_00191200 |  |  |
| 8772 | TTHERM_00191210 |  |  |
| 9396 | TTHERM_00128320 |  |  |
| 11117 | TTHERM_00046380 |  |  |
| 11399 | TTHERM_00052190 |  |  |
| 11777 | TTHERM_00011710 |  |  |
| 12762 | TTHERM_00402180 |  |  |
| 13753 | TTHERM_00202810 |  |  |
| 14470 | TTHERM_00037350 |  |  |
| 15853 | TTHERM_00647470 |  |  |
| 15986 | TTHERM_00321680 |  |  |
| 16269 | TTHERM_00082230 |  |  |
| 17995 | TTHERM_01027660 |  |  |
| 18950 | TTHERM_00242120 |  |  |
| 22027 | TTHERM_01053040 |  |  |
| 22028 | TTHERM_01053050 |  |  |
| 22030 | TTHERM_01053070 |  |  |
| 22135 | TTHERM_00600720 |  |  |
| 23089 | TTHERM_00106950 |  |  |
| 23582 | TTHERM_00394580 |  |  |
| 26502 | TTHERM_00433600 |  |  |
| 27112 | TTHERM_00112980 |  |  |
| 27113 | TTHERM_00112990 |  |  |
| 27377 | TTHERM_00607210 |  |  |
| 27912 | TTHERM_00561510 |  |  |
| 27914 | TTHERM_00561530 |  |  |
| 27915 | TTHERM_00561540 |  |  |
| 27916 | TTHERM_00561550 |  |  |
| 30040 | TTHERM_01128610 |  |  |
| 30041 | TTHERM_01128620 |  |  |
| 30042 | TTHERM_01128630 |  |  |
| 30043 | TTHERM_01128640 |  |  |
| 21904 | TTHERM_00726390 |  | A22 (impas 1 endopeptidase) |
| 24517 | TTHERM_00835020 |  |  |
| 27498 | TTHERM_00355120 |  |  |
| 1206 | TTHERM_00853070 | **Cysteine** | C1 (papain family) |
| 1247 | TTHERM_02592230 |  |  |
| 3001 | TTHERM_00691630 |  |  |
| 3861 | TTHERM_00445920 |  |  |
| 4281 | TTHERM_00494530 |  |  |
| 4392 | TTHERM_00925850 |  |  |
| 4394 | TTHERM_00925870 |  |  |
| 4492 | TTHERM_01248970 |  |  |
| 4729 | TTHERM_00530660 |  |  |
| 5278 | TTHERM_00348660 |  |  |
| 5481 | TTHERM_00439240 |  |  |
| 5482 | TTHERM_00439250 |  |  |
| 5633 | TTHERM_00442330 |  |  |
| 6274 | TTHERM_00483400 |  |  |
| 6275 | TTHERM_00483410 |  |  |
| 6344 | TTHERM_00525070 |  |  |
| 6517 | TTHERM_00629950 |  |  |
| 7899 | TTHERM_00558010 |  |  |
| 8076 | TTHERM_00502370 |  |  |
| 8108 | TTHERM_00502690 |  |  |
| 8778 | TTHERM_00191270 |  |  |
| 8779 | TTHERM_00191280 |  |  |
| 8982 | TTHERM_00564440 |  |  |
| 9463 | TTHERM_00128990 |  |  |
| 9464 | TTHERM_00129000 |  |  |
| 9465 | TTHERM_00129010 |  |  |
| 9706 | TTHERM_00877100 |  |  |
| 9718 | TTHERM_00878210 |  |  |
| 9721 | TTHERM_00879230 |  |  |
| 9723 | TTHERM_00879250 |  |  |
| 9725 | TTHERM_00879270 |  |  |
| 9726 | TTHERM_00879280 |  |  |
| 9727 | TTHERM_00879290 |  |  |
| 9728 | TTHERM_00879300 |  |  |
| 9732 | TTHERM_00881320 |  |  |
| 9733 | TTHERM_00881330 |  |  |
| 9737 | TTHERM_00881370 |  |  |
| 9738 | TTHERM_00881380 |  |  |
| 9739 | TTHERM_00881390 |  |  |
| 9741 | TTHERM_00881410 |  |  |
| 9742 | TTHERM_00881420 |  |  |
| 9744 | TTHERM_00881440 |  |  |
| 9745 | TTHERM_00881450 |  |  |
| 9840 | TTHERM_00268050 |  |  |
| 9841 | TTHERM_00268060 |  |  |
| 10029 | TTHERM_00075840 |  |  |
| 10034 | TTHERM_00075890 |  |  |
| 10184 | TTHERM_00079380 |  |  |
| 10191 | TTHERM_00079450 |  |  |
| 10200 | TTHERM_00079550 |  |  |
| 10203 | TTHERM_00079580 |  |  |
| 10205 | TTHERM_00079600 |  |  |
| 10206 | TTHERM_00079610 |  |  |
| 10207 | TTHERM_00079620 |  |  |
| 10208 | TTHERM_00079630 |  |  |
| 10209 | TTHERM_00079640 |  |  |
| 10210 | TTHERM_00079650 |  |  |
| 10211 | TTHERM_00079660 |  |  |
| 10212 | TTHERM_00079670 |  |  |
| 10584 | TTHERM_00641150 |  |  |
| 10803 | TTHERM_01028740 |  |  |
| 10804 | TTHERM_01028750 |  |  |
| 11826 | TTHERM_00013200 |  |  |
| 11965 | TTHERM_00016590 |  |  |
| 13257 | TTHERM_00069680 |  |  |
| 13595 | TTHERM_00418260 |  |  |
| 14804 | TTHERM_01256490 |  |  |
| 14807 | TTHERM_01256520 |  |  |
| 14973 | TTHERM_00874740 |  |  |
| 14974 | TTHERM_00874750 |  |  |
| 15761 | TTHERM_01001420 |  |  |
| 15946 | TTHERM_00320290 |  |  |
| 16138 | TTHERM_00683010 |  |  |
| 16139 | TTHERM_00683020 |  |  |
| 16143 | TTHERM_00683060 |  |  |
| 16245 | TTHERM_00842600 |  |  |
| 16295 | TTHERM_00083480 |  |  |
| 16297 | TTHERM_00083500 |  |  |
| 17099 | TTHERM_01354260 |  |  |
| 17100 | TTHERM_01354270 |  |  |
| 18272 | TTHERM_00755950 |  |  |
| 18516 | TTHERM_00760310 |  |  |
| 19594 | TTHERM_00486630 |  |  |
| 19864 | TTHERM_00532060 |  |  |
| 20451 | TTHERM_00823810 |  |  |
| 20741 | TTHERM_00149810 |  |  |
| 21700 | TTHERM_01359470 |  |  |
| 21701 | TTHERM_01359480 |  |  |
| 21954 | TTHERM_00895720 |  |  |
| 22705 | TTHERM_00802340 |  |  |
| 22977 | TTHERM_00102770 |  |  |
| 23713 | TTHERM_00908100 |  |  |
| 23714 | TTHERM_00909100 |  |  |
| 23715 | TTHERM_00910100 |  |  |
| 23716 | TTHERM_00911100 |  |  |
| 23778 | TTHERM_00660340 |  |  |
| 23779 | TTHERM_00660350 |  |  |
| 23780 | TTHERM_00660360 |  |  |
| 23782 | TTHERM_00660380 |  |  |
| 23783 | TTHERM_00660390 |  |  |
| 23785 | TTHERM_00660410 |  |  |
| 23786 | TTHERM_00660420 |  |  |
| 23788 | TTHERM_00660440 |  |  |
| 23789 | TTHERM_00660450 |  |  |
| 24088 | TTHERM_00586760 |  |  |
| 24687 | TTHERM_00628610 |  |  |
| 25447 | TTHERM_00735230 |  |  |
| 27689 | TTHERM_01074550 |  |  |
| 28303 | TTHERM_01276400 |  |  |
| 28505 | TTHERM_00161130 |  |  |
| 28717 | TTHERM_00375030 |  |  |
| 29570 | TTHERM_00847000 |  |  |
| 30108 | TTHERM_00945290 |  |  |
| 30109 | TTHERM_00945300 |  |  |
| 1675 | TTHERM_01261780 |  | C2 (calpain family) |
| 4024 | TTHERM_00825600 |  |  |
| 4074 | TTHERM_00584660 |  |  |
| 4625 | TTHERM_00529560 |  |  |
| 5944 | TTHERM_01084250 |  |  |
| 6850 | TTHERM_00196650 |  |  |
| 8285 | TTHERM_00979860 |  |  |
| 8733 | TTHERM_00190820 |  |  |
| 11370 | TTHERM_00051890 |  |  |
| 12385 | TTHERM_00522700 |  |  |
| 13098 | TTHERM_00059140 |  |  |
| 14651 | TTHERM_00885870 |  |  |
| 16156 | TTHERM_00683190 |  |  |
| 16448 | TTHERM_00086990 |  |  |
| 17895 | TTHERM_00931970 |  |  |
| 19047 | TTHERM_00715730 |  |  |
| 19628 | TTHERM_00486970 |  |  |
| 19643 | TTHERM_00487120 |  |  |
| 20287 | TTHERM_00455580 |  |  |
| 20392 | TTHERM_01108610 |  |  |
| 20534 | TTHERM_00898290 |  |  |
| 20869 | TTHERM_00152110 |  |  |
| 22532 | TTHERM_00471200 |  |  |
| 24797 | TTHERM_00387030 |  |  |
| 25923 | TTHERM_00287920 |  |  |
| 29310 | TTHERM_00259450 |  |  |
| 2404 | TTHERM_00245080 |  | C12 (ubiquitin C-terminal hydrolase 1 family) |
| 3166 | TTHERM_00535810 |  |  |
| 20450 | TTHERM_00823800 |  |  |
| 9578 | TTHERM_01035670 |  | C13 (legumain) |
| 13179 | TTHERM_00066890 |  |  |
| 13180 | TTHERM_00066900 |  |  |
| 13531 | TTHERM_00415590 |  |  |
| 13532 | TTHERM_00415600 |  |  |
| 5319 | TTHERM_00349070 |  | C15 (pyroglutamyl-peptidase) |
| 6462 | TTHERM_00527300 |  |  |
| 1677 | TTHERM_01261800 |  | C19 (ubiquitin-specific protease family) |
| 2303 | TTHERM_00475040 |  |  |
| 2729 | TTHERM_01205390 |  |  |
| 2953 | TTHERM_00691150 |  |  |
| 3107 | TTHERM_00535220 |  |  |
| 3390 | TTHERM_00312010 |  |  |
| 3554 | TTHERM_00313670 |  |  |
| 3865 | TTHERM_00445960 |  |  |
| 3987 | TTHERM_00825230 |  |  |
| 4028 | TTHERM_00825640 |  |  |
| 4235 | TTHERM_00492990 |  |  |
| 5287 | TTHERM_00348750 |  |  |
| 5635 | TTHERM_00442350 |  |  |
| 5652 | TTHERM_00442520 |  |  |
| 6060 | TTHERM_00338180 |  |  |
| 6672 | TTHERM_00193880 |  |  |
| 7984 | TTHERM_00499410 |  |  |
| 7994 | TTHERM_00499510 |  |  |
| 8174 | TTHERM_00757780 |  |  |
| 8638 | TTHERM_00188880 |  |  |
| 10083 | TTHERM_00077370 |  |  |
| 10309 | TTHERM_00378550 |  |  |
| 10431 | TTHERM_00568020 |  |  |
| 11183 | TTHERM_00047040 |  |  |
| 11249 | TTHERM_00047700 |  |  |
| 11266 | TTHERM_00048860 |  |  |
| 13219 | TTHERM_00069290 |  |  |
| 15498 | TTHERM_00450840 |  |  |
| 15504 | TTHERM_00450900 |  |  |
| 15509 | TTHERM_00450950 |  |  |
| 16047 | TTHERM_00324420 |  |  |
| 16247 | TTHERM_00842620 |  |  |
| 18166 | TTHERM_00684530 |  |  |
| 18484 | TTHERM_00721760 |  |  |
| 20216 | TTHERM_01289070 |  |  |
| 20406 | TTHERM_01108760 |  |  |
| 20970 | TTHERM_00316590 |  |  |
| 21492 | TTHERM_00408760 |  |  |
| 21962 | TTHERM_00895800 |  |  |
| 22539 | TTHERM_00471280 |  |  |
| 22600 | TTHERM_00471920 |  |  |
| 22922 | TTHERM_00101230 |  |  |
| 24119 | TTHERM_00590040 |  |  |
| 26019 | TTHERM_00290870 |  |  |
| 26941 | TTHERM_01094790 |  |  |
| 28526 | TTHERM_00161340 |  |  |
| 29070 | TTHERM_01008660 |  |  |
| 15438 | TTHERM_00449200 |  | C26 (gamma-glutamyl hydrolase ) |
| 17976 | TTHERM_01027470 |  |  |
| 5125 | TTHERM_00621280 |  | C48 (Ulp1 endopeptidase) |
| 5232 | TTHERM_00348200 |  |  |
| 5512 | TTHERM_00440580 |  |  |
| 5648 | TTHERM_00442480 |  |  |
| 10063 | TTHERM_00077170 |  |  |
| 15727 | TTHERM_00297160 |  | C50 (separase) |
| 6365 | TTHERM_00526270 |  | C54 (ATG4 peptidase) |
| 7375 | TTHERM_00622880 |  |  |
| 7376 | TTHERM_00622890 |  |  |
| 11713 | TTHERM_00011010 |  |  |
| 11714 | TTHERM_00011020 |  |  |
| 3333 | TTHERM_00766400 |  | C56 (PfpI endopeptidase family) |
| 2167 | TTHERM_00658820 | **Metallo** | M1 (aminopeptidase N ) |
| 2307 | TTHERM_00475080 |  |  |
| 3008 | TTHERM_00691700 |  |  |
| 5337 | TTHERM_00352220 |  |  |
| 5360 | TTHERM_00353460 |  |  |
| 5799 | TTHERM_00579060 |  |  |
| 5800 | TTHERM_00579070 |  |  |
| 8523 | TTHERM_00786960 |  |  |
| 8524 | TTHERM_00786970 |  |  |
| 10463 | TTHERM_00569330 |  |  |
| 19709 | TTHERM_00637060 |  |  |
| 19711 | TTHERM_00637080 |  |  |
| 25883 | TTHERM_00285540 |  |  |
| 4291 | TTHERM_00494630 |  | M8 (leishmanolysin family) |
| 5544 | TTHERM_01009860 |  |  |
| 5545 | TTHERM_01009870 |  |  |
| 8290 | TTHERM_00979910 |  |  |
| 8291 | TTHERM_00979920 |  |  |
| 8292 | TTHERM_00979930 |  |  |
| 8293 | TTHERM_00979940 |  |  |
| 8294 | TTHERM_00979950 |  |  |
| 9043 | TTHERM_00857790 |  |  |
| 9384 | TTHERM_00127210 |  |  |
| 12724 | TTHERM_00400800 |  |  |
| 14964 | TTHERM_00873660 |  |  |
| 14965 | TTHERM_00873670 |  |  |
| 14998 | TTHERM_00541520 |  |  |
| 14999 | TTHERM_00541530 |  |  |
| 15000 | TTHERM_00541540 |  |  |
| 15001 | TTHERM_00541550 |  |  |
| 15002 | TTHERM_00541560 |  |  |
| 15003 | TTHERM_00542560 |  |  |
| 15004 | TTHERM_00543560 |  |  |
| 15005 | TTHERM_00543570 |  |  |
| 15006 | TTHERM_00543580 |  |  |
| 15910 | TTHERM_00319900 |  |  |
| 16447 | TTHERM_00086980 |  |  |
| 17860 | TTHERM_01082860 |  |  |
| 18490 | TTHERM_00721820 |  |  |
| 19401 | TTHERM_00302090 |  |  |
| 20610 | TTHERM_00147520 |  |  |
| 21433 | TTHERM_00794340 |  |  |
| 24740 | TTHERM_01102690 |  |  |
| 25973 | TTHERM_00289410 |  |  |
| 25974 | TTHERM_00289430 |  |  |
| 25982 | TTHERM_00289510 |  |  |
| 25983 | TTHERM_00289520 |  |  |
| 25984 | TTHERM_00289530 |  |  |
| 25985 | TTHERM_00289540 |  |  |
| 25986 | TTHERM_00289550 |  |  |
| 25987 | TTHERM_00289560 |  |  |
| 25988 | TTHERM_00289570 |  |  |
| 25990 | TTHERM_00289590 |  |  |
| 25991 | TTHERM_00289600 |  |  |
| 25992 | TTHERM_00289610 |  |  |
| 25993 | TTHERM_00290610 |  |  |
| 25995 | TTHERM_00290630 |  |  |
| 26990 | TTHERM_00942690 |  |  |
| 27090 | TTHERM_00112760 |  |  |
| 27654 | TTHERM_01731490 |  |  |
| 29718 | TTHERM_00166110 |  |  |
| 2302 | TTHERM_00475030 |  | M14 (carboxypeptidase A) |
| 3054 | TTHERM_01124000 |  |  |
| 3643 | TTHERM_00655450 |  |  |
| 4637 | TTHERM_00529680 |  |  |
| 5794 | TTHERM_00579010 |  |  |
| 6465 | TTHERM_00527330 |  |  |
| 7286 | TTHERM_00727850 |  |  |
| 8130 | TTHERM_01068150 |  |  |
| 9020 | TTHERM_00566800 |  |  |
| 10147 | TTHERM_00079010 |  |  |
| 13894 | TTHERM_00767550 |  |  |
| 15422 | TTHERM_00449040 |  |  |
| 16134 | TTHERM_00682970 |  |  |
| 16281 | TTHERM_00083340 |  |  |
| 17365 | TTHERM_00929530 |  |  |
| 19094 | TTHERM_00716220 |  |  |
| 20975 | TTHERM_00316640 |  |  |
| 21022 | TTHERM_00317220 |  |  |
| 21415 | TTHERM_00794160 |  |  |
| 21535 | TTHERM_00410180 |  |  |
| 21864 | TTHERM_00725980 |  |  |
| 22132 | TTHERM_00600690 |  |  |
| 24521 | TTHERM_00835060 |  |  |
| 25956 | TTHERM_00289240 |  |  |
| 26186 | TTHERM_00344070 |  |  |
| 29218 | TTHERM_00551140 |  |  |
| 29906 | TTHERM_00185840 |  |  |
| 30315 | TTHERM_00332080 |  |  |
| 22372 | TTHERM_00219440 |  | M15 (D-Ala-D-Ala carboxypeptidase family) |
| 24335 | TTHERM_00136100 |  |  |
| 1072 | TTHERM_00670480 |  | M16 (pitrilysin family) |
| 3752 | TTHERM_00939020 |  |  |
| 4769 | TTHERM_00277260 |  |  |
| 7399 | TTHERM_00624110 |  |  |
| 8077 | TTHERM_00502380 |  |  |
| 8462 | TTHERM_00826840 |  |  |
| 17781 | TTHERM_00836690 |  |  |
| 19331 | TTHERM_00300360 |  |  |
| 19396 | TTHERM_00302040 |  |  |
| 20404 | TTHERM_01108740 |  |  |
| 22823 | TTHERM_00937640 |  |  |
| 22929 | TTHERM_00101300 |  |  |
| 24147 | TTHERM_00590330 |  |  |
| 25767 | TTHERM_00463150 |  |  |
| 28813 | TTHERM_01165230 |  |  |
| 29826 | TTHERM_00181080 |  |  |
| 13046 | TTHERM_00058610 |  | M17 (leucyl aminopeptidase) |
| 29403 | TTHERM_00263380 |  |  |
| 11718 | TTHERM_00011120 |  | M18 (aminopeptidase I) |
| 27522 | TTHERM_00355360 |  |  |
| 27523 | TTHERM_00355370 |  |  |
| 2325 | TTHERM_00475260 |  | M20 (carboxypeptidase family) |
| 6145 | TTHERM_00340040 |  |  |
| 6147 | TTHERM_00340060 |  |  |
| 10323 | TTHERM_00378690 |  |  |
| 10362 | TTHERM_00379080 |  |  |
| 20529 | TTHERM_00898240 |  |  |
| 24467 | TTHERM_00138400 |  |  |
| 5655 | TTHERM_00442550 |  | M22 (O-sialoglycoprotein endopeptidase) |
| 3867 | TTHERM_00445980 |  | M24 (methionyl aminopeptidase) |
| 4838 | TTHERM_00281000 |  |  |
| 4872 | TTHERM_00283330 |  |  |
| 7428 | TTHERM_00624400 |  |  |
| 7431 | TTHERM_00624430 |  |  |
| 7466 | TTHERM_00624780 |  |  |
| 11966 | TTHERM_00016600 |  |  |
| 20120 | TTHERM_00703440 |  |  |
| 22073 | TTHERM_00600100 |  |  |
| 27610 | TTHERM_01004930 |  |  |
| 25110 | TTHERM_00596540 |  | M28 (aminopeptidase) |
| 13597 | TTHERM_00418280 |  | M41 (FtsH endopeptidase) |
| 18351 | TTHERM_00992890 |  |  |
| 19244 | TTHERM_00298420 |  |  |
| 24473 | TTHERM_00138460 |  |  |
| 4684 | TTHERM_00530150 |  | M48 (Ste24 endopeptidase) |
| 28073 | TTHERM_01107240 |  |  |
| 3519 | TTHERM_00313320 |  | M49 (dipeptidyl-peptidase) |
| 11750 | TTHERM_00011440 |  |  |
| 5407 | TTHERM_00437510 | **Serine** | S1 (chymotrypsin family) |
| 19754 | TTHERM_00637510 |  |  |
| 4935 | TTHERM_00283970 |  | S8 (subtilisin family) |
| 6438 | TTHERM_00527000 |  |  |
| 14241 | TTHERM_00030100 |  |  |
| 14242 | TTHERM_00030110 |  |  |
| 14245 | TTHERM_00030140 |  |  |
| 14247 | TTHERM_00030160 |  |  |
| 2363 | TTHERM_00243670 |  | S9 (prolyl oligopeptidase) |
| 12281 | TTHERM_00856550 |  |  |
| 15418 | TTHERM_00449000 |  |  |
| 15419 | TTHERM_00449010 |  |  |
| 24143 | TTHERM_00590290 |  |  |
| 3403 | TTHERM_00312150 |  | S10 (carboxypeptidase Y) |
| 4186 | TTHERM_00492500 |  |  |
| 4457 | TTHERM_00773660 |  |  |
| 4458 | TTHERM_00773670 |  |  |
| 5071 | TTHERM_00617770 |  |  |
| 5073 | TTHERM_00617790 |  |  |
| 8795 | TTHERM_00191440 |  |  |
| 13401 | TTHERM_00361860 |  |  |
| 13406 | TTHERM_00361910 |  |  |
| 15272 | TTHERM_00927190 |  |  |
| 15273 | TTHERM_00927200 |  |  |
| 15274 | TTHERM_00927210 |  |  |
| 15275 | TTHERM_00927220 |  |  |
| 15410 | TTHERM_00448920 |  |  |
| 15411 | TTHERM_00448930 |  |  |
| 15416 | TTHERM_00448980 |  |  |
| 20066 | TTHERM_01141610 |  |  |
| 23018 | TTHERM_00105160 |  |  |
| 25236 | TTHERM_00249550 |  |  |
| 25237 | TTHERM_00249560 |  |  |
| 25238 | TTHERM_00249570 |  |  |
| 25249 | TTHERM_00249680 |  |  |
| 25252 | TTHERM_00249710 |  |  |
| 25261 | TTHERM_00249800 |  |  |
| 26265 | TTHERM_00346900 |  |  |
| 1271 | TTHERM_00780590 |  | S11 (D-Ala-D-Ala carboxypeptidase A) |
| 2797 | TTHERM_00732760 |  |  |
| 3151 | TTHERM_00535660 |  |  |
| 4422 | TTHERM_00773310 |  |  |
| 5488 | TTHERM_00439310 |  |  |
| 11037 | TTHERM_01284710 |  |  |
| 12153 | TTHERM_00538960 |  |  |
| 12323 | TTHERM_00522080 |  |  |
| 19249 | TTHERM_00298470 |  |  |
| 21202 | TTHERM_00923230 |  |  |
| 23150 | TTHERM_00688550 |  |  |
| 27570 | TTHERM_00355840 |  |  |
| 9226 | TTHERM_00649120 |  | S16 (Lon-A peptidase) |
| 22949 | TTHERM_00101500 |  |  |
| 8934 | TTHERM_00563960 |  | S26 (signal peptidase) |
| 16929 | TTHERM_01093560 |  |  |
| 3587 | TTHERM_01085520 |  | S28 (lysosomal Pro-Xaa carboxypeptidase) |
| 4666 | TTHERM_00529970 |  |  |
| 12880 | TTHERM_00653930 |  |  |
| 14199 | TTHERM_00028690 |  |  |
| 14200 | TTHERM_00028700 |  |  |
| 14401 | TTHERM_00035670 |  |  |
| 11281 | TTHERM_00049020 |  | S33 (prolyl aminopeptidase) |
| 18545 | TTHERM_00760600 |  |  |
| 18857 | TTHERM_00239190 |  |  |
| 6452 | TTHERM_00527200 |  | S45 (penicillin G acylase precursor) |
| 9272 | TTHERM_00125100 |  |  |
| 9273 | TTHERM_00125110 |  |  |
| 9803 | TTHERM_00266690 |  |  |
| 20523 | TTHERM_00898180 |  |  |
| 21036 | TTHERM_00317360 |  |  |
| 19967 | TTHERM_00534100 |  | S49 (signal peptide peptidase) |
| 24870 | TTHERM_00389740 |  |  |
| 26600 | TTHERM_00140950 |  |  |
| 4735 | TTHERM_00530720 |  | S59 (nucleoporin) |
| 10359 | TTHERM_00379050 | **Threonine** | T1A (proteasome, beta component) |
| 14275 | TTHERM_00030440 |  |  |
| 15065 | TTHERM_00548160 |  |  |
| 28022 | TTHERM_00633530 |  |  |
| 28411 | TTHERM_00158210 |  |  |
| 23909 | TTHERM_00666560 |  |  |
| 27812 | TTHERM_01050690 |  |  |
| 11065 | TTHERM_00043880 |  |  |
| 19642 | TTHERM_00487110 |  |  |
| 22479 | TTHERM_00470650 |  |  |
| 20614 | TTHERM_00147560 |  |  |
| 23090 | TTHERM_00106960 |  |  |
| 20964 | TTHERM_00316530 |  |  |
| 19960 | TTHERM_00534030 |  | T1B (HslV component of HslUV peptidase) |

D. ID numbers for all *T. thermophila* genes encoding proteins with cytoskeletal-related functions referred to in Table 8. First number, Preliminary TIGR ID. Second number, Genbank Accession.

**actin and actin related**

1384-TTHERM_00790940

3387-TTHERM_00311980

5610-TTHERM_00442100

8746-TTHERM_00190950

9018-TTHERM_00566780

10312-TTHERM_00378580

15617-TTHERM_00295070

17635-TTHERM_01020680

21000-TTHERM_00317000

21351-TTHERM_00310470

24270-TTHERM_00133470

27636-TTHERM_01005190

28168-TTHERM_00975380

28662-TTHERM_00372480

**profilin**

23043-TTHERM_00105430

**cofilin**

21414-TTHERM_00794150

**CapZlike**

27135-TTHERM_00113210

**paxillin**

26633-TTHERM_00142270

**Fimbrin**

24376-TTHERM_00136510

**α - tubulin**

7958-TTHERM_00558620

**α - tubulinlike**

15819-TTHERM_00647130

21179-TTHERM_00923000

19813-TTHERM_01054190

**β - tubulin**

5263-TTHERM_00348510

**β- tubulinlike**

1888-TTHERM_01104960

1889-TTHERM_01104970

1890-TTHERM_01104980

18246-TTHERM_01120580

24577-TTHERM_01620840

24511-TTHERM_00834960

**γ - tubulin**

10197-TTHERM_00079520

**ε - tubulin**

6040-TTHERM_00335970

**δ - tubulin**

27186-TTHERM_00118700

**η- tubulin**

9801-TTHERM_00266660

**ι - tubulin**

15746-TTHERM_01001260

15745-TTHERM_01001250

29199-TTHERM_00550910

**TPX2 like**

13215-TTHERM_00069250

**XMAP215**

5458-TTHERM_00439010

8322-TTHERM_00488190

**EB1**

5764-TTHERM_00578710

7942-TTHERM_00558460

27058-TTHERM_00112440

24293-TTHERM_00133700

1971-TTHERM_01092350

6207-TTHERM_00476790

28993-TTHERM_00577060

**centrin**

24783-TTHERM_00384910

12421-TTHERM_00523060

23181-TTHERM_00689850

3855-TTHERM_00444870

6717-TTHERM_00194330

28617-TTHERM_00371010

**katanin p60**

15995-TTHERM_00322760

13501-TTHERM_00414230

**kinesin heavy chain**

3803-TTHERM_00444350

4272-TTHERM_00494380

4311-TTHERM_00494840

4329-TTHERM_01142740

4843-TTHERM_00283030

5241-TTHERM_00348290

5616-TTHERM_00442160

6254-TTHERM_00481220

6255-TTHERM_00481230

6651-TTHERM_00193680

7195-TTHERM_00420700

7346-TTHERM_01245620

7691-TTHERM_01347910

7929-TTHERM_00558320

8098-TTHERM_00502590

8991-TTHERM_00564530

9312-TTHERM_00125500

9449-TTHERM_00128850

9577-TTHERM_01035660

9757-TTHERM_00266230

10348-TTHERM_00378940

11898-TTHERM_00014920

12384-TTHERM_00522690

12566-TTHERM_00227560

12668-TTHERM_00399210

12786-TTHERM_00405390

12789-TTHERM_00405420

13014-TTHERM_00058290

13299-TTHERM_00071090

14757-TTHERM_01188400

15731-TTHERM_00297200

15992-TTHERM_00321740

16107-TTHERM_00681700

16125-TTHERM_00681890

16205-TTHERM_00841200

16410-TTHERM_00085620

16897-TTHERM_00713460

17359-TTHERM_00929470

18479-TTHERM_00721710

18731-TTHERM_00469290

18818-TTHERM_00238790

18837-TTHERM_00238980

19345-TTHERM_00300500

19349-TTHERM_00300540

19386-TTHERM_00301900

19538-TTHERM_00486060

20875-TTHERM_00152170

21073-TTHERM_00318720

21406-TTHERM_00794070

22386-TTHERM_00219590

23055-TTHERM_00105550

24380-TTHERM_00137540

24831-TTHERM_00388360

25951-TTHERM_00289190

26242-TTHERM_00346610

27157-TTHERM_00115410

28029-TTHERM_00633600

28876-TTHERM_01216100

29398-TTHERM_00263330

29858-TTHERM_00185360

29877-TTHERM_00185550

1384-TTHERM_00790940

1812-TTHERM_01041960

8544-TTHERM_00787170

8981-TTHERM_00564430

9090-TTHERM_00859280

9175-TTHERM_00648540

15553-TTHERM_00293440

15631-TTHERM_00295210

15647-TTHERM_00295370

19778-TTHERM_00637750

20388-TTHERM_01108570

21462-TTHERM_00794630

21463-TTHERM_00794640

21531-TTHERM_00410140

22322-TTHERM_00218940

25564-TTHERM_00784600

26427-TTHERM_00429870

**dynein heavy chain**

1882-TTHERM_01104900

4219-TTHERM_00492830

6635-TTHERM_00193520

7050-TTHERM_00919540

7159-TTHERM_00420340

7756-TTHERM_00821980

7928-TTHERM_00558310

7960-TTHERM_00558640

7973-TTHERM_00499300

8999-TTHERM_00565600

11110-TTHERM_00046310

11233-TTHERM_00047540

17996-TTHERM_01027670

19591-TTHERM_00486600

19663-TTHERM_00947430

19847-TTHERM_00531870

20185-TTHERM_01151430

21093-TTHERM_00774810

23142-TTHERM_00688470

23736-TTHERM_00912290

24963-TTHERM_00850620

25325-TTHERM_00252430

26521-TTHERM_00433800

27496-TTHERM_00355100

28305-TTHERM_01276420

**myosin heavy chain**

6415-TTHERM_00526770

6689-TTHERM_00194050

9585-TTHERM_01035740

9972-TTHERM_00074290

11126-TTHERM_00046470

13089-TTHERM_00059050

13970-TTHERM_00423370

15526-TTHERM_00454090

22140-TTHERM_00600770

22141-TTHERM_00600780

25768-TTHERM_00463160

27057-TTHERM_00112430

30235-TTHERM_00327320

**radial spoke head RSP 4/6like**

3786-TTHERM_00444180

5909-TTHERM_01044600

8097-TTHERM_00502580

9021-TTHERM_00566810

13596-TTHERM_00418270

14095-TTHERM_00427590

**PF16**

28384-TTHERM_00157940

**PF20**

24313-TTHERM_00134890

**IFT20**

5995-TTHERM_00334500

**IFT52**

9205-TTHERM_00648910

**IFT57**

1565-TTHERM_01298520

**IFT72**

20682-TTHERM_00149230

**IFT81**

15125-TTHERM_01013160

**IFT88**

4327-TTHERM_01142720

9434-TTHERM_00128700

**IFT140**

22409-TTHERM_00220810

**IFT172**

16475-TTHERM_00089240

**tubulin deacetylase HDAC6**

5158-TTHERM_00621610

4600-TTHERM_01014700

**tubulin tyrosine ligaselike**

1191-TTHERM_00852920

2322-TTHERM_00475230

4399-TTHERM_00773080

4715-TTHERM_00530520

4940-TTHERM_00284020

5212-TTHERM_00348000

5441-TTHERM_00438840

5646-TTHERM_00442460

5949-TTHERM_01084300

6640-TTHERM_00193570

7407-TTHERM_00624190

8075-TTHERM_00502360

8194-TTHERM_00758970

8948-TTHERM_00564100

9091-TTHERM_00859290

9322-TTHERM_00125600

10075-TTHERM_00077290

10329-TTHERM_00378750

12774-TTHERM_00404280

12825-TTHERM_01341600

13198-TTHERM_00068090

13589-TTHERM_00418200

13915-TTHERM_00770730

14402-TTHERM_00035680

14922-TTHERM_00702240

15101-TTHERM_00549600

15690-TTHERM_00295800

17798-TTHERM_00837850

17951-TTHERM_01109810

18814-TTHERM_00238750

19263-TTHERM_00299600

19884-TTHERM_00532260

20714-TTHERM_00149550

20934-TTHERM_00316230

21494-TTHERM_00408780

21687-TTHERM_01295310

22042-TTHERM_00599750

23270-TTHERM_00985000

23744-TTHERM_00913400

23801-TTHERM_00661560

23913-TTHERM_00666600

24087-TTHERM_00586750

24346-TTHERM_00136210

25002-TTHERM_00997770

25570-TTHERM_00784660

26740-TTHERM_00145320

26863-TTHERM_00705080

26996-TTHERM_00942750

29742-TTHERM_00170310

30214-TTHERM_00327110

**NIMArelated kinases**

1112-TTHERM_00670880

1405-TTHERM_01144900

1924-TTHERM_01114180

2122-TTHERM_00657380

2218-TTHERM_00473180

2469-TTHERM_00245740

3425-TTHERM_00312380

3979-TTHERM_00825150

4647-TTHERM_00529780

6348-TTHERM_00525110

7740-TTHERM_00821800

8589-TTHERM_00188390

10831-TTHERM_01030010

13547-TTHERM_00415780

15435-TTHERM_00449170

15734-TTHERM_01000150

17542-TTHERM_00697500

17999-TTHERM_01027700

18448-TTHERM_00721400

18676-TTHERM_00467740

19380-TTHERM_00301840

19674-TTHERM_00947540

20296-TTHERM_00455670

20809-TTHERM_00151480

21625-TTHERM_00412070

21626-TTHERM_00412080

22242-TTHERM_00216170

22348-TTHERM_00219200

23433-TTHERM_00809380

24670-TTHERM_00628440

24902-TTHERM_00390060

24991-TTHERM_00997660

26810-TTHERM_00146030

28433-TTHERM_00158430

28502-TTHERM_00161100

29340-TTHERM_00260770

29341-TTHERM_00260780

29473-TTHERM_00265180

**Aurora kinase**

2219-TTHERM_00473190

2224-TTHERM_00473240

5973-TTHERM_00334280

9766-TTHERM_00266320

12754-TTHERM_00402100

13285-TTHERM_00070950

13287-TTHERM_00070970

15246-TTHERM_00926910

16602-TTHERM_01350000

18172-TTHERM_00684590

19498-TTHERM_01266090

19636-TTHERM_00487050

22429-TTHERM_00221010

25157-TTHERM_00941510

26118-TTHERM_00343380

26122-TTHERM_00343420

**Polo kinase**

5034-TTHERM_01046870

8237-TTHERM_01076980

8238-TTHERM_01076990

8635-TTHERM_00188850

8829-TTHERM_00191790

18358-TTHERM_00992970

18380-TTHERM_01443850

22503-TTHERM_00470890
