# Supplementary material for: Macronuclear Genome Sequence of the Ciliate Tetrahymena thermophila, a Model Eukaryote
Source: PLoS Biol. 2006 Aug 29;4(9):e286. doi: 10.1371/journal.pbio.0040286 (PMC1557398; doi:10.1371/journal.pbio.0040286)
Supplement: Supplementary file 19 [file pbio.0040286.st012.doc]

Table S12. Human disease genes with orthologs in *T. thermophila*, but not *S. cerevisiae*. Best hits in *T. thermophila* are listed, but many of the human genes have multiple *T. thermophila* counterparts.

| **Human ENSEMBL ID** | **Preliminary TIGR ID** | **Genbank Accession** | **OMIM ID** | **Disease Description** |
| --- | --- | --- | --- | --- |
| ENSG00000134575 | 18037 | TTHERM_01193550 | 171650 | Lysosomal acid phosphatase deficiency |
| ENSG00000128683 | 25940 | TTHERM_00289080 | 266100 | Pyridoxine dependency with seizures |
| ENSG00000105607 | 24442 | TTHERM_00138150 | 231670 | Glutaricaciduria, type I |
| ENSG00000171298 | 3149 | TTHERM_00535640 | 232300 | Glycogen storage disease II |
| ENSG00000171403 | 27743 | TTHERM_01018250 | 144200 | Epidermolytic palmoplantar keratoderma |
| ENSG00000198650 | 11097 | TTHERM_00046180 | 276600 | Tyrosinemia, type II |
| ENSG00000164258 | 6431 | TTHERM_00526930 | 602694 | Complex I deficiency |
| ENSG00000167792 | 6675 | TTHERM_00193910 | 161015 | Mitochondrial complex I deficiency |
| ENSG00000113966 | 3168 | TTHERM_00535830 | 600151 | Bardet-Biedl syndrome 3 |
| ENSG00000166228 | 5948 | TTHERM_01084290 | 126090 | Hyperphenylalaninemia due to pterin-4a-carbinolamine dehydratase deficiency |
| ENSG00000139687 | 5460 | TTHERM_00439030 | 180200 | Retinoblastoma |
| ENSG00000046651 | 13858 | TTHERM_00214750 | 311200 | Oral-facial-digital syndrome 1 |
| ENSG00000149925 | 19640 | TTHERM_00487090 | 103850 | Aldolase A deficiency |
| ENSG00000108784 | 25879 | TTHERM_00285500 | 252920 | Sanfilippo syndrome, type B |
| ENSG00000135636 | 5514 | TTHERM_00440600 | 253601 | Muscular dystrophy, limb-girdle, type 2B |
| ENSG00000115155 | 5514 | TTHERM_00440600 | 601071 | Deafness, autosomal recessive 9 |
| ENSG00000166147 | 20568 | TTHERM_01098980 | 134797 | Shprintzen-Goldberg syndrome |
| ENSG00000143387 | 9463 | TTHERM_00128990 | 601105 | Pycnodysostosis |
| ENSG00000003393 | 10081 | TTHERM_00077350 | 205100 | Amyotrophic lateral sclerosis, juvenile |
| ENSG00000158104 | 7590 | TTHERM_00678260 | 276710;122000 | Tyrosinemia, type III (1);Corneal dystrophy, posterior polymorphous |
| ENSG00000113924 | 3891 | TTHERM_00446240 | 203500 | Alkaptonuria |
| ENSG00000023228 | 6710 | TTHERM_00194260 | 157655 | Lactic acidosis due to defect in iron-sulfur cluster of complex I |
| ENSG00000122126 | 24073 | TTHERM_00586610 | 309000 | Lowe syndrome |
| ENSG00000083123 | 20176 | TTHERM_00704010 | 248611 | Maple syrup urine disease, type Ib |
| ENSG00000140463 | 19822 | TTHERM_01054280 | 600374 | Bardet-Biedl syndrome 4 |
| ENSG00000103876 | 11077 | TTHERM_00044990 | 276700 | Tyrosinemia, type I |
| ENSG00000131238 | 24180 | TTHERM_00591650 | 600722 | Ceroid lipofuscinosis, neuronal-1, infantile |
| ENSG00000188128 | 3891 | TTHERM_00446240 | 203500 | Alkaptonuria |
| ENSG00000198515 | 5628 | TTHERM_00442280 | 123825 | Retinitis pigmentosa, autosomal recessive |
| ENSG00000102393 | 24330 | TTHERM_00136050 | 301500 | Fabry disease |
| ENSG00000104267 | 20775 | TTHERM_00151140 | 259730 | Renal tubular acidosis-osteopetrosis syndrome |
| ENSG00000188419 | 19347 | TTHERM_00300520 | 303100 | Choroideremia |
| ENSG00000108395 | 6192 | TTHERM_00476630 | 253250 | Mulibrey nanism |
| ENSG00000148175 | 22548 | TTHERM_00471380 | 185000 | Stomatocytosis I |
| ENSG00000049860 | 4314 | TTHERM_00494870 | 268800 | Spinal muscular atrophy, HEXB-related |
| ENSG00000125124 | 25838 | TTHERM_00463860 | 209900 | Bardet-Biedl syndrome 2 |
| ENSG00000118762 | 3820 | TTHERM_00444520 | 173910 | Polycystic kidney disease, adult, type II |
| ENSG00000109323 | 24424 | TTHERM_00137980 | 248510 | Mannosidosis, beta- |
| ENSG00000151729 | 13437 | TTHERM_00363210 | 601226 | Progressive external ophthalmoplegia, type 2 |
| ENSG00000151552 | 22399 | TTHERM_00220710 | 261630 | Phenylketonuria due to dihydropteridine reductase deficiency |
| ENSG00000142046 | 30160 | TTHERM_00325580 | 248600 | Maple syrup urine disease, type Ia |
| ENSG00000137992 | 21453 | TTHERM_00794540 | 248610 | Maple syrup urine disease, type II |
| ENSG00000128928 | 17625 | TTHERM_01019590 | 243500 | Isovalericacidemia |
| ENSG00000104774 | 12609 | TTHERM_00230960 | 248500 | Mannosidosis, alpha- |
| ENSG00000170266 | 15973 | TTHERM_00321550 | 230500 | Mucopolysaccharidosis IVB |
| ENSG00000092529 | 6850 | TTHERM_00196650 | 114240 | Muscular dystrophy, limb-girdle, type 2A |
| ENSG00000164751 | 14170 | TTHERM_00833870 | 170993 | Zellweger syndrome-3 |
| ENSG00000042781 | 27003 | TTHERM_00942830 | 276901 | Usher syndrome, type 2 |
| ENSG00000106992 | 21020 | TTHERM_00317200 | 103000 | Hemolytic anemia due to adenylate kinase deficiency |
| ENSG00000121031 | 13773 | TTHERM_00203010 | 600899 | Severe combined immunodeficiency, type I |
| ENSG00000090402 | 11895 | TTHERM_00013890 | 222900 | Sucrose intolerance |
| ENSG00000198951 | 11054 | TTHERM_00043770 | 104170 | Schindler disease |
| ENSG00000196569 | 27003 | TTHERM_00942830 | 156225 | Muscular dystrophy, congenital merosin-deficient |
| ENSG00000107815 | 3009 | TTHERM_00691710 | 157640 | PEO with mitochondrial DNA deletions, type 1 |
| ENSG00000083720 | 5618 | TTHERM_00442180 | 245050 | Ketoacidosis due to SCOT deficiency |
| ENSG00000156113 | 15281 | TTHERM_00927280 | 270400 | Smith-Lemli-Opitz syndrome |
| ENSG00000117305 | 18224 | TTHERM_00686100 | 246450 | HMG-CoA lyase deficiency |
| ENSG00000109861 | 3861 | TTHERM_00445920 | 245000 | Papillon-Lefevre syndrome |
