# Supplementary material for: Macronuclear Genome Sequence of the Ciliate Tetrahymena thermophila, a Model Eukaryote
Source: PLoS Biol. 2006 Aug 29;4(9):e286. doi: 10.1371/journal.pbio.0040286 (PMC1557398; doi:10.1371/journal.pbio.0040286)
Supplement: Supplementary file 20 [file pbio.0040286.st013.doc]

Table S13. Dynein subunit genes in *T. thermophila.* Expression of all genes except LC3A (n/d) was confirmed by RNA-directed PCR; when possible, primers spanned introns in order to readily distinguish the amplified products from genomic vs. cDNAs. The TTHERM Genbank accession numbers refer to TIGR-generated gene models. Older Genbank accession numbers refer to experimentally derived gene coding sequences.

| ***T. thermophila* subunit** | **Complex^1^** | **Preliminary TIGR Gene ID** | **Genbank accession** | **Older Genbank accession** |
| --- | --- | --- | --- | --- |
| *Light chains***^2^** |  |  |  |  |
| LC1 | OAD | 5974 | TTHERM_00334290 |  |
| LC2A (LC2) **^3^** | OAD | 3792 | TTHERM_00444240 |  |
| LC2B(Tctex2B) | IADI1 | 27905 | TTHERM_00561440 |  |
| LC3A | OAD | 23850 | TTHERM_00664030 |  |
| LC3B | OAD | 7392 | TTHERM_00623050 |  |
| LC4A | OAD | 2674 | TTHERM_01075620 |  |
| LC4B | OAD | 17631 | TTHERM_01020640 |  |
| LC7A | OAD, IADI1, cyto1 | 20862 | TTHERM_00152040 |  |
| LC7B | OAD, IADI1, cyto1 | 14252 | TTHERM_00030210 |  |
| LC8 | OAD, cyto1 | 9257 | TTHERM_00649430 |  |
| LC8-like B | ? | 25803 | TTHERM_00463510 |  |
| LC8-like C | ? | 15421 | TTHERM_00449030 |  |
| LC8-like D | ? | 18307 | TTHERM_00756440 |  |
| LC8-like E | ? | 17673 | TTHERM_01079060 |  |
| LC8-like F | ? | 1454 | TTHERM_00971840 |  |
| LC10**^4^** | OAD | 19097 | TTHERM_00716250 |  |
| Tctex1A | IADI1, cyto1, OAD | 19512 | TTHERM_00485800 |  |
| Tctex1B | IADI1, cyto1, OAD | 25508 | TTHERM_00782060 |  |
| p28A | IADI2 | 16206 | TTHERM_00841210 |  |
| p28B | IADI2 | 15918 | TTHERM_00319990 |  |
| p28C | IADI2 | 30052 | TTHERM_01129720 |  |
| *Light-intermediate chains***^5^** |  |  |  |  |
| D1LIC | cyto1 | 15604 | TTHERM_00294940 |  |
| D2LIC | cyto2 | 4922 | TTHERM_00283830 | AY842288 |
| *Intermediate chains***^5^** |  |  |  |  |
| IC2/IC78 | OAD | 19646 | TTHERM_00487150 |  |
| IC3/IC69 | OAD | 10169 | TTHERM_00079230 |  |
| IC138 | IADI1 | 3910 | TTHERM_00446430 |  |
| IC140 | IADI1 | 6823 | TTHERM_00196380 |  |
| IC74 | cyto1 | 4466 | TTHERM_00773750 |  |
| *Heavy chains* |  |  |  |  |
| DYH1 | cyto1 | 11110 | TTHERM_00046310 | AF346733 |
| DYH2 | Cyto2 | 7928 | TTHERM_00558310 | AY770505 |
| DYH3 (α) | OAD | 28305 | TTHERM_01276420 | AF152585 |
| DYH4 (β) | OAD | 7973 | TTHERM_00499300 | AF072878 |
| DYH5 (γ) | OAD | 19591 | TTHERM_00486600 | AF153267 |
| DYH6 (1-α) | IADI1 | 23142 | TTHERM_00688470 | AF153268 |
| DYH7 1-β) | IADI1 | 23736 | TTHERM_00912290 | AF153702 |
| DYH8 | IAD**^6^** | 19847 | TTHERM_00531870 | AF153704 |
| DYH9 | IAD | 19663 | TTHERM_00947430 | AF153271 |
| DYH10 | IAD | 7159 | TTHERM_00420340 | AF153269 |
| DYH11 | IAD | 25325 | TTHERM_00252430 | AF153270 |
| DYH12 | IAD | 7050 | TTHERM_00919540 | AF153703 |
| DYH13 | IAD | 1882 | TTHERM_01104900 | AF153705 |
| DYH14 | IAD | 4219 | TTHERM_00492830 | DQ438945 |
| DYH15 | IAD | 26521 | TTHERM_00433800 | DQ386633 |
| DYH16 | IAD | 7960 | TTHERM_00558640 | DQ386634 |
| DYH17 | IAD | 24963 | TTHERM_00850620 | DQ395125 |
| DYH18 | IAD | 11305 | TTHERM_00047540 | DQ395126 |
| DYH19 | IAD | 17996 | TTHERM_01027670 | DQ395127 |
| DYH20 | IAD | 7756 | TTHERM_00821980 | DQ395128 |
| DYH21 | IAD | 20185 | TTHERM_01151430 | DQ395129 |
| DYH22 | IAD | 8999 | TTHERM_00565600 | DQ395130 |
| DYH23 | IAD | 27496 | TTHERM_00355100 | DQ404154 |
| DYH24 | IAD | 6635 | TTHERM_00193520 | DQ404155 |
| DYH25 | IAD | 21093 | TTHERM_00774810 | DQ375457 |

Dynein complexes: cyto1 and cyto2, non-axonemal dyneins -1 and -2; OAD, three-headed ciliary outer arm dynein; IAD, ciliary inner arm dynein; IADI1, ciliary two-headed inner arm dynein complex 1; IADI2, inner arm dynein complex 2 (see [1]).

^2^ Dynein light chains use the nomenclature established for *Chlamydomonas* dyneins [2,3].

^3^ LC2A is LC2 in *Chlamydomonas*; LC2B is Tctex2B in *Chlamydomonas*.

^4^ *T. thermophila* LC10 is the ortholog of sea urchin and mouse LC4 (accession numbers BAA24152 and BAA24153, respectively).

^5^ Dynein light-intermediate and intermediate chains use the nomenclature established for either *Chlamydomonas* [2] or mammals [4].

^6^ DYH8-25 are presumed to encode single-headed inner arm dyneins.

1. Kamiya R (2002) Functional diversity of axonemal dyneins as studied in Chlamydomonas mutants. Int Rev Cytol 219: 115-155.

2. DiBella LM, Smith EF, Patel-King RS, Wakabayashi K, King SM (2004) A novel Tctex2-related light chain is required for stability of inner dynein arm I1 and motor function in the Chlamydomonas flagellum. J Biol Chem 279: 21666-21676.

3. Pfister KK, Fay RB, Witman GB (1982) Purification and polypeptide composition of dynein ATPases from Chlamydomonas flagella. Cell Motil 2: 525-547.

4. Vale RD (2003) The molecular motor toolbox for intracellular transport. Cell 112: 467-480.
