# Supplementary material for: Macronuclear Genome Sequence of the Ciliate Tetrahymena thermophila, a Model Eukaryote
Source: PLoS Biol. 2006 Aug 29;4(9):e286. doi: 10.1371/journal.pbio.0040286 (PMC1557398; doi:10.1371/journal.pbio.0040286)
Supplement: Supplementary file 21 [file pbio.0040286.st014.doc]

Table S14. Membrane traffic component homologs in *T. thermophila*.

| **Protein** | **Preliminary TIGR ID** | **GenBank Accession** |
| --- | --- | --- |
| Clathrin heavy chain | 9915 | TTHERM_00275740 |
| **AP complexes** |  |  |
| Alpha subunits | 21978 | TTHERM_00896070 |
| Beta subunits | 22365, 28346, 25650 | TTHERM_00219370, TTHERM_00155580, TTHERM_00582140 |
| Mu subunits | 28990, 20393, 20259, 15036 | TTHERM_0057703, TTHERM_01108620, TTHERM_00455300, TTHERM_00545860 |
| Sigma subunits | 17429, 30230 | TTHERM_01079140, TTHERM_0032720 |
| **COPI** |  |  |
| Alpha | 1651 | TTHERM_01345820 |
| Beta | 8338 | TTHERM_00488350 |
| Beta’ | 6180 | TTHERM_00476510 |
| Gamma1 | 3800 | TTHERM_00444320 |
| Delta | 18686 | TTHERM_00467850 |
| Epsilon | 20363 | TTHERM_00459320 |
| Zeta1 | 11440 | TTHERM_00052600 |
| **COPII** |  |  |
| Sec13 | 6716 | TTHERM_00194320 |
| Sec23 | 2275 | TTHERM_00474750 |
| Sec24 | 24470, 26253 | TTHERM_00138430, TTHERM_00346730 |
| **Retromer** |  |  |
| Vps26 | 3054 | TTHERM_01124000 |
| Vps29 | 11242 | TTHERM_00047630 |
| Vps35 | 15622 | TTHERM_00295120 |
