# Supplementary material for: Macronuclear Genome Sequence of the Ciliate Tetrahymena thermophila, a Model Eukaryote
Source: PLoS Biol. 2006 Aug 29;4(9):e286. doi: 10.1371/journal.pbio.0040286 (PMC1557398; doi:10.1371/journal.pbio.0040286)
Supplement: Supplementary file 22 [file pbio.0040286.st015.doc]

Table S15. Rab homologs in the *T. thermophila* genome assembly

| **Name** | **Preliminary TIGR ID** | **GenBank Accession** | **BLASTP value to selected homolog** | **Length (amino acids)** |
| --- | --- | --- | --- | --- |
| **Group I** |  |  |  |  |
| **RAB1** |  |  |  |  |
| Rab1A | 20939 | TTHERM_00316280 | 2e-56 (Rab1A Hs) | 238 |
| Rab1B | 26486 | TTHERM_00433430 | 6e-50 (Rab1B Hs) | 250 |
| Rab1C | 3985 | TTHERM_00825210 | 1e-50 (Rab1A Hs) | 210 |
| Rab1D | 7104 | TTHERM_00419740 | 3e-40 (Rab1A Hs) | 211 |
|  |  |  |  |  |
| **Group II** |  |  |  |  |
| **RAB2** |  |  |  |  |
| Rab2A | 18681 | TTHERM_00467790 | 1e-73 (Rab2A Hs) | 211 |
| Rab2B | 1053 | TTHERM_00670290 | 4e-53 (Rab2 Hs) | 261 |
| Rab2C | 21874 | TTHERM_00726080 | 8e-51 (Rab2 Mm) | 270 |
| Rab2D | 5815 | TTHERM_00579220 | 2e-56 (Rab2 Dr) | 243 |
| Rab2E | 20011 | TTHERM_00994150 | 2e-33 (Rab2 Mm) | 204 |
| Rab2F | 7885 | TTHERM_00557870 | 2e-33 (Rab2 Mm) | 213 |
| **RAB4** |  |  |  |  |
| Rab4A | 11517 | TTHERM_00001080 | 8e-53 (Rab4 Hs) | 212 |
| Rab4B | 26961 | TTHERM_01097960 | 5e-54 (Rab4 Hs) | 212 |
| **RAB11** |  |  |  |  |
| Rab11A | 20971 | TTHERM_00316600 | 4e-69 (Rab11A Hs) | 230 |
| Rab11B | 25160 | TTHERM_00941540 | 7e-66 (Rab11B Hs) | 214 |
| Rab11C | 5211 | TTHERM_00347990 | 2e-40 (Rab11B Hs) | 182 |
| Rab11D | 22521 | TTHERM_00471070 | 3e-47 (Rab11B Hs) | 225 |
| Rab11E | 24892 | TTHERM_00389960 | 1e-47 (Rab11B Hs) | 214 |
| Rab11F | 20594 | TTHERM_01099240 | 6e-39 (Rab11A Hs) | 214 |
| Rab11G | 20020 | TTHERM_00994240 | 2e-33 (Rab11B Hs) | 212 |
|  |  |  |  |  |
| **Group III** |  |  |  |  |
| **RAB3** |  |  |  |  |
| Rab3A | 20702 | TTHERM_00149430 | 9e-42 (Rab3D Hs) | 207 |
|  |  |  |  |  |
| **Group IV** |  |  |  |  |
| ND |  |  |  |  |
|  |  |  |  |  |
| **Group V** |  |  |  |  |
| Rab17A | 21654 | TTHERM_00957580 | 4e-25 (Rab17 Mm) | 198 |
| Rab21A | 12168 | TTHERM_00540100 | 1e-41 (Rab21 Hs) | 207 |
| Rab22 | 21422 | TTHERM_00794230 | 9e-47 (Rab22 Hs) | 197 |
| Rab5A | 16858 | TTHERM_00711980 | 5e-40 (Rab5B Hs) | 195 |
| Rab5B | 5997 | TTHERM_00334520 | 1e-29 (Rab5B Hs) | 255 |
| Rab5C | 19120 | TTHERM_00614720 | 1e-27 (Rab5C Mm) | 217 |
|  |  |  |  |  |
| **Group VI** |  |  |  |  |
| **RAB6** |  |  |  |  |
| Rab6A | 18678 | TTHERM_00467760 | 4e-61 (Rab6A Hs) | 277 |
| Rab6B | 17436 | TTHERM_01079210 | 2e-52 (Rab6A Hs) | 225 |
| Rab6C | 10235 | TTHERM_00079900 | 5e-55 (Rab6A Hs) | 227 |
| Rab6D | 18022 | TTHERM_01164180 | 9e-44 (Rab6A Hs) | 257 |
|  |  |  |  |  |
| **Group VII** |  |  |  |  |
| ND |  |  |  |  |
|  |  |  |  |  |
| **Group VIII** |  |  |  |  |
| **RAB8/13** |  |  |  |  |
| Rab8A | 4816 | TTHERM_00279750 | 4e-51 (Rab13 Mm) | 205 |
| Rab8B | 12482 | TTHERM_00225720 | 2e-44 (Rab8B Mm) | 204 |
| Rab13A | 9358 | TTHERM_00126950 | 5e-37 (Rab13 Dr) | 207 |
|  |  |  |  |  |
| **Misc** |  |  |  |  |
| Rab28 | 18012 | TTHERM_01164080 | 6e-20 (Rab28 Dr) | 237 |
| Rab32 | 1184 | TTHERM_00852850 | 5e-30 (Rab32 Hs) | 233 |
|  |  |  |  |  |
| **NOVEL** |  |  |  |  |
| **Tt Clade I** |  |  |  |  |
|  | 18077 | TTHERM_00723170 | 7e-28 (Rab1A Hs) | 226 |
|  | 15613 | TTHERM_00295030 | 3e-33 (Rab10 Hs) | 215 |
|  | 18061 | TTHERM_00723010 | 6e-19 (Rab10 Hs) | 217 |
|  | 8578 | TTHERM_00188280 | 5e-13 (Rab1B Hs) | 213 |
|  | 13178 | TTHERM_00066880 | 5e-38 (Rab13 Mm) | 229 |
|  |  |  |  |  |
| **Tt Clade II** |  |  |  |  |
|  | 23853 | TTHERM_00664060 | 2e-36 (Rab1A Hs) | 234 |
|  | 19980 | TTHERM_01407960 | 1e-15 (Rab3 Dm) | 170 |
|  | 19979 | TTHERM_01407950 | 2e-13 (Rab3A Hs) | 170 |
|  | 7948 | TTHERM_00558520 | 8e-23 (Rab3C Mm) | 183 |
|  | 13802 | TTHERM_00207260 | 5e-25 (Rab17 Mm) | 219 |
|  | 13805 | TTHERM_00209270 | 3e-27 (Rab17 Hs) | 175 |
|  | 13801 | TTHERM_00207250 | 3e-26 (Rab17 Hs) | 175 |
|  | 13739 | TTHERM_00201680 | Ambiguous scores | 193 |
|  | 13740 | TTHERM_00201690 | Ambiguous scores | 174 |
|  | 13741 | TTHERM_00201700 | Ambiguous scores | 174 |
|  |  |  |  |  |
| **Tt Clade III** |  |  |  |  |
|  | 10943 | TTHERM_00671970 | 9e-23 (Rab13 Hs) | 226 |
|  | 12036 | TTHERM_00024230 | 7e-27 (Rab1A Dr) | 211 |
|  | 26464 | TTHERM_00431230 | 4e-16 (Rab8B Mm) | 223 |
|  | 17445 | TTHERM_01079300 | 8e-21 (Rab15 Mm) | 337 |
|  |  |  |  |  |
| **Various** |  |  |  |  |
|  | 16857 | TTHERM_00711970 | Ambiguous scores | 279 |
|  | 23112 | TTHERM_00107200 | 3e-15 (Rab13 Dr) | 204 |
|  | 19253 | TTHERM_00298510 | 3e-24 (RAYL Hs) | 199 |
|  | 1808 | TTHERM_01041920 | 4e-38 (Rab13 Hs) | 222 |
|  | 7253 | TTHERM_00727520 | 2e-31 (Rab13 Dr) | 189 |
|  | 7255 | TTHERM_00727540 | 2e-40 (Rab13 Dr) | 250 |
|  | 25557 | TTHERM_00784530 | 3e-34 (Rab1B Hs) | 194 |
|  | 30048 | TTHERM_01129680 | 2e-20 (Rab1A Hs) | 183 |
|  | 6730 | TTHERM_00194460 | 6e-26 (Rab35 Hs) | 168 |
|  | 16313 | TTHERM_00083660 | 9e-22 (Rab35 Hs) | 241 |
|  | 16314 | TTHERM_00083670 | 1e-22 (Rab13 Dr) | 172 |
|  | 6077 | TTHERM_00338360 | 3e-26 (Rab2 Mm) | 317 |
|  | 13129 | TTHERM_00059450 | 1e-21 (Rab39B Hs) | 185 |
|  | 19755 | TTHERM_00637520 | 6e-19 (Rab13 Rn) | 216 |
|  | 26022 | TTHERM_00290900 | 4e-27 (Rab6 Hs) | 243 |
