# Supplementary material for: Macronuclear Genome Sequence of the Ciliate Tetrahymena thermophila, a Model Eukaryote
Source: PLoS Biol. 2006 Aug 29;4(9):e286. doi: 10.1371/journal.pbio.0040286 (PMC1557398; doi:10.1371/journal.pbio.0040286)
Supplement: Supplementary file 23 [file pbio.0040286.st016.doc]

Table S16. Testing different gene finder parameterizations.

| **Parameterization** | **Nucleotide**  **Accuracy** | **Splice Site Sensitivity** | **Splice Site Specificity** | **Exon Sensitivity** | **Exon Specificity** |
| --- | --- | --- | --- | --- | --- |
| **"hybrid"** | 95% | 66% | 49% | 71% | 32% |
| **"long-ORFs"** | 79% | 29% | 19% | 10% | 10% |
