# Supplementary material for: Macronuclear Genome Sequence of the Ciliate Tetrahymena thermophila, a Model Eukaryote
Source: PLoS Biol. 2006 Aug 29;4(9):e286. doi: 10.1371/journal.pbio.0040286 (PMC1557398; doi:10.1371/journal.pbio.0040286)
Supplement: Supplementary file 24 [file pbio.0040286.st017.doc]

Table S17. The 50 longest 100% identical repeats in the T. thermophila genome assembly

|  | **First Repeat Copy** | | **Second Repeat Copy** | | **percentage amino acid coding** |
| --- | --- | --- | --- | --- | --- |
| **Repeat length (bp)** | **Scaffold ID** | **Start position** | **Scaffold ID** | **Start position** |  |
| 5795 | 8254462 | 2563 | 8254629 | 107305 | 56.37% |
| 5729 | 8254462 | 2212 | 8254538 | 2 | 52.56% |
| 5378 | 8254629 | 107305 | 8254538 | 353 | 56.54% |
| 4898 | 8254357 | 598 | 8254357 | 10469 | 84.29% |
| 4890 | 8254134 | 5492 | 8253904 | 504 | 81.49% |
| 4761 | 8254480 | 0 | 8254006 | 165058 | 86.27% |
| 4741 | 8254077 | 83683 | 8254480 | 12937 | 73.59% |
| 4703 | 8254077 | 83442 | 8254480 | 334 | 81.91% |
| 4462 | 8254480 | 575 | 8254480 | 12937 | 80.93% |
| 4427 | 8254077 | 83442 | 8254006 | 165058 | 86.70% |
| 4375 | 8253803 | 3605 | 8254139 | 31 | 63.87% |
| 4186 | 8254480 | 12937 | 8254006 | 165058 | 85.93% |
| 4109 | 8254703 | 4989 | 8254787 | 110 | 88.56% |
| 3734 | 8254787 | 0 | 8254787 | 13464 | 86.80% |
| 3624 | 8254703 | 4989 | 8254787 | 13574 | 89.20% |
| 3403 | 8254442 | 14407 | 8254525 | 52687 | 42.45% |
| 3273 | 8254442 | 14537 | 8254479 | 0 | 33.41% |
| 3273 | 8254479 | 0 | 8254525 | 52687 | 40.15% |
| 3088 | 8254442 | 0 | 8254525 | 53316 | 44.79% |
| 3025 | 8254816 | 27201 | 8253967 | 742 | 76.10% |
| 2985 | 8254366 | 4829 | 8254583 | 385 | 43.92% |
| 2931 | 8254719 | 6317 | 8254737 | 247214 | 90.40% |
| 2916 | 8254057 | 0 | 8253825 | 0 | 75.79% |
| 2887 | 8254027 | 828 | 8254246 | 676 | 49.08% |
| 2774 | 8254442 | 314 | 8254442 | 14407 | 44.12% |
| 2644 | 8254442 | 444 | 8254479 | 629 | 41.36% |
| 2562 | 8253970 | 699 | 8253976 | 10871 | 97.74% |
| 2540 | 8253970 | 13823 | 8253976 | 11009 | 96.50% |
| 2536 | 8254027 | 1157 | 8254031 | 1490 | 46.92% |
| 2536 | 8254031 | 1490 | 8254246 | 1005 | 46.92% |
| 2482 | 8254057 | 665 | 8254057 | 11559 | 86.50% |
| 2424 | 8253970 | 837 | 8253970 | 13823 | 97.61% |
| 2251 | 8254057 | 11559 | 8253825 | 665 | 85.12% |
| 2246 | 8254710 | 14484 | 8254786 | 436925 | 63.13% |
| 2244 | 8254686 | 0 | 8254712 | 42797 | 65.86% |
| 2148 | 8254654 | 297251 | 8254572 | 183580 | 100.05% |
| 2142 | 8254493 | 0 | 8254501 | 5489 | 20.82% |
| 2086 | 8254657 | 0 | 8254592 | 402573 | 70.30% |
| 2082 | 8254520 | 222 | 8254549 | 248781 | 59.13% |
| 2066 | 8254697 | 81 | 8254756 | 2450 | 89.57% |
| 2040 | 8254057 | 3718 | 8253825 | 4330 | 96.27% |
| 2040 | 8254287 | 1232 | 8254501 | 5952 | 21.23% |
| 1970 | 8254820 | 55670 | 8254820 | 71224 | 48.07% |
| 1931 | 8254332 | 38062 | 8254332 | 48704 | 51.22% |
| 1881 | 8254816 | 61194 | 8254139 | 4877 | 17.60% |
| 1879 | 8254727 | 10406 | 8254770 | 235 | 60.51% |
| 1875 | 8254653 | 352040 | 8254653 | 366611 | 0.00% |
| 1834 | 8254658 | 1574 | 8254817 | 3598 | 70.09% |
| 1829 | 8253843 | 126818 | 8254332 | 37699 | 50.77% |
| 1828 | 8254271 | 19666 | 8254520 | 458 | 64.14% |
